# Routine‐data‐compatible quality indicators for the ambulatory care of osteoarthritis of the knee and hip: A systematic review

**DOI:** 10.1002/ksa.12614

**Published:** 2025-02-13

**Authors:** Tobias Bock, Ronja Flemming, Philip Bammert, Rüdiger von Eisenhart‐Rothe, Michael T. Hirschmann, Leonie Sundmacher

**Affiliations:** ^1^ Department of Health Economics Technical University of Munich Munich Germany; ^2^ Department of Orthopaedics and Sport Orthopaedics, University Hospital rechts der Isar Technical University Munich Munich Germany; ^3^ Department of Orthopaedic Surgery and Traumatology, Kantonsspital Baselland (Bruderholz, Liestal, Laufen) University of Basel Basel Switzerland

**Keywords:** ambulatory care, hip, knee, osteoarthritis, quality indicators, routine data

## Abstract

**Purpose:**

Germany has high rates of total joint arthroplasty for osteoarthritis of the knee and hip. Ambulatory health interventions can affect the progression of these conditions and the need for total joint arthroplasty. Quality indicators and guideline recommendations facilitate the transparent measurement and demonstration of care quality. Therefore, a systematic literature review of quality indicators and guideline recommendations for ambulatory care of osteoarthritis of the knee and hip before total joint arthroplasty was conducted, focusing on those that could be quantified using routine data from German statutory health insurers.

**Methods:**

Five electronic databases for quality indicators and guidelines published between 2000 and 2021 related to the ambulatory management of osteoarthritis of the knee and hip before total joint arthroplasty were searched. Two reviewers independently selected and appraised the quality of the studies. To synthesise a routine‐data‐compatible set of quality indicators, similarities and differences among existing quality indicator sets and guideline recommendations were identified and resolved.

**Results:**

This systematic search yielded 10,841 potentially relevant records, leading to the identification of 20 sets of quality indicators and 35 guidelines with measures quantifiable using routine data. The present evidence synthesis produced 24 routine‐data‐compatible process quality indicators related to the type, order or frequency of musculoskeletal appointments, diagnostic imaging procedures, referrals to physical therapists and pharmaceutical prescriptions.

**Conclusion:**

The synthesised set of routine‐data‐compatible quality indicators can provide a resource‐saving tool for offering individual feedback to physicians on the processes involved in the ambulatory management of osteoarthritis of the knee and hip. Engaging in interdisciplinary discussions on variations in quality indicator outcomes could contribute to improving interdisciplinary physician collaboration in ambulatory care for these conditions.

**Level of Evidence:**

Level III.

**Abbreviations**
AAOS: American Academy of Orthopaedic Surgeons
ACR: American College of Rheumatology
AGREE: Appraisal of Guidelines for Research and Evaluation
AIRE: Appraisal of Indicators through Research and Evaluation
APTA: American Physical Therapy Association
aQua‐Institute: Institute for Applied Quality Improvement and Research in Health Care GmbH
AWMF: Association of the Scientific Medical Societies in Germany
CT: computer tomography
CV: cardiovascular
DVA: Department of Veterans Affairs
ESCEO: European Society for Clinical and Economic Aspects of Osteoporosis, Osteoarthritis and Musculoskeletal Diseases
EULAR: European League Against Rheumatism
GEDA: German Health Update
GI: gastrointestinal
IA: intra‐articular
IGeL: individual health services
KNGF: The Royal Dutch Society for Physiotherapy
LOE: level of evidence
MQIC: Michigan Quality Improvement Consortium Guideline
MRI: magnetic resonance imaging
NHG: Dutch College of General Practitioners
NHMRC: National Health and Medical Research Council
NICE: National Institute for Health and Care Excellence
NSAID: nonsteroidal anti‐inflammatory drugs
OARSI: Osteoarthritis Research Society International
OECD: Organisation for Economic Cooperation and Development
PANLAR: Pan‐American League of Associations for Rheumatology
PCPI: Physician Consortium for Performance Improvement
PPI: proton pump inhibitors
PT: physical therapist
QI: quality indicator
RAM: RAND/UCLA Appropriateness Method
SFR: French Society of Rheumatology
SHI: statutory health insurers
SIR: Italian Society for Rheumatology
SOR: strength of recommendation
TENS: transcutaneous electrical nerve stimulation
TJA: total joint arthroplasty
WHO: World Health Organization

## INTRODUCTION

With over 500 million prevalent cases worldwide in 2019 and a rapid increase in incidence in recent decades, osteoarthritis remains a major public health concern, imposing a heavy burden on healthcare systems [55]. According to the 2019/2020 survey of the German Health Update (GEDA), 17.1% of German adults aged 18 years or older reported having osteoarthritis in the previous 12 months. Owing to the ageing population, the number of cases is expected to rise substantially in the future [32].

Total joint arthroplasty (TJA) is recognised as an effective procedure to reduce pain, restore joint functionality and reduce the risk of developing comorbidities. Between 2009 and 2019, pre‐COVID‐19 pandemic knee TJA rates increased by 35% and hip TJA rates by 22% on average across more than 32 member countries of the Organisation for Economic Cooperation and Development (OECD) [54]. In 2021, average age‐standardised rates of 119 knee TJA and 172 hip TJA per 100,000 population were reported across more than 32 OECD countries with comparable data. However, the rates in Germany were markedly higher, with 201 knee TJA and 301 hip TJA per 100,000 population that same year [55].

Assumptions about the causes of the high TJA rates focus on whether they reflect the needs of an ageing population, improved access to healthcare or overutilisation of health resources. With regard to overutilisation, misguided financial incentives from the diagnosis‐related group payment system and evidence of large geographical disparities have been discussed [41, 66]. To reduce the disease burden for individuals and healthcare systems, TJA should generally be performed only after ambulatory health interventions have proven ineffective [48]. From a health policy and research perspective, it is thus important to assess which ambulatory health interventions have been used before TJA and to what extent.

Although national clinical practice guidelines exist in Germany [19, 20, 21], a structured and coordinated care pathway for osteoarthritis treatment has not yet been developed. The use of quality indicators (QIs) could be instrumental in addressing this gap. QIs provide essential insights into healthcare delivery and can help identify areas in which care pathways can be improved. QIs establish norms for different aspects of care, thereby facilitating the transparent measurement and demonstration of care quality. However, for their practical implementation in the external quality assurance of health interventions, it is necessary to explore how QIs can use routine data for definitive quality measurements [34].

Existing QI sets predominantly focus on the discharge management of patients after TJA, or they require clinical data collection [39, 70]. Currently, no set of QIs specifically focuses on ambulatory care of osteoarthritis of the knee and hip before TJA and can be quantified using routine data from statutory health insurers (SHIs) in Germany. This systematic literature review aimed to identify all existing QIs and guideline recommendations suitable for QI derivation [33] for ambulatory care of osteoarthritis of the knee and hip before TJA that are quantifiable using routine data from German SHIs.

## MATERIALS AND METHODS

This systematic review is part of the first phase of the broader MobilE‐ARTH study (German Clinical Trials Register, ID: DRKS00027516). MobilE‐ARTH aims to improve the quality of evidence‐based ambulatory care of osteoarthritis of the knee and hip before TJA. The three‐phase study design comprises development, pilot testing and evaluation of routine‐data‐compatible QIs. In accordance with the RAND/UCLA Appropriateness Method (RAM) [28], the development phase consists of a systematic literature review followed by a Delphi consensus process.

### Search strategy

A comprehensive search strategy was applied across five electronic databases: Pubmed, Cochrane Library, Web of Science, Scopus and PEDro. The search terms and detailed search strategy are provided in Appendix 1. In addition, the reference lists of eligible QI sets and guidelines were manually screened for further relevant publications. Websites of organisations and institutions identified during the screening process for relevant records were also reviewed.

### Selection of eligible records

All records identified were imported to the database searches into the online systematic review management platform Rayyan® and duplicates were removed. Then, a two‐step process was followed to screen and select the remaining records. In the first step, two authors (T. B. and R. F.) independently screened all titles and abstracts for eligibility. Records were eligible for full‐text review if they met all of the following criteria:

– The record was published between 1 January 2000 and 17 June 2021 in English or German.
– The record reported QIs or guideline recommendations for adults (≥18 years) with osteoarthritis of the knee and hip.
– The record focused on diagnostic procedures or conservative treatments before TJA.

The two authors resolved disagreements in the selection process through consensus. The agreement level between both reviewers was assessed using Cohen's κ. In the second step, one author (T. B.) retrieved and reviewed full‐text versions of the eligible records. These publications were eligible for inclusion if they met all of the following criteria:

– The type and scope of the ambulatory care reported were covered by SIH in Germany.
– If the publication reported QIs, it had to be the primary source of the QIs or an adaptation of already existing QIs into another healthcare setting.
– If the publication was a guideline, it had to be the most recent version of this guideline.
– The publication was available in full length.

### Assessment of methodological quality

After the full‐text review, two authors (T. B. and P. B.) independently appraised the methodological quality of the included QI sets and guidelines. However, the results of this appraisal were not a decisive factor in selecting the QIs and guideline recommendations for subsequent analysis and synthesis. This approach was chosen to ensure consideration of the widest range of options that could be quantified with routine data.

To appraise the methodological quality of the included QI sets, the two authors used the Appraisal of Indicators through Research and Evaluation (AIRE) instrument [17]. It consists of 20 items grouped into four quality domains, with each item rated on a 4‐point scale (1 = ‘strongly disagree’; 4 = ‘strongly agree’). Instead of scoring each QI individually, items were summed per domain for the total QI set because publications usually provide information on the scientific evidence and development of the QI set as a whole [59]. Standardised domain scores were calculated according to the following formula: (total score − minimum possible score)/(maximum possible score − minimum possible score) × 100%. A score of 50% or higher indicates a higher methodological quality in a domain. It is important to note that domain scores are independent and should not be aggregated into a single overall quality score.

To appraise the methodological quality of the included guidelines, the two authors used the Appraisal of Guidelines for Research and Evaluation (AGREE) II instrument [14]. It comprises 23 items grouped into six domains evaluating the development of guidelines. Items are rated on a 7‐point scale (1 = ‘strongly disagree’; 7 = ‘strongly agree’). Similar to the AIRE instrument, scores for individual items in a domain are totalled and then expressed as a percentage of the maximum possible score for that domain. The AGREE II instrument does not provide guidance for interpreting domain scores and thus whether a guideline can be recommended for use. To provide an additional classification of guidelines based on the individual domain scores, the classification system of a previous quality appraisal [38] was used to classify the guidelines included in the review into three levels:

– Level A: high quality, recommended (≥4 domains were scored >60%, including the domain ‘rigour of development’).
– Level B: medium quality, recommended with modification (≥4 domains were scored ≥30%).
– Level C: low quality, not recommended (≥3 of domains were scored <30%).

### Data collection, analysis and synthesis

The information extracted from the included publications comprised the title, first author or publishing institution's name, publication year, country and target population. Additionally, extraction data included information on the approach used to synthesise evidence and achieve consensus when developing the QIs, as well as the grades for the level of evidence (LOE) and strength of recommendation (SOR) for each guideline recommendation. This approach aimed to facilitate an individual weighting of QIs and guideline recommendations.

To synthesise a new QI set, the included QIs and guideline recommendations were analysed for their relevance to ambulatory care of osteoarthritis of the knee and hip before TJA in Germany and their potential for quantification using routine data. Following Donabedian's framework of quality of medical care [23], each QI was classified as a structure, process or outcome measure. This classification aimed to determine which quality dimension could be quantified using routine data and to what extent.

To provide a more detailed overview by intervention type, QIs and guideline recommendations were subsequently categorised into six intervention categories: musculoskeletal appointments, diagnostic imaging, exercise therapy, physical therapy, medication and TJA preconditions. Within these categories, the QIs and guideline recommendations were assigned to specific ambulatory health interventions.

Finally, the similarities and differences among analogous QIs and analogous guideline recommendations were analysed and resolved and then synthesised into a single, consolidated set of QIs. First, the definitions and descriptions of each QI and guideline recommendation were listed side by side. These descriptions included detailed information on the delivery of each intervention, such as the frequency of a particular treatment or the amount of medication to be prescribed [33]. Then, all variations in the delivery of each specific intervention were identified and contents that did not vary were condensed. Subsequently, the descriptions of QIs and guideline recommendations for their potential to provide valid measures and information about the quality of care structures, processes or outcomes using routine data from German SHIs were assessed. A publication's original description of a QI or guideline recommendation was adopted if routine data compatibility was possible. Where necessary and feasible, QIs or guideline recommendations were modified so that they could be used with routine data.

## RESULTS

### Search results

**Figure 1:**
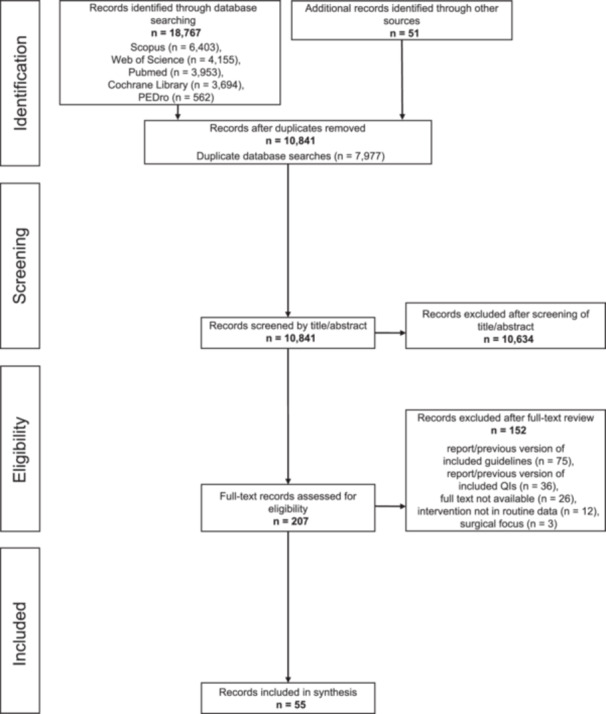
Flow chart of the study selection.

The database searches yielded 10,790 potentially relevant records following the removal of duplicates. The manual search identified 51 additional records of potential relevance. After screening the titles and abstracts, 207 were deemed relevant for a full‐text review. A Cohen's κ of 0.81 for inter‐rater reliability indicated almost perfect agreement between the two authors (T. B. and R. F.) [42]. A total of 181 of these records were obtained as full‐text publications. Subsequently, 12 of these publications were excluded as they involved interventions not covered by routine data, and three further publications because they had a surgical focus. The remaining 166 publications consisted of 56 that reported QIs and 110 that reported guideline recommendations for ambulatory care of osteoarthritis of the knee and hip. Among the QI‐reporting publications, 36 were excluded because they used QIs from the remaining 20 included QI sets without making any adaptations. Among the guideline‐reporting publications, 75 were excluded because they were either a previous version of one of the remaining 35 included guidelines or used recommendations from the included guidelines for further research. This yielded a total of 55 publications for inclusion in the synthesis process. Figure 1 gives a PRISMA flowchart of the study selection process and search results.

### Characteristics of included QI sets

Table 1 shows the characteristics of the QI sets, which were published in North America [3, 6, 8, 24, 46, 47, 50, 65, 68, 82] or Europe [26, 31, 35, 39, 56, 58, 69, 72, 74, 76] between 2000 and 2018. These either focused on populations with OA‐related diseases [3, 8, 24, 26, 31, 35, 39, 46, 50, 56, 58, 69, 72, 74] or formed part of a holistic assessment of health status in elderly patients [6, 47, 65, 68, 76, 82]. Three publications developed QI sets using a systematic review for evidence synthesis [26, 39, 46] and 10 publications developed QI sets using a RAM‐modified Delphi consensus [6, 8, 24, 31, 39, 46, 47, 50, 69, 72]. Five publications adapted existing QIs to another healthcare setting [65, 68, 69, 76, 82].

**Table 1:** Characteristics of included QI sets.

First author/institution	Year	Country	Osteoarthritis target population	Evidence synthesis	Consensus method	Number of QIs	Dimension of quality		
							Structure	Process	Outcome
Østerås N [56]	2018	Norway	Patients with osteoarthritis	Literature review, not specified to be systematic	Refinement of QIs in expert panels and patient interviews	16		16	
Barber CE [8]	2015	Canada	Patients with moderate to severe osteoarthritis	Integrative review	RAM‐modified Delphi consensus	18	3	13	2
Doubova SV [24]	2015	Mexico	Patients with osteoarthritis of the knee/hip (≥19 years)	Literature review, not specified to be systematic	RAM‐modified Delphi consensus	6		6	
Grypdonck L [31]	2014	Belgium	Patients with osteoarthritis of the knee	Literature review, not specified to be systematic	RAM‐modified Delphi consensus	22		22	
Peter WF [58]	2013	The Netherlands	Patients with osteoarthritis of the knee/hip in physiotherapy care	QIs based on earlier published guideline recommendations [57]	Recommendations rated by an expert panel of physical therapists	19		19	
EUMUSC.net [26]	2012	n/s (Europe)	Patients with osteoarthritis(≥18 years)	Systematic review for the development of standards of care	Translation of standards of care into QIs and refinement in expert panels	12		9	3
Wierenga PC [76]	2011	The Netherlands	Elderly hospitalised patients	Adaptation of earlier published QIs [45] for elderly hospitalised patients	Delphi process with a multidisciplinary expert panel	2		2	
Jansen MJ [35]	2010	The Netherlands	Patients with osteoarthritis of the knee/hip in physiotherapy care	QIs derived from physiotherapy guideline [75]	QIs independently assessed by two people	17		9	8
Kleudgen S [39]	2009	Germany	Patients with osteoarthritis	Systematic review	RAM‐modified Delphi consensus	4		4	
Zingmond DS [82]	2009	US	Community dwellers in nursing homes aged 65 years and older	Adaptation of earlier published QIs [65] for use with routine data	n/s	9		9	
MacLean CH [46]	2007	US	Vulnerable elders with osteoarthritis	Systematic review	RAM‐modified Delphi consensus	13		13	
Smith KL [68]	2007	US	Homebound elderly patients in home‐based primary care	Adaptation of earlier published QIs [45, 65] for use in home‐based primary care	Modified Delphi process with a panel of nursing home experts	7		7	
PCPI [3]	2006	US	Patients with osteoarthritis(≥21 years)	n/s	n/s	7		7	
Asch SM [6]	2004	US	Patients in outpatient and inpatient care for acute and chronic conditions	Literature review, not specified to be systematic	RAM‐modified Delphi consensus	3		3	
Saliba D [65]	2004	US	Vulnerable elders in US nursing homes	Adaptation of earlier published QIs [45] for use in US nursing homes	Modified Delphi process with a panel of nursing home experts	7		7	
Steel N [69]	2004	UK	Elderly patients aged 65 years or older in primary and secondary care	Adaptation of earlier published QIs [45] for use in UK healthcare system	RAM‐modified Delphi consensus	6		6	
Vandenberghe H [74]	2004	Belgium	Patients with osteoarthritis(≥60 years) in general practice	n/s	n/s	5		5	
McGlynn EA [47]	2003	US	Adults living in 12 metropolitan areas in the US	Literature review, not specified to be systematic	RAM‐modified Delphi consensus	3		3	
Underwood M [72]	2002	UK	Patients with osteoarthritis in general practice	Literature review, not specified to be systematic	RAM‐modified Delphi consensus	4		4	
Moore A [50]	2000	US	Patients with osteoarthritis	Literature review, not specified to be systematic	RAM‐modified Delphi consensus	7		7	

Abbreviations: n/s, not specified; PCPI, Physician Consortium for Performance Improvement; QI, quality indicator; RAM, RAND Appropriateness Method; UK, United Kingdom; US, United States.

After the included QIs were classified as structure, process or outcome measures, it was determined that the only QIs for ambulatory care of osteoarthritis of the knee and hip that could be quantified using routine data were process QIs (see Appendix 2). In the AIRE quality appraisal (see Appendix 3), 14 QI sets scored 50% or higher in all four domains [3, 6, 8, 24, 26, 31, 39, 46, 47, 58, 65, 68, 69, 76].

The QIs could be assigned to 15 distinct ambulatory health interventions that were quantifiable using routine data from German SHIs (see Table 2). The majority of QI sets contained QIs related to interventions performed during a physician visit for musculoskeletal disorders to assess disease progression [3, 6, 8, 26, 35, 39, 46, 47, 50, 56, 58, 65, 68, 69, 82]. Diagnostic imaging procedures were addressed in one QI set [31]. The beneficial influence of exercise therapy was mentioned frequently throughout the QI sets [3, 6, 24, 26, 31, 35, 46, 47, 50, 56, 58, 65, 68, 69, 82]. Physical therapy interventions encompassed transcutaneous electrical nerve stimulation (TENS) [35], massage therapy [35], the application of orthoses/braces [26, 31] and walking aids [26, 46, 56]. Interventions involving nonprescription medications could be found in a large majority of QI sets, including initial pain treatment with paracetamol [3, 6, 24, 31, 39, 46, 47, 50, 56, 65, 68, 69, 72, 74, 76, 82], subsequent pain treatment with oral nonsteroidal anti‐inflammatory drugs (NSAIDs) [3, 31, 72, 74], oral NSAID treatment for specific comorbidities [3, 24, 26, 31, 46, 68] and the consideration of gastrointestinal prophylaxis when using oral NSAIDs [3, 24, 31, 46, 72, 82]. Two QI sets included interventions involving the prescription of opioids [31, 56] or the injection of corticosteroids [56] in patients with advanced osteoarthritis of the knee or hip. Some QI sets included QIs related to interventions assessing the need for TJA in patients with end‐stage osteoarthritis of the knee or hip [26, 31, 46, 56, 69, 72].

**Table 2:** Routine‐data‐compatible ambulatory health interventions that are addressed in included QI sets.

Intervention category	Ambulatory health intervention quantifiable in routine data	Østerås N [56]	Barber CE [8]	Doubova SV [24]	Grypdonck L [31]	Peter WF [58]	EUMUSC.net [26]	Wierenga PC [76]	Jansen MJ [35]	Kleudgen S [39]	Zingmond DS [82]	MacLean CH [46]	Smith KL [68]	PCPI [3]	Asch SM [6]	Saliba D [65]	Steel N [69]	Vandenberghe H [74]	McGlynn EA [47]	Underwood M [72]	Moore A [50]
Musculo‐skeletal appointment	Physician visit for musculoskeletal disorders	**✓**	**✓**			**✓**	**✓**		**✓**	**✓**	**✓**	**✓**	**✓**	**✓**	**✓**	**✓**	**✓**		**✓**		**✓**
Diagnostic imaging	Radiographic assessment (prior to CT, MRI, sonography)				**✓**																
Exercise therapy	Exercise therapy/referral to PT	**✓**		**✓**	**✓**	**✓**	**✓**		**✓**		**✓**	**✓**	**✓**	**✓**	**✓**	**✓**	**✓**		**✓**		**✓**
Physical therapy	TENS								**✓**												
	Massage								**✓**												
	Orthoses/braces				**✓**		**✓**														
	Walking aids	**✓**					**✓**					**✓**									
Medication	Paracetamol (initial medication)	**✓**		**✓**	**✓**			**✓**		**✓**	**✓**	**✓**	**✓**	**✓**	**✓**	**✓**	**✓**	**✓**	**✓**	**✓**	**✓**
	Oral NSAIDs				**✓**									**✓**				**✓**		**✓**	
	Oral NSAID treatment with caution for comorbidities (GI/CV/renal)			**✓**	**✓**		**✓**					**✓**	**✓**	**✓**							
	Oral NSAID treatment with concomitant PPI/misoprostol in case of Gl risk factors			**✓**	**✓**						**✓**	**✓**		**✓**						**✓**	
	Weak opioids	**✓**																			
	Strong opioids				**✓**																
	Corticosteroid injection	**✓**																			
TJA preconditions	Referral to orthopaedic surgeon if conservative therapy failed	**✓**			**✓**		**✓**					**✓**					**✓**			**✓**	

Abbreviations: ✓, ambulatory health intervention is addressed within the included QI sets; CT, computer tomography; CV, cardiovascular; GI, gastrointestinal; MRI, magnetic resonance imaging; NSAID, nonsteroidal anti‐inflammatory drugs; PCPI, Physician Consortium for Performance Improvement; PPI, proton pump inhibitors; PT, physical therapist; QI, quality indicator; TENS, transcutaneous electrical nerve stimulation; TJA, total joint arthroplasty.

### Characteristics of included guidelines

Table 3 gives an overview of the included guideline recommendations that could be used to derive additional QIs. The synthesis drew upon guidelines from 24 organisations, institutions, research societies or scientific groups from North and South America [1, 2, 10, 11, 12, 13, 16, 18, 40, 49, 60, 62], Europe [4, 9, 15, 19, 20, 21, 27, 30, 36, 37, 52, 61, 63, 64, 67, 73, 80], Asia [71, 78, 79, 81], Australia [51] or with an international alignment [7]. Seven guidelines were published before 2016 [9, 13, 27, 37, 60, 63, 80], and the remaining 28 were published after 2016. The grading systems for LOE and SOR varied considerably among the guidelines (see Appendix 4).

**Table 3:** Characteristics of included guidelines.

First author/institution	Year	Country	Osteoarthritis target population (focus)	Number of recommendations
AWMF [21]	2021	Germany	Patients with osteoarthritis of the hip	32
AAOS [2]	2021	US	Patients with osteoarthritis of the knee	29
MQIC [49]	2021	US	Patients with osteoarthritis	15
Yeap SS [79]	2021	Malaysia	Patients with osteoarthritis of the knee	9
ACR [40]	2020	US	Patients with osteoarthritis of the knee/hip/hand	45
DVA [18]	2020	US	Patients with osteoarthritis of the knee/hip	18
KNGF [73]	2020	The Netherlands	Patients with osteoarthritis of the knee/hip (exercise therapy)	11
NICE [52]	2020	UK (England, Wales)	Patients with osteoarthritis	43
SFR [67]	2020	France	Patients with osteoarthritis of the knee (medication)	11
Zhang Z [81]	2020	China	Patients with osteoarthritis	16
AWMF [20]	2019	Germany	Patients with osteoarthritis of the hip	56
ESCEO [15]	2019	n/s (Europe)	Patients with osteoarthritis of the knee	15
Jarl G [36]	2019	Scandinavia	Patients with osteoarthritis of the knee (orthotic treatment)	11
OARSI [7]	2019	n/s (international)	Patients with osteoarthritis of the knee/hip/hand	22
SIR [4]	2019	Italy	Patients with osteoarthritis of the knee/hip/hand	16
Yabuki S [78]	2019	South Korea	Patients with osteoarthritis/OA/chronic low back pain (medication)	28
AWMF [19]	2018	Germany	Patients with osteoarthritis of the knee	44
EULAR [30]	2018	n/s (Europe)	Patients with osteoarthritis/inflammatory arthritis	10
EULAR [61]	2018	n/s (Europe)	Patients with osteoarthritis/inflammatory arthritis (exercise therapy)	10
NHMRC [51]	2018	Australia	Patients with osteoarthritis of the knee/hip	64
TLAR [71]	2018	Turkey	Patients with osteoarthritis of the knee	11
AAOS [1]	2017	US	Patients with osteoarthritis of the hip	18
APTA [16]	2017	US	Patients with osteoarthritis of the hip	15
EULAR [64]	2017	n/s (Europe)	Patients with osteoarthritis of the peripheral joint (imaging)	7
Ottawa Panel [11]	2017	Canada	Patients with osteoarthritis of the knee (exercise therapy)	26
Ottawa Panel [10]	2017	Canada	Patients with osteoarthritis of the knee (exercise therapy)	5
Ottawa Panel [12]	2016	Canada	Patients with osteoarthritis of the hip (exercise therapy)	16
PANLAR [62]	2016	n/s (South America)	Patients with osteoarthritis of the knee/hip/hand	51
EULAR [27]	2013	n/s (Europe)	Patients with osteoarthritis of the knee/hip	11
NHG [9]	2008	The Netherlands	Patients with nontraumatic knee problems	25
EULAR [80]	2005	n/s (Europe)	Patients with osteoarthritis of the hip	10
Ottawa Panel [13]	2005	Canada	Patients with osteoarthritis (exercise therapy)	15
MOVE consensus [63]	2005	UK	Patients with osteoarthritis of the knee/hip (exercise therapy)	10
EULAR [37]	2003	n/s (Europe)	Patients with osteoarthritis of the knee	10
Philadelphia Panel [60]	2001	US	Patients with osteoarthritis of the knee	10

Abbreviations: AAOS, American Academy of Orthopaedic Surgeons; ACR, American College of Rheumatology; APTA, American Physical Therapy Association; AWMF, Association of the Scientific Medical Societies in Germany; DVA, Department of Veterans Affairs; ESCEO, European Society for Clinical and Economic Aspects of Osteoporosis, Osteoarthritis and Musculoskeletal Diseases; EULAR, European League Against Rheumatism; KNGF, The Royal Dutch Society for Physiotherapy; MQIC, Michigan Quality Improvement Consortium Guideline; n/s, not specified; NHG, Dutch College of General Practitioners; NHMRC, National Health and Medical Research Council; NICE, National Institute for Health and Care Excellence; OARSI, Osteoarthritis Research Society International; PANLAR, Pan‐American League of Associations for Rheumatology; SFR, French Society of Rheumatology; SIR, Italian Society for Rheumatology; TLAR, Turkish League Against Rheumatism; UK, United Kingdom; US, United States.

In the quality appraisal using AGREE II (see Appendix 3), 16 guidelines were classified as Level A [1, 2, 4, 7, 16, 18, 21, 27, 30, 40, 51, 61, 64, 73, 81], 17 guidelines as Level B [10, 11, 12, 13, 15, 19, 20, 36, 37, 60, 62, 63, 67, 71, 78, 79, 80] and two guidelines as Level C [9, 49].

Each of the guideline recommendations was assigned to one of 21 ambulatory health interventions that were quantifiable using routine data (see Table 4). Recommendations stating the need for a comprehensive clinical assessment as part of a physician visit for musculoskeletal disorders were contained in numerous guidelines [4, 9, 16, 19, 20, 21, 27, 30, 37, 49, 52, 61, 64, 78, 79, 80, 81]. Many guidelines also recommended radiographic assessment of the affected joint to guide needs‐oriented therapy [4, 9, 18, 19, 20, 21, 37, 64, 80]. Almost every guideline contained a recommendation in favour of some type of supervised exercise programme [1, 2, 4, 7, 9, 10, 11, 12, 13, 15, 16, 18, 19, 20, 27, 30, 37, 40, 49, 51, 52, 60, 61, 62, 63, 71, 73, 78, 79, 80, 81]. Apart from the provision of walking aids [2, 4, 7, 9, 16, 27, 30, 37, 40, 51, 52, 62, 80], however, there was no clear consensus in the guidelines concerning the use of physical therapy alternatives like TENS [2, 4, 9, 18, 19, 20, 40, 51, 52, 60, 62, 71, 73], neuromuscular electrical stimulation [2, 19, 60], massage therapy [2, 9, 18, 20, 40, 51, 60, 73, 81], acupuncture [2, 4, 9, 18, 19, 20, 40, 51, 52, 81], orthoses/braces [2, 16, 18, 30, 36, 37, 40, 51, 52, 62] or shoes/insoles [2, 4, 9, 27, 30, 36, 37, 40, 51, 52, 62, 80]. With one exception [19], the guidelines recommended paracetamol for initiating pharmacological pain management [2, 4, 9, 15, 18, 37, 40, 49, 51, 52, 62, 67, 71, 78, 80]. In addition, there was a broad consensus that topical NSAIDs should be prescribed before oral NSAIDs [2, 4, 7, 9, 15, 18, 19, 37, 40, 51, 52, 62, 67, 71, 78, 81]. There was also a broad consensus on the consideration of oral NSAIDs in the treatment of specific comorbidities [4, 7, 9, 19, 49, 51, 52, 62, 67, 71, 78] and on the use of gastrointestinal prophylaxis when prescribing oral NSAIDs [4, 7, 19, 49, 52, 62, 78, 81]. There were inconsistent recommendations with regard to prescribing opioids as an alternative analgesic option in patients with advanced osteoarthritis of the knee and hip [2, 4, 9, 15, 18, 19, 20, 37, 40, 49, 51, 52, 62, 67, 71, 78, 79, 80, 81]. The use of intra‐articular (IA) corticosteroids in inflammatory osteoarthritis of the knee or hip [1, 2, 4, 7, 9, 15, 18, 19, 21, 37, 40, 51, 52, 62, 67, 71, 80, 81] and the consideration of duloxetine [7, 15, 18, 40, 51, 62, 67, 71, 78, 81] as alternative options in patients with chronic pain were recommended repeatedly. Patients with end‐stage osteoarthritis of the knee or hip, who are resistant to conservative treatments, are recommended to be referred to an orthopaedic surgeon for an assessment for TJA [4, 9, 15, 19, 21, 37, 52, 62, 71, 79, 80, 81]. When considering TJA, there should be documentation that the patient has been offered a treatment combining appropriate pharmacological and nonpharmacological therapy options [4, 21, 37, 52, 71, 78, 79, 80].

**Table 4:** Routine‐data‐compatible ambulatory health interventions that are addressed in recommendations of included guidelines.

Intervention category	Ambulatory health intervention quantifiable in routine data	AWMF [21]	AAOS [2]	MQIC [49]	Yeap SS [79]	ACR [40]	DVA [18]	KNGF [73]	NICE [52]	SFR [67]	Zhang Z [81]	AWMF [20]	ESCEO [15]	Jarl G [36]	OARSI [7]	SIR [4]	Yabuki S [78]	AWMF [19]	EULAR [30]	EULAR [61]	NHMRC [51]
Musculo‐skeletal appointment	Physician visit for musculoskeletal disorders	**✓**		**✓**	**✓**				**✓**		**✓**	**✓**				**✓**	**✓**	**✓**	**✓**	**✓**	
Diagnostic imaging	Radiographic assessment (prior to CT, MRI, sonography)	**✓**					**✓**					**✓**				**✓**		**✓**			
Exercise therapy	Exercise therapy/referral to PT		**✓**	**✓**	**✓**	**✓**	**✓**	**✓**	**✓**		**✓**	**✓**	**✓**		**✓**	**✓**	**✓**	**✓**	**✓**	**✓**	**✓**
Physical therapy	TENS		**✓**			**✓**	**✓**	**✓**	**✓**			**✓**				**✓**		**✓**			**✓**
	NMES		**✓**															**✓**			
	Massage		**✓**			**✓**	**✓**	**✓**			**✓**	**✓**									**✓**
	Acupuncture		**✓**			**✓**	**✓**		**✓**		**✓**	**✓**				**✓**		**✓**			**✓**
	Orthoses/braces		**✓**			**✓**	**✓**		**✓**					**✓**					**✓**		**✓**
	Walking aids		**✓**			**✓**			**✓**						**✓**	**✓**			**✓**		**✓**
	Shoes/insoles		**✓**			**✓**			**✓**					**✓**		**✓**			**✓**		**✓**
Medication	Paracetamol (initial medication)		**✓**	**✓**		**✓**	**✓**		**✓**	**✓**			**✓**			**✓**	**✓**	**✓**			**✓**
	Topical NSAID		**✓**			**✓**	**✓**		**✓**	**✓**	**✓**		**✓**		**✓**	**✓**	**✓**	**✓**			**✓**
	Oral NSAID		**✓**		**✓**	**✓**	**✓**		**✓**	**✓**	**✓**	**✓**	**✓**		**✓**	**✓**	**✓**				**✓**
	Oral NSAID treatment with caution for comorbidities (GI/CV/renal)			**✓**					**✓**	**✓**					**✓**	**✓**	**✓**	**✓**			**✓**
	Oral NSAID treatment with concomitant PPI/misoprostol in case of Gl risk factors			**✓**					**✓**		**✓**				**✓**	**✓**	**✓**	**✓**			
	Weak opioids		**✓**	**✓**	**✓**	**✓**	**✓**		**✓**	**✓**	**✓**	**✓**	**✓**			**✓**	**✓**	**✓**			**✓**
	Strong opioids					**✓**				**✓**			**✓**			**✓**					**✓**
	Corticosteroid injection	**✓**	**✓**			**✓**	**✓**		**✓**	**✓**	**✓**		**✓**		**✓**	**✓**		**✓**			**✓**
	Duloxetine					**✓**	**✓**			**✓**	**✓**		**✓**		**✓**		**✓**				**✓**
TJA preconditions	Referral to orthopaedic surgeon if conservative therapy failed	**✓**			**✓**				**✓**		**✓**		**✓**			**✓**		**✓**			
	Combination of pharmacological and non‐pharma‐cological therapies (before TJA)	**✓**			**✓**				**✓**							**✓**	**✓**				

Intervention category	Ambulatory health intervention quantifiable in routine data	TLAR [71]	AAOS [1]	APTA [16]	EULAR [64]	Ottawa Panel [11]	Ottawa Panel [10]	Ottawa Panel [12]	PANLAR [62]	EULAR [27]	NHG [9]	EULAR [80]	Ottawa Panel [13]	MOVE consensus [63]	EULAR [37]	Philadelphia Panel [60]
Musculo‐skeletal appointment	Physician visit for musculoskeletal disorders			**✓**	**✓**					**✓**	**✓**	**✓**			**✓**	
Diagnostic imaging	Radiographic assessment (prior to CT, MRI, sonography)				**✓**						**✓**	**✓**			**✓**	
Exercise therapy	Exercise therapy/referral to PT	**✓**	**✓**	**✓**		**✓**	**✓**	**✓**	**✓**	**✓**	**✓**	**✓**	**✓**	**✓**	**✓**	**✓**
Physical therapy	TENS	**✓**							**✓**		**✓**					**✓**
	NMES															**✓**
	Massage										**✓**					**✓**
	Acupuncture										**✓**					
	Orthoses/braces			**✓**					**✓**						**✓**	
	Walking aids			**✓**					**✓**	**✓**	**✓**	**✓**			**✓**	
	Shoes/insoles								**✓**	**✓**	**✓**	**✓**			**✓**	
Medication	Paracetamol (initial medication)	**✓**							**✓**		**✓**	**✓**			**✓**	
	Topical NSAID	**✓**							**✓**		**✓**				**✓**	
	Oral NSAID	**✓**	**✓**						**✓**		**✓**	**✓**			**✓**	
	Oral NSAID treatment with caution for comorbidities (GI/CV/renal)	**✓**							**✓**		**✓**					
	Oral NSAID treatment with concomitant PPI/misoprostol in case of Gl risk factors								**✓**							
	Weak opioids	**✓**							**✓**		**✓**	**✓**			**✓**	
	Strong opioids															
	Corticosteroid injection	**✓**	**✓**						**✓**		**✓**	**✓**			**✓**	
	Duloxetine	**✓**							**✓**							
TJA preconditions	Referral to orthopaedic surgeon if conservative therapy failed	**✓**							**✓**		**✓**	**✓**			**✓**	
	Combination of pharmacological and non‐pharma‐cological therapies (before TJA)	**✓**										**✓**			**✓**	

Legends: **✓** = ambulatory health intervention is addressed within the included guideline recommendations; AAOS = American Academy of Orthopaedic Surgeons; ACR = American College of Rheumatology; APTA = American Physical Therapy Association; AWMF = Association of the Scientific Medical Societies in Germany; CT = computer tomography; CV = cardiovascular; DVA = Department of Veterans Affairs; ESCEO = European Society for Clinical and Economic Aspects of Osteoporosis, Osteoarthritis and Musculoskeletal Diseases; EULAR = European League Against Rheumatism; GI = gastrointestinal; KNGF = The Royal Dutch Society for Physiotherapy; MQIC = Michigan Quality Improvement Consortium Guideline; MRI = magnetic resonance imaging; NHG = Dutch College of General Practitioners; NHMRC = National Health and Medical Research Council; NICE = National Institute for Health and Care Excellence; NMES = neuromuscular electrical stimulation; NSAID = non‐steroidal anti‐inflammatory drugs; OARSI = Osteoarthritis Research Society International; PANLAR = Pan‐American League of Associations for Rheumatology; PPI = proton pump inhibitors; PT = physical therapist; SFR = French Society of Rheumatology; SIR = Italian Society for Rheumatology; TENS = transcutaneous electrical nerve stimulation; TJA = total joint arthroplasty.

### Synthesis of routine‐data‐compatible QIs

Overall, 24 QIs that are compatible with routine data were synthesised (Table 5). All of these focused on the process dimension of care quality from a healthcare provider's perspective. Fourteen of the QIs were derived solely from guideline recommendations. Depending on their evidence base, some of the QIs are only for use for patients with osteoarthritis of the knee, some only for patients with osteoarthritis of the hip and some are for both.

**Table 5:** Routine‐data‐compatible QIs for ambulatory care of osteoarthritis of the knee and hip.

Intervention category	QI	Literature
Musculoskeletal appointment	1. If a patient has a diagnosis of osteoarthritis of the knee/hip for >12 months, then there should be documentation of at least one appointment with a qualified physician annually.	[39, 46, 69]
Diagnostic imaging	2. If a patient is initially diagnosed with osteoarthritis of the knee/hip, there must be documentation that the initial diagnosis of osteoarthritis of the knee/hip was based on radiologic imaging.	[19, 20]
	3. If the initial diagnosis of osteoarthritis of the knee/hip has been radiologically examined, then conventional radiography should be used prior to other imaging modalities (MRI, CT, sonography).	[4, 9, 19, 20, 21, 64]
	4. If a patient has a diagnosis of osteoarthritis of the knee/hip for >24 months, then there should be documentation that the location of osteoarthritis of the knee/hip was documented at least once every 2 years.	[6, 47, 50]
	5. If a patient with osteoarthritis of the knee is newly prescribed a prescription medication and has no previous radiograph, then a radiograph of the affected joint should be performed.	[31]
	6. If a patient with osteoarthritis of the knee/hip has undergone TJA, there should be documentation of radiographic imaging of the affected joint before TJA.	[4, 21, 37, 71, 80]
Exercise therapy	7. If a patient has a diagnosis of osteoarthritis of the knee/hip for >12 months, then there should be documentation of at least one referral to a physical therapist.	[24, 68, 82]
	8. If a patient has a diagnosis of osteoarthritis of the knee/hip for >3 months, there should be documentation of at least one referral to a physical therapist within 3 months after the initial diagnosis of osteoarthritis of the knee/hip.	[26, 46, 65, 68, 69]
Medication	9. If pharmacological therapy is initiated in a patient with osteoarthritis of the knee/hip, topical pharmacological treatments should be prescribed ahead of oral pharmacological treatments.	[15, 19, 52, 78]
	10. If oral pharmacological therapy is initiated in a patient with osteoarthritis of the knee/hip, paracetamol should be the first prescribed oral analgesic unless contraindicated.	[4, 6, 9, 24, 31, 37, 39, 46, 47, 50, 52, 56, 65, 68, 69, 72, 76, 80, 82]
	11. If a patient with osteoarthritis of the knee/hip is treated with paracetamol, then the prescribed maximum dose should not exceed 3 g/day.	[4, 15, 31, 49, 62, 80]
	12. If a patient with osteoarthritis of the knee/hip is newly prescribed oral NSAIDs, then ibuprofen should be the first prescribed oral NSAID unless contraindicated.	[72]
	13. If a patient with osteoarthritis of the knee/hip has an additional diagnosis of CV risk factors (diabetes mellitus, hypertension, hyperlipidaemia) and is prescribed oral NSAIDs, then naproxen should be the first prescribed oral NSAID unless contraindicated.	[4, 19, 62]
	14. If a patient with osteoarthritis of the knee/hip has a risk factor for GI bleeding (aged ≥75 years, peptic ulcer disease, history of GI bleeding, warfarin use, chronic glucocorticoid use) and is prescribed (COX) nonselective NSAIDs, then misoprostol or a PPI should be prescribed concomitantly.	[3, 4, 7, 19, 24, 31, 46, 49, 52, 62, 72, 78, 81, 82]
	15. If a patient with osteoarthritis of the knee/hip does not have a diagnosis of relevant comorbidities (CV, renal, GI) and is prescribed oral NSAIDs, then the prescription period should not exceed 3 months.	[81]
	16. If a patient with osteoarthritis of the knee/hip is unresponsive/contraindicated to paracetamol and/or oral NSAIDs, then weak opioids^a^ should be prescribed.	[4, 9, 15, 20, 37, 52, 62, 71, 78, 79, 80, 81]
	17. If a patient with osteoarthritis of the knee/hip is prescribed duloxetine, there should be documentation that paracetamol and/or oral NSAIDs and/or weak opioids^a^ have been prescribed before the prescription of duloxetine unless contraindicated.	[7, 15, 18, 40, 51, 62, 67, 71, 78, 81]
	18. If a patient with osteoarthritis of the knee/hip is treated with IA corticosteroids, there should be documentation that paracetamol and/or oral NSAIDs and/or weak opioids^a^ have been prescribed before the application of IA corticosteroids unless contraindicated.	[4, 9, 18, 62, 71, 79, 80]
	19. If a patient with osteoarthritis of the knee/hip is treated with IA corticosteroids more than once, then the injection interval should not be shorter than 4 months.	[71, 81]
	20. If a patient with osteoarthritis of the knee is contraindicated to TJA and has documentation of being prescribed weak opioids^a^, then strong opioids should be prescribed unless contraindicated.	[15, 67]
TJA preconditions	21. If a patient with osteoarthritis of the knee/hip has undergone TJA, there should be documentation of a combination of nonpharmacological and pharmacological treatment modalities before TJA.	[21, 37, 52, 71, 78, 79, 80]
	22. If a patient with osteoarthritis of the hip has undergone TJA, there should be documentation of a combination of nonpharmacological and pharmacological treatment modalities for at least 3 months before TJA.	[21]
	23. If a patient with osteoarthritis of the hip is treated with IA corticosteroids, then TJA should be realised 3 months after the last IA injection date.	[21]
	24. If a patient with osteoarthritis of the knee/hip is referred to an orthopaedic surgeon, then the waiting time from first referral to appointment should not exceed 3 months.	[26]

Abbreviations: CT, computer tomography; COX, cyclooxygenase; CV, cardiovascular; GI, gastrointestinal; IA, intra‐articular; MRI, magnetic resonance imaging; NSAID, nonsteroidal anti‐inflammatory drugs; PPI, proton pump inhibitors; QI, quality indicator; TJA, total joint arthroplasty; WHO, World Health Organization.

^a^ The distinction between weak and strong opioids is made according to the three‐level scale of the WHO for the management of pain [20].

In summary, it was found that routine data can be used to quantify QIs or guideline recommendations related to musculoskeletal appointments (e.g., for the clinical assessment of pain, functionality or the need for a TJA). This quantification is possible through the use of routine data on the number and timing of patient visits with qualified physicians. Moreover, such data allow for a transparent analysis of how frequently diagnostic imaging procedures are employed. The crucial role of exercise therapy in the management of osteoarthritis of the knee and hip is addressed by being able to use routine data to measure the initiation of such therapy under professional supervision (i.e., based on the presence of a referral to a physical therapist). Furthermore, it is possible to trace the sequence and volume of prescriptions, the use of pharmaceuticals and the related expenditures before TJA using routine data. In contrast, QIs related to physical therapy options were scarce. The guideline recommendations for physical therapy were sometimes contradictory and often specified that physical therapy should be tailored to a patient's needs and preferences. As a result, the prescription of physical therapy options could not be included in the set as a QI for the ambulatory care of patients with osteoarthritis of the knee and hip.

## DISCUSSION

The main finding of this systematic review is that, despite the requirement of routine data compatibility, the synthesised QI set can quantify a broad range of the processes employed in the ambulatory care of osteoarthritis of the knee and hip. The evidence synthesis resulted in a comprehensive set of routine‐data‐compatible QIs that can guide physicians in evaluating their practice, identifying areas for improvement and formulating potential courses of action. Routine‐data‐compatible QIs are advantageous because they do not require primary data collection, generally depict all reimbursed health resource utilisation and facilitate comparative analysis among physicians treating patients covered by SHIs [77]. Given the often protracted nature of the treatment of osteoarthritis of the knee and hip, which involves multiple medical specialties, sharing and discussing QI outcomes can foster interdisciplinary physician collaboration, and thereby improve the coordination and continuity of ambulatory care of osteoarthritis of the knee and hip.

The available evidence largely concurs with most of the recommended health interventions for ambulatory care of osteoarthritis of the knee and hip before TJA. Despite this consensus, the identified QIs and guideline recommendations vary in specific details, such as the frequency of treatments or the duration of certain health interventions. These discrepancies might be due to different healthcare contexts [5], the specific osteoarthritis populations targeted [59] or the degree of osteoarthritis severity. Additionally, the considerable variation in the grading systems of both LOE and SOR [53] requires an individual assessment of each guideline. To ensure that the QI set is applied appropriately in different healthcare settings, it is important to critically review the evidence underpinning each QI, particularly regarding the comparability of relevant contextual factors.

A recurring theme in the literature is the combination of health interventions, including exercise therapy, pharmaceutical treatment and patient education within evidence‐based ambulatory care of osteoarthritis of the knee and hip [25, 43, 53]. The synthesised set of QIs can quantify the provision of exercise therapy and pharmaceutical treatment. However, disease‐specific patient education, which is widely seen as crucial for developing an individual self‐management plan, is not captured by routine‐data‐compatible QIs, at least in the German healthcare system. As of the time of writing, the fee schedule used by German SHIs lacks an explicit item for standardised physician–patient communication.

A number of health interventions recommended in the international literature are not part of the catalogue of services covered by German SHIs due to a lack of clear evidence. Nevertheless, some of these interventions, such as IA hyaluronic acid treatments, can be purchased out of pocket by patients (so‐called ‘IGeL’ services in Germany). However, because these services are not included in routine data collection, their utilisation cannot be monitored.

Additionally, routine‐data‐compatible QIs do not consider essential aspects of health service provision, such as patients' individual experiences, preferences and socioeconomic context. These factors are crucial for patient‐specific disease management, especially in chronic diseases [44]. Moreover, routine data do not include information on clinical aspects such as the severity of osteoarthritis or individual levels of pain and suffering. Such clinical considerations are more critical when deciding on TJA than the quantity of previous ambulatory health interventions. Despite these limitations, the potential of routine data for conclusive quality measurement in ambulatory care of osteoarthritis of the knee and hip was examined due to their ready availability and generally high reliability.

Concerning the three quality dimensions of care that can be represented as QIs [23], it is evident that the synthesised QI set focuses solely on the process dimension. This emphasis is largely attributable to the use of routine data as the predefined basis for quantification. The items on the fee schedule for German SHIs may only allow limited conclusions to be drawn about outcome parameters of ambulatory care of osteoarthritis of the knee and hip, such as pain, disability and joint stiffness, which could be evaluated using the Western Ontario and McMaster Universities Arthritis Index [29].

Process QIs can be directly influenced by healthcare providers and usually do not require risk adjustment [22]. Thus, the synthesised QI set can serve as a practical tool for developing individual action plans. These process QIs enable straightforward comparability, potentially fostering discussion and exchange among physicians.

Given the interrelation of the different dimensions of care quality [23, 34], adhering to process QIs could contribute to improved therapy outcomes. Further research is, therefore, warranted to explore how insights about therapy outcomes can be gained from routine‐data‐compatible process QIs. Such investigations could deepen the understanding of the critical issues that need to be addressed to improve the overall quality of care.

## CONCLUSION

The synthesised QI set can provide physicians with information for the evaluation of their work, thus serving as a basis for the identification of potential for improvements in the provision of ambulatory care for osteoarthritis of the knee and hip. Interpreting QI results always requires caution and a balanced approach, taking the specific healthcare context into account. Failing to meet a QI does not automatically imply misconduct by a healthcare provider, as there could be valid medical reasons in individual cases. To maximise the impact of this synthesised set of QIs on quality of care, exploring its dissemination among quality circles and interdisciplinary physician networks could be beneficial.

## AUTHOR CONTRIBUTIONS

Tobias Bock was the leading author of the article. Leonie Sundmacher and Ronja Flemming were involved in acquisition of funding. Leonie Sundmacher and Ronja Flemming planned and designed the study and developed the intervention. Tobias Bock and Ronja Flemming developed the search strategy of this review and dually conducted the first screening phase (title/abstract). Tobias Bock conducted the second screening phase (full‐text). Tobias Bock conducted extraction, analysis and synthesis of evidence. Tobias Bock drafted the manuscript in its published form together with Ronja Flemming, Philip Bammert and Leonie Sundmacher. All authors provided input, reviewed and revised the original manuscript. All authors approved the submitted version of the manuscript.

## CONFLICT OF INTEREST STATEMENT

The authors declare no conflicts of interest.

## ETHICS STATEMENT

This study was approved by the Ethics Committee of the Faculty of Medicine of the Technical University of Munich (566/21 S‐NP), granted on 13 October 2021. All changes in the protocol throughout the trial must be approved by the ethics committee and the responsible supervisory authorities.

## Supporting information

Supporting information.

Supporting information.

Supporting information.

Supporting information.
