# Supplementary material for: Routine‐data‐compatible quality indicators for the ambulatory care of osteoarthritis of the knee and hip: A systematic review
Source: Knee Surg Sports Traumatol Arthrosc. 2025 Feb 13;33(10):3523–41. doi: 10.1002/ksa.12614 (PMC12459328; doi:10.1002/ksa.12614)
Supplement: Supplementary file 1 — Supporting information. [file KSA-33-3523-s004.docx]

**APPENDIX 1.**

Search strategy.

[Key search terms for database searches. 2](#_Toc158989222)

[Pubmed. 3](#_Toc158989223)

[Cochrane Library. 5](#_Toc158989224)

[Web of Science. 8](#_Toc158989225)

[Scopus. 10](#_Toc158989226)

[PEDro. 12](#_Toc158989227)

[List of organisations and institutions. 13](#_Toc158989228)

## **Key search terms for database searches.**

|  | **Table 1.** Key search terms for database searches. | |
| --- | --- | --- |
|  | thematic block | search terms |
| indication | disease | arthritis, osteoarthritis, arthrosis, coxarthrosis, gonarthrosis, degenerative joint disease,  joint space narrowing, osteophyte |
|  | body part | hip, knee |
| ambulatory  care | setting | ambulatory, outpatient, primary health care, conservative, non-surgical, non-operative,  first-line, pre-operative, pre-surgery, referral, rehabilitation, continuity of care, coordination of care, multidisciplinary/interdisciplinary care |
|  | treatment option | education, exercise, physical therapy, physiotherapy, kinesiotherapy, ergotherapy, medication, drug therapy, pharmacological, non-pharmacological, natural/alternative medicine, occupational medicine |
| quality assessment | evidence-based medicine | evidence-based medicine, critical/clinical pathway, disease management |
|  | guideline | (clinical practice) guideline, recommendation |
|  | quality indicator | quality indicator, quality assurance/assessment, performance measure/metric/benchmark, standard of care, routine/claims/administrative/secondary data |

## **Pubmed.**

( ( osteoarthr*[ti] OR “osteoarthritis, knee”[Mesh] OR ( ( knee[tiab] OR knees[tiab] OR Knie[tiab] OR Kniegelenk* ) AND ( arthritis [ti] OR arthrosis [ti] OR arthrose[ti] OR osteoarthr*[ti] OR degenerat*[tiab] ) ) OR gonarthr*[tiab] OR Kniearthrose[tiab] OR Kniegelenkarthrose[tiab] OR Kniegelenksarthrose[tiab] OR “osteoarthritis, hip”[Mesh] OR ( ( hip[tiab] OR hips[tiab] OR Hüfte[tiab] OR Hüftgelenk*[tiab] ) AND ( arthritis [ti] OR arthrosis [ti] OR arthrose[ti] OR osteoarthr*[ti] OR degenerat*[tiab] ) ) OR coxarthr*[tiab] OR Hüftarthrose[tiab] OR Hüftgelenkarthrose[tiab] OR Hüftgelenksarthrose[tiab] OR ( ( Knie[tiab] OR Hüfte[tiab] OR Gelenk*[tiab] ) AND ( Gelenkspaltverengung*[tiab] OR Gelenkspaltverschmälerung*[tiab] OR Gelenkverschlei*[tiab] OR Verengung*[tiab] OR Verschmälerung*[tiab] OR Verschlei*[tiab] ) ) OR “joint space narrowing”[tiab] OR ( joint*[tiab] AND space[tiab] AND narrow*[tiab] AND ( knee[tiab] OR knees[tiab] OR hip[tiab] OR hips[tiab] ) ) OR “degenerative joint disease*”[tiab] OR ( degenerat*[tiab] AND joint*[tiab] AND disease*[tiab] AND ( knee[tiab] OR knees[tiab] OR hip[tiab] OR hips[tiab] ) ) OR “degenerative Gelenkerkankung*”[tiab] OR ( Gelenk* AND degenerative* ) OR “osteophyte”[Mesh] OR osteophyt*[tiab] ) AND ( ( ( ambulatory[tiab] OR outpatient[tiab] OR conservative[tiab] OR “non-surg*”[tiab] OR nonsurg*[tiab] OR “non-operat*”[tiab] OR nonoperat*[tiab] OR “first line”[tiab] OR referral*[tiab] OR rehabilit*[tiab] OR preoperat*[tiab] OR “pre-operat*”[tiab] OR presurg*[tiab] OR “pre-surg*”[tiab] OR ambulant*[tiab] OR niedergelassen*[tiab] OR konservativ*[tiab] OR “nicht chirurgisch*”[tiab] OR “nicht operativ*”[tiab] OR primär*[tiab] OR überweis*[tiab] OR kontinui*[tiab] OR Behandlungskontinuität[tiab] OR präoperat*[tiab] OR “primary health care”[Mesh] OR ( ( primary[tiab] OR continu*[tiab] ) AND (care[tiab] OR healthcare[tiab] ) ) ) AND ( exercise*[tiab] OR physical[tiab] OR medication*[tiab] OR pharmacolog*[tiab] OR nonpharmacolog*[tiab] OR pharmaceutic*[tiab] OR nonpharmaceutic*[tiab] OR pharmacotherap*[tiab] OR physiotherap*[tiab] OR kinesiotherap*[tiab] OR educat*[tiab] OR instruction*[tiab] OR advice*[tiab] OR ergotherap*[tiab] OR homeopath*[tiab] OR multidisciplin*[tiab] OR interdisciplin*[tiab] OR Bewegung*[tiab] OR Übung*[tiab] OR Betätigung*[tiab] OR Kräftigung*[tiab] OR physikalisch*[tiab] OR pharmazeut*[tiab] OR pharmako*[tiab] OR Aufklärung*[tiab] OR Bildung*[tiab] OR Anweisung*[tiab] OR Anleitung*[tiab] OR Beratung*[tiab] OR Empfehlung*[tiab] OR Hinweis*[tiab] OR multidisziplin*[tiab] OR interdisziplin*[tiab] OR Naturheilkunde[tiab] OR komplementärmedizin*[tiab] OR alternativmedizin*[tiab] OR Homöopath*[tiab] OR "occupational therapy"[Mesh] OR ( occupational[tiab] AND therap*[tiab] ) OR "naturopathy"[Mesh] OR naturopath*[tiab] OR ( natur*[tiab] AND ( healing[tiab] OR medicine[tiab] ) ) OR “exercise”[Mesh] OR “physical therapy modalities”[Mesh] OR “physical therapy modalit*”[tiab] OR ( physical[tiab] AND therap*[tiab] ) OR “drug therapy”[Mesh] OR ( drug*[tiab] AND therap*[tiab] ) ) AND ( ( “evidence-based”[tiab] OR evidencebased[tiab] OR “based on evidence”[tiab] OR guideline*[ti] OR recommendation*[tiab] OR evidenzbasiert*[tiab] OR Leitlinie*[tiab] OR Praxisleitlinie*[tiab] OR Richtlinie*[tiab] OR Empfehlung*[tiab] OR (klinisch*[tiab] AND (Evidenz[tiab] OR Behandlungspfad*[tiab] OR Audit[tiab] OR Audits[tiab] ) ) OR “evidence-based practice”[Mesh] OR “evidence-based medicine”[Mesh] OR ( evidence[tiab] AND based[tiab] ) OR “practice guidelines as topic”[Mesh] OR “clinical medicine”[Mesh] OR (clinical[tiab] AND ( medicine[tiab] OR evidence[tiab] OR path[tiab] OR paths[tiab] OR pathway*[tiab] OR audit*[tiab] OR audits[tiab] ) ) OR “critical pathways”[Mesh] OR “critical path*”[tiab] OR ( critical[tiab] AND ( path[tiab] OR paths[tiab] OR pathway*[tiab] ) ) OR “disease management”[Mesh] OR ( disease*[tiab] AND management[tiab]) OR “practice guideline”[Publication Type] ) OR ( “cohort stud*”[tiab] OR “case control stud*”[tiab] OR “clinical trial*”[tiab] OR RCT[tiab] OR RCTs[tiab] OR “randomized controlled trial*”[tiab] OR “randomised controlled trial*”[tiab] OR "cross-sectional stud*"[tiab] OR "cross-over stud*"[tiab] OR "longitudinal stud*"[tiab] OR Kohortenstudie*[tiab] OR “Fall-Kontroll-Studie*”[tiab] OR Fallkontrollstudie*[tiab] OR “klinische Studie*”[tiab] OR ( randomisiert*[tiab] AND kontrolliert*[tiab] AND studie*[tiab] ) OR Querschnittstudie*[tiab] OR Längsschnittstudie*[tiab] OR "cohort studies"[Mesh] OR "case-control studies"[Mesh] OR "clinical trials as topic"[Mesh] OR "controlled clinical trials as topic"[Mesh] OR "randomized controlled trials as topic"[ Mesh] OR "cross-sectional studies"[Mesh] OR "cross-over studies"[Mesh] OR "longitudinal studies"[ Mesh] OR "clinical trial"[Publication Type] OR "controlled clinical trial"[Publication Type] OR "randomized controlled trial"[Publication Type] ) ) ) OR ( ( indicator*[ti] OR qualit*[ti] OR performance*[tiab] OR benchmark*[tiab] OR ( standard*[tiab] AND ( care[tiab] OR healthcare[tiab] ) ) OR Indikator*[tiab] OR Qualitätsindikator*[tiab] OR Performanz[tiab] OR Leistung*[tiab] OR Maßstab[tiab] OR Maßstäbe[tiab] OR Richtwert*[tiab] OR Richtgröße*[tiab] OR Bewertung*[tiab] OR Beurteilung*[tiab] OR Katalog*[tiab] OR ( Standard*[tiab] AND ( Versorgung[tiab] OR Gesundheits*[tiab] ) ) OR “quality indicators, health care”[Mesh] OR “standard of care”[Mesh] OR “quality assurance, health care”[Mesh] OR ( quality[tiab] AND assurance[tiab] ) ) AND ( panel*[tiab] OR expert*[tiab] OR consens*[tiab] OR “steering group*”[tiab] OR delphi[tiab] OR rand[tiab] OR “meta-analys*”[tiab] OR metaanalys*[tiab] OR “systematic review*”[tiab] OR “literature review*”[tiab] OR Gremi*[tiab] OR Lenkungs*[tiab] OR Steuerungs*[tiab] OR Leitungs*[tiab] OR Ausschuss[tiab] OR Konsens*[tiab] OR Routinedaten[tiab] OR Sekundärdaten[tiab] OR Abrechnungsdaten[tiab] OR ( systematisch*[tiab] AND ( Review*[tiab] OR Literaturreview*[tiab] OR Übersichtsarbeit*[tiab] ) ) OR "meta-analysis as topic"[Mesh] OR "systematic reviews as topic"[Mesh] OR "meta-analysis"[Publication Type] OR "systematic review"[Publication Type] OR “routinely collected health data”[Mesh] OR “routine data”[tiab] OR “administrative data”[tiab] OR “secondary data”[tiab] OR claims[tiab] ) ) ) ) AND **( English[lang] OR German[lang] ) AND ( “2000/01/01”[PDAT] : “2021/06/15”[PDAT] ) AND ( “humans”[Mesh] ) NOT ( postoperat*[ti] OR “post-operat*”[ti] OR “post-op”[ti] OR postsurg*[ti] OR “post-surg*”[ti]** OR animal[tiab] OR animals[tiab] OR canine*[tiab] OR dog[tiab] OR dogs[tiab] OR feline[tiab] OR hamster*[tiab] OR lamb[tiab] OR lambs[tiab] OR mice[tiab] OR monkey[tiab] OR monkeys[tiab] OR mouse[tiab] OR murine[tiab] OR pig[tiab] OR pigs[tiab] OR piglet*[tiab] OR porcine[tiab] OR primate*[tiab] OR rabbit*[tiab] OR rats[tiab] OR rat[tiab] OR rodent*[tiab] OR sheep*[tiab] **)**

## Cochrane Library.

#1 (((knee OR knees) AND degenerat*) OR gonarthr* OR Kniearthrose OR Kniegelenkarthrose OR Kniegelenksarthrose OR ((hip OR hips) AND degenerat*) OR coxarthr* OR Hüftarthrose OR Hüftgelenkarthrose OR Hüftgelenksarthrose OR ((Knie OR Hüfte OR Gelenk*) AND (Gelenkspaltverengung* OR Gelenkspaltverschmälerung* OR Gelenkverschlei* OR Verengung* OR Verschmälerung* OR Verschlei*)) OR “joint space narrowing” OR (joint* AND space AND narrow* AND (knee OR knees OR hip OR hips)) OR “degenerative joint disease*” OR (degenerat* AND joint* AND disease* AND (knee OR knees OR hip OR hips)) OR osteophyt*):ti,ab,kw (Word variations have been searched)

#2 (osteoarthr*):ti (Word variations have been searched)

#3 (knee OR knees OR Knie OR Kniegelenk*):ti,ab,kw (Word variations have been searched)

#4 (arthritis OR arthrosis OR arthrose OR osteoarthr*):ti (Word variations have been searched)

#5 #3 AND #4 (Word variations have been searched)

#6 (hip OR hips OR Hüfte OR Hüftgelenk*):ti,ab,kw (Word variations have been searched)

#7 (arthritis OR arthrosis OR arthrose OR osteoarthr*):ti (Word variations have been searched)

#8 #6 AND #7 (Word variations have been searched)

#9 #1 OR #2 OR #5 OR #8 (Word variations have been searched)

#10 (ambulatory OR outpatient OR conservative OR “non-surg*” OR nonsurg* OR “non-operat*” OR nonoperat* OR “first line” OR referral* OR rehabilit* OR preoperat* OR "pre-operat*" OR presurg* OR "pre-surg*" OR ambulant* OR niedergelassen* OR konservativ* OR “nicht chirurgisch*” OR “nicht operativ*” OR primär* OR überweis* OR kontinui* OR Behandlungskontinuität OR präoperat* OR ((primary OR continu*) AND (care OR healthcare))):ti,ab,kw (Word variations have been searched)

#11 (exercise* OR physical OR medication* OR pharmacolog* OR nonpharmacolog* OR pharmaceutic* OR nonpharmaceutic* OR pharmacotherap* OR physiotherap* OR kinesiotherap* OR educat* OR instruction* OR advice* OR ergotherap* OR homeopath* OR Bewegung* OR Übung* OR Betätigung* OR Kräftigung* OR physikalisch* OR pharmazeut* OR pharmako* OR Aufklärung* OR Bildung* OR Anweisung* OR Anleitung* OR Beratung* OR Empfehlung* OR Hinweis* OR multidisciplin* OR interdisciplin* OR multidisziplin* OR interdisziplin* OR Naturheilkunde OR komplementärmedizin* OR alternativmedizin* OR Homöopath* OR (occupational AND therap*) OR naturopath* OR (natur* AND (healing OR medicine)) OR “physical therapy modalit*” OR (physical AND therap*) OR (drug* AND therap*)):ti,ab,kw (Word variations have been searched)

#12 (“evidence-based” OR evidencebased OR “based on evidence” OR recommendation* OR evidenzbasiert* OR Leitlinie* OR Praxisleitlinie* OR Richtlinie* OR Empfehlung* OR (klinisch* AND (Evidenz OR Behandlungspfad* OR Audit OR Audits)) OR (evidence AND based) OR (clinical AND (medicine OR evidence OR path OR paths OR pathway* OR audit OR audits)) OR “critical path*” OR (critical AND (path OR paths OR pathway*)) OR (disease* AND management)):ti,ab,kw (Word variations have been searched)

#13 (guideline*):ti (Word variations have been searched)

#14 #12 OR #13 (Word variations have been searched)

#15 (performance* OR metric* OR assessment* OR benchmark* OR (standard* AND (care OR healthcare)) OR set OR sets OR list* OR Indikator* OR Qualitätsindikator* OR Performanz OR Leistung* OR Maßstab OR Maßstäbe OR Richtwert* OR Richtgröße* OR Bewertung* OR Beurteilung* OR Katalog* OR (Standard* AND (Versorgung OR Gesundheits*)) OR (quality AND assurance)):ti,ab,kw (Word variations have been searched)

#16 (indicator* OR qualit*):ti (Word variations have been searched)

#17 #15 OR #16 (Word variations have been searched)

#18 (“cohort stud*” OR “case control stud*” OR “clinical trial*” OR RCT OR RCTs OR “randomized controlled trial*” OR “randomised controlled trial*” OR "cross-sectional stud*" OR "cross-over stud*" OR "longitudinal stud*" OR Kohortenstudie* OR “Fall-Kontroll-Studie*” OR Fallkontrollstudie* OR “klinische Studie*” OR (randomisiert* AND kontrolliert* AND studie*) OR Querschnittstudie* OR Längsschnittstudie*):ti,ab,kw (Word variations have been searched)

#19 (valid* OR panel* OR expert* OR consens* OR “steering group*” OR delphi OR rand OR "meta-analys*" OR metaanalys* OR “systematic review*” OR (literature AND review*) OR Gremi* OR Lenkungs* OR Steuerungs* OR Leitungs* OR Ausschuss OR Konsens* OR Routinedaten OR Sekundärdaten OR Abrechnungsdaten OR (systematisch* AND (Review* OR Literaturreviw* OR Übersichtsarbeit*)) OR "routine data" OR "administrative data" OR "secondary data" OR claims):ti,ab,kw (Word variations have been searched)

#20 MeSH descriptor: [Osteoarthritis, Knee] explode all trees

#21 MeSH descriptor: [Osteoarthritis, Hip] explode all trees

#22 MeSH descriptor: [Osteophyte] explode all trees

#23 MeSH descriptor: [Primary Health Care] explode all trees

#24 MeSH descriptor: [Exercise] explode all trees

#25 MeSH descriptor: [Physical Therapy Modalities] explode all trees

#26 MeSH descriptor: [Drug Therapy] explode all trees

#27 MeSH descriptor: [Occupational Therapy] explode all trees

#28 MeSH descriptor: [Naturopathy] explode all trees

#29 MeSH descriptor: [Evidence-Based Practice] explode all trees

#30 MeSH descriptor: [Evidence-Based Medicine] explode all trees

#31 MeSH descriptor: [Practice Guidelines as Topic] explode all trees

#32 MeSH descriptor: [Clinical Medicine] explode all trees

#33 MeSH descriptor: [Critical Pathways] explode all trees

#34 MeSH descriptor: [Disease Management] explode all trees

#35 MeSH descriptor: [Practice Guideline] explode all trees

#36 MeSH descriptor: [Quality Indicators, Health Care] explode all trees

#37 MeSH descriptor: [Standard of Care] explode all trees

#38 MeSH descriptor: [Quality Assurance, Health Care] explode all trees

#39 MeSH descriptor: [Cohort Studies] explode all trees

#40 MeSH descriptor: [Case-Control Studies] explode all trees

#41 MeSH descriptor: [Clinical Trials as Topic] explode all trees

#42 MeSH descriptor: [Controlled Clinical Trials as Topic] explode all trees

#43 MeSH descriptor: [Randomized Controlled Trials as Topic] explode all trees

#44 MeSH descriptor: [Cross-Sectional Studies] explode all trees

#45 MeSH descriptor: [Cross-Over Studies] explode all trees

#46 MeSH descriptor: [Longitudinal Studies] explode all trees

#47 MeSH descriptor: [Meta-Analysis as Topic] explode all trees

#48 MeSH descriptor: [Systematic Reviews as Topic] explode all trees

#49 MeSH descriptor: [Routinely Collected Health Data] explode all trees

#50 ("post-op" OR "post-surg*" OR postsurg* OR postoperat* OR "post-operat*" OR animal OR animals OR canine* OR dog OR dogs OR feline OR hamster* OR lamb OR lambs OR mice OR monkey OR monkeys OR mouse OR murine* OR pig OR pigs OR piglet* OR porcine OR primate* OR rabbit* OR rats OR rat OR rodent* OR sheep*):ti

#51 #9 OR #20 OR #21 OR #22 (Word variations have been searched)

#52 #10 OR #23 (Word variations have been searched)

#53 #11 OR #24 OR #25 OR #26 OR #27 OR #28 (Word variations have been searched)

#54 #14 OR #29 OR #30 OR #31 OR #32 OR #33 OR #34 OR #35 (Word variations have been searched)

#55 #17 OR #36 OR #37 OR #38 (Word variations have been searched)

#56 #18 OR #39 #40 OR #41 OR #42 OR #43 OR #44 OR #45 OR #46 (Word variations have been searched)

#57 #19 OR #47 OR #48 OR #49 (Word variations have been searched)

#58 #54 OR #56 (Word variations have been searched)

#59 #51 AND #52 AND #53 AND #58 (Word variations have been searched)

#60 #51 AND #55 AND #57 (Word variations have been searched)

#61 #59 OR #60 (Word variations have been searched)

#62 #61 NOT #50 with Cochrane Library publication date Between Jan 2000 and Jun 2021 (Word variations have been searched)

## Web of Science.

**#1**:

( TS= ( ( ( knee OR knees) AND degenerat* ) OR gonarthr* OR Kniearthrose OR Kniegelenkarthrose OR Kniegelenksarthrose OR ( ( hip OR hips ) AND degenerat* ) OR coxarthr* OR Hüftarthrose OR Hüftgelenkarthrose OR Hüftgelenksarthrose OR ( (Knie OR Hüfte OR Gelenk*) AND

( Gelenkspaltverengung* OR Gelenkspaltverschmälerung* OR Gelenkverschlei* OR Verengung* OR Verschmälerung* OR Verschlei* ) ) OR "joint space narrowing" OR ( joint* AND space AND narrow* AND ( knee OR knees OR hip OR hips ) ) OR "degenerative joint disease*" OR ( degenerat* AND joint* AND disease* AND ( knee OR knees OR hip OR hips ) ) OR “degenerative Gelenkerkrankung*” OR (Gelenk* AND degenerat* ) OR osteophyt* ) OR TI= ( osteoarthr* ) OR (TS= (knee* ) AND TI= ( arthritis OR arthrosis OR osteoarthr* ) ) OR (TS= (hip ) AND TI= ( arthritis OR arthrosis OR osteoarthr* ) ) )

**#2**:

( TS= ( ( ambulatory OR outpatient OR conservative OR "non-surg*" OR nonsurg* OR "non-operat*" OR nonoperat* OR "first line" OR referral* OR rehabilit* OR preoperat* OR “pre-operat*” OR presurg* OR “pre-surg*” OR ambulant* OR niedergelassen* OR konservativ* OR “nicht chirurgisch*” OR “nicht operativ*” OR primär* OR überweis* OR kontinui* OR Behandlungskontinuität OR präoperat* OR ( ( primary OR continu* ) AND ( care OR healthcare ) ) ) ) )

**#3**:

(TS= ( exercise* OR physical OR medication* OR pharmacolog* OR nonpharmacolog* OR pharmaceutic* OR nonpharmaceutic* OR pharmacotherapy* OR physiotherap* OR kinesiotherap* OR educat* OR instruction* OR advice* OR ergotherap* OR homeopath* OR multidisciplin* OR interdisciplin* OR Bewegung* OR Übung* OR Betätigung* OR Kräftigung* OR physikalisch* OR pharmazeut* OR pharmako* OR Aufklärung* OR Bildung* OR Anweisung* OR Anleitung* OR Beratung* OR Empfehlung* OR Hinweis* OR multidisziplin* OR interdisziplin* OR Naturheilkunde OR komplementärmedizin* OR alternativmedizin* OR Homöopath* OR ( occupational AND therap* ) OR naturopath* OR ( natur* AND ( healing OR medicine ) ) OR "physical therapy modalit*" OR ( physical AND therap* ) OR ( drug* AND therap* ) ) )

**#4**:

( TS= ( "evidence-based" OR evidencebased OR "based on evidence" OR recommendation* OR evidenzbasiert* OR Leitlinie* OR Praxisleitlinie* OR Richtlinie* OR Empfehlung* OR ( klinisch* AND ( Evidenz OR Behandlungspfad* OR Audit OR Audits ) ) OR ( evidence AND based ) OR ( clinical AND ( medicine OR evidence OR path OR paths OR pathway* OR audit OR audits ) ) OR "critical path*" OR ( critical AND ( path* OR paths OR pathway* ) ) OR ( disease* AND management ) ) OR TI= ( guideline* ) )

**#5**:

( TS= ( performance* OR metric* OR assessment* OR benchmark* OR ( standard* AND ( care OR healthcare ) ) OR set OR sets OR list* OR Indikator* OR Qualitätsindikator* OR Performanz OR Leistung* OR Maßstab OR Maßstäbe OR Richtwert* OR Richtgröße* OR Bewertung* OR Beurteilung* OR Katalog* OR (Standard* AND (Versorgung OR Gesundheits* ) ) OR ( quality AND assurance ) ) OR TI= ( indicator* ) OR TI= ( qualit* ) )

**#6**: ( TS= ( "cohort stud*" OR "case control stud*" OR "clinical trial*" OR rct OR rcts OR "randomized controlled trial*" OR "randomised controlled trial*" OR "cross-sectional stud*" OR "cross-over stud*" OR "longitudinal stud*" OR Kohortenstudie* OR “Fall-

Kontroll-Studie*” OR Fallkontrollstudie* OR “klinische Studie*” OR ( randomisiert* AND kontrolliert* AND Studie* ) OR Querschnittstudie* OR Längsschnittstudie* ) )

**#7**:

( TS= ( valid* OR panel* OR expert* OR consens* OR "steering group*" OR delphi OR rand OR "meta-analys*" OR "systematic review*" OR "literature review*" OR Gremi* OR Lenkungs* OR Steuerungs* OR Leitungs* OR Ausschuss OR Konsens* OR Routinedaten OR Sekundärdaten OR Abrechnungsdaten OR ( systematisch* AND ( Review* OR Literaturreview* OR Übersichtsarbeit* ) ) OR “routine data” OR “administrative data” OR “secondary data” OR claims ) )

**#8**:

( LA= ( ( English ) OR ( German ) ) ) NOT TI= (postoperat* OR “post-operat*” OR “post-op” OR postsurg* OR “post-surg*” OR animal OR animals OR canine* OR dog OR dogs OR feline OR hamster* OR lamb OR lambs OR mice OR monkey OR monkeys OR mouse OR murine OR pig OR pigs OR piglet* OR porcine OR primate* OR rabbit* OR rats OR rat OR rodent* OR sheep* )

**#9:**

#6 OR #4

**#10:**

#9 AND #3 AND #2 AND #1

**#11:**

#7 AND #5 AND #1

**#12:**

#11 OR #10

**#13:**

#12 AND #8

Indexes= SCI-EXPANDED, SSCI, CPCI-S, CPCI-SSH, BKCI-S, BKCI-SSH Timespan=2000-2021

## Scopus.

( ( ( ( TITLE-ABS-KEY ( ( ( ( knee OR knees ) AND degenerat* ) OR gonarthr* OR Kniearthrose OR Kniegelenkarthrose OR Kniegelenksarthrose OR ( ( hip OR hips ) AND degenerat* ) OR coxarthr* OR Hüftarthrose OR Hüftgelenkarthrose OR Hüftgelenksarthrose OR ( ( Knie OR Hüfte OR Gelenk* ) AND ( Gelenkspaltverengung* OR Gelenkspaltverschmälerung* OR Gelenkverschlei* OR Verengung* OR Verschmälerung* OR Verschlei* ) ) OR "joint space narrowing" OR ( joint* AND space AND narrow* AND ( knee OR knees OR hip OR hips ) ) OR "degenerative joint disease*" OR ( degenerat* AND joint* AND disease* AND ( knee OR knees OR hip OR hips ) ) OR “degenerative Gelenkerkrankung*” OR ( Gelenk* AND degenerat* ) OR osteophyt* ) ) ) OR ( TITLE ( ( osteoarthr* ) ) ) OR ( ( TITLE-ABS-KEY ( ( ( knee OR knees OR Knie OR Kniegelenk* ) ) ) ) AND ( TITLE ( ( arthritis OR arthrosis OR arthrose OR osteoarthr* ) ) ) ) OR ( ( TITLE-ABS-KEY ( ( ( hip OR hips OR Hüfte OR Hüftgelenk* ) ) ) ) AND ( TITLE ( ( arthritis OR arthrosis OR arthrose OR osteoarthr* ) ) ) ) ) AND ( TITLE-ABS-KEY ( ( ambulatory OR outpatient OR conservative OR "non-surg*" OR nonsurg* OR "non-operat*" OR nonoperat* OR "first line" OR referral* OR rehabilit* OR preoperat* OR “pre-operat*” OR presurg* OR “pre-surg*” OR ambulant* OR niedergelassen* OR konservativ* OR “nicht chirurgisch*” OR “nicht operativ*” OR primär* OR überweis* OR kontinui* OR Behandlungskontinuität OR präoperat* OR ( ( primary OR continu* ) AND ( care OR healthcare ) ) ) ) ) AND ( TITLE-ABS-KEY ( ( exercise* OR physical OR medication* OR pharmacolog* OR nonpharmacolog* OR pharmaceutic* OR nonpharmaceutic* OR pharmacotherap* OR physiotherap* OR kinesiotherap* OR educat* OR instruction* OR advice* OR ergotherap* OR homeopath* OR multidisciplin* OR interdisciplin* OR Bewegung* OR Übung* OR Betätigung* OR Kräftigung* OR physikalisch* OR pharmazeut* OR pharmako* OR Aufklärung* OR Bildung* OR Anweisung* OR Anleitung* OR Beratung* OR Empfehlung* OR Hinweis* OR multidisziplin* OR interdisziplin* OR Naturheilkunde OR komplementärmedizin* OR alternativmedizin* OR Homöopath* OR ( occupational AND therap* ) OR naturopath* OR ( natur* AND ( healing OR medicine ) ) OR "physical therapy modalit*" OR ( physical AND therap* ) OR ( drug* AND therap* ) ) ) ) AND ( ( ( TITLE-ABS-KEY ( ( "evidence-based" OR evidencebased OR "based on evidence" OR recommendation* OR evidenzbasiert* OR Leitlinie* OR Praxisleitlinie* OR Richtlinie* OR Empfehlung* OR ( klinisch* AND ( Evidenz OR Behandlungspfad* OR Audit OR Audits ) ) OR ( evidence AND based ) OR ( clinical AND ( medicine OR evidence OR path OR paths OR pathway* OR audit OR audits ) ) OR "critical path*" OR ( critical AND ( path OR paths OR pathway* ) ) OR ( disease* AND management ) ) ) ) OR ( TITLE ( ( guideline* ) ) ) ) OR ( ( TITLE-ABS-KEY ( ( "cohort stud*" OR "case control stud*" OR "clinical trial*" OR rct OR rcts OR "randomized controlled trial*" OR "randomised controlled trial*" OR "cross-sectional stud*" OR "cross-over stud*" OR "longitudinal stud*" OR Kohortenstudie* OR “Fall-Kontroll-Studie*” OR Fallkontrollstudie* OR “klinische Studie*” OR ( randomisiert* AND kontrolliert* AND Studie* ) OR Querschnittstudie* OR Längsschnittstudie* ) ) ) ) ) ) OR ( ( ( TITLE-ABS-KEY ( ( ( ( knee OR knees ) AND degenerat* ) OR gonarthr* OR Kniearthrose OR Kniegelenkarthrose OR Kniegelenksarthrose OR ( ( hip OR hips ) AND degenerat* ) OR coxarthr* OR Hüftarthrose OR Hüftgelenkarthrose OR Hüftgelenksarthrose OR ( ( Knie OR Hüfte OR Gelenk* ) AND ( Gelenkspaltverengung* OR Gelenkspaltverschmälerung* OR Gelenkverschlei* OR Verengung* OR Verschmälerung* OR Verschlei* ) ) OR "joint space narrowing" OR ( joint* AND space AND narrow* AND ( knee OR knees OR hip OR hips ) ) OR "degenerative joint disease*" OR ( degenerat* AND joint* AND disease* AND ( knee OR knees OR hip OR hips ) ) OR “degenerative Gelenkerkrankung*” OR ( Gelenk* AND degenerat* ) OR osteophyt* ) ) ) OR ( TITLE ( ( osteoarthr* ) ) ) OR ( ( TITLE-ABS-KEY ( ( ( knee OR knees OR Knie OR Kniegelenk* ) ) ) ) AND ( TITLE ( ( arthritis OR arthrosis OR arthrose OR osteoarthr* ) ) ) ) OR ( ( TITLE-ABS-KEY ( ( ( hip OR hips OR Hüfte OR Hüftgelenk* ) ) ) ) AND ( TITLE ( ( arthritis OR arthrosis OR arthrose OR osteoarthr* ) ) ) ) ) AND ( ( TITLE-ABS-KEY ( ( performance* OR metric* OR assessment* OR benchmark* OR ( standard* AND ( care OR healthcare ) ) OR set OR sets OR list* OR Indikator* OR Qualitätsindikator* OR Performanz OR Leistung* OR Maßstab OR Maßstäbe OR Richtwert* OR Richtgröße* OR Bewertung* OR Beurteilung* OR Katalog* OR ( Standard* AND ( Versorgung OR Gesundheits* ) ) OR ( quality AND assurance ) ) ) ) OR ( TITLE ( ( indicator* OR qualit* ) ) ) ) AND ( TITLE-ABS-KEY ( ( valid* OR panel* OR expert* OR consens* OR "steering group*" OR delphi OR rand OR "meta-analys*" OR "systematic review*" OR "literature review*" OR Gremi* OR Lenkungs* OR Steuerungs* OR Leitungs* OR Ausschuss OR Konsens* OR Routinedaten OR Sekundärdaten OR Abrechnungsdaten OR ( systematisch* AND ( Review* OR Literaturreview* OR Übersichtsarbeit* ) ) OR “routine data” OR “administrative data” OR “secondary data” OR claims ) ) ) ) ) AND NOT ( ( TITLE ( ( postoperat* OR "post-operat*" OR "post-op" OR postsurg* OR "post-surg*" OR animal OR animals OR canine* OR dog OR dogs OR feline OR hamster* OR lamb OR lambs OR mice OR monkey OR monkeys OR mouse OR murine OR pig OR pigs OR piglet* OR porcine OR primate* OR rabbit* OR rats OR rat OR rodent* OR sheep*) ) ) ) AND ( ( ( PUBYEAR > 1999 ) ) ) AND ( LIMIT-TO ( LANGUAGE,"English" ) OR LIMIT-TO ( LANGUAGE,"German" ) )

## PEDro.

For the PEDro database, we performed 17 advanced searches of the following keywords separately:

osteoarthr* ambulatory indicator*

outpatient guideline*

conservative recommendation*

“non surgical”

“first line”

“evidence based”

## List of organisations and institutions.

Accreditation Association for Ambulatory Health Care Institute for Quality Improvement (AAAHC)

American Academy of Pain Medicine (AAPM)

African League of Associations for Rheumatology (AFLAR)

Agency for Healthcare Research and Quality (AHRQ)

American Academy of Family Physicians (AAFP)

American Academy of Orthopaedic Surgeons (AAOS)

American Association of Hip and Knee Surgeons (AAHKS)

American College of Rheumatology (ACR)

American College of Surgeons (ACS)

American Geriatrics Society (AGS)

American Medical Association (AMA)

American Physical Therapy Association (APTA)

Arthritis Society Canada (ASC)

Asia Pacific League of Associations for Rheumatology (APLAR)

Association of the Scientific Medical Societies in Germany (AWMF)

Australian Commission on Safety and Quality in Health Care (ACSQHC)

Australian Institute of Health and Welfare (AIHW)

Brazilian Society of Rheumatology (BSR)

British Society for Rheumatology (BSR)

Canadian Rheumatology Association (CRA)

Centers for Disease Control and Prevention (CDC)

Commonwealth Fund (CWF)

Department of Veterans Affairs (DVA)

Dutch College of General Practitioners (NHG)

European League Against Rheumatism (EULAR)

European Society for Clinical and Economic Aspects of Osteoporosis, Osteoarthritis and Musculoskeletal Diseases (ESCEO)

French Society of Rheumatology (SFR)

German Agency for Quality in Medicine (ÄZQ)

Guidelines International Network (GIN)

Hong Kong Society of Rheumatology (HKSR)

Institute for Applied Quality Improvement and Research in Health Care GmbH (aQua-Institute)

Institute for Clinical Systems Improvement (ISCI)

International League of Associations for Rheumatology (ILAR)

Italian Society for Rheumatology (SIR)

Michigan Quality Improvement Consortium (MQIC)

National Association of Statutory Health Insurance Funds (KBV)

National Center for Complementary and Integrative Health (NCCIH)

National Committee for Quality Assurance (NCQA)

National Health and Medical Research Council (NHMRC)

National Health Service (NHS)

National Institute for Health and Care Excellence (NICE)

National Quality Forum (NQF)

Orthopedic Research Society (ORS)

Osteoarthritis Research Society International (OARSI)

Pan-American League of Associations for Rheumatology (PANLAR)

Pharmacy Quality Alliance (PQA)

Queensland Clinical Guidelines (QCG)

Research and Development Corporation (RAND)

Royal Dutch Society for Physiotherapy (KNGF)

Scottish Intercollegiate Guidelines Network (SIGN)

South African Rheumatism and Arthritis Association (SARAA)

Turkish League Against Rheumatism (TLAR)
