# Supplementary material for: Routine‐data‐compatible quality indicators for the ambulatory care of osteoarthritis of the knee and hip: A systematic review
Source: Knee Surg Sports Traumatol Arthrosc. 2025 Feb 13;33(10):3523–41. doi: 10.1002/ksa.12614 (PMC12459328; doi:10.1002/ksa.12614)
Supplement: Supplementary file 2 — Supporting information. [file KSA-33-3523-s003.docx]

**APPENDIX 2.**

Data extraction: QI sets.

**Asch SM, et al. (1)**

| **Characteristics** | | | |
| --- | --- | --- | --- |
| Year | 2004 | | |
| Country | US | | |
| OA Target population | Patients of outpatient and inpatient care (that is, screening,  diagnosis, treatment, and follow-up) for acute and chronic conditions and preventive care processes representing the leading causes of morbidity, death, and health care use among older male patients (including patients with OA). | | |
| Evidence synthesis | Literature review, not specified to be systematic | | |
| Consensus method | RAM-modified Delphi consensus | | |
| **OA quality indicators (3)** | | **Dimension of quality** | **Ambulatory health intervention before TJR quantifiable in routine data from German SHIs** |
| Providers caring for patients with symptoms of osteoarthritis should document all of the following at least once in 2 years: the location of symptoms and/or the presence or absence of limitations in daily activities. | | Process | Physician visit for musculoskeletal disorders |
| Patients with a new diagnosis of osteoarthritis who wish to take medication for joint symptoms should be offered a trial of acetaminophen. | | Process | Paracetamol (initial medication) |
| Providers caring for patients with symptoms of hip or knee osteoarthritis should recommend exercise programs at least once in 2 years. | | Process | Exercise therapy/  referral to PT |

**Barber CE, et al. (2)**

| **Characteristics** | | | |
| --- | --- | --- | --- |
| Year | 2015 | | |
| Country | Canada | | |
| Target population | Patients with moderate to severe OA who required either surgical (total hip or knee arthroplasty) or nonsurgical management (requiring specialist consultation) | | |
| Evidence synthesis | Integrative review including an update of a systematic review of the literature conducted by the European Musculoskeletal Conditions Surveillance and Information Network in two literature databases (MEDLINE and Embase) | | |
| Consensus method | RAM-modified Delphi consensus | | |
| **OA quality indicators (18)** | | **Dimension of quality** | **Ambulatory health intervention before TJR quantifiable in routine data from German SHIs** |
| Time from OA referral receipt to referral completion for initially incomplete referrals | | Process |  |
| Percentage of OA referrals received with complete information | | Process |  |
| Time from receipt of complete OA referral to musculoskeletal appointment | | Process | Physician visit for musculoskeletal disorders |
| Percentage of patients who receive information regarding resources and tools available for management while waiting for first musculoskeletal specialty contact | | Process |  |
| Percentage of osteoarthritis referrals scored using Western Canada Criteria Waiting List priority referral | | Process |  |
| Distribution of OA referrals in each urgency category (as scored using the Western Canada Waiting List referral tool) | | Process |  |
| Percentage of OA referrals triaged as highest urgency based on high Western Canada Waiting List priority criteria scores seen within Wait Time Alliance benchmarks | | Process |  |
| Percentage of referrals rejected or redirected when received at centralized intake | | Process |  |
| Percentage of musculoskeletal appointments completed as scheduled | | Process |  |
| Percentage of specialist providers participating in centralized intake | | Process |  |
| Number of referrals received through centralized intake | | Process |  |
| Patient experience with centralized intake | | Outcome |  |
| Referring clinician experience with centralized intake | | Structure |  |
| Musculoskeletal specialty care provider experience with centralized intake | | Structure |  |
| Ratio of patient flow to estimated clinic capacity of osteoarthritis teams participating in centralized intake | | Process |  |
| Operating room time for arthroplasty surgeons in Alberta | | Process |  |
| Administrative staff and allied health professional experience with centralized intake | | Structure |  |
| Agreement of centralized intake suspected diagnosis of severe OA cases (e.g., patients who are candidates for hip or knee joint replacements) versus confirmed diagnosis of severe OA | | Outcome |  |

**Doubova SV, et al. (3)**

| **Characteristics** | | | |
| --- | --- | --- | --- |
| Year | 2015 | | |
| Country | Mexico | | |
| Target population | KOA/HOA patients older than 19 years | | |
| Evidence synthesis | Literature review, not specified to be systematic | | |
| Consensus method | RAM-modified Delphi consensus | | |
| **OA quality indicators (6)** | | **Dimension of quality** | **Ambulatory health intervention before TJR quantifiable in routine data from German SHIs** |
| (Patients with knee/hip OA who have documented recommendations for general aerobic and/or muscle strengthening exercise at least once per year, unless contraindicated (e.g. significant heart failure)/ Total number of patients with KHOA without contraindications for general aerobic exercise) * 100 | | Process | Exercise therapy/  referral to PT |
| (Overweight (BMI ≥27 kg/m2) patients with KHOA who have documented nutritional counselling provided by the Nutrition and Dietary Service and/or who were encouraged by their family physician at least one time per year to lose weight/ Total number of overweight patients with KHOA) * 100 | | Process |  |
| (Patients with newly diagnosed of KHOA who received prescription of acetaminophen as initial oral analgesic, unless* contraindicated/ Total number of patients with recent diagnosis of KHOA) * 100 | | Process | Paracetamol (initial medication) |
| (Patients aged 65 years or older with KHOA and one of the following comorbidities (history of peptic ulcer disease or gastrointestinal bleeding, chronic kidney disease, cardiac insufficiency and/or those receiving anticoagulant or glucocorticoids) who receive NSAID prescription/ Total number of patients aged 65 years or older with KHOA and one of the previously mentioned comorbidities)* 100 | | Process | Oral NSAID treatment with caution for comorbidities (GI/CV/renal) |
| (Patients with KHOA and high risk for gastrointestinal complications who received NSAID prescription concomitant with either misoprostol or a proton-pump inhibitor/ Total number of patients with KHOA and high risk of gastrointestinal complications who received NSAIDs) * 100 | | Process | Oral NSAID treatment with concomitant PPI/ misoprostol in case of Gl risk factors |
| (Patients with KHOA and with NSAID prescription for 6 months or longer who were referred for the following laboratory tests (blood count, serum creatinine and liver enzymes) at least once in the previous 12 months / Total number of patients with KHOA and with NSAID prescription for 6 months or longer) * 100 | | Process |  |

**EUMUSC.net (4)**

| **Characteristics** | | | |
| --- | --- | --- | --- |
| Year | 2012 | | |
| Country | not specified (Europe) | | |
| Target population | OA patients aged 18 years or older | | |
| Evidence synthesis | Systematic review for the development of standards of care | | |
| Consensus method | Translation of the standards of care into QIs and refinement by sixteen eumusc.net researchers and patient representatives from various European countries (Norway, Poland, Finland, Spain, Sweden, Italy, Denmark, the Netherlands and the UK) | | |
| **OA quality indicators (12)** | | **Dimension of quality** | **Ambulatory health intervention before TJR quantifiable in routine data from German SHIs** |
| If a patient is diagnosed with symptomatic osteoarthritis (OA), then he or she should be assessed for pain, functional ability, level of physical activity, body mass index (BMI), and labor force participation at baseline or when experiencing significant pain or functional limitation. | | Process | Physician visit for musculoskeletal disorders |
| If a patient with symptomatic OA is prescribed NSAID or aspirin then GI bleeding risk, CVD risks, and renal risks should be assessed. | | Process | Oral NSAID treatment with caution for comorbidities (GI/CV/renal) |
| If a patient with OA is overweight (as defined by a BMI > 27), then he or she should receive information on weight management and offered referral to a weight management program. | | Process |  |
| If a patient is newly diagnosed with symptomatic OA, then, he or she should be given individually tailored education by relevant health professionals about the natural history, treatment, and self-management of the disease within 3 months. | | Process |  |
| If a patient is diagnosed with symptomatic OA then a referral to a relevant health professional for instruction in an individualized exercise program including advice for physical activity, range of motion‐, muscle strengthening‐ and aerobic exercises should be provided within 3 months. | | Process | Exercise therapy/  referral to PT |
| If a patient with a diagnosis of symptomatic OA reports difficulties in ambulatory and/or non-ambulatory activities of daily living the need for assistive devices, orthoses, and environmental adaptations should be assessed and addressed. | | Process | Walking aids;  Orthoses/braces |
| All professionals managing patients with OA at a primary health care centre should have continuous access to education on important preventive and therapeutic strategies in the management of OA. | | Process |  |
| If a patient has a diagnosis of symptomatic OA and has failed to respond to pharmacological and non-pharmacological therapy then the patient should be referred to an orthopedic surgeon when relevant. | | Process | Referral to orthopaedic surgeon if conservative therapy failed |
| If a patient is diagnosed with OA and has been referred to an orthopedic surgeon, then the waiting time from first referral should not exceed three months. | | Process | Referral to orthopaedic surgeon if conservative therapy failed |
| If a patient is diagnosed with symptomatic OA and has functional limitation then an improvement of his/her functional ability by 20% on a patient reported outcome measure should be reached within three months after initiation/change of pharmacological/non pharmacological treatment. | | Outcome |  |
| If a patient is diagnosed with symptomatic OA then his/her pain level should be reduced by 20% on a patient reported outcome measure within three months after initiation/change of pharmacological/non pharmacological treatment. | | Outcome |  |
| If an individual of working age is diagnosed with symptomatic OA, then he/she should be enabled to participate in the labor market. | | Outcome |  |

**Grypdonck L, et al. (5)**

| **Characteristics** | | | |
| --- | --- | --- | --- |
| Year | 2014 | | |
| Country | Belgium | | |
| Target population | KOA patients across the entire spectrum of disciplines involved in KOA care | | |
| Evidence synthesis | Literature review, not specified to be systematic | | |
| Consensus method | RAM-modified Delphi consensus | | |
| **OA quality indicators (22)** | | **Dimension of quality** | **Ambulatory health intervention before TJR quantifiable in routine data from German SHIs** |
| If a patient is clinically diagnosed with knee OA and suffering from pain resistant to conservative treatment with acetaminophen and/or NSAID, then a radiography (weight-bearing, semiflexed PA, plus lateral and skyline view) of the symptomatic knee should be taken for the morphological assessment and grading of knee OA (especially to detect unicompartmental OA, for which treatment modalities may differ). CT and MRI scan should not be used. | | Process | Radiographic assessment  (prior to CT, MRI, sonography) |
| If a patient with knee OA has a recurrent clinically evident effusion, then he/she should be further assessed (with aspiration and analysis of synovial fluid) in order to differentiate from inflammation caused by other arthritis. | | Process |  |
| If a patient has knee OA, he/she should be given information access and education about the objectives of treatment and the importance of changes in lifestyle, exercise, pacing of activities, weight reduction, and other measures to unload the damaged joints. | | Process |  |
| If a patient with knee OA is overweight, then he/she should be encouraged to lose weight and maintain his/her weight at a lower level. | | Process |  |
| If a patient with knee OA is following exercise therapy, then the exercise therapy should be combined with education/self-management interventions to improve patients’ mental and physical performance and to alleviate pain. | | Process |  |
| If a patient has knee OA, then a brace should not be prescribed (except in unicompartmental knee OA with axial deviation). | | Process | Orthoses/braces |
| If a patient has knee OA, then exercise therapy should be prescribed, including at least muscle strengthening, aerobic exercises and functional exercises, and combined with range of motion exercises in case of range of motion restrictions. | | Process | Exercise therapy/  referral to PT |
| If a patient has symptomatic knee OA, then he/she has to be referred to a physical therapist for instruction of the patient in appropriate exercises, for motivation of the patient to implement exercise and adhere to exercise, and to evaluate performance. | | Process | Exercise therapy/  referral to PT |
| If a patient with knee OA is following exercise therapy, then the content and intensity of the exercise program should be tailored to the patient’s individual goals in terms of limitations of activity and restrictions of participation. | | Process |  |
| If a patient with knee OA is following exercise therapy, then the treatment sessions should be spread over longer periods with lower frequencies in the later stages of the exercise program to facilitate the transition from exercise therapy to independent exercising and maintaining sufficient level of physical activity. | | Process |  |
| If a patient with knee OA is following exercise therapy, then he/she should be referred to regular community exercise and sports activities after a period of supervised exercise. | | Process |  |
| If a patient has knee OA, then acetaminophen up to 3 g/day should be used as the initial oral analgesic. | | Process | Paracetamol (initial medication) |
| If a patient has knee OA and there is no adequate response on acetaminophen, or there is severe pain and/or inflammation, then oral NSAID should be used. | | Process | Oral NSAID |
| If a patient has knee OA, then chondroitin and glucosamine-chondroitin combination products should not be used. | | Process |  |
| If NSAID are used in a patient with knee OA, then they should be used intermittently (max 3 weeks sustained use) and at the lowest effective dose. | | Process | Oral NSAID |
| If a patient with knee OA and a history of bleeding gastric ulcers has a need for NSAID, then either a COX-2 selective agent or a non-selective NSAID with coprescription of a proton pump inhibitor/misoprostol should be used instead of a non-selective NSAID. | | Process | Oral NSAID treatment with concomitant PPI/ misoprostol in case of Gl risk factors |
| If a patient with knee OA has heart failure grade 2–4, ischemic heart disease, or renal insufficiency with a GFR < 40 ml/min, then NSAID should not be used. In case of other cardiovascular risk factors (e.g., hypertension), NSAID should be used with caution. | | Process | Oral NSAID treatment with caution for comorbidities (GI/CV/renal) |
| If a patient has knee OA, then strong opioids (oxymorphone, oxycodone, fentanyl, morphine sulfate) should not be used. | | Process | Strong opioids |
| If a patient has knee OA, then arthroscopic interventions are not recommended. Coexisting meniscal lesions should not be treated. Only in case of locking of the knee from a large meniscal fragment or a loose body or an extension loss from an anterior anvil osteophyte is arthroscopic treatment indicated. | | Process |  |
| If a patient with knee OA is not obtaining adequate pain relief and functional improvement, then he/she should be considered for joint replacement. | | Process | Referral to orthopaedic surgeon if conservative therapy failed |
| If a patient has unicompartmental knee OA, then a unicompartmental knee replacement should be considered. | | Process |  |
| If a patient with knee OA is following exercise therapy, then regular evaluations by the physiotherapist are necessary. To make the switchover from a supervised to an autonomous program, an evaluation session should be performed every 3 months in the first year, every 6 months in the second year, and once per year afterward. | | Process |  |

**Jansen MJ, et al. (6)**

| **Characteristics** | | | |
| --- | --- | --- | --- |
| Year | 2010 | | |
| Country | The Netherlands | | |
| Target population | KOA/HOA patients in physiotherapy care | | |
| Evidence synthesis | QIs derived from Dutch physiotherapy guideline on KOA and/or HOA (21) | | |
| Consensus method | QIs independently assessed by two people | | |
| **OA quality indicators (17)** | | **Dimension of quality** | **Ambulatory health intervention before TJR quantifiable in routine data from German SHIs** |
| Problem areas recorded (i.e. inflammation, pain, impairments of function, activity limitations, participation restrictions, passive coping behaviour)  (benchmark >90%) | | Process | Physician visit for musculoskeletal disorders |
| Classification recorded (patient profile A: acute complaints, patient profile B: episodic complaints, patient profile C: chronic complaints)  (benchmark >90%) | | Process |  |
| Visual Analog Scale (VAS) measurements for severity of pain and Algofunctional Index (AI) measurements at baseline, at 6 weeks and at the end of the treatment episode  (benchmark >90%) | | Process |  |
| Information and advice  (benchmark >90%) | | Process |  |
| Exercise therapy for functions  (benchmark >90%) | | Process | Exercise therapy/  referral to PT |
| Exercise therapy for activities  (benchmark >90%) | | Process | Exercise therapy/  referral to PT |
| Avoidance of physical modalities with the exception of TENS (e.g. pulsed shortwave)  (benchmark <10%) | | Process | TENS |
| Avoidance of massage therapy  (benchmark <10%) | | Process | Massage |
| Aftercare (e.g. home exercise programme, follow-up consultation with the physical therapist, advice to remain active of participation in a special exercise programme for the elderly)  (benchmark >90%) | | Process |  |
| Decrease in VAS-pain scores (≤ 25%)  (No specific benchmark reported) | | Outcome |  |
| Decrease in AI ≤ 25%  (No specific benchmark reported) | | Outcome |  |
| The extent to which the treatment goals were achieved (No specific benchmark reported) | | Outcome |  |
| Number of treatment sessions  (No specific benchmark reported) | | Outcome |  |
| Duration of treatment episode less than 6 weeks  (No specific benchmark reported) | | Outcome |  |
| Treatment frequency (sessions/week) (No specific benchmark reported) | | Outcome |  |
| Patient’s satisfaction with treatment care  (No specific benchmark reported) | | Outcome |  |
| Global perceived on restrictions in daily activities (5 point Likert scale) (No specific benchmark reported) | | Outcome |  |

**Kleudgen S, et al. (7)**

| **Characteristics** | | | |
| --- | --- | --- | --- |
| Year | 2009 | | |
| Country | Germany | | |
| Target population | OA patients | | |
| Evidence synthesis | Systematic review | | |
| Consensus method | RAM-modified Delphi consensus | | |
| **OA quality indicators (4)** | | **Dimension of quality** | **Ambulatory health intervention before TJR quantifiable in routine data from German SHIs** |
| Anteil der Patienten mit der Diagnose Arthrose, bei denen die Einnahme frei verkäuflicher Analgetika erhoben wurde. | | Process |  |
| Anteil der Patienten mit einer oralen Pharmakotherapie zur Behandlung einer Arthrose, die Paracetamol als Medikament der ersten Wahl erhielten, außer es bestand eine dokumentierte Kontraindikation. | | Process | Paracetamol (initial medication) |
| Anteil der Patienten mit der Diagnose einer symptomatischen Arthrose des Knies oder der Hüfte mit einer jährlichen Schmerzbestimmung. | | Process | Physician visit for musculoskeletal disorders |
| Anteil der übergewichtigen Patienten mit symptomatischer Arthrose des Knies oder der Hüfte, denen empfohlen wurde, Gewicht abzunehmen, um die Beschwerden der Arthrose zu vermindern und in deren Akte dies dokumentiert ist. | | Process |  |

**MacLean CH, et al. (8)**

| **Characteristics** | | | |
| --- | --- | --- | --- |
| Year | 2007 | | |
| Country | US | | |
| Target population | Vulnerable elders with OA | | |
| Evidence synthesis | Systematic Review | | |
| Consensus method | RAM-modified Delphi consensus | | |
| **OA quality indicators (13)** | | **Dimension of quality** | **Ambulatory health intervention before TJR quantifiable in routine data from German SHIs** |
| IF a VE is obese (body mass index (BMI) ≥30kg/m2), THEN he or she should be advised annually to lose weight, BECAUSE weight loss reduces the risk of developing symptomatic knee and hip OA. | | Process |  |
| IF a VE has symptomatic OA of the knee or hip, THEN pain should be assessed when new to a primary care or musculoskeletal disease practice and annually. | | Process | Physician visit for musculoskeletal disorders |
| IF a VE has symptomatic OA of the knee or hip, functional status should be assessed when new to a primary care or musculoskeletal disease practice and annually, BECAUSE this information should direct therapeutic decisions. | | Process | Physician visit for musculoskeletal disorders |
| IF an ambulatory VE has symptomatic OA of the knee or hip for longer than 3 months and is able to exercise, THEN a directed or supervised muscle strengthening or aerobic exercise program should be recommended and activity reviewed annually, BECAUSE directed or supervised exercise programs improve functional status and reduce pain. | | Process | Exercise therapy/  referral to PT |
| IF a VE has symptomatic OA of the hip or knee and has difficulty walking that makes activities of daily living difficult for longer than 3 months, THEN the need for ambulatory assistive devices should be assessed, BECAUSE these devices will improve walking and help maintain function and independence. | | Process | Walking aids |
| IF a VE has symptomatic OA and has difficulty with nonambulatory activities of daily living, THEN the need for ADL assistive devices should be assessed, BECAUSE such devices will help to maintain function and independence. | | Process |  |
| IF a VE is started on pharmacological therapy to treat OA, THEN acetaminophen should be tried first, BECAUSE acetaminophen achieves pain relief comparable to that of an NSAID (nonselective and selective) for many patients and is associated with a lower burden of common serious adverse events. | | Process | Paracetamol (initial medication) |
| IF a VE is prescribed chronic high-dose acetaminophen (≥3 g/d) or a VE with liver disease is prescribed chronic acetaminophen, THEN he or she should be advised of the risk of liver toxicity, BECAUSE these risks are greater with high doses of acetaminophen and when underlying liver disease is present. | | Process | Paracetamol (initial medication) |
| IF a VE is prescribed an NSAID (non-selective or selective), THEN GI bleeding risks should be discussed and documented, BECAUSE selective NSAIDs, non-selective NSAIDs, and aspirin increase the risk of bleeding. | | Process | Oral NSAID treatment with caution for comorbidities (GI/CV/renal) |
| IF a VE is prescribed daily aspirin (including low-dose, <325 mg/d), THEN GI bleeding risks should be discussed and documented, BECAUSE selective NSAIDs, non-selective NSAIDs, and aspirin increase the risk of bleeding. | | Process |  |
| IF a VE with a risk factor for GI bleeding (aged ≥75, peptic ulcer disease, history of GI bleeding, warfarin use, chronic glucocorticoid use) is treated with a nonselective NSAID, THEN he or she should be treated concomitantly with misoprostol or a proton pump inhibitor (PPI). | | Process | Oral NSAID treatment with concomitant PPI/misoprostol in case of Gl risk factors |
| IF a VE with two or more risk factors for GI bleeding (aged ≥75, peptic ulcer disease, history of GI bleeding, warfarin use, chronic glucocorticoid use) is treated with daily aspirin, THEN he or she should be treated concomitantly with either misoprostol or a PPI, BECAUSE this will reduce the risk of GI bleeding. | | Process |  |
| IF a VE has severe symptomatic OA of the knee or hip despite nonsurgical therapy, THEN a referral to an orthopedic surgeon should be made, BECAUSE joint surgery may reduce pain and improve functional status and quality of life. | | Process | Referral to orthopaedic surgeon if conservative therapy failed |

**McGlynn EA, et al. (9)**

| **Characteristics** | | | |
| --- | --- | --- | --- |
| Year | 2003 | | |
| Country | US | | |
| Target population | Adults living in 12 metropolitan areas in the US | | |
| Evidence synthesis | Literature review, not specified to be systematic | | |
| Consensus method | RAM-modified Delphi consensus | | |
| **OA quality indicators (3)** | | **Dimension of quality** | **Ambulatory health intervention before TJR quantifiable in routine data from German SHIs** |
| Providers caring for patients with symptoms of OA should document all at least one of the following at least once in 2 years: the location of symptoms, and/or the presence or absence of limitations in daily activities. | | Process | Physician visit for musculoskeletal disorders |
| Patients with a new diagnosis of OA who wish to take medication for joint symptoms should be offered a trial of acetaminophen. | | Process | Paracetamol (initial medication) |
| Providers caring for patients with symptoms of hip or knee OA should recommend exercise programs at least once in 2 years. | | Process | Exercise therapy/  referral to PT |

**Moore A (10)**

| **Characteristics** | | | |
| --- | --- | --- | --- |
| Year | 2000 | | |
| Country | US | | |
| Target population | OA patients | | |
| Evidence synthesis | Literature review, not specified to be systematic | | |
| Consensus method | RAM-modified Delphi consensus | | |
| **OA quality indicators (7)** | | **Dimension of quality** | **Ambulatory health intervention before TJR quantifiable in routine data from German SHIs** |
| Providers caring for patients with symptoms of OA should document all of the following at least once in 2 years:  a. the location of symptoms;  b. the presence or absence of limitations in daily activities;  c. the presence or absence of a history or symptoms of systemic or inflammatory disease;  d. the use and effectiveness of treatment modalities. | | Process | Physician visit for musculoskeletal disorders |
| Providers caring for patients with incident symptoms of OA should document at least one of the following:  • the presence or absence of a history of any systemic or inflammatory disease that may mimic OA;  • the presence or absence of any current symptoms of systemic or inflammatory disease that may mimic OA;  • the presence or absence of a history of joint trauma or surgery. | | Process | Physician visit for musculoskeletal disorders |
| Providers caring for patients with symptoms of OA should document the following for any one affected joint at least once in 2 years:  a. the presence or absence of effusion;  b. the presence or absence of bony enlargement;  c. the presence or absence of tenderness;  d. the presence or absence of limitations in range of motion. | | Process | Physician visit for musculoskeletal disorders |
| Patients with incident symptoms of hip OA should be offered an anteroposterior film of the affected hip. | | Process |  |
| Patients with a new diagnosis of OA who wish to take medication for joint symptoms should be offered a trial of acetaminophen. | | Process | Paracetamol (initial medication) |
| Providers caring for patients with symptoms of hip or knee OA should recommend both of the following at least once in 2 years:  a. exercise programs for persons with hip or knee OA;  b. weight loss among persons with knee OA and a BMI >25. | | Process | Exercise therapy/  referral to PT |
| Patients receiving care for symptoms of OA should be seen in follow-up at least every 6 months. | | Process | Physician visit for musculoskeletal disorders |

**Østerås N, et al. (11)**

| **Characteristics** | | | |
| --- | --- | --- | --- |
| Year | 2018 | | |
| Country | Norway | | |
| Target population | OA patients | | |
| Evidence synthesis | Literature review, not specified to be systematic | | |
| Consensus method | Refinement of QIs in expert panels and patient interviews | | |
| **OA quality indicators (16)** | | **Dimension of quality** | **Ambulatory health intervention before TJR quantifiable in routine data from German SHIs** |
| Have you been given information about osteoarthritis from a health professional? | | Process |  |
| Have you been given information about different treatment alternatives? | | Process |  |
| Have you been given information about how you can self-manage the disease? | | Process |  |
| Have you been given information about the importance of physical activity and exercise? | | Process |  |
| Have you been referred or offered a referral to a health professional who can advise you about physical activity and exercise? | | Process | Exercise therapy/  referral to PT |
| Have you been advised to lose weight, if you are overweight? | | Process |  |
| Have you been referred or offered a referral to someone who can help you to lose weight, if you are overweight? | | Process |  |
| If you have problems with daily activities, have these problems been assessed by a health professional? | | Process | Physician visit for musculoskeletal disorders |
| If you have joint pain, has it been assessed by a health professional? | | Process | Physician visit for musculoskeletal disorders |
| If you have problems with walking, has your need for a walking aid been assessed? (e.g., stick, crutch or walker) | | Process | Walking aids |
| If you have problems related to other daily activities, has your need for appliances and aids been assessed? (e.g., splints, assistive technology for cooking or personal hygiene, a special chair) | | Process |  |
| If you have joint pain, was paracetamol the first medication that was recommended? | | Process | Paracetamol (initial medication) |
| If you have prolonged severe joint pain, which is not relieved sufficiently by paracetamol, have you been offered stronger pain killing medications? (e.g., co-codamol, codeine, tramadol, co-proxamol, co-dydramol, dihydrocodeine) | | Process | Weak opioids |
| If you use anti-inflammatory medications, have you been given information about the effects and possible side-effects of this medication? (e.g., ibuprofen (Nurofen, Brufen), diclofenac (Voltarol), naproxen (Naprosyn), celecoxib (Celebrex)) | | Process |  |
| If you have experienced an acute deterioration of your symptoms, have you been given or offered a steroid injection? | | Process | Corticosteroid injection |
| If you are severely troubled by your osteoarthritis, and exercise and medication do not help, have you been referred or offered a referral for an assessment for operation? (e.g., joint replacement) | | Process | Referral to orthopaedic surgeon if conservative therapy failed |

**PCPI (12)**

| **Characteristics** | | | |
| --- | --- | --- | --- |
| Year | 2006 | | |
| Country | US | | |
| Target population | OA patients aged 21 years or older | | |
| Evidence synthesis | not specified | | |
| Consensus method | not specified | | |
| **OA quality indicators (7)** | | **Dimension of quality** | **Ambulatory health intervention before TJR quantifiable in routine data from German SHIs** |
| Percentage of patient visits for patients aged 21 years and older with a diagnosis of OA with an assessment for use of anti-inflammatory or analgesic OTC medications | | Process | Paracetamol (initial medication),  oral NSAID |
| Percentage of patient visits for patients aged 21 years and older with a diagnosis of OA during which GI prophylaxis was considered | | Process | Oral NSAID treatment with concomitant PPI/misoprostol in case of Gl risk factors |
| Percentage of patient visits for patients aged 21 years and older with a diagnosis of osteoarthritis with assessment for function and pain | | Process | Physician visit for musculoskeletal disorders |
| Percentage of patients aged 21 years and older with a diagnosis of OA for whom a physical examination of the involved joint was performed during the initial visit | | Process | Physician visit for musculoskeletal disorders |
| Percentage of patients aged 21 years and older with a diagnosis of OA on prescribed or OTC NSAIDs who were assessed for GI and renal risk factors | | Process | Oral NSAID treatment with caution for comorbidities (GI/CV/renal) |
| Percentage of patient visits for patients aged 21 years and older with a diagnosis of OA during which an anti-inflammatory agent or analgesic was considered | | Process |  |
| Percentage of patient visits for patients aged 21 years and older with a diagnosis of OA of the hip or knee during which therapeutic exercise for the hip or knee (therapeutic exercise instructed or physical therapy prescribed) was considered | | Process | Exercise therapy/  referral to PT |

**Peter WF, et al. (13)**

| **Characteristics** | | | |
| --- | --- | --- | --- |
| Year | 2013 | | |
| Country | The Netherlands | | |
| Target population | KOA/HOA patients in physiotherapy care | | |
| Evidence synthesis | QIs based on key recommendations of the Dutch physiotherapy guideline on cox- and gonarthrosis (22) | | |
| Consensus method | Recommendations rated by an expert panel of physical therapists (n=16) | | |
| **OA quality indicators (19)** | | **Dimension of quality** | **Ambulatory health intervention before TJR quantifiable in routine data from German SHIs** |
| Inventory of health-related problems according to the International Classification of Functioning, Disability and Health (ICF) | | Process |  |
| Assessing the presence of personal and environmental problems in so far as these relate to the limitations in activities and restrictions in participation | | Process |  |
| Assessing the presence of hip and knee OA-specific ‘red flags’ | | Process | Physician visit for musculoskeletal disorders |
| Treating patients with strengthening of muscles | | Process | Exercise therapy/  referral to PT |
| Treating patients with improving of aerobic capacity | | Process | Exercise therapy/  referral to PT |
| Treating patients with walking exercises | | Process | Exercise therapy/  referral to PT |
| Treating patients with functional exercises | | Process | Exercise therapy/  referral to PT |
| Treating patients with postoperative exercises | | Process |  |
| Providing information concerning knowledge and understanding of OA of the hip and/or knee | | Process |  |
| Providing information concerning the consequences for the patient’s functional performance in terms of movements, activities and participation | | Process |  |
| Providing information concerning the relationship between burden and tolerance level | | Process |  |
| Providing information concerning the way a patient copes with health problems | | Process |  |
| Providing information concerning what constitutes an active and healthy lifestyle (in terms of exercise and nutrition/overweight | | Process |  |
| Providing information concerning behavioural change (regarding physical activity) | | Process |  |
| Providing information concerning joint protection and the use of aids | | Process |  |
| Evaluating treatment with the recommended measurement instruments | | Process |  |
| Evaluating treatment with the combination of a questionnaire and a performance test | | Process |  |
| Evaluating treatment with a patient-specific complaint list | | Process |  |
| Evaluating treatment with the Timed Up and Go test (TUG) | | Process |  |

**Saliba D, et al. (14)**

| **Characteristics** | | | |
| --- | --- | --- | --- |
| Year | 2004 | | |
| Country | US | | |
| Target population | Vulnerable elders in US nursing homes | | |
| Evidence synthesis | Adaption of earlier published QIs (23) for the use in US nursing homes | | |
| Consensus method | Modified Delphi-process with a panel of nursing home experts | | |
| **OA quality indicators (7)** | | **Dimension of quality** | **Ambulatory health intervention before TJR quantifiable in routine data from German SHIs** |
| IF a NH resident has a new joint pain that is reported to the primary care provider THEN the joint and periarticular structures should be examined within 1 month or there should be documentation that the problem has resolved. | | Process | Physician visit for musculoskeletal disorders |
| IF a non-OTC drug is newly prescribed to treat new joint pain THEN evidence that the affected joint was examined should be documented within 4 weeks. | | Process | Physician visit for musculoskeletal disorders |
| IF a NH resident has monoarticular joint pain associated with redness, warmth, and/or swelling and the patient also has an oral temperature >38.0°C, and does not have a previously established diagnosis of pseudogout or gout THEN a diagnostic aspiration of the painfully swollen red joint should be performed that day. | | Process |  |
| IF an ambulatory NH resident is newly diagnosed with symptomatic osteoarthritis of the knee and has no contraindication to exercise and is physically and mentally able to exercise THEN a directed or supervised strengthening or aerobic exercise program should be prescribed within 1 month of diagnosis. | | Process | Exercise therapy/  referral to PT |
| IF an ambulatory NH resident has a diagnosis of symptomatic knee osteoarthritis for >3 months, has no contraindication to exercise, and is physically and mentally able to exercise THEN there should be evidence that a directed or supervised strengthening or aerobic exercise program was prescribed at least once since the time of diagnosis. | | Process | Exercise therapy/  referral to PT |
| IF oral pharmacologic therapy is initiated to treat osteoarthritis THEN acetaminophen should be the first drug used. | | Process | Paracetamol (initial medication) |
| IF oral pharmacologic therapy for symptomatic osteoarthritis is changed from acetaminophen to a different oral agent THEN there should be evidence that the NH resident has had a trial of maximum dose acetaminophen (suitable for age and comorbid conditions). | | Process | Paracetamol (initial medication) |

**Smith KL, et al. (15)**

| **Characteristics** | | | |
| --- | --- | --- | --- |
| Year | 2007 | | |
| Country | US | | |
| Target population | Homebound elderly patients in home-based primary care | | |
| Evidence synthesis | Adaption of earlier published QIs (14, 23) for the use in home-based primary care | | |
| Consensus method | Modified Delphi-process with a panel of nursing home experts | | |
| **OA quality indicators (7)** | | **Dimension of quality** | **Ambulatory health intervention before TJR quantifiable in routine data from German SHIs** |
| IF an ambulatory homebound patient is newly diagnosed with osteoarthritis of the knee, has no contraindication to exercise, and is physically and mentally able to exercise, THEN a directed or supervised strengthening or aerobic exercise program should be prescribed within 3 months of diagnosis. | | Process | Exercise therapy/  referral to PT |
| IF an ambulatory homebound patient has had a diagnosis of symptomatic osteoarthritis of the knee for longer than 12 months and is physically and mentally able to exercise, THEN there should be evidence that a physical therapy evaluation for focused strengthening exercises was prescribed at least once since the time of diagnosis. | | Process | Exercise therapy/  referral to PT |
| IF oral pharmacologic therapy is initiated to treat osteoarthritis in a homebound patient, THEN acetaminophen should be the first drug used, unless there is a documented contraindication to use | | Process | Paracetamol (initial medication) |
| IF oral pharmacologic therapy for osteoarthritis in a homebound patient is changed from acetaminophen to a different oral agent, THEN there should be evidence that the patient has had a trial of maximum-dose acetaminophen (suitable for age and comorbid conditions). | | Process | Paracetamol (initial medication) |
| IF a patient is treated with a COX-nonselective NSAID, THEN there should be evidence that the patient was advised of the risk for gastrointestinal bleeding, as well as cardiovascular risk associated with these drugs. | | Process | Oral NSAID treatment with caution for comorbidities (GI/CV/renal) |
| IF a homebound patient is diagnosed with symptomatic osteoarthritis, THEN his or her functional status and the degree of pain should be assessed at each visit. | | Process | Physician visit for musculoskeletal disorders |
| IF a homebound patient has monoarticular joint pain associated with redness, warmth, or swelling AND the patient also has an oral temperature greater than 38.0 °C and does not have a previously established diagnosis of pseudogout or gout, THEN diagnostic aspiration of the painfully swollen, red joint should be performed that day. | | Process |  |

**Steel N, et al. (16)**

| **Characteristics** | | | |
| --- | --- | --- | --- |
| Year | 2004 | | |
| Country | UK | | |
| Target population | Elderly patients aged 65 years or older in primary and secondary care | | |
| Evidence synthesis | Adaption of earlier published QIs (23) for the use in UK healthcare system | | |
| Consensus method | Modified RAND/UCLA Appropriateness Method | | |
| **OA quality indicators (6)** | | **Dimension of quality** | **Ambulatory health intervention before TJR quantifiable in routine data from German SHIs** |
| IF a person aged 65 or older is treated for symptomatic osteoarthritis, THEN functional status and degree of pain should be assessed at least annually. | | Process | Physician visit for musculoskeletal disorders |
| IF an ambulatory person aged 65 or older has had a diagnosis of symptomatic osteoarthritis of the knee for longer than 3 months and has no contraindications to exercise and is physically and mentally able to exercise, THEN a directed or supervised strengthening or aerobic exercise programme should have been prescribed at least once. | | Process | Exercise therapy/  referral to PT |
| IF an ambulatory person aged 65 or older has a diagnosis of symptomatic osteoarthritis, THEN education regarding the natural history, treatment and selfmanagement of the disease should be offered at least once. | | Process |  |
| IF oral pharmacological therapy is initiated to treat osteoarthritis among people aged 65 or older, THEN paracetamol should be the first drug used, unless there is a contraindication to use. | | Process | Paracetamol (initial medication) |
| IF oral pharmacological therapy for osteoarthritis is changed from paracetamol to a different oral agent among people aged 65 or older, THEN the patient should have had a trial of maximum dose paracetamol (suitable for age/co-morbidities). | | Process | Paracetamol (initial medication) |
| IF a person aged 65 or older with severe symptomatic osteoarthritis of the knee or hip has failed to respond to non-pharmacological and pharmacological therapy, THEN the patient should be offered referral to an orthopaedic surgeon to be evaluated for total joint replacement within 6 months unless surgery is contraindicated. | | Process | Referral to orthopaedic surgeon if conservative therapy failed |

**Underwood M, et al. (17)**

| **Characteristics** | | | |
| --- | --- | --- | --- |
| Year | 2002 | | |
| Country | UK | | |
| Target population | OA patients in general practice | | |
| Evidence synthesis | Literature review, not specified to be systematic | | |
| Consensus method | Modified RAND/UCLA Appropriateness Method | | |
| **OA quality indicators (4)** | | **Dimension of quality** | **Ambulatory health intervention before TJR quantifiable in routine data from German SHIs** |
| Patients with a new diagnosis of osteoarthritis who wish to take medication for joint symptoms should be offered a trial of paracetamol if not already tried. | | Process | Paracetamol (initial medication) |
| If NSAIDs are considered, ibuprofen should be considered for first-line treatment unless contraindicated or intolerant. | | Process | Oral NSAID |
| Patients with osteoarthritis prescribed oral NSAIDs who are at high risk of gastrointestinal side-effects (past history of dyspepsia or known peptic ulcer) should be considered for a coprescription of PPIs, $H_{2}$ antagonists or misoprostol, unless contraindicated or intolerant. | | Process | Oral NSAID treatment with concomitant PPI/misoprostol in case of Gl risk factors |
| Patients with severe symptomatic osteoarthritis of the knee or hip who have failed to respond to conservative therapy should be offered referral to an orthopaedic surgeon for consideration of joint replacement. | | Process | Referral to orthopaedic surgeon if conservative therapy failed |

**Vandenberghe H, et al. (18)**

| **Characteristics** | | | |
| --- | --- | --- | --- |
| Year | 2004 | | |
| Country | Belgium | | |
| Target population | OA patients aged 60 years or older in general practice | | |
| Evidence synthesis | not specified | | |
| Consensus method | not specified | | |
| **OA quality indicators (5)** | | **Dimension of quality** | **Ambulatory health intervention before TJR quantifiable in routine data from German SHIs** |
| Patients with a drug prescription for osteoarthritis in the past month (numerator)/ all patients with osteoarthritis (denominator) | | Process |  |
| Patients who were prescribed paracetamol (numerator)/ all patients with a drug prescription for osteoarthritis in the past month (denominator) | | Process | Paracetamol (initial medication) |
| Patients which were prescribed an NSAID (numerator)/ all patients with a drug prescription for osteoarthritis in the past month (denominator) | | Process | Oral NSAID |
| Patients who were prescribed a coxib (numerator)/ all patients who received an NSAID for osteoarthritis in the past month (denominator) | | Process | Oral NSAID |
| Patients who received a repeated prescription/ all patients who received an NSAID for osteoarthritis in the past month (denominator) | | Process | Oral NSAID |

**Wierenga PC (19)**

| **Characteristics** | | | |
| --- | --- | --- | --- |
| Year | 2011 | | |
| Country | The Netherlands | | |
| Target population | Elderly hospitalised patients | | |
| Evidence synthesis | Adaption of earlier published QIs (23) for elderly hospitalised patients | | |
| Consensus method | Delphi-process with a multidisciplinary expert panel | | |
| **OA quality indicators (2)** | | **Dimension of quality** | **Ambulatory health intervention before TJR quantifiable in routine data from German SHIs** |
| IF oral pharmacological therapy is initiated to treat osteoarthritis in an elder, THEN paracetamol (acetaminophen) should be the first drug used, UNLESS there is a documented contra-indication. | | Process | Paracetamol (initial medication) |
| IF oral pharmacological therapy for osteoarthritis in an elder is changed from paracetamol (acetaminophen) to a different oral agent, THEN there should be evidence that the patient has had a trial of maximum dose of paracetamol (suitable for age and co-morbid conditions). | | Process | Paracetamol (initial medication) |

**Zingmond DS (20)**

| **Characteristics** | | | |
| --- | --- | --- | --- |
| Year | 2009 | | |
| Country | US | | |
| Target population | Community dwellers in nursing homes aged 65 years and older | | |
| Evidence synthesis | Adaption of earlier published QIs (14) for use with routinely collected data | | |
| Consensus method | not specified | | |
| **OA quality indicators (9)** | | **Dimension of quality** | **Ambulatory health intervention before TJR quantifiable in routine data from German SHIs** |
| IF a NH Resident has a new joint pain that is reported to the primary care provider, THEN the joint and peri-articular structures should be examined within 1 month or there should be documentation that the problem has resolved. | | Process | Physician visit for musculoskeletal disorders |
| IF a non-OTC drug is newly prescribed to treat joint pain, THEN evidence that the affected joint was examined should be documented within 4 weeks. | | Process | Physician visit for musculoskeletal disorders |
| IF a NH Resident has monoarticular joint pain associated with redness, warmth and/or swelling and the patient also has an oral temperature >38°C, and does not have a previously established diagnosis of pseudogout or gout, THEN a diagnostic aspiration of the painfully swollen red joint should be performed that day. | | Process |  |
| IF an ambulatory NH Resident is newly diagnosed with symptomatic osteoarthritis of the knee and has no contraindication to exercise and is physically and mentally able to exercise, THEN a directed or supervised strengthening or aerobic exercise program should be prescribed within 1 month of diagnosis. | | Process | Exercise therapy/  referral to PT |
| IF an ambulatory NH Resident has had a diagnosis of symptomatic osteoarthritis of the knee for >12 months and has no contraindication to exercise and is physically and mentally able to exercise, THEN there should be evidence that a directed or supervised strengthening or aerobic exercise program was prescribed at least once since the time of diagnosis. | | Process | Exercise therapy/  referral to PT |
| IF oral pharmacologic therapy is initiated to treat symptomatic osteoarthritis, THEN acetaminophen should be the first drug used. | | Process | Paracetamol (initial medication) |
| IF oral pharmacologic therapy for symptomatic osteoarthritis is changed from acetaminophen to a different oral agent, THEN there should be evidence that the NH Resident has had a trial of maximum dose acetaminophen (suitable for age/comorbidities). | | Process | Paracetamol (initial medication) |
| IF a NH Resident is over age 75 or has any of the following: history of peptic ulcer disease, history of gastrointestinal bleeding, OR current warfarin use; AND the resident is being treated with a non-COX-2 inhibitor NSAID, THEN s/he should be offered treatment with misoprostol or a proton pump inhibitor. | | Process | Oral NSAID treatment with concomitant PPI/misoprostol in case of Gl risk factors |
| IF a NH Resident is treated with nonsteroidal anti-inflammatory drugs (NSAIDs), THEN there should be evidence that the risks associated with these drugs were described to the resident, if the resident is capable of understanding. | | Process |  |

**REFERENCES**

1. Asch SM, McGlynn EA, Hogan MM, Rodney Hayward RA, Shekelle P et al (2004) Comparison of Quality of Care for Patients in the Veterans Health Administration and Patients in a National Sample. Ann Intern Med 141(12):938-945. <https://doi.org/10.7326/0003-4819-141-12-200412210-00010>

2. Barber CE, Patel JN, Woodhouse L, Smith C, Weiss S, Homik J et al (2015) Development of key performance indicators to evaluate centralized intake for patients with osteoarthritis and rheumatoid arthritis. Arthritis Res Ther 17(322). <https://doi.org/10.1186/s13075-015-0843-7>

3. Doubova SV, Perez-Cuevas R (2015) Quality of care for hip and knee osteoarthritis at family medicine clinics: lessons from Mexico. IJQHC 27(2):125-131. <https://doi.org/10.1093/intqhc/mzv003>

4. EUMUSC.net (2012) Health care quality indicators for OA. <https://www.eumusc.net/myUploadData/files/EUMUSC%20Health%20care%20quality%20indicators%20for%20OA%20KE.pdf>

5. Grypdonck L, Aertgeerts B, Luyten F, Wollersheim H, Bellemans J, Peers K, Verschueren S (2014) Development of Quality Indicators for an Integrated Approach of Knee Osteoarthritis. J Rheumatol 41(6):1155-1162. <https://doi.org/10.3899/jrheum.130680>

6. Jansen MJ, Hendriks EJ, Oostendorp RAB, Dekker J, de Bie RA (2010) Quality indicators indicate good adherence to the clinical practice guideline on “Osteoarthritis of the hip and knee” and few prognostic factors influence outcome indicators: a prospective cohort study. Eur J Phys Rehabil Med 46(3):337-345. <https://www.minervamedica.it/en/journals/europa-medicophysica/article.php?cod=R33Y2010N03A0337>

7. Kleudgen S, Diel F, Burgdorf F, Quasdorf I (2009) KBV entwickelt Starter-Set ambulanter Qualitätsindikatoren - Ergebnisse des Projektes „AQUIK – Ambulante Qualitätsindikatoren und Kennzahlen”.

8. MacLean CH, Pencharz JN, Saag KG (2007) Quality indicators for the care of osteoarthritis in vulnerable elders. JAGS 55 Suppl 2:S383-91. <https://doi.org/10.1111/j.1532-5415.2007.01346.x>

9. McGlynn EA, Asch SM, Adams J, Keesey J, Hicks J, DeCristofaro A et al (2003) The quality of health care delivered to adults in the United States. N Eng J Med 348(26):2635-2645. <https://doi.org/10.1056/NEJMsa022615>

10. Moore A (2000) Quality of Care for General Medical Conditions: Chapter 17. Osteoarthritis treatment. In: Kerr EA, Asch SM, Hamilton EG, McGlynn EA (ed) Quality of Care for General Medical Conditions: A Review of the Literature and Quality Indicators. Santa Monica, CA: RAND Corporation, 247-262.

11. Østerås N, Tveter AT, Garratt AM, Svinøy OE, Kjeken I, Natvig B, Grotle M, Hagen KB (2018) Measurement properties for the revised patient-reported OsteoArthritis Quality Indicator questionnaire. Osteoarthr Cartil 26(10):1300-1310. <https://doi.org/10.1016/j.joca.2018.06.007>

12. American Academy of Orthopaedic Surgeons (AAOS), Physician Consortium for Performance Improvement (PCPI) (2006) Osteoarthritis: Physician Performance Measurement Set.

13. Peter WF, van der Wees PJ, Hendriks EJ, de Bie RA, Verhoef J, de Jong Z et al (2013) Quality indicators for physiotherapy care in hip and knee osteoarthritis: development and clinimetric properties. Musculoskelet Care 11(4):193-202. <https://doi.org/10.1002/msc.1041>

14. Saliba D, Solomon D, Rubenstein L, Young R, Schnelle J, Roth C et al (2004) Quality indicators for the management of medical conditions in nursing home residents. JAMDA 5(5):297-309. <https://doi.org/10.1097/01.JAM.0000136960.25327.61>

15. Smith KL, Soriano TA, Boal J (2007) Brief communication: National quality-of-care standards in home-based primary care. Ann Intern Med 146(3):188-192. <https://doi.org/10.7326/0003-4819-146-3-200702060-00008>

16. Steel N, Melzer D, Shekelle PG, Wenger NS, Forsyth D, McWilliams BC (2004) Developing quality indicators for older adults: transfer from the USA to the UK is feasible. Qual Saf Health Care 13(4):260-264. <https://doi.org/10.1136/qhc.13.4.260>

17. Underwood M (2002) Osteoarthritis. In: Marshall M, Campbell S, Hacker J, Roland M (ed) Quality indicators for general practice. A practical guide to clinical quality indicators for primary care health professionals and managers. Manchester: National Primary Care Research and Development Centre University of Manchester, 56–66.

18. Vandenberghe H, Van Casteren V, Jonckheer P, Lafontaine MF, De Clercq E (2004) Quality of care assessment using GPs’ electronic patient records: do we need data from home visits? Stud Health Technol Inform 110:35-41. <https://ebooks.iospress.nl/publication/10006>

19. Wierenga PC, Klopotowska JE, Smorenburg SM, van Kan HJ, Bijleveld YA, Dijkgraaf MG (2011) Quality indicators for in-hospital pharmaceutical care of Dutch elderly patients: development and validation of an ACOVE-based quality indicator set. Drugs Aging 28(4):295-304. <https://doi.org/10.2165/11587700-000000000-00000>

20. Zingmond DS, Saliba D, Wilber KH, MacLean CH, Wenger NS (2009) Measuring the quality of care provided to dually enrolled Medicare and Medicaid beneficiaries living in nursing homes. Med Care 47(5):536-544. <https://doi.org/10.1097/MLR.0b013e318190cd8b>

21. Vogels EMHM, Hendriks HJM, Baar ME, Dekker J, Hopman-Rock M, Oostendorp RAB (2001) KNGF-guidelines for physical therapy in patients with osteoarthritis of the hip or knee. Nederlands Tijdschrift voor Fysiotherapie 111(3 suppl):1-34. <https://www.ifompt.org/site/ifompt/files/pdf/Osteoarthr.H-K.Gln.pdf>

22. Peter WF, Jansen MJ, Hurkmans EJ, Bloo H, Dekker-Bakker LMMC, Dilling R et al (2011) Physiotherapy in hip and knee osteoarthritis: Development of a practice guideline concerning initial assessment, treatment and evaluation. Acta Reumatol Port 36:268-281. <https://www.arprheumatology.com/oldsite/conteudo/pdfs/12_-_PC_-_Physiotherapy_ARP2010.pdf>

23. MacLean CH (2001) Quality indicators for the management of osteoarthritis in vulnerable elders. Ann Intern Med 135(8 Pt 2):711-721. <https://doi.org/10.7326/0003-4819-135-8_part_2-200110161-00010>
