# Supplementary material for: Routine‐data‐compatible quality indicators for the ambulatory care of osteoarthritis of the knee and hip: A systematic review
Source: Knee Surg Sports Traumatol Arthrosc. 2025 Feb 13;33(10):3523–41. doi: 10.1002/ksa.12614 (PMC12459328; doi:10.1002/ksa.12614)
Supplement: Supplementary file 3 — Supporting information. [file KSA-33-3523-s002.docx]

**APPENDIX 3.**

Quality appraisal of QI sets and guidelines.

**Table 1.** Appraisal of Indicators through Research and Evaluation (AIRE) Instrument Score.

**Table 2.** Appraisal of Guidelines for Research and Evaluation (AGREE) Instrument Score.

| **Table 1.** Appraisal of Indicators through Research and Evaluation (AIRE) Instrument Score. | | | | |
| --- | --- | --- | --- | --- |
|  | **AIRE instrument^1^ domain score (%)** | | | |
| **first author/institution** | **domain 1:  purpose, relevance and organisational context** | **domain 2:  stakeholder involvement** | **domain 3:  scientific evidence** | **domain 4:  additional evidence, formulation and usage** |
| Østerås N (1) | 57 | 44 | 61 | 57 |
| Barber CE (2) | 80 | 94 | 72 | 57 |
| Doubova SV (3) | 77 | 67 | 78 | 65 |
| Grypdonck L (4) | 80 | 83 | 78 | 65 |
| Peter WF (5) | 87 | 67 | 72 | 57 |
| EUMUSC.net (6) | 80 | 78 | 83 | 70 |
| Wierenga PC (7) | 87 | 61 | 56 | 67 |
| Jansen MJ (8) | 70 | 50 | 56 | 48 |
| Kleudgen S (9) | 87 | 89 | 89 | 85 |
| Zingmond DS (10) | 80 | 33 | 44 | 65 |
| MacLean CH (11) | 83 | 78 | 94 | 81 |
| Smith KL (12) | 87 | 83 | 67 | 70 |
| PCPI (13) | 87 | 83 | 72 | 87 |
| Asch SM (14) | 80 | 72 | 56 | 72 |
| Saliba D (15) | 80 | 78 | 50 | 67 |
| Steel N (16) | 77 | 72 | 61 | 67 |
| Vandenberghe H (17) | 50 | 17 | 33 | 65 |
| McGlynn EA (18) | 83 | 67 | 72 | 74 |
| Underwood M (19) | 43 | 61 | 61 | 48 |
| Moore A (20) | 43 | 44 | 50 | 46 |
| Legends: AIRE = Appraisal of Indicators through Research and Evaluation.  ^1^ Items of the domains were scored by two raters independently (1 = “strongly disagree” (criterion was not met or no information was provided), 2–3 = “agree/ disagree” (not sure if the criterion was met), 4 = “strongly agree”) and summed per domain. If the scores between raters varied by more than one, variability has been discussed and disagreements were resolved through consensus. The formula for the calculation of the domain score is: (total score - minimum possible score) / (maximum possible score - minimum possible score) × 100%. A higher score indicates a higher methodological level (range 0–100%). QI sets with a domain score of 50% or higher (overall “agree” or “strongly agree”) were considered to have a high methodological quality in the relevant domain. The domain scores are independent and should not be aggregated into a single overall quality score. | | | | |

| **Table 2.** Appraisal of Guidelines for Research and Evaluation (AGREE) Instrument Score. | | | | |  | |  | |  | |
| --- | --- | --- | --- | --- | --- | --- | --- | --- | --- | --- |
|  | **AGREE instrument^1^ domain score (%)** | | | | | | | | | |
| **first author/institution** | **domain 1:  scope and purpose** | **domain 2:  stakeholder involvement** | **domain 3:  rigour of development** | **domain 4:  clarity of presentation** | | **domain 5: applicability** | | **domain 6: editorial independence** | | **overall assessment** |
| AWMF (21) | 86 | 86 | 72 | 86 | | 50 | | 79 | | A |
| AAOS (22) | 92 | 89 | 86 | 75 | | 60 | | 79 | | A |
| MQIC (23) | 33 | 33 | 18 | 53 | | 13 | | 21 | | C |
| Yeap SS (24) | 92 | 78 | 69 | 58 | | 44 | | 50 | | B |
| ACR (25) | 92 | 92 | 82 | 81 | | 65 | | 79 | | A |
| DVA (26) | 86 | 94 | 86 | 81 | | 69 | | 79 | | A |
| KNGF (27) | 75 | 58 | 75 | 72 | | 69 | | 67 | | A |
| NICE (28) | 83 | 78 | 79 | 89 | | 58 | | 67 | | A |
| SFR (29) | 67 | 69 | 53 | 64 | | 38 | | 38 | | B |
| Zhang Z (30) | 81 | 81 | 71 | 64 | | 38 | | 63 | | A |
| AWMF (31) | 69 | 53 | 30 | 56 | | 15 | | 21 | | B |
| ESCEO (32) | 75 | 72 | 57 | 67 | | 44 | | 50 | | B |
| Jarl G (33) | 61 | 50 | 47 | 39 | | 27 | | 63 | | B |
| OARSI (34) | 75 | 58 | 64 | 67 | | 33 | | 83 | | A |
| SIR (35) | 81 | 81 | 71 | 72 | | 50 | | 83 | | A |
| Yabuki S (36) | 47 | 44 | 41 | 56 | | 33 | | 54 | | B |
| AWMF (37) | 58 | 53 | 30 | 56 | | 21 | | 21 | | B |
| EULAR (38) | 75 | 75 | 68 | 67 | | 35 | | 46 | | A |
| EULAR (39) | 75 | 81 | 71 | 67 | | 48 | | 50 | | A |
| NHMRC (40) | 92 | 81 | 72 | 86 | | 50 | | 75 | | A |
| TLAR (41) | 69 | 47 | 54 | 53 | | 29 | | 67 | | B |
| AAOS (42) | 89 | 75 | 82 | 75 | | 63 | | 79 | | A |
| APTA (43) | 94 | 78 | 81 | 72 | | 71 | | 67 | | A |
| EULAR (44) | 81 | 78 | 67 | 69 | | 46 | | 71 | | A |
| Ottawa Panel (45) | 86 | 67 | 60 | 44 | | 38 | | 71 | | B |
| Ottawa Panel (46) | 81 | 58 | 59 | 69 | | 33 | | 75 | | B |
| Ottawa Panel (47) | 78 | 56 | 60 | 36 | | 31 | | 71 | | B |
| PANLAR (48) | 67 | 64 | 46 | 56 | | 33 | | 79 | | B |
| EULAR (49) | 83 | 78 | 69 | 75 | | 38 | | 79 | | A |
| NHG (50) | 25 | 25 | 28 | 31 | | 23 | | 21 | | C |
| EULAR (51) | 64 | 47 | 59 | 72 | | 42 | | 33 | | B |
| Ottawa Panel (52) | 72 | 64 | 65 | 44 | | 40 | | 33 | | B |
| MOVE consensus (53) | 64 | 58 | 49 | 39 | | 29 | | 54 | | B |
| EULAR (54) | 53 | 33 | 57 | 53 | | 48 | | 29 | | B |
| Philadelphia Panel (55) | 69 | 64 | 55 | 39 | | 42 | | 25 | | B |
| Legends: AAOS = American Academy of Orthopaedic Surgeons; ACR = American College of Rheumatology; AGREE = Appraisal of Guidelines for Research and Evaluation; APTA = American Physical Therapy Association; AWMF = Association of the Scientific Medical Societies in Germany; cLBP = chronic low back pain; DVA = Department of Veterans Affairs; ESCEO = European Society for Clinical and Economic Aspects of Osteoporosis, Osteoarthritis and Musculoskeletal Diseases; EULAR = European League Against Rheumatism; IA = inflammatory arthritis; KNGF = The Royal Dutch Society for Physiotherapy; MQIC = Michigan Quality Improvement Consortium Guideline; NHG = Dutch College of General Practitioners; NHMRC = National Health and Medical Research Council; NICE = National Institute for Health and Care Excellence; OARSI = Osteoarthritis Research Society International; PANLAR = Panamerican League of Associations for Rheumatology; SFR = French Society of Rheumatology; SIR = Italian Society for Rheumatology.  ^1^ Items of the domains were scored by two raters independently (1 = “strongly disagree”, 2-6 = the item does not meet all criteria, but is scored higher the more considerations are adressed; 7 = “strongly agree”) and summed per domain. If the scores between raters varied by ≥3, variability has been discussed and disagreements were resolved through consensus. The formula for the calculation of the domain score is: (total score - minimum possible score) / (maximum possible score - minimum possible score) × 100%. A higher score indicates a higher methodological level (range 0–100%). The domain scores are independent and should not be aggregated into a single quality score. The overall assessment for a guideline, making use of the classification system of a previous quality appraisal (56), classifies guidelines into three levels (Level A: high quality, recommended (≥4 domains were scored >60%, including the domain ‘rigour of development’); Level B: medium quality, recommended with modification (≥4 domains were scored ≥30%); Level C: low quality, not recommended (≥3 of domains were scored <30%)). | | | | | | | | | | |

**REFRENCES**

1. Østerås N, Tveter AT, Garratt AM, Svinøy OE, Kjeken I, Natvig B, Grotle M, Hagen KB (2018) Measurement properties for the revised patient-reported OsteoArthritis Quality Indicator questionnaire. Osteoarthr Cartil 26(10):1300-1310. <https://doi.org/10.1016/j.joca.2018.06.007>

2. Barber CE, Patel JN, Woodhouse L, Smith C, Weiss S, Homik J et al (2015) Development of key performance indicators to evaluate centralized intake for patients with osteoarthritis and rheumatoid arthritis. Arthritis Res Ther 17(322). <https://doi.org/10.1186/s13075-015-0843-7>

3. Doubova SV, Perez-Cuevas R (2015) Quality of care for hip and knee osteoarthritis at family medicine clinics: lessons from Mexico. IJQHC 27(2):125-131. <https://doi.org/10.1093/intqhc/mzv003>

4. Grypdonck L, Aertgeerts B, Luyten F, Wollersheim H, Bellemans J, Peers K, Verschueren S (2014) Development of Quality Indicators for an Integrated Approach of Knee Osteoarthritis. J Rheumatol 41(6):1155-1162. <https://doi.org/10.3899/jrheum.130680>

5. Peter WF, van der Wees PJ, Hendriks EJ, de Bie RA, Verhoef J, de Jong Z et al (2013) Quality indicators for physiotherapy care in hip and knee osteoarthritis: development and clinimetric properties. Musculoskelet Care 11(4):193-202. <https://doi.org/10.1002/msc.1041>

6. EUMUSC.net (2012) Health care quality indicators for OA. <https://www.eumusc.net/myUploadData/files/EUMUSC%20Health%20care%20quality%20indicators%20for%20OA%20KE.pdf>

7. Wierenga PC, Klopotowska JE, Smorenburg SM, van Kan HJ, Bijleveld YA, Dijkgraaf MG (2011) Quality indicators for in-hospital pharmaceutical care of Dutch elderly patients: development and validation of an ACOVE-based quality indicator set. Drugs Aging 28(4):295-304. <https://doi.org/10.2165/11587700-000000000-00000>

8. Jansen MJ, Hendriks EJ, Oostendorp RAB, Dekker J, de Bie RA (2010) Quality indicators indicate good adherence to the clinical practice guideline on “Osteoarthritis of the hip and knee” and few prognostic factors influence outcome indicators: a prospective cohort study. Eur J Phys Rehabil Med 46(3):337-345. <https://www.minervamedica.it/en/journals/europa-medicophysica/article.php?cod=R33Y2010N03A0337>

9. Kleudgen S, Diel F, Burgdorf F, Quasdorf I (2009) KBV entwickelt Starter-Set ambulanter Qualitätsindikatoren - Ergebnisse des Projektes „AQUIK – Ambulante Qualitätsindikatoren und Kennzahlen”.

10. Zingmond DS, Saliba D, Wilber KH, MacLean CH, Wenger NS (2009) Measuring the quality of care provided to dually enrolled Medicare and Medicaid beneficiaries living in nursing homes. Med Care 47(5):536-544. <https://doi.org/10.1097/MLR.0b013e318190cd8b>

11. MacLean CH, Pencharz JN, Saag KG (2007) Quality indicators for the care of osteoarthritis in vulnerable elders. JAGS 55 Suppl 2:S383-91. <https://doi.org/10.1111/j.1532-5415.2007.01346.x>

12. Smith KL, Soriano TA, Boal J (2007) Brief communication: National quality-of-care standards in home-based primary care. Ann Intern Med 146(3):188-192. <https://doi.org/10.7326/0003-4819-146-3-200702060-00008>

13. American Academy of Orthopaedic Surgeons (AAOS), Physician Consortium for Performance Improvement (PCPI) (2006) Osteoarthritis: Physician Performance Measurement Set.

14. Asch SM, McGlynn EA, Hogan MM, Rodney Hayward RA, Shekelle P et al (2004) Comparison of Quality of Care for Patients in the Veterans Health Administration and Patients in a National Sample. Ann Intern Med 141(12):938-945. <https://doi.org/10.7326/0003-4819-141-12-200412210-00010>

15. Saliba D, Solomon D, Rubenstein L, Young R, Schnelle J, Roth C et al (2004) Quality indicators for the management of medical conditions in nursing home residents. JAMDA 5(5):297-309. <https://doi.org/10.1097/01.JAM.0000136960.25327.61>

16. Steel N, Melzer D, Shekelle PG, Wenger NS, Forsyth D, McWilliams BC (2004) Developing quality indicators for older adults: transfer from the USA to the UK is feasible. Qual Saf Health Care 13(4):260-264. <https://doi.org/10.1136/qhc.13.4.260>

17. Vandenberghe H, Van Casteren V, Jonckheer P, Lafontaine MF, De Clercq E (2004) Quality of care assessment using GPs’ electronic patient records: do we need data from home visits? Stud Health Technol Inform 110:35-41. <https://ebooks.iospress.nl/publication/10006>

18. McGlynn EA, Asch SM, Adams J, Keesey J, Hicks J, DeCristofaro A et al (2003) The quality of health care delivered to adults in the United States. N Eng J Med 348(26):2635-2645. <https://doi.org/10.1056/NEJMsa022615>

19. Underwood M (2002) Osteoarthritis. In: Marshall M, Campbell S, Hacker J, Roland M (ed) Quality indicators for general practice. A practical guide to clinical quality indicators for primary care health professionals and managers. Manchester: National Primary Care Research and Development Centre University of Manchester, 56–66.

20. Moore A (2000) Quality of Care for General Medical Conditions: Chapter 17. Osteoarthritis treatment. In: Kerr EA, Asch SM, Hamilton EG, McGlynn EA (ed) Quality of Care for General Medical Conditions: A Review of the Literature and Quality Indicators. Santa Monica, CA: RAND Corporation, 247-262.

21. Deutsche Gesellschaft für Orthopädie und Unfallchirurgie e.V. (DGOU) (2021) Evidenz- und konsensbasierte Indikationskriterien zur Hüfttotalendoprothese bei Coxarthrose (EKIT-Hüfte). S3-Leitlinie der Deutschen Gesellschaft für Orthopädie und Unfallchirurgie e.V. (DGOU). <https://register.awmf.org/assets/guidelines/187-001l_S3_Indikationskriterien_H%C3%BCfttotalendoprothese_bei_Coxarthrose_2021-04.pdf>

22. American Academy of Orthopaedic Surgeons (AAOS) (2021) Management of Osteoarthritis of the Knee (Non-Arthroplasty): Evidence-Based Clinical Practice Guideline. <https://www.aaos.org/globalassets/quality-and-practice-resources/osteoarthritis-of-the-knee/oak3cpg.pdf>

23. Michigan Quality Improvement Consortium Guideline (MQIC) (2021) Medical Management of Adults with Osteoarthritis. <https://www.mahp.org/wp-content/uploads/2024/03/mqicmedicalmanagementofadultswithosteoarthritisFinal2024.pdf>

24. Yeap SS, Amin SRA, Baharuddin H, Koh KC, Lee JK, Lee VKM et al (2021) A Malaysian Delphi consensus on managing knee osteoarthritis. BMC Musculoskelet Disord 22:514. <https://doi.org/10.1186/s12891-021-04381-8>

25. Kolasinski SL, Tuhina N, Hochberg MC, Oatis C, Guyatt G, Block J et al (2020) 2019 American College of Rheumatology/Arthritis Foundation Guideline for the Management of Osteoarthritis of the Hand, Hip, and Knee. Arthritis Care Res 72(2):149-162. <https://doi.org/10.1002/acr.24131>

26. Department of Veterans Affairs (DVA) (2020) VA/DoD Clinical Practice Guideline for the Non-Surgical Management of Hip & Knee Osteoarthritis. <https://www.healthquality.va.gov/guidelines/cd/oa/index.asp>

27. van Doormaal MCM, Meerhoff GA, Vliet Vlieland TPM, Peter WF (2020) A clinical practice guideline for physical therapy in patients with hip or knee osteoarthritis. Musculoskelet Care 18(4):575-595. <https://doi.org/10.1002/msc.1492>

28. National Institute for Health and Care Excellence (NICE) (2020) Osteoarthritis: care and management. <https://www.nice.org.uk/guidance/cg177>

29. Sellam J, Courties A, Eymard F, Ferrero S, Latourte A, Ornetti P et al (2020) Recommendations of the French Society of Rheumatology on pharmacological treatment of knee osteoarthritis. Jt Bone Spine 87(6):548-555. <https://doi.org/10.1016/j.jbspin.2020.09.004>

30. Zhang Z, Huang C, Jiang Q, Zheng Y, Liu Y, Liu S et al (2020) Guidelines for the diagnosis and treatment of osteoarthritis in China (2019 edition). Ann Transl Med 8(19):1213. <https://doi.org/10.21037/atm-20-4665>

31. Deutsche Gesellschaft für Orthopädie und Unfallchirurgie e.V. (DGOU) (2019) S2k-Leitlinie Koxarthrose.

32. Bruyère O, Honvo G, Veronese N, Arden NK, Branco J, Curtis EM et al (2019) An updated algorithm recommendation for the management of knee osteoarthritis from the European Society for Clinical and Economic Aspects of Osteoporosis, Osteoarthritis and Musculoskeletal Diseases (ESCEO). Semin Arthritis Rheum 49(3):337-350. <https://doi.org/10.1016/j.semarthrit.2019.04.008>

33. Jarl G, Hellstrand Tang U, Nordén E, Johannesson A, Rusaw DF (2019) Nordic clinical guidelines for orthotic treatment of osteoarthritis of the knee: A systematic review using the AGREE II instrument. Prosthet Orthot Int 43(5):556-563. <https://doi.org/10.1177/0309364619857854>

34. Bannuru RR, Osani MC, Vaysbrot EE, Arden NK, Bennell K, Bierma-Zeinstra SMA et al (2019) OARSI guidelines for the non-surgical management of knee, hip, and polyarticular osteoarthritis. Osteoarthr Cartil 27(11):1578-1589. <https://doi.org/10.1016/j.joca.2019.06.011>

35. Ariani A, Manara M, Fioravanti A, Iannone F, Salaffi F, Ughi N, Prevete I et al (2019) The Italian Society for Rheumatology clinical practice guidelines for the diagnosis and management of knee, hip and hand osteoarthritis. Reumatismo 71(S1):5-21. <https://doi.org/10.4081/reumatismo.2019.1188>

36. Yabuki S, Ip AKK, Tam CK, Murakami T, Ushida T, Wang JH et al (2019) Evidence-Based Recommendations on the Pharmacological Management of Osteoarthritis and Chronic Low Back Pain: An Asian Consensus. AJA 57(2):37-54. <https://doi.org/10.6859/aja.201906_57(2).0003>

37. Deutsche Gesellschaft für Orthopädie und Unfallchirurgie e.V. (DGOU) (2018) S2k-Leitlinie Gonarthrose.

38. Geenen R, Overman CL, Christensen R, Åsenlöf P, Capela S, Huisinga KL et al (2018) EULAR recommendations for the health professional’s approach to pain management in inflammatory arthritis and osteoarthritis. Ann Rheum Dis 77(6):797-807. <https://doi.org/10.1136/annrheumdis-2017-212662>

39. Rausch Osthoff AK, Niedermann K, Braun J, Adams J, Brodin N, Dagfinrud H et al (2018) 2018 EULAR recommendations for physical activity in people with inflammatory arthritis and osteoarthritis. Ann Rheum Dis 77(9):1251-1260. <https://doi.org/10.1136/annrheumdis-2018-213585>

40. National Health and Medical Research Council (NHMRC) (2018) Guideline for the management of knee and hip osteoarthritis. Second edition. <https://www.racgp.org.au/getattachment/71ab5b77-afdf-4b01-90c3-04f61a910be6/Guideline-for-the-management-of-knee-and-hip-osteoarthritis.aspx>

41. Tuncer T, Cay FH, Altan L, Gurer G, Kacar C, Ozcakir S et al (2018) 2017 update of the Turkish League Against Rheumatism (TLAR) evidence-based recommendations for the management of knee osteoarthritis. Rheumatol Int 38(8):1315-1331. <https://doi.org/10.1007/s00296-018-4044-y>

42. American Academy of Orthopaedic Surgeons (AAOS) (2017) Management of Osteoarthritis of the Hip. Evidence-based Clinical Practice Guideline. <https://www.aaos.org/globalassets/quality-and-practice-resources/osteoarthritis-of-the-hip/oa-hip-cpg_6-11-19.pdf>

43. Cibulka MT, Bloom NJ, Enseki KR, Macdonald CW, Woehrle J, McDonough CM (2017) Hip Pain and Mobility Deficits – Hip Osteoarthritis: Revision 2017. J Orthop Sports Phys Ther 47(6):A1-A37. <https://doi.org/10.2519/jospt.2017.0301>

44. Sakellariou G, Conaghan PG, Zhang W, Bijlsma JWJ, Boyesen P, D’Agostino MA et al (2017) EULAR recommendations for the use of imaging in the clinical management of peripheral joint osteoarthritis. Ann Rheum Dis 76(9):1484-1494. <https://doi.org/10.1136/annrheumdis-2016-210815>

45. Brosseau L, Taki J, Desjardins B, Thevenot O, Fransen M, Wells GA et al (2017) The Ottawa panel clinical practice guidelines for the management of knee osteoarthritis. Part two: strengthening exercise programs. Clin Rehabil 31(5):596-611. <https://doi.org/10.1177/0269215517691084>

46. Brosseau L, Taki J, Desjardins B, Thevenot O, Fransen M, Wells GA et al (2017) The Ottawa panel clinical practice guidelines for the management of knee osteoarthritis. Part three: aerobic exercise programs. Clin Rehabil 31(5):612-624. <https://doi.org/10.1177/0269215517691085>

47. Brosseau L, Wells GA, Pugh AG, Smith CA, Rahman P, Àlvarez Gallardo IC et al (2016) Ottawa Panel evidence-based clinical practice guidelines for therapeutic exercise in the management of hip osteoarthritis. Clin Rehabil 30(10):935-946. <https://doi.org/10.1177/0269215515606198>

48. Rillo O, Riera H, Acosta C, Liendo V, Bolaños J, Monterola L et al (2016) PANLAR Consensus Recommendations for the Management in Osteoarthritis of Hand, Hip, and Knee. J Clin Rheumatol 22(7):345-354. <https://doi.org/10.1097/RHU.0000000000000449>

49. Fernandes L, Hagen KB, Bijlsma JW, Andreassen O, Christensen P, Conaghan PG et al (2013) EULAR recommendations for the non-pharmacological core management of hip and knee osteoarthritis. Ann Rheum Dis 72(7):1125-1135. <https://doi.org/10.1136/annrheumdis-2012-202745>

50. Belo JN, Bierma-Zeinstra SMA, Raaijmakers AJ, van der Wissel F, Opstelten W (2008) Nontraumatic Knee Complaints in Adults in General Practice. Huisarts en Wetenschap 51(5):229-240. <https://repub.eur.nl/pub/17454/090701__Belo,%20Jannetje%20Neeltje.pdf>

51. Zhang W, Doherty M, Arden N, Bannwarth B, Bijlsma J, Gunther KP et al (2005) EULAR evidence based recommendations for the management of hip osteoarthritis: report of a task force of the EULAR Standing Committee for International Clinical Studies Including Therapeutics (ESCISIT). Ann Rheum Dis 64(5):669-681. <https://doi.org/10.1136/ard.2004.028886>

52. Brosseau L, Wells GA, Tugwell P, Egan M, Dubouloz CJ, Casimiro L et al (2005) Ottawa panel evidence-based clinical practice guidelines for therapeutic exercises and manual therapy in the management of osteoarthritis. Phys Ther 85(9):907-971. <https://doi.org/10.1093/ptj/85.9.907>

53. Roddy E, Zhang W, Doherty M, Arden NK, Barlow J, Birrell F et al (2005) Evidence-based recommendations for the role of exercise in the management of osteoarthritis of the hip or knee – the MOVE consensus. Rheumatology (Oxford) 44(1):67-73. <https://doi.org/10.1093/rheumatology/keh399>

54. Jordan KM, Arden NK, Doherty M, Bannwarth B, Bijlsma JW, Dieppe P et al (2003) EULAR Recommendations 2003: an evidence based approach to the management of knee osteoarthritis: Report of a Task Force of the Standing Committee for International Clinical Studies Including Therapeutic Trials (ESCISIT). Ann Rheum Dis 62(12):1145-1155. <https://doi.org/10.1136/ard.2003.011742>

55. Philadelphia Panel (2001) Philadelphia Panel evidence-based clinical practice guidelines on selected rehabilitation interventions for knee pain. Phys Ther 81(10):1675-1700. <https://doi.org/10.1093/ptj/81.10.1675>

56. Kim JY, Kim JH, Goo BH, Park YC, Seo BK, Baek YH (2021) Quality assessment of conventional and traditional oriental medicine clinical practice guidelines for knee osteoarthritis using AGREE II instrument. Medicine 100(51):e28426. <https://doi.org/10.1097/MD.0000000000028426>
