# Supplementary material for: Routine‐data‐compatible quality indicators for the ambulatory care of osteoarthritis of the knee and hip: A systematic review
Source: Knee Surg Sports Traumatol Arthrosc. 2025 Feb 13;33(10):3523–41. doi: 10.1002/ksa.12614 (PMC12459328; doi:10.1002/ksa.12614)
Supplement: Supplementary file 4 — Supporting information. [file KSA-33-3523-s001.docx]

**APPENDIX 4.**

Data extraction: guidelines.

**AAOS (1)**

| **Characteristics** | | |
| --- | --- | --- |
| Year | 2021 | |
| Country | US | |
| Target population | KOA patients | |
| LOE Scale | - Overall Strength of Evidence: Strong or Moderate   - Description of Evidence Quality: Evidence from two or more “High” quality studies with consistent findings for recommending for or against the intervention. Or Rec is upgrade from Moderate using the EtD framework - Overall Strength of Evidence: Strong, Moderate or Limited   - Description of Evidence Quality: Evidence from two or more “Moderate” quality studies with consistent findings, or evidence from a single “High” quality study for recommending for or against the intervention. Or Rec is upgraded or downgraded from Limited or Strong using the EtD framework. - Overall Strength of Evidence: Limited or Moderate   - Description of Evidence Quality: Evidence from one or more “Low” quality studies with consistent findings or evidence from a single “Moderate” quality study recommending for or against the intervention. Or Rec is downgraded from Moderate using the EtD Framework. - Overall Strength of Evidence: No evidence   - Description of Evidence Quality: There is no supporting evidence, or higher quality evidence was downgraded due to major concerns addressed in the EtD framework. In the absence of reliable evidence, the guideline work group is making a recommendation based on their clinical opinion. | |
| SOR Scale | - Strength of recommendation: Strong   - Overall Strength of Evidence: Strong or Moderate - Strength of recommendation: Moderate   - Overall Strength of Evidence: Strong, Moderate or Limited - Strength of recommendation: Limited   - Overall Strength of Evidence: Limited or Moderate - Strength of recommendation: Consensus   - Overall Strength of Evidence: No evidence | |
| **Recommendations (29)** | | **Ambulatory health intervention before TJR quantifiable in routine data from German SHIs** |
| Lateral wedge insoles are not recommended for patients with knee osteoarthritis.   - Strength of Recommendation: Strong | | Shoes/insoles |
| Canes could be used to improve pain and function in patients with knee osteoarthritis.   - Strength of Recommendation: Moderate | | Walking aids |
| Brace treatment could be used to improve function, pain, and quality of life in patients with knee osteoarthritis.   - Strength of Recommendation: Moderate | | Orthoses/braces |
| The following supplements may be helpful in reducing pain and improving function for patients with mild to moderate knee osteoarthritis; however, the evidence is inconsistent/limited and additional research clarifying the efficacy of each supplement is needed. (Turmeric; Ginger extract; Glucosamine; Chondroitin; Vitamin D)   - Strength of Recommendation: Limited | |  |
| Topical NSAIDs should be used to improve function and quality of life for treatment of osteoarthritis of the knee, when not contraindicated.   - Strength of Recommendation: Strong | | Topical NSAIDs |
| Supervised exercise, unsupervised exercise, and/or aquatic exercise are recommended over no exercise to improve pain and function for treatment of knee osteoarthritis.   - Strength of Recommendation: Strong | | Exercise therapy/  referral to PT |
| Neuromuscular training (i.e. balance, agility, coordination) programs in combination with traditional exercise could be used to improve performance-based function and walking speed for treatment of knee osteoarthritis.   - Strength of Recommendation: Moderate | |  |
| Self-management programs are recommended to improve pain and function for patients with knee osteoarthritis.   - Strength of Recommendation: Strong | |  |
| Patient education programs are recommended to improve pain in patients with knee osteoarthritis.   - Strength of Recommendation: Strong | |  |
| Sustained weight loss is recommended to improve pain and function in overweight and obese patients with knee osteoarthritis.   - Strength of Recommendation: Moderate | |  |
| Manual therapy in addition to an exercise program may be used to improve pain and function in patients with knee osteoarthritis.   - Strength of Recommendation: Limited | |  |
| Massage may be used in addition to usual care to improve pain and function in patients with knee osteoarthritis.   - Strength of Recommendation: Limited | | Massage |
| FDA-approved laser treatment may be used to improve pain and function in patients with knee osteoarthritis.   - Strength of Recommendation: Limited | |  |
| Acupuncture may improve pain and function in patients with knee osteoarthritis.   - Strength of Recommendation: Limited | | Acupuncture |
| Modalities that may be used to improve pain and/or function in patients with knee osteoarthritis include:  a. Transcutaneous Electrical Nerve Stimulation (pain)   - Strength of Recommendation: Limited | | TENS |
| Modalities that may be used to improve pain and/or function in patients with knee osteoarthritis include:   1. Percutaneous Electrical Nerve Stimulation (pain and function) 2. Pulsed Electromagnetic Field Therapy (pain)  - Strength of Recommendation: Limited | | NMES |
| Extracorporeal shockwave therapy may be used to improve pain and function for treatment of osteoarthritis of the knee.   - Strength of Recommendation: Limited | |  |
| Oral NSAIDs are recommended to improve pain and function in the treatment of knee osteoarthritis when not contraindicated.   - Strength of Recommendation: Strong | | Oral NSAID |
| Oral acetaminophen is recommended to improve pain and function in the treatment of knee osteoarthritis when not contraindicated.   - Strength of Recommendation: Strong | | Paracetamol (initial medication) |
| Oral narcotics, including tramadol, result in a significant increase of adverse events and are not effective at improving pain or function for treatment of osteoarthritis of the knee.   - Strength of Recommendation: Strong | | Weak opioids |
| Hyaluronic acid intra-articular injection(s) is not recommended for routine use in the treatment of symptomatic osteoarthritis of the knee.   - Strength of Recommendation: Strong | |  |
| Intra-articular (IA) corticosteroids could provide short-term relief for patients with symptomatic osteoarthritis of the knee.   - Strength of Recommendation: Moderate | | Corticosteroid injection |
| Platelet-rich plasma (PRP) may reduce pain and improve function in patients with symptomatic osteoarthritis of the knee.   - Strength of Recommendation: Limited | |  |
| Denervation therapy may reduce pain and improve function in patients with symptomatic osteoarthritis of the knee.   - Strength of Recommendation: Limited | |  |
| Arthroscopy with lavage and/or debridement in patients with a primary diagnosis of knee osteoarthritis is not recommended.   - Strength of Recommendation: Moderate | |  |
| Arthroscopic partial meniscectomy can be used for the treatment of meniscal tears in patients with concomitant mild to moderate osteoarthritis who have failed physical therapy or other nonsurgical treatments.   - Strength of Recommendation: Moderate | |  |
| High tibial osteotomy may be considered to improve pain and function in properly indicated patients with unicompartmental knee osteoarthritis.   - Strength of Recommendation: Limited | |  |
| In the absence of reliable evidence, it is the opinion of the workgroup that the utility/efficacy of dry needling is unclear and requires additional evidence.   - Strength of Recommendation: Consensus | |  |
| In the absence of reliable or new evidence, it is the opinion of the work group not to use free-floating (un-fixed) interpositional devices in patients with symptomatic medial compartment osteoarthritis of the knee.   - Strength of Recommendation: Consensus | |  |

**AAOS (2)**

| **Characteristics** | | |
| --- | --- | --- |
| Year | 2017 | |
| Country | US | |
| Target population | HOA patients | |
| LOE Scale | - Overall Strength of Evidence: Strong   - Description of Evidence Quality: Evidence from two or more “High” quality studies with consistent findings for recommending for or against the intervention. - Overall Strength of Evidence: Moderate   - Description of Evidence Quality: Evidence from two or more “Moderate” quality studies with consistent findings, or evidence from a single “High” quality study for recommending for or against the intervention. - Overall Strength of Evidence: Low Strength Evidence of Conflicting Evidence   - Description of Evidence Quality: : Evidence from two or more “Low” quality studies with consistent findings or evidence from a single “Moderate” quality study recommending for against the intervention or diagnostic or the evidence is insufficient or conflicting and does not allow a recommendation for or against the intervention. - Overall Strength of Evidence: No evidence   - Description of Evidence Quality: There is no supporting evidence, or higher quality evidence was downgraded due to major concerns addressed in the EtD framework. In the absence of reliable evidence, the guideline work group is making a recommendation based on their clinical opinion. | |
| SOR Scale | - Strength of recommendation: Strong   - Overall Strength of Evidence: Strong - Strength of recommendation: Moderate   - Overall Strength of Evidence: Moderate - Strength of recommendation: Limited   - Overall Strength of Evidence: Low Strength Evidence or Conflicting Evidence - Strength of recommendation: Consensus   - Overall Strength of Evidence: No Evidence | |
| **Recommendations (18)** | | **Ambulatory health intervention before TJR quantifiable in routine data from German SHIs** |
| Moderate strength evidence supports that the practitioner could use risk assessment tools to assist in predicting adverse events, assessing surgical risks and educating patients with symptomatic osteoarthritis of the hip undergoing total hip arthroplasty.   - Strength of Recommendation: Moderate | |  |
| Moderate strength evidence supports that obese patients with symptomatic osteoarthritis of the hip, when compared to non-obese patients, may achieve lower absolute outcome scores but a similar level of patient satisfaction and relative improvement in pain and function after total hip arthroplasty.   - Strength of Recommendation: Moderate | |  |
| Limited strength evidence supports that obese patients with symptomatic osteoarthritis of the hip, when compared to non-obese patients, have increased incidence of postoperative dislocation, superficial wound infection, and blood loss after total hip arthroplasty.   - Strength of Recommendation: Limited | |  |
| Moderate strength evidence supports that increased age is associated with lower functional and quality of life outcomes in patients with symptomatic osteoarthritis of the hip undergoing total hip arthroplasty.   - Strength of Recommendation: Moderate | |  |
| Limited strength evidence supports that increased age may be associated with a higher risk of mortality in patients with symptomatic osteoarthritis of the hip undergoing total hip arthroplasty.   - Strength of Recommendation: Limited | |  |
| Limited strength evidence supports that younger age may be associated with a higher risk of revision in patients with symptomatic osteoarthritis of the hip undergoing total hip arthroplasty.   - Strength of Recommendation: Limited | |  |
| Moderate strength evidence supports that mental health disorders, such as depression, anxiety, and psychosis, are associated with decreased function, pain relief, and quality of life outcomes in patients with symptomatic osteoarthritis of the hip who undergo total hip arthroplasty (THA).   - Strength of Recommendation: Moderate | |  |
| Limited strength evidence supports that patients who use tobacco products are at an increased risk for complications after total hip arthroplasty.   - Strength of Recommendation: Limited | |  |
| Strong evidence supports that NSAIDs improve short-term pain, function, or both in patients with symptomatic osteoarthritis of the hip.   - Strength of Recommendation: Strong | | Oral NSAIDs |
| Moderate strength evidence does not support the use of glucosamine sulfate because it did not perform better than placebo for improving function, reducing stiffness and decreasing pain for patients with symptomatic osteoarthritis of the hip.   - Strength of Recommendation: Moderate | |  |
| Strong evidence supports the use of intraarticular corticosteroids to improve function and reduce pain in the short-term for patients with symptomatic osteoarthritis of the hip.   - Strength of Recommendation: Strong | | Corticosteroid injection |
| Strong evidence does not support the use of intraarticular hyaluronic acid because it does not perform better than placebo for function, stiffness, and pain in patients with symptomatic osteoarthritis of the hip.   - Strength of Recommendation: Strong | |  |
| Strong evidence supports the use of physical therapy as a treatment to improve function and reduce pain for patients with osteoarthritis of the hip and mild to moderate symptoms.   - Strength of Recommendation: Strong | | Exercise therapy/  referral to PT |
| Limited evidence supports the use of pre-operative physical therapy to improve early function in patients with symptomatic osteoarthritis of the hip following total hip arthroplasty.   - Strength of Recommendation: Limited | |  |
| Limited evidence supports the use of neuraxial anesthesia compared to general anesthesia to reduce complications in patients with symptomatic osteoarthritis of the hip undergoing total hip arthroplasty.   - Strength of Recommendation: Limited | |  |
| Moderate strength evidence supports that the practitioner could use intravenous or topical tranexamic acid for patients with symptomatic osteoarthritis of the hip who are undergoing total hip arthroplasty (THA) as a part of the effort to reduce blood loss.   - Strength of Recommendation: Moderate | |  |
| Moderate strength evidence supports that there were no clinically significant differences in patient oriented outcomes related to the surgical approach for patients with symptomatic osteoarthritis of the hip undergoing total hip arthroplasty.   - Strength of Recommendation: Moderate | |  |
| Moderate evidence supports the use of post-operative physical therapy because it could improve early function to a greater extent than no physical therapy management for patients with symptomatic osteoarthritis of the hip who have undergone total hip arthroplasty.   - Strength of Recommendation: Moderate | |  |

**ACR (3)**

| **Characteristics** | | |
| --- | --- | --- |
| Year | 2020 | |
| Country | US | |
| Target population | KOA/HOA/hand OA patients | |
| LOE Scale | - High - Moderate - Low - Very low | |
| SOR Scale | - Strongly recommended - Conditionally recommended - Strongly recommended against - Conditionally recommended against - No recommendation - The strength of the recommendation is based on a 70% consensus among the Voting Panel members:   - strong recommendation: clear benefit-harm-ratio of the intervention proved in available evidence (recommendation applicable to almost all patients; only a small proportion of patients should not follow the recommendation)   - conditional recommendation: uncertainty over benefit-harm-ratio of the intervention proved in available evidence (recommendations are value-and preference-sensitive and always require a full shared decision-making approach) | |
| **Recommendations (45)** | | **Ambulatory health intervention before TJR quantifiable in routine data from German SHIs** |
| Exercise is strongly recommended for patients with knee, hip, and/or hand OA. | | Exercise therapy/  referral to PT |
| Balance exercises are conditionally recommended for patients with knee and/or hip OA | |  |
| Weight loss is strongly recommended for patients with knee and/or hip OA who are overweight or obese. | |  |
| Self-efficacy and self-management programs are strongly recommended for patients with knee, hip, and/or hand OA. | |  |
| Tai chi is strongly recommended for patients with knee and/or hip OA. | |  |
| Yoga is conditionally recommended for patients with knee OA. | |  |
| Cognitive behavioral therapy (CBT) is conditionally recommended for patients with knee, hip, and/or hand OA. | |  |
| Cane use is strongly recommended for patients with knee and/or hip OA in whom disease in 1 or more joints is causing a sufficiently large impact on ambulation, joint stability, or pain to warrant use of an assistive device. | | Walking aids |
| Tibiofemoral knee braces are strongly recommended for patients with knee OA in whom disease in 1 or both knees is causing a sufficiently large impact on ambulation, joint stability, or pain to warrant use of an assistive device, and who are able to tolerate the associated inconvenience and burden associated with bracing. | | Orthoses/braces |
| Patellofemoral braces are conditionally recommended for patients with patellofemoral knee OA in whom disease in 1 or both knees is causing a sufficiently large impact on ambulation, joint stability, or pain to warrant use of an assistive device. | | Orthoses/braces |
| Kinesiotaping is conditionally recommended for patients with knee and/or first CMC joint OA. | |  |
| Modified shoes are conditionally recommended against in patients with knee and/or hip OA. | | Shoes/insoles |
| Lateral and medial wedged insoles are conditionally recommended against in patients with knee and/or hip OA. | | Shoes/insoles |
| Acupuncture is conditionally recommended for patients with knee, hip, and/or hand OA. | | Acupuncture |
| Thermal interventions (locally applied heat or cold) are conditionally recommended for patients with knee, hip, and/ or hand OA. | |  |
| Radiofrequency ablation is conditionally recommended for patients with knee OA. | |  |
| Massage therapy is conditionally recommended against in patients with knee and/or hip OA. | | Massage |
| Manual therapy with exercise is conditionally recommended against over exercise alone in patients with knee and/or hip OA. | |  |
| Iontophoresis is conditionally recommended against in patients with first CMC joint OA | |  |
| Pulsed vibration therapy is conditionally recommended against in patients with knee OA. | |  |
| Transcutaneous electrical stimulation (TENS) is strongly recommended against in patients with knee and/or hip OA. | | TENS |
| Topical NSAIDs are strongly recommended for patients with knee OA and conditionally recommended for patients with hand OA. | | Topical NSAIDs |
| Topical capsaicin is conditionally recommended for patients with knee OA and conditionally recommended against in patients with hand OA. | |  |
| Oral NSAIDs are strongly recommended for patients with knee, hip, and/or hand OA. | | Oral NSAIDs |
| Intraarticular glucocorticoid injections are strongly recommended for patients with knee and/or hip OA and conditionally recommended for patients with hand OA. | | Corticosteroid injection |
| Intraarticular glucocorticoid injections versus other injections are conditionally recommended for patients with knee, hip, and/or hand OA. | |  |
| Ultrasound guidance for intraarticular glucocorticoid injection is strongly recommended for injection into hip joints. | |  |
| Acetaminophen is conditionally recommended for patients with knee, hip, and/or hand OA. | | Paracetamol (initial medication) |
| Duloxetine is conditionally recommended for patients with knee, hip, and/or hand OA. | | Duloxetine |
| Tramadol is conditionally recommended for patients with knee, hip, and/or OA. | | Weak opioids |
| Non-tramadol opioids are conditionally recommended against in patients with knee, hand, and/or hip OA with the recognition that they may be used under certain circumstances, particularly when alternatives have been exhausted. | | Strong opioids |
| Colchicine is conditionally recommended against in patients with knee, hip, and/or hand OA. | |  |
| Fish oil is conditionally recommended against in patients with knee, hip, and/or hand OA. | |  |
| Vitamin D is conditionally recommended against in patients with knee, hip, and/or hand OA. | |  |
| Bisphosphonates are strongly recommended against in patients with knee, hip, and/or hand OA. | |  |
| Glucosamine is strongly recommended against in patients with knee, hip, and/or hand OA. | |  |
| Chondroitin sulfate is strongly recommended against in patients with knee and/or hip OA as are combination products that include glucosamine and chondroitin sulfate, but is conditionally recommended for patients with hand OA. | |  |
| Hydroxychloroquine is strongly recommended against in patients with knee, hip, and/or hand OA. | |  |
| Methotrexate is strongly recommended against in patients with knee, hip, and/or hand OA. | |  |
| Intraarticular hyaluronic acid injections are conditionally recommended against in patients with knee and/or first CMC joint OA and strongly recommended against in patients with hip OA. | |  |
| Intraarticular botulinum toxin injections are conditionally recommended against in patients with knee and/or hip OA. | |  |
| Prolotherapy is conditionally recommended against in patients with knee and/or hip OA. | |  |
| Platelet-rich plasma treatment is strongly recommended against in patients with knee and/or hip OA. | |  |
| Stem cell injections are strongly recommended against in patients with knee and/or hip OA. | |  |
| Tumor necrosis factor inhibitors and interleukin-1 receptor antagonists are strongly recommended against in patients with knee, hip, and/or hand OA. | |  |

**APTA (4)**

| **Characteristics** | | |
| --- | --- | --- |
| Year | 2017 | |
| Country | US | |
| Target population | HOA patients | |
| LOE Scale | - I: Evidence obtained from high-quality randomized controlled trials, prospective studies, or diagnostic studies - II:  Evidence obtained from lesser-quality randomized controlled trials, prospective studies, or  diagnostic studies (eg, improper randomization, no blinding, <80% follow-up) - III:  Case-controlled studies or retrospective studies - IV:  Case series - V:  Expert opinion | |
| SOR Scale | - A:  Strong Evidence: A preponderance of level I and/or level II studies support the recommendation. This msut include at least 1 level I study. - B: Moderate Evidence: A single high-quality randomized controlled trial or a preponderance of level II studies support the recommendation. - C: Weak Evidence: A single level II study or a preponderance of level III and level IV studies, including statements of consensus by experts support the recommendation. - D: Conflicting Evidence: Higher- quality studies conducted on this topic disagree with respect to their conclusions. The recommendation is based on these conflicting studies. - E: Theoretical/foundational evidence: A preponderance of evidence from animal or cadaver studies, from conceptual models/principles, or from basic sciences/bench research support this conclusion. - F: Expert opinion: Best practice based on the clinical experience of the guidelines development team. | |
| **Recommendations (15)** | | **Ambulatory health intervention before TJR quantifiable in routine data from German SHIs** |
| Clinicians should use the following criteria to classify adults over the age of 50 years into the International Statistical Classification of Diseases and Related Health Problems (ICD) category of coxarthrosis and the associated International Classification of Functioning, Disability and Health (ICF) impairment-based category of hip pain (b28016 Pain in joints) and mobility deficits (b7100 Mobility of a single joint): moderate anterior or lateral hip pain during weight bearing activities, morning stiffness less than 1 hour in duration after wakening, hip internal rotation range of motion less than 24° or internal rotation and hip flexion 15° less than the nonpainful side, and/or increased hip pain associated with passive hip internal rotation.   - Level of evidence: II - Grade of Recommendation: A | |  |
| Clinicians should revise the diagnosis and change their plan of care, or refer the patient to the appropriate clinician, when the patient’s history, reported activity limitations, or impairments of body function and structure are not consistent with those presented in the diagnosis/classification section of this guideline, or when the patient’s symptoms are not diminishing with interventions aimed at normalization of the patient’s impairments of body function.   - Level of evidence: not reported - Grade of Recommendation: F | | Physician visit for musculoskeletal disorders |
| Clinicians should use validated outcome measures that include domains of hip pain, body function impairment, activity limitation, and participation restriction to assess outcomes of treatment of hip osteoarthritis. Measures to assess hip pain may include the Western Ontario and McMaster Universities Osteoarthritis Index (WOMAC) pain subscale, Brief Pain Inventory (BPI), pressure pain threshold (PPT), and pain visual analog scale (VAS). Activity limitation and participation restriction outcome measures may include the WOMAC physical function subscale, the Hip disability and Osteoarthritis Outcome Score (HOOS), Lower Extremity Functional Scale (LEFS), and Harris Hip Score (HHS).   - Level of evidence: I, II, III - Grade of Recommendation: A | |  |
| To assess activity limitation, participation restrictions, and changes in the patient’s level of function over the episode of care, clinicians should utilize reliable and valid physical performance measures, such as the 6-minute walk test, 30-second chair stand, stair measure, timed up-and-go test, self-paced walk, timed single-leg stance, 4-square step test, and step test.   - Level of evidence: I - Grade of Recommendation: A | |  |
| Clinicians should measure balance performance and activities that predict the risk of falls in adults with hip osteoarthritis, especially those with decreased physical function or a high risk of falls because of past history. Recommended balance tests for patients with osteoarthritis include the Berg Balance Scale, 4-square step test, and timed single-leg stance test.   - Level of evidence: I - Grade of Recommendation: A | |  |
| Clinicians should use published recommendations from the Academy of Geriatric Physical Therapy of the American Physical Therapy Association to guide fall risk management in patients with hip osteoarthritis to assess and manage fall risk.   - Level of evidence: I - Grade of Recommendation: F | |  |
| When examining a patient with hip pain/hip osteoarthritis over an episode of care, clinicians should document the flexion, abduction, and external rotation (FABER or Patrick’s) test and passive hip range of motion and hip muscle strength, including internal rotation, external rotation, flexion, extension, abduction, and adduction.   - Level of evidence: I, III - Grade of Recommendation: A | |  |
| Clinicians should provide patient education combined with exercise and/or manual therapy. Education should include teaching activity modification, exercise, supporting weight reduction when overweight, and methods of unloading the arthritic joints.   - Level of evidence: I, II - Grade of Recommendation: B | | Exercise therapy/  referral to PT |
| Clinicians should provide impairment-based functional, gait, and balance training, including the proper use of assistive devices (canes, crutches, walkers), to patients with hip osteoarthritis and activity limitations, balance impairment, and/or gait limitations when associated problems are observed and documented during the history or physical assessment of the patient.   - Level of evidence: III - Grade of Recommendation: C | | Walking aids |
| Clinicians should individualize prescription of therapeutic activities based on the patient’s values, daily life participation, and functional activity needs.   - Level of evidence: III - Grade of Recommendation: C | |  |
| Clinicians should use individualized flexibility, strengthening, and endurance exercises to address impairments in hip range of motion, specific muscle weaknesses, and limited thigh (hip) muscle flexibility. For group-based exercise programs, effort should be made to tailor exercises to address patients’ most relevant physical impairments. Dosage and duration of treatment for effect should range from 1 to 5 times per week over 6 to 12 weeks in patients with mild to moderate hip osteoarthritis.   - Level of evidence: I, II, III - Grade of Recommendation: A | |  |
| Clinicians should use manual therapy for patients with mild to moderate hip osteoarthritis and impairment of joint mobility, flexibility, and/or pain. Manual therapy may include thrust, nonthrust, and soft tissue mobilization. Doses and duration may range from 1 to 3 times per week over 6 to 12 weeks in patients with mild to moderate hip osteoarthritis. As hip motion improves, clinicians should add exercises including stretching and strengthening to augment and sustain gains in the patient’s range of motion, flexibility, and strength.   - Level of evidence: I, II, III, IV - Grade of Recommendation: A | |  |
| Clinicians may use ultrasound (1 MHz; 1 W/cm2 for 5 minutes each to the anterior, lateral, and posterior hip for a total of 10 treatments over a 2-week period) in addition to exercise and hot packs in the short-term management of pain and activity limitation in individuals with hip osteoarthritis.   - Level of evidence: I - Grade of Recommendation: B | |  |
| Clinicians should not use bracing as a first line of treatment. A brace may be used after exercise or manual therapies are unsuccessful in improving participation in activities that require turning/pivoting for patients with mild to moderate hip osteoarthritis, especially in those with bilateral hip osteoarthritis.   - Level of evidence: IV   Grade of Recommendation: F | | Orthoses/braces |
| In addition to providing exercise intervention, clinicians should collaborate with physicians, nutritionists, or dietitians to support weight reduction in individuals with hip osteoarthritis who are overweight or obese.   - Level of evidence: III - Grade of Recommendation: C | |  |

**AWMF (5)**

| **Characteristics** | | |
| --- | --- | --- |
| Year | 2021 | |
| Country | Germany | |
| Target population | HOA patients | |
| LOE Scale | - 1++  High-quality meta-analyses, systematic reviews of Randomized Controlled Trials (RCTs), or RCTs with a very low risk of bias - 1+ Well-conducted meta-analyses, systematic reviews, or RCTs with a low risk of bias - 1- Meta-analyses, systematic reviews, or RCTs with a low risk of bias - 2++ High quality systematic reviews of case-control or cohort studies, High-quality case-control or cohort studies with a very low risk of confounding or bias and a high probability that the relationship is causal - 2+ Well-conducted case-control or cohort studies with a very low risk of confounding or bias and a moderate probability that the relationship is causal - 2- Case-control or cohort studies with a high risk of confounding or bias and a significant risk that the relationship is not causal - 3 Non-analytic studies, e.g. case reports, case series - 4 Expert opinion | |
| SOR Scale | Expertpanel Consensus:   - strong consensus (>95%) - consensus (>75%) - majority agreement (>50%) - no majority agreement (<50%)   In addition to the evidence, clinical expertise and patient preferences were taken into account when grading the recommendations. Criteria such as consistency of the study results, clinical relevance of the endpoints and effect sizes, benefit-harm ratio, ethical, legal and economic obligations, applicability to the target patient group and the German health care system, as well as the feasibility of implementation in everyday life or in different areas of care were also taken into account in the graduation of the recommendation:   - A: strong recommendation - B: recommendation - 0: neither for or against a recommendation - EK: expert consensus (based on good clinical practice for recommendations without sufficient evidence) | |
| **Recommendations (32)** | | **Ambulatory health intervention before TJR quantifiable in routine data from German SHIs** |
| Die Diagnose Coxarthrose soll klinisch anhand einer spezifischen Anamnese (Hüftschmerzen, Morgensteifigkeit < 60min) und einer spezifischen klinischen Untersuchung (schmerzhafte Innenrotation und eingeschränkte Flexion) gesichert werden.   - Grade of recommendation: EK - Level of evidence: not reported - Expertpanel consensus: 96% | |  |
| Vor Indikationsstellung zur Hüft-TEP sollen relevante Differentialdiagnosen der Coxarthrose ausgeschlossen werden. Dies gilt insbesondere für jüngere Patient*innen, bei denen evtl. noch gelenkerhaltende Operationen möglich sind.   - Grade of recommendation: A - Level of evidence: 2+ - Expertpanel consensus: 96% | |  |
| Zur Diagnosesicherung und Beurteilung des Ausmaßes der degenerativen Veränderungen soll vor Indikationsstellung zu einer Hüft-TEP eine Bildgebung mittels konventionellem Röntgen (Beckenübersicht und eine zweite Ebene des betroffenen Hüftgelenks) vorliegen.   - Grade of recommendation: EK - Level of evidence: not reported - Expertpanel consensus: 100% | | Radiographic assessment (prior to CT, MRI, sonography) |
| Eine weiterführende Bildgebung mittels MRT und / oder CT im Rahmen der Indikationsstellung zur Hüft-TEP soll nur bei Diskrepanz zwischen klinischem und röntgenologischem Befund erfolgen.   - Grade of recommendation: EK - Level of evidence: 4 - Expertpanel consensus: 100% | | Radiographic assessment (prior to CT, MRI, sonography) |
| Bei zweifelhafter oder unklarer Diagnosestellung kann vor Empfehlung zur endoprothetischen Versorgung eine bildgestützte intraartikuläre Infiltration mit einem Lokalanästhetikum erfolgen.   - Grade of recommendation: EK - Level of evidence: not reported - Expertpanel consensus: 93% | |  |
| In der operierenden Einrichtung soll durch eine spezifische Anamnese, klinische Untersuchung und Prüfung der Röntgenaufnahmen der Schweregrad der Coxarthrose beurteilt werden.   - Grade of recommendation: EK - Level of evidence: not reported - Expertpanel consensus: 100% | |  |
| Eine Hüft-TEP-Operation soll nur bei radiologisch nachgewiesener fortgeschrittener Coxarthrose (Kellgren & Lawrence Grad 3 oder 4) erfolgen.   - Grade of recommendation: A - Level of evidence: 2+ - Expertpanel consensus: 96% | | Radiographic assessment (prior to CT, MRI, sonography) |
| Bei Nachweis einer Hüftkopfnekrose sollte ab ARCO Stadium III c die Indikation zur Implantation einer Hüft-TEP überprüft werden.   - Grade of recommendation: B - Level of evidence: 2++ - Expertpanel consensus: 93% | |  |
| Vor Indikationsstellung zu einer Hüft-TEP soll der Leidensdruck der Patient*innen durch die Erhebung folgender Coxarthrose-bedingter Symptome beurteilt werden:   - Schmerzen, - Einschränkungen der Funktion und der Aktivitäten des täglichen Lebens, - Einschränkungen der gesundheitsbezogenen Lebensqualität. - Grade of recommendation: A - Level of evidence: 1+ - Expertpanel consensus: 100% | | Physician visit for musculoskeletal disorders |
| Zur Beurteilung des Leidensdruckes der Patient*innen sollen validierte Instrumente patientenberichteter Outcomes genutzt werden. Dazu bieten sich krankheitsspezifische und generische Instrumente an.   - Grade of recommendation: A - Level of evidence: 2+ - Expertpanel consensus: 100% | |  |
| Die Indikationsstellung zur Hüft-TEP soll in Betracht gezogen werden, wenn Patient*innen, trotz vorangegangener konservativer Therapie, über hohen subjektiven Leidensdruck hinsichtlich   - hüftbezogener Beschwerden (Schmerzen, Funktionseinschränkungen, Einschränkungen bei den Aktivitäten des täglichen Lebens) und - der gesundheitsbezogenen Lebensqualität berichten. - Grade of recommendation: A - Level of evidence: 3 - Expertpanel consensus: 100% | |  |
| Im Aufklärungsgespräch sollen unter Berücksichtigung der individuellen Symptomausprägung die Vor- und Nachteile einer frühen bzw. späten Indikationsstellung zur Hüft-TEP mit den Patient*innen abgewogen werden.   - Grade of recommendation: A - Level of evidence: 2+ - Expertpanel consensus: 100% | |  |
| Vor der Indikationsstellung zur Hüft-TEP sollen Patient*innen mit symptomatischer Coxarthrose mit einer Kombination aus medikamentöser und nicht-medikamentöser konservativer Therapie behandelt werden.   - Grade of recommendation: A - Level of evidence: 1+ - Expertpanel consensus: 96% | | Combination of pharmacological and non-pharmacological therapies (before TJR) |
| Vor der Indikationsstellung zur Hüft-TEP-Operation sollen Patient*innen mindestens die Kernelemente der nicht-medikamentösen konservativen Therapie durchgeführt haben bzw. sollen empfohlen werden:   - Patient*innenedukation (Information, Aufklärung und Beratung zur Erkrankung), - Bewegungstherapie und Förderung der körperlichen Aktivität, - Gewichtsreduktion bei Patient*innen mit Übergewicht und Adipositas. - Grade of recommendation: A - Level of evidence: 1+ - Expertpanel consensus: 89% | | Combination of pharmacological and non-pharmacological therapies (before TJR) |
| Wenn trotz leitliniengerechter konservativer Therapiemaßnahmen über mindestens drei Monate die Patient*innen über einen hohen subjektiven Leidensdruck berichten, sollte die Indikationsstellung zur Hüft-TEP erfolgen.   - Grade of recommendation: B - Level of evidence: 1+ - Expertpanel consensus: 84% | | Referral to orthopaedic surgeon if conservative therapy failed |
| Vor einer Hüft-TEP-Operation soll eine vorangegangene Infektion des zu operierenden Hüftgelenkes auf noch bestehende Aktivität geprüft werden.   - Grade of recommendation: A - Level of evidence: 2+ - Expertpanel consensus: 93% | |  |
| Vor einer Hüft-TEP-Operation soll eine aktive Infektion (insbesondere von Gelenken, Weichteilen oder hämatogen streuend) ausgeheilt sein.   - Grade of recommendation: A - Level of evidence: 2- - Expertpanel consensus: 85% | |  |
| Bei akuten oder chronischen Begleiterkrankungen, die mit einem erhöhten Sterblichkeitsrisiko verbunden sind, soll durch Operateur*innen die Empfehlung zur Durchführung bzw. dem Zeitpunkt der Hüft-TEP-Operation nach anästhesiologischer und gegebenenfalls fachinternistischer Risikoeinschätzung getroffen werden und kann durch eine orthopädisch-unfallchirurgische Zusatzkonsultation bestätigt werden.   - Grade of recommendation: EK - Level of evidence: not reported - Expertpanel consensus: 93% | |  |
| Bei einem BMI ≥ 40 kg/m² soll aufgrund der deutlich erhöhten Komplikationsgefahr eine besonders kritische Abwägung von Nutzen und Risiken der Hüft-TEP-Operation erfolgen.   - Grade of recommendation: A - Level of evidence: 2++ - Expertpanel consensus: 92% | |  |
| Bei Vorliegen modifizierbarer Risikofaktoren sollen Patient*innen über die Möglichkeit aufgeklärt werden, die Hüft-TEP-Operation zu verschieben, um diese Risiken zu minimieren.   - Grade of recommendation: EK - Level of evidence: 2+ - Expertpanel consensus: 100% | |  |
| Raucher*innen soll empfohlen werden, den Nikotinkonsum mindestens einen Monat vor geplanter Hüft-TEP-Operation zu beenden.   - Grade of recommendation: A - Level of evidence: 2++ - Expertpanel consensus: 100% | |  |
| Bei Patient*innen mit Diabetes mellitus sollen die Blutzuckerwerte vor einer HüftTEP-Operation bestmöglich eingestellt sein.   - Grade of recommendation: A - Level of evidence: 2++ - Expertpanel consensus: 96% | |  |
| Ein HbA1c-Wert unter 8% sollte angestrebt werden.   - Grade of recommendation: EK - Level of evidence: 2++ - Expertpanel consensus: 96% | |  |
| Bei einem BMI ≥ 30 kg/m2 sollte Patient*innen empfohlen werden, ihr Gewicht vor der Hüft-TEP-Operation zu reduzieren.   - Grade of recommendation: B - Level of evidence: 2++ - Expertpanel consensus: 92% | |  |
| Eine asymptomatische Bakteriurie soll nicht wegen einer geplanten Hüft-TEP-Operation behandelt werden.   - Grade of recommendation: A - Level of evidence: 2+ - Expertpanel consensus: 89% | |  |
| Bei Verdacht auf eine psychische Erkrankung sollte den Patient*innen vor einer Hüft-TEP-Operation eine fachspezifische Abklärung empfohlen werden.   - Grade of recommendation: EK - Level of evidence: 2+ - Expertpanel consensus: 89% | |  |
| Vor einer Hüft-TEP-Operation soll eine Anämiediagnostik erfolgen und im Falle einer behandlungsbedürftigen Anämie eine Optimierung vorgenommen werden.   - Grade of recommendation: A - Level of evidence: 2+ - Expertpanel consensus: 90% | |  |
| Nach einer intraartikulären Injektion von Cortikosteroiden sollte eine Hüft-TEP-Operation frühestens nach 6 Wochen erfolgen, zu empfehlen jedoch erst nach 3 Monaten.   - Grade of recommendation: B - Level of evidence: 2++ - Expertpanel consensus: 88% | | Corticosteroid injection |
| Patient*innen sollen dazu angeregt werden, individuelle Therapieziele zu formulieren. Die gemeinsam erarbeiteten Therapieziele sollten dokumentiert werden.   - Grade of recommendation: EK - Level of evidence: not reported - Expertpanel consensus: 93% | |  |
| Patient*innen sollen darüber aufgeklärt werden, inwiefern sich die individuellen Therapieziele durch eine Hüft-TEP oder alternative Therapieoptionen realisieren lassen.   - Grade of recommendation: EK - Level of evidence: not reported - Expertpanel consensus: 100% | |  |
| Für das Beratungs- und Aufklärungsgespräch sollen patientenverständliche Informationsmaterialien genutzt werden.   - Grade of recommendation: EK - Level of evidence: not reported - Expertpanel consensus: 93% | |  |
| Für die gemeinsam getroffene Entscheidung zur Hüft-TEP sollen sich Patient*innen und Operateur*innen darin einig sein, dass der zu erwartende Nutzen der Therapie die möglichen Risiken überwiegt.   - Grade of recommendation: EK - Level of evidence: not reported - Expertpanel consensus: 93% | |  |

**AWMF (6)**

| **Characteristics** | | |
| --- | --- | --- |
| Year | 2019 | |
| Country | Germany | |
| Target population | HOA patients | |
| LOE Scale | Expertpanel Consensus:   - strong consensus (>95%) - consensus (>75% - 95%) - majority consensus (>50% - 75%) - no majority consensus (<50%) | |
| SOR Scale |  |  |
| **Recommendations (56)** | | **Ambulatory health intervention before TJR quantifiable in routine data from German SHIs** |
| Die Diagnose einer Koxarthrose kann in den meisten Fällen mit hinreichender Wahrscheinlichkeit anhand von Anamnese sowie klinischem und radiologischem Befund gestellt werden.   - Expertpanel consensus: strong consensus | | Radiographic assessment (prior to CT, MRI, sonography) |
| Für die Diagnose einer Koxarthrose sollen die Kriterien des American College of Rheumatology (ACR) erfüllt sein.   - Expertpanel consensus: consensus | |  |
| Bei Angabe von Hüftbeschwerden sollten in der allgemeinen Anamnese folgende Daten erhoben werden:   - die persönlichen Daten des Patienten - die Sozial-, Berufs- und Familienanamnese • Stoffwechselstörungen - vorangegangene Hüftgelenkserkrankungen - andere Gelenkerkrankungen • frühere Verletzungen - frühere Krankenhausaufenthalte und Operationen - Nikotin- und Alkoholabusus - körperliche Belastung (sportlich, beruflich) - Medikamentenanamnese. - Expertpanel consensus: strong consensus | |  |
| Bei Angabe von Hüftbeschwerden sollten in der speziellen Anamnese folgende Daten erfragt werden:   - Schmerzen in der Hüfte - Ruhe-/Nachtschmerzen - länger als 30 min. und kürzer als 60 min. andauernde   Morgensteifigkeit in der Hüfte - eine schmerzhafte Innenrotation - eine Bewegungseinschränkung - die maximale Gehstrecke - Schmerzhaftigkeit anderer Gelenke inkl. Rückenschmerzen - eine vorausgegangene Behandlung des betroffenen Gelenkes. - Expertpanel consensus: strong consensus | | Physician visit for musculoskeletal disorders |
| Die allgemeine klinische Untersuchung bei Vorliegen von Hüftbeschwerden sollte folgende Punkte umfassen:   - Inspektion (Schwellung, Rötung, Überwärmung), - Beurteilung der Durchblutung, - orientierende neurologische Untersuchung (Motorik, Sensibilität) der   unteren Extremitäten - Expertpanel consensus: strong consensus | |  |
| Die spezielle klinische Untersuchung bei Vorliegen von Hüftbeschwerden sollte folgende Punkte umfassen:   - Gangbild - Beckenstand und Beinlänge - Beinachse - Trophik und Funktion der Bein- und Glutealmuskulatur - Leistendruck-, Trochanterklopf- und –druckschmerz - Bewegungsausmaß der betroffenen Hüfte - Bewegungsausmaß der kontralateralen Hüfte - Bewegungsausmaß der benachbarten Gelenke. - Expertpanel consensus: strong consensus | |  |
| Bei anhaltenden Hüftbeschwerden, abhängig von Alter, Dauer der Schmerzen und möglichen Differentialdiagnosen, sollte eine bildgebende Diagnostik durchgeführt werden.   - Expertpanel consensus: strong consensus | | Radiographic assessment (prior to CT, MRI, sonography) |
| Im Rahmen der radiologischen Diagnostik bei Verdacht auf Koxarthrose sollte zunächst eine ap-Aufnahme der betroffenen Hüfte zur Diagnosesicherung und Beurteilung des Ausmaßes der degenerativen Veränderungen angefertigt werden.   - Expertpanel consensus: consensus | |  |
| Zur Differentialdiagnostik und Therapieplanung sollten darüber hinaus eine Beckenübersichtsaufnahme und/oder weitere Röntgenaufnahmen (z. B. Axialaufnahme) veranlasst werden.   - Expertpanel consensus: strong consensus | | Radiographic assessment (prior to CT, MRI, sonography) |
| Die Indikation zur Sonographie kann bestehen, wenn eine Diskrepanz zwischen Klinik und Röntgenbild vorliegt oder wenn die Ursache der Gelenkbeschwerden nach Anamnese sowie klinischer und radiologischer Untersuchung noch unklar ist.   - Expertpanel consensus: strong consensus | | Radiographic assessment (prior to CT, MRI, sonography) |
| Die Indikation zur MRT kann bestehen:   - bei Diskrepanz zwischen Klinik und Röntgenbild bzw. CT - bei ausbleibender Besserung unter Standardtherapie - bei Gelenkbeschwerden, deren Ursache nach Anamnese sowie   klinischer und radiologischer Untersuchung noch unklar ist - Expertpanel consensus: strong consensus | | Radiographic assessment (prior to CT, MRI, sonography) |
| Die Indikation zur CT kann bestehen:   - bei Diskrepanz zwischen Klinik und Röntgenbild bzw. MRT - bei ausbleibender Besserung unter Standardtherapie - bei Gelenkbeschwerden, deren Ursache nach Anamnese sowie   klinischer und radiologischer Untersuchung noch unklar ist. - Expertpanel consensus: consensus | | Radiographic assessment (prior to CT, MRI, sonography) |
| Die Indikation zur Szintigraphie kann bestehen:   - bei Diskrepanz zwischen Klinik und Röntgenbild - bei ausbleibender Besserung unter Standardtherapie - bei Verdacht auf Entzündung oder Tumor - bei Gelenkbeschwerden, deren Ursache nach Anamnese sowie   klinischer und radiologischer Untersuchung noch unklar ist - Expertpanel consensus: consensus | |  |
| Die radiologische Stadieneinteilung der Koxarthrose sollte nach KELLGREN und LAWRENCE (1957) in 4 Schweregrade erfolgen:   - Grad I: Gelenkspaltverschmälerung möglich, Osteophyten möglich - Grad II: Gelenkspaltverschmälerung sicher, Osteophyten möglich,   minimale Sklerose - Grad III: Deutliche Gelenkspaltverschmälerung, geringe Osteophyten,   geringe Sklerose - Grad IV: Erhebliche Gelenkspaltverschmälerung, große Osteophyten,   Sklerose, Zysten, ausgeprägte Deformierung. - Expertpanel consensus: consensus | |  |
| Für die klinische Stadieneinteilung der Koxarthrose können folgende Scores verwendet werden:   - Western Ontario Mac Master Arthritis Center (WOMAC) Arthrose-Index von Bellamy und   Buchanan (1986) - Harris Hip Score (HHS, 1969) - Score nach Merle d'Aubignè - Score nach Lequesne et al. (1987) - SF-36-Fragebogen. - Expertpanel consensus: consensus | |  |
| Bei Hüftbeschwerden sollten folgende intraartikulären Differentialdiagnosen zur Koxarthrose in Betracht gezogen werden, ihr Ausschluss muss nicht dokumentiert werden:  Infektionen (bakteriell, viral)   - Infektionen (bakteriell, viral) - die Chondromatose - Schenkelhals- und Azetabulum-Frakturen - die Hüftdysplasie - Labrumeinrisse - Hüftkopfnekrosen - entzündlich-rheumatische Gelenkerkrankungen - femoroazetabuläres Impingement. - Expertpanel consensus: consensus | |  |
| Bei Hüftbeschwerden sollten folgende extraartikuläre Differentialdiagnosen zur Koxarthrose in Betracht gezogen werden, ihr Ausschluss muss nicht dokumentiert werden:   - vertebragene Ursachen - intraabdominelle Erkrankungen - Leisten-, Obturatorius-, Schenkelhernien - pseudo-radikuläre Syndrome - das Piriformis-Syndrom - Bursitiden - Affektionen des Iliosakralgelenkes - extraartikuläre proximale Femurfrakturen - Extraartikuläre Impingement-Formen (knöcherne als auch weichteilige) - neurogene Inguinalsyndrome - Osteomyelitiden - Primärtumoren - Metastasen - das Syndrom der schnappenden Hüfte - gelenknahe Insertionstendinopathien - pelvine, inguinale, retroperitoneale Angiopathien - das Hamstring-Syndrom - Expertpanel consensus: consensus | |  |
| Zur Verbesserung der Gelenkfunktion und Schmerzreduktion soll die Konsens Hydrotherapie, insbesondere die Therapie im Bewegungsbad, als physikalische Therapiemaßnahme bei Koxarthrose Anwendung finden.   - Expertpanel consensus: consensus | |  |
| Weitere physikalische Therapieverfahren (Elektrotherapie/Ultraschalltherapie, Massage, Wärme- und Kälteapplikation, Balneotherapie) können zu einer Symptomlinderung beitragen.   - Expertpanel consensus: consensus | | TENS; Massage |
|  |  |  |
| Die physiotherapeutische Behandlung der Koxarthrose beinhaltet als Kernelement die Bewegungstherapie und sollte in Abhängigkeit von Alter, Komorbidität, Schmerzintensität und Bewegungseinschränkungen sowohl Übungen zur Kräftigung als auch zur generellen Steigerung der körperlichen Belastungsfähigkeit umfassen.   - Expertpanel consensus: consensus | |  |
| Physiotherapeuten sollten dazu frühzeitig in die Festlegung einer Behandlungsstrategie zur Symptomkontrolle und Verbesserung der funktionellen Beweglichkeit einbezogen werden.   - Expertpanel consensus: strong consensus | | Exercise therapy/  referral to PT |
| Im Rahmen der Behandlung sollen Instruktionen zum Selbstmanagement vermittelt werden. Anleitung für Techniken und Fähigkeiten zum Schmerzmanagement, Entspannung und Animation zu regelmäßiger Bewegung stellen hierbei die Inhalte für diese Instruktionen dar.   - Expertpanel consensus: strong consensus | |  |
| Techniken der Manuellen Therapie können zur Verbesserung der gestörten Gelenkbeweglichkeit und zur Schmerzlinderung als ergänzende Maßnahme angewendet werden.   - Expertpanel consensus: consensus | |  |
| Akupunktur kann zusätzlich zur Standardtherapie zu einer Verbesserung der Gelenkfunktion und der allgemeinen Lebensqualität bei Koxarthrose beitragen.   - Expertpanel consensus: majority consensus | | Acupuncture |
| Bei Koxarthrose können NSAR und Coxibe eingesetzt werden bei inadäquater Schmerzlinderung durch andere Therapiemaßnahmen.   - Expertpanel consensus: strong consensus | | Oral NSAID |
| NSAR und Coxibe sollten bei Koxarthrose in der niedrigsten effektiven Dosis und so kurz wie möglich eingesetzt werden.   - Expertpanel consensus: consensus | | Oral NSAID |
| Metamizol kann kurzzeitig als Analgetikum bei Gegenanzeigen oder einer Unverträglichkeit von NSAR und Coxibe eingesetzt werden.   - Expertpanel consensus: consensus | |  |
| Vor der Gabe von Metamizol soll eine Risikoaufklärung erfolgen.   - Expertpanel consensus: consensus | |  |
| Der kurzfristige Einsatz von schwachen Opioiden kann bei nicht operablen Patienten oder bei Patienten, die für kurze Zeit bis zu einer Operation begleitet werden, in Erwägung gezogen werden. Opioide der Stufe 2 WHO, wie z.B. Tramadol sollten primär dann eingesetzt werden, wenn Stufe 1 Medikation unwirksam oder aus anderen Gründen kontraindiziert sind.   - Expertpanel consensus: consensus | | Weak opioids |
| Opioide sollten dann für den kurzfristigen Einsatz in der niedrigsten wirksamen Dosis verwendet werden.   - Expertpanel consensus: consensus | | Weak opioids |
| Die Gabe von Glucosamin kann bei Patienten mit NSAR-Unverträglichkeit in Erwägung gezogen werden.   - Expertpanel consensus: consensus | |  |
| Das symptomatische FAI sollte behandelt werden.   - Expertpanel consensus: consensus | |  |
| Die symptomatische Labrumläsion auf dem Boden eines FAIs soll mit dem Ziel der Schmerzreduktion sowie aus biologischen und biomechanisch experimentellen Überlegungen operativ therapiert werden.   - Expertpanel consensus: majority consensus | |  |
| Bei Patienten mit lokalisiert vollschichtigen und klinisch symptomatischen Knorpelschäden des Hüftgelenks sollte bei geeigneter Indikation und nach Korrektur ggf. bestehender mechanischer Präarthrosen eine knorpelreparative Therapie durchgeführt werden.   - Expertpanel consensus: consensus | |  |
| Bei osteochondralen Defekten des Hüftkopfs kann ein osteochondraler Transfer oder nach knöcherner Defektauffüllung, z.B. mittels impaktierter Spongiosa oder Knochenstanzzylindern aus dem vorderen Beckenkamm, eine zellbasierte Knorpeltherapie indiziert sein.   - Expertpanel consensus: consensus | |  |
| Zur Indikationsstellung einer gelenkerhaltenden Operation soll eine standardisierte ap-Aufnahme des Beckens sowie eine 2. Ebene der betroffenen Hüfte durchgeführt werden.   - Expertpanel consensus: strong consensus | |  |
| Es sollten die relevanten radiologischen Parameter des Hüftgelenkes bestimmt und mit den Normwerten verglichen werden. Bei speziellen Fragestellungen können zusätzliche Spezialaufnahmen (z.B. faux profile oder Rippstein II) erfolgen.   - Expertpanel consensus: strong consensus | |  |
| Für eine ausreichende diagn. Beurteilung der Rotation und Inklination des Beckens soll bei Frauen auf den Gonadenschutz verzichtet werden, bei Männern soll der Gonadenschutz distal der Symphyse positioniert werden.   - Expertpanel consensus: consensus | |  |
| Folgende Vermessungen/Angaben sollten im Bereich des Beckens vorgenommen werden:   - a. Gelenkspaltweite, - LCE-Winkel, - Tragflächenwinkel, - Crossing-Zeichen vorhanden ja/nein, - Posterior wall sign vorhanden ja/nein, - Ischial spine sign vorhanden ja/nein - Folgende Vermessungen/Angaben sollten im Bereich des   Femurs vorgenommen werden: a. Alpha-Winkel Optional kann   vermessen werden: Torsion Femur, Caxa vara/valga (ggf.   zusätzliche Rö-Aufnahmen erforderlich), CCD-Winkel   (Dunn/Rippstein I + II Voraussetzung), Fovea-Stellung. - Expertpanel consensus: consensus | |  |
| Bei einem gelenkerhaltenden Therapieansatz sollte präoperativ ein MRT durchgeführt werden.   - Expertpanel consensus: strong consensus | | Radiographic assessment (prior to CT, MRI, sonography) |
| Zur differentialdiagnostischen Sicherung der intraartikulären Schmerzgenese sollte im Zweifelsfall eine fluoroskopisch oder sonographisch gesteuerte Punktion des Hüftgelenkes mit Infiltration eines Lokalanästhetikums erfolgen.   - Expertpanel consensus: strong consensus | |  |
| Die kontinuierliche Traktion zur Behandlung des zentralen Kompartimentes soll so kurz wie möglich gehalten werden.   - Expertpanel consensus: strong consensus | |  |
| Folgende Ausstattung der Arthroskopieeinheit sollte vorliegen:   1. Kamerasystem mit 70° Optik und Bild- und Videoaufnahmefunktion 2. Rollenpumpe zur Erzielung eines kontinuierlichen Druckes der   Arthroskopieflüssigkeit oder/und CO2 Gasarthroskopiesystem 3. Shaversystem mit mindestens einem   Weichteilresektionsaufsatz und einem   Knochenfräsaufsatz(wenn eine knöcherne Resektion geplant   ist) 4. Einheit zur Elektrokautherisierung / Koagulationselektrode v.   Speicher oder Druckeinheit zur Bild- und Videodokumentation.  - Expertpanel consensus: strong consensus | |  |
| Ein Röntgenbildverstärker zum sicheren Legen der Zugänge, intra- und postoperativen Überprüfung und Dokumentation der knöchernen Resektionen soll vorhanden sein. Der Röntgenbildverstärker soll eine Dokumentationsmöglichkeit durch Papierausdruck oder/und digitaler Bildspeicherung besitzen.   - Expertpanel consensus: strong consensus | |  |
| Aufgrund der besonderen Gelenkanatomie sollten überlange und besonders geformte Instrumente verwendet werden. (z.B. Ankersysteme für die Labrumrefixation, Bohrer, Instrumente für die Knorpeltherapie, Fasszangen, Kapselmesser, etc.).   - Expertpanel consensus: strong consensus | |  |
| Bei der Korrektur soll ein lateraler CE-Winkel im Normbereich angestrebt werden. Ein Crossing-Zeichen bei fokaler Retroversion sollte behoben werden. Ein intaktes Labrum sollte möglichst erhalten oder refixiert werden.   - Expertpanel consensus: strong consensus | |  |
| Eine Überresektion des Pfannenrandes soll auf alle Fälle vermieden werden, da diese zu einer instabilen, dysplastischen Hüfte mit rascher Degeneration führen kann.   - Expertpanel consensus: strong consensus | |  |
| Eine Labrumnaht/-refixation sollte bei geeigneten Rupturen mittels Ankernähten am Azetabulum durchgeführt werden. Ein Labrumerhalt sollte angestrebt werden.   - Expertpanel consensus: strong consensus | |  |
| Labrumresektion sollte bei stark verknöcherten oder degenerierten Labra und nicht mehr refixierbaren Labrumrupturen erfolgen.   - Expertpanel consensus: strong consensus | |  |
| Labrumrekonstruktion durch allogenes oder autologes Gewebe kann bei teilweisem Labrumverlust durchgeführt werden.   - Expertpanel consensus: consensus | |  |
| Eine Synovektomie sollte bei Pathologien der Gelenkschleimhaut inkl. Probebiopsien erfolgen.   - Expertpanel consensus: strong consensus | |  |
| Freie Gelenkkörper und Chondrome sollen entfernt werden.   - Expertpanel consensus: strong consensus | |  |
| Rupturen des Lig. capitis femoris sollten behandelt werden.   - Expertpanel consensus: consensus | |  |
| Kapseleingriffe:   1. Ein Kapselrelease kann bei Kontrakturen erfolgen. 2. Eine Kapselraffung/Kapselplikatur kann bei   Kapselerweiterungen und Instabilitäten z.B. Grenzdysplasie)   durchgeführt werden. 3. Bei Kapseldefekten sollte eine Kapselrekonstruktion erfolgen.  - Expertpanel consensus: strong consensus | |  |
| Knocheneingriffe:   1. Bei Knochenzysten am Femur und Azetabulum kann deren   Ausräumung mit und ohne Knochenauffüllung erfolgen. 2. Transfemorale Nekrosenausräumungen können arthroskopisch   gestützt mit Auffüllung von Spongiosa durchgeführt werden.  - Expertpanel consensus: strong consensus | |  |
| Bei Patienten mit lokalisiert vollschichtigen und klinisch symptomatischen Knorpelschäden des Hüftgelenks sollte bei geeigneter Indikation und nach Korrektur ggf. bestehender mechanischer Präarthrosen eine knorpelreparative Therapie an Gelenkpfanne (häufig) und Femurkopf (selten) durchgeführt werden:   1. Abrasionsarthroplastik. 2. Knochenmarkstimulation z.B. durch Mikro- und Nanofrakturierung   oder Bohrungen. 3. Matrixgekoppelte Knochenmarkstimulation 4. Knorpelprobenentnahme für eine geplante MACT aus einem wenig   belasteten Knorpelbereich des Femurs oder des Azetabulums. 5. Autologe matrixgekoppelte Chondrocytentransplantation (zweizeitig).  - Expertpanel consensus: strong consensus | |  |

**AWMF (7)**

| **Characteristics** | | |
| --- | --- | --- |
| Year | 2018 | |
| Country | Germany | |
| Target population | KOA patients | |
| LOE Scale | Expertpanel Consensus:   - strong consensus (>95%) - consensus (>75% - 95%) - majority consensus (>50% - 75%) - no majority consensus (<50%) | |
| SOR Scale |  |  |
| **Recommendations (44)** | | **Ambulatory health intervention before TJR quantifiable in routine data from German SHIs** |
| Die Diagnose der Gonarthrose soll klinisch und radiologisch gestellt werden.   - Expertpanel consensus: strong consensus | | Radiographic assessment (prior to CT, MRI, sonography) |
| Das Ausmaß der Gelenkschädigung sollte nach radiologischen Kriterien klassifiziert werden.   - Expertpanel consensus: consensus | | Radiographic assessment (prior to CT, MRI, sonography) |
| Es soll eine korrekte endstellige ICD-Kodierung verwendet werden.   - Expertpanel consensus: strong consensus | |  |
| Patienten sollen über die Erkrankung, Vorbeugung der Krankheitsprogression, Verbesserung der Lebensqualität und Mobilität aufgeklärt werden (motivationale Beratung).   - Expertpanel consensus: strong consensus | |  |
| Die Therapie sollte anhand eines individuellen Versorgungsplans mittels gemeinsamer Entscheidungsfindung durch Arzt und Patient erfolgen.   - Expertpanel consensus: strong consensus | |  |
| Eine Kontrolle des Gewichts soll den Patienten empfohlen werden.   - Expertpanel consensus: strong consensus | |  |
| Eine ausführliche Anamnese und körperliche Untersuchung soll vor der Interpretation von Röntgenbildern stehen.   - Expertpanel consensus: strong consensus | | Physician visit for musculoskeletal disorders |
| Die primäre bildgebende Diagnostik sind die konventionellen „Röntgenaufnahmen“. Zur besseren Auswertbarkeit der Röntgenaufnahme in der Frontalebene sollte diese im belasteten Zustand erfolgen. Eine weiterführende bildgebende Diagnostik soll speziellen Fragestellungen vorbehalten bleiben.   - Expertpanel consensus: strong consensus | | Radiographic assessment (prior to CT, MRI, sonography) |
| Die derzeitige Beleglage ist nicht ausreichend, um den Einsatz von Biomarkern für die Diagnose, Prognose oder Therapiekontrolle der Gonarthrose zu empfehlen.   - Expertpanel consensus: strong consensus | |  |
| Die topische Applikation von NSAR bei Kniearthrose sollte vor deren oralen Anwendung zur Analgesie und Funktionsverbesserung erwogen werden.   - Expertpanel consensus: consensus | | Topical NSAID |
| Um das Risiko gastrointestinaler Wirkungen zu verringern, sollen folgende Hinweise berücksichtigt werden:   - Die Einzeldosis sollte ausreichend, aber so niedrig wie möglich sein. - NSAR sollten nicht kombiniert angewendet werden. - Patienten mit erhöhtem gastrointestinalem Risiko sollten eine   Kombination eines NSAR mit PPI erhalten. - Der Patient ist über mögliche gastrointestinale Symptome wie z.B.   Oberbauchschmerzen, Sodbrennen, Dyspepsie zu informieren. - Der topische Einsatz von NSAR (tNSAR) sollte vor deren oralen   Anwendung erwogen werden. - Besondere Vorsicht ist bei Patienten im höheren Lebensalter zu   beachten (siehe Empfehlung ‚höheres Lebensalter’). - Sofern die Applikation eines NSAR bei Patienten mit einem blutenden   Ulkus in der Vorgeschichte notwendig erscheint, ist die Kombination   eines COX-2-Hemmers mit einem PPI zu bevorzugen - Expertpanel consensus: strong consensus | | Oral NSAID treatment with concomitant PPI/ misoprostol in case of Gl risk factors |
| Um das Risiko unerwünschter Wirkungen zu verringern, sollen folgende Hinweise bei Patienten im höheren Lebensalter berücksichtigt werden:   - NSAR bei Patienten im höheren Alter (>60 Jahre): Einsatz von NSAR   mit kurzer Halbwertszeit, Ulkus-Prophylaxe, routinemäßige   Überwachung des Gastrointestinaltraktes und Blutdrucks sowie   der Nierenfunktion, altersadaptierte Reduktion der Tagesdosis. - In höherem Lebensalter (> 75 Jahre) sollten NSAR bevorzugt topisch   eingesetzt werden (siehe Empfehlung ‚Gastrointestinale Wirkung’). - Expertpanel consensus: strong consensus | | Oral NSAID treatment with caution for comorbidities (GI/CV/renal) |
| NSAR sollten bei Patienten mit kardiovaskulären Risikofaktoren (Diabetes mellitus, Rauchen, Hyperlipidämie, Hypertonie) nur nach strenger Indikationsstellung, so niedrig und so kurz wie möglich angewendet werden. Hierbei sollte die bevorzugte Anwendung von Naproxen erwogen werden, wobei auch der zusätzliche Einsatz von PPI zu prüfen ist. Bei diesen Patienten können auch Alternativen (z.B. Hyaluronsäure, schwach wirksame Opioide) in Betracht gezogen werden.   - Expertpanel consensus: consensus | | Oral NSAID treatment with caution for comorbidities (GI/CV/renal) |
| Mögliche Interaktionen mit niedrig dosiertem ASS sowie mit Antihypertonika sollten beachtet werden.   - Expertpanel consensus: consensus | |  |
| Intraartikulär applizierte Corticosteroide sollten in einer möglichst niedrigen, aber wirksamen, Dosierung angewendet werden.   - Expertpanel consensus: strong consensus | | Corticosteroid injection |
| Die aseptische intraartikuläre Injektion von Corticosteroiden sollte leitliniengerecht durchgeführt werden.   - Expertpanel consensus: strong consensus | |  |
| Der kurzfristige Einsatz von schwachen Opioiden kann bei nicht operablen Patienten oder bei Patienten, die für kurze Zeit bis zu einer Operation begleitet werden, sinnvoll sein.   - Expertpanel consensus: consensus | | Weak opioids |
| Die intraartikuläre Hyaluronsäureinjektion kann bei Patienten eingesetzt werden, bei denen der Einsatz von NSAR kontraindiziert ist oder bei denen NSAR nicht ausreichend wirksam sind.   - Expertpanel consensus: consensus | |  |
| Eine Empfehlung für ein bestimmtes Verfahren zur Herstellung von PRP kann derzeit noch nicht gegeben werden.   - Expertpanel consensus: strong consensus | |  |
| Paracetamol sollte bei Patienten mit Gonarthrose nicht angewendet werden.   - Expertpanel consensus: strong consensus | | Paracetamol (initial medication) |
| Maßnahmen der Bewegungstherapie als Kraft-, Ausdauer- und Beweglichkeitstraining sollen zur primären Behandlung der Gonarthrose angewendet werden.   - Expertpanel consensus: strong consensus | | Exercise therapy/  referral to PT |
| Aquatisches Training sollte bei Gonarthrose angewendet werden.   - Expertpanel consensus: strong consensus | |  |
| Elektrophysikalische Therapien können angewendet werden.   - Expertpanel consensus: strong consensus | |  |
| Zur Reduzierung von Schmerz kann TENS eingesetzt werden.   - Expertpanel consensus: strong consensus | | TENS |
| Neuromuskuläre elektrische Stimulation (NMES) sollte zur Muskelkräftigung nicht eingesetzt werden.   - Expertpanel consensus: strong consensus | | NMES |
| Zur Reduzierung von Schmerz kann Lasertherapie in Kombination mit Bewegungstherapie eingesetzt werden.   - Expertpanel consensus: strong consensus | |  |
| Infrarottherapie sollte zur Behandlung der Gonarthrose nicht eingesetzt werden.   - Expertpanel consensus: strong consensus | |  |
| Zur Reduzierung von Schmerz und Verbesserung der Beweglichkeit kann die Stoßwellentherapie eingesetzt werden.   - Expertpanel consensus: strong consensus | |  |
| Zur Reduzierung von Schmerz und zur Verbesserung der Lebensqualität kann Traktionsbehandlung zur Anwendung kommen.   - Expertpanel consensus: strong consensus | |  |
| Ergotherapie kann bei Patienten mit Gonarthrose angewendet werden.   - Expertpanel consensus: strong consensus | |  |
| Akupunktur kann bei Kniearthrose angewendet werden.   - Expertpanel consensus: consensus | | Acupuncture |
| Die Anwendung der Balneotherapie ist insbesondere bei Patienten mit Co-Morbiditäten eine sinnvolle Therapie.   - Expertpanel consensus: strong consensus | |  |
| Zur Blutegeltherapie bei Kniearthrose kann aufgrund der Studienlage keine Aussage gemacht werden.   - Expertpanel consensus: consensus | |  |
| Die Datenlage zu Wirksamkeit und Unbedenklichkeit von Weihrauch ist derzeit nicht ausreichend, um eine Empfehlung abzugeben. Weihrauchpräparate sind in Deutschland nicht als Arzneimittel verfügbar.   - Expertpanel consensus: strong consensus | |  |
| Weitere topische Phytotherapeutika sollten nicht bei Kniearthrose angewendet werden.   - Expertpanel consensus: consensus | |  |
| Schlammpackungen können bei Kniearthrose angewendet werden.   - Expertpanel consensus: strong consensus | |  |
| Die alleinige Arthroskopie mit Lavage und/oder Debridement kann bei klinisch und radiologisch gesicherter Gonarthrose nicht empfohlen werden.   - Expertpanel consensus: strong consensus | |  |
| Arthroskopische Meniskektomie und Entfernung freier Gelenkkörper können auch bei Vorliegen einer Gonarthrose sinnvoll sein.   - Expertpanel consensus: strong consensus | |  |
| Arthroskopische Knorpelersatzverfahren können bei Gonarthrose nicht angewendet werden.   - Expertpanel consensus: strong consensus | |  |
| Osteotomien (valgisierend oder varisierend) können bei Achsdeformitäten indiziert sein.   - Expertpanel consensus: strong consensus | |  |
| Die Radiosynoviorthese (RSO) kann zur Schmerzreduktion bei Gonarthrose nach Ausschöpfen alternativer Therapieverfahren eingesetzt werden.   - Expertpanel consensus: strong consensus | |  |
| Bei isolierter medialer oder lateraler Gonarthrose kann die mediale oder laterale unikondyläre Endoprothese eine mittelfristig effektive therapeutische Option sein.   - Expertpanel consensus: strong consensus | |  |
| Bei isolierter patellofemoraler Gonarthrose kann die trochleare Endoprothese eine mittelfristig therapeutische Option sein. Es existieren jedoch noch keine langfristigen Ergebnisse.   - Expertpanel consensus: strong consensus | |  |
| Bei fortgeschrittener Gonarthrose ist die Totalendoprothese eine langfristig effektive therapeutische Option.   - Expertpanel consensus: strong consensus | | Referral to orthopaedic surgeon if conservative therapy failed |

**DVA (8)**

| **Characteristics** | | |
| --- | --- | --- |
| Year | 2020 | |
| Country | US | |
| Target population | KOA/HOA patients | |
| LOE Scale | - High - Moderate - Low - Very Low | |
| SOR Scale | - Strong for (or “We recommend offering this option …”) - Weak for (or “We suggest offering this option …”) - No recommendation for or against (or “There is insufficient evidence …”) - Weak against (or “We suggest not offering this option …”) - Strong against (or “We recommend against offering this option …”) | |
| **Recommendations (18)** | | **Ambulatory health intervention before TJR quantifiable in routine data from German SHIs** |
| We suggest against obtaining magnetic resonance imaging for the diagnosis of osteoarthritis of the hip and knee.   - Strength of Recommendation: Weak against | | Radiographic assessment (prior to CT, MRI, sonography) |
| We suggest a self-management program, including exercise and weight loss for osteoarthritis of the hip and knee, and bracing for osteoarthritis of the knee.   - Strength of Recommendation: Weak for | | Exercise therapy/  referral to PT;  Orthoses/braces |
| We suggest offering physical therapy as part of a comprehensive management plan for patients with osteoarthritis of the hip or knee.   - Strength of Recommendation: Weak for | | Exercise therapy/  referral to PT |
| We recommend offering topical non-steroidal anti-inflammatory drugs for patients with pain associated with osteoarthritis of the knee.   - Strength of Recommendation: Strong for | | Topical NSAID |
| There is insufficient evidence to recommend for or against the use of topical non-steroidal anti-inflammatory drugs for patients with pain associated with osteoarthritis of the hip.   - Strength of Recommendation: Neither for nor against | | Topical NSAID |
| We suggest offering topical capsaicin for patients with pain associated with osteoarthritis of the knee.   - Strength of Recommendation: Weak for | |  |
| There is insufficient evidence to recommend for or against the use of topical capsaicin for patients with pain associated with osteoarthritis of the hip.   - Strength of Recommendation: Neither for nor against | |  |
| We suggest offering acetaminophen and/or oral non-steroidal anti-inflammatory drugs for pain associated with osteoarthritis of the hip and knee.   - Strength of Recommendation: Weak for | | Paracetamol (initial medication); oral NSAID |
|  |  |  |
| We suggest offering duloxetine as an alternative or adjunctive therapy for patients with an inadequate response or contraindications to acetaminophen or non-steroidal anti-inflammatory drugs for pain associated with osteoarthritis of the knee.   - Strength of Recommendation: Weak for | | Duloxetine |
| We suggest against initiating opioids (including tramadol) for pain associated with osteoarthritis of the hip and knee. For patients already on long-term opioid therapy, refer to the current VA/DoD Clinical Practice Guideline for the Management of Opioid Therapy for Chronic Pain.   - Strength of Recommendation: Weak against | | Weak opioids |
| We suggest offering an intra-articular corticosteroid injection for patients with persistent pain due to osteoarthritis of the knee inadequately relieved by other interventions.   - Strength of Recommendation: Weak for | | Corticosteroid injection |
| We suggest offering intra-articular viscosupplementation injection(s) for patients with persistent pain due to osteoarthritis of the knee inadequately relieved by other interventions.   - Strength of Recommendation: Weak for | |  |
| We suggest against the use of intra-articular viscosupplementation injection(s) of the hip.   - Strength of Recommendation: Weak against | |  |
| There is insufficient evidence to recommend for or against platelet-rich plasma injections for the treatment of osteoarthritis of the hip or knee.   - Strength of Recommendation: Neither for nor against | |  |
| We suggest against stem cell injections (e.g., mesenchymal, adipose-derived, and bone marrow-derived) for the treatment of osteoarthritis of the knee.   - Strength of Recommendation: Weak against | |  |
| There is insufficient evidence to recommend for or against the use of the following dietary supplements or nutraceuticals for the treatment of osteoarthritis of the hip or knee:   - Avocado and soybean extract - Boswellia serrata - Cannabidiol (CBD oil) - Chondroitin - Curcumin (active component of turmeric) - Collagen - Glucosamine - Glucosamine plus chondroitin - Methylsulfonylmethane - Omega-3 fatty acid - Pycnogenol (pine bark) - Rosehip - Traditional Chinese medicine - Vitamin D - Vitamin E - Willow bark extract - Strength of Recommendation: Neither for nor against | |  |
| There is insufficient evidence to recommend for or against the use of complementary and integrative health interventions for the treatment of osteoarthritis of the hip or knee, including:   - Acupuncture - Massage - Light touch - Meditation - Tai chi - Yoga - Strength of Recommendation: Neither for nor against | | Acupuncture;  Massage |
|  |  |  |
| There is insufficient evidence to recommend for or against the use of transcutaneous electrical nerve stimulation for the treatment of pain in osteoarthritis of the knee.   - Strength of Recommendation: Neither for nor against | | TENS |

**ESCEO (9)**

| **Characteristics** | | |
| --- | --- | --- |
| Year | 2019 | |
| Country | not specified (Europe) | |
| Target population | KOA patients | |
| LOE Scale | - Strong do (definitely do it) - Weak do (probably do it) - No recommendation - Weak don’t (probably don’t do it) - Strong don’t (definitely don’t do it) - grading of the recommendation involves:   - description of evidence (the higher the quality of evidence, the more likely a strong recommendation)   - benefit-harm-ratio of the intervention   - position of the intervention within the algorithm Expertpanel Consensus   (the strength of recommendation was determined as “strong” rather than “weak” if at least 75% of the working group members rated a recommendation as “strong”) | |
| SOR Scale |  |  |
| **Recommendations (15)** | | **Ambulatory health intervention before TJR quantifiable in routine data from German SHIs** |
| The ESCEO working group affords a strong recommendation to the application of a core set comprising: information access/education, weight loss, and an exercise program, which is applicable throughout the management of knee OA. | | Exercise therapy/  referral to PT |
| The ESCEO working group gives a weak recommendation that paracetamol (acetaminophen) should not be used on a regular basis as Step 1 long-term background pharmacological therapy for the management of knee OA. | | Paracetamol (initial medication) |
| The ESCEO working group gives a weak recommendation that paracetamol (acetaminophen) at doses no greater than 3 g/day may be used as short-term rescue analgesia only, given on top of a background of Step 1 chronic therapy with SYSADOAs. | | Paracetamol (initial medication) |
| The ESCEO working group affords a strong recommendation to the use of pCGS as Step 1 long-term background therapy for the management of knee OA, and discourages the use of other glucosamine formulations. | |  |
| The ESCEO working group affords a strong recommendation to the use of prescription CS as Step 1 long-term background therapy, as an alternative to pCGS, and the prescription drug should be distinguished from low quality OTC products. | |  |
| The ESCEO working group gives a weak recommendation that a combination of glucosamine and CS should not be used in Step 1 of background therapy, as there is no preparation containing both prescription products and no convincing evidence for existing non-prescription formulations. | |  |
| The ESCEO working group gives a weak recommendation to the use of SYSADOAs other than CS and pCGS (i.e. ASU and diacerein) as alternative Step 1 background therapy. | |  |
| The ESCEO working group affords a strong recommendation to the use of topical NSAIDs as cyclic add-on analgesia in Step 1, for patients who are still symptomatic after the use of Step 1 background therapy, and prior to use of oral NSAIDs. | | Topical NSAID |
| The ESCEO working group affords a strong recommendation to the use of oral NSAIDs (selective or non-selective) as Step 2 therapy, if used only intermittently or for longer cycles; the use of oral NSAIDs should be based on the patient risk profile. | | Oral NSAID |
| The ESCEO working group affords a weak recommendation to the use of IAHA in patients who have contraindications to NSAIDs, or if the patient is still symptomatic despite the use of NSAIDs. | |  |
| The ESCEO working group affords a weak recommendation to the use of IA corticosteroids, which are more effective than IAHA in the first few weeks of treatment in the same patient population; more severe pain may be a better predictor of this short-term efficacy than inflammatory signs. | | Corticosteroid injection |
| The ESCEO working group gives a weak recommendation to the use of short-term weak opioids in Step 3 of the treatment algorithm as the last pharmacological attempt before surgery. | | Weak opioids |
| The ESCEO working group gives a weak recommendation to the use of duloxetine as an alternative to weak opioids in Step 3 of the algorithm, especially in patients with pain from central sensitization. | | Duloxetine |
| The ESCEO working group affords a strong recommendation to total knee replacement surgery for end-stage knee OA patients, which is a highly selective and cost-effective procedure although not devoid of adverse outcomes; the role of other surgical procedures, especially unicompartmental knee replacement, should be further investigated. | | Referral to orthopaedic surgeon if conservative therapy failed |
| The ESCEO working group gives a weak recommendation to the use of classical oral or transdermal opioids in end-stage knee OA patients for whom surgery is contraindicated. | | Strong opioids |

**EULAR (10)**

| **Characteristics** | | |
| --- | --- | --- |
| Year | 2018 | |
| Country | not specified (Europe) | |
| Target population | OA/inflammatory arthritis patients | |
| LOE Scale | - 1A: Meta-analysis of randomised controlled trials - 1B:  At least one randomised controlled trial - 2A:  At least one controlled trial without randomisation - 2B:  At least one type of quasi-experimental study - 3:  Descriptive studies, such as comparative studies, correlation studies or case-control studies - 4:  Expert committee reports or opinions and/or clinical experience of respected authorities | |
| SOR Scale | Strength of recommendations is a combination of the information from the systematic literature review and expert opinion:   - Systematic Review:   - A:  category I evidence   - B: category II evidence or extrapolated recommendations from category I evidence   - C: category III evidence or extrapolated recommendations from category I or II evidence   - D: category IV evidence or extrapolated recommendations from category II or III evidence - Expert opinion: Level of Agreement (LOA): 0-10 numerical rating scale (0=complete disagreement, 10=complete agreement) | |
| **Recommendations (10)** | | **Ambulatory health intervention before TJR quantifiable in routine data from German SHIs** |
| Assessment by the health professional should include the following aspects (the assessment is brief or extensive depending on factors such as available time, whether it is a first or regular consultation, and the needs of the patient):   - Patient’s needs, preferences and priorities regarding pain management and important activities, values and goals in daily life. - Patient’s pain characteristics including severity, type, spread and quality. Previous and ongoing pain treatments and the perceived efficacy. - Current inflammation and joint damage as sources of pain, and whether these are adequately treated. - Pain-related factors that might need attention: (a) the nature and extent of pain-related disability, (b) beliefs and emotions about pain and pain-related disability, (c) social influences related to pain and its consequences, (d) sleep problems and (e) obesity. - Level of evidence: 4 - Strength of recommendation: D - Level of Agreement: 9.3 | | Physician visit for musculoskeletal disorders |
| The patient should receive a personalised management plan with the aim of reducing pain and pain-related distress and improving pain-related function and participation in daily life. This plan is guided by shared decision-making, the expressed needs of the patient, the health professional’s assessment and evidence-based treatment options. A stepped-care approach may include, in step 1, education and self-management support (recommendation 3); in step 2, one or more treatment options by a specialist if indicated (recommendations 4 to 9); or, in step 3, multidisciplinary treatment (recommendation 10).   - Level of evidence: 4 - Strength of recommendation: D - Level of Agreement: 9.0 | |  |
| The patient should receive education.  * All patients have easy access to (1) educational materials (such as brochures or links to online resources with encouragement to stay active, sleep hygiene guidelines and so on), (2) psychoeducation by the health professional and (3) online or face-to-face self-management interventions.   - Level of evidence: 1A - Strength of recommendation: A - Level of Agreement: 9.7 | |  |
| If indicated, the patient should receive physical activity and exercise.  * The health professional and patient appraise whether advice to stay active, supervised physical exercise or multidisciplinary treatment is needed.  * If the patient is not able to initiate physical activity and exercises without help, then consider the possibility for referral to a physiotherapist for individually tailored graded physical exercise or strength training.  * If psychosocial factors such as fear of movement or catastrophising cognitions underlie a disabled, sedentary lifestyle, then consider a multidisciplinary intervention including cognitive – behavioural therapy.   - Level of evidence: 1A - Strength of recommendation: A - Level of Agreement: 9.8 | | Exercise therapy/  referral to PT |
| If indicated, the patient should receive orthotics.  * If a patient has pain during activities of daily living which impedes functioning, orthotics (such as splints, braces, gloves, sleeves, insoles and shoes), daily living aids (such as a tin opener), an assistive device (such as a cane or rollator) or ergonomic adaptation (at home, workplace) can be offered. If the patients wants to use this assistive support, then consider referral to the occupational therapist, who can proceed with several actions: offer education about appropriate ways to use joints and ergonomic principles, appraise the need for the use of an orthotic or assistive device, give advice about how to acquire it, fit the customised aid to the patient, offer training in the use of it, refer to the appropriate specialist who will do this, eg, orthopaedic shoemaker.   - Level of evidence: 1A - Strength of recommendation: A - Level of Agreement: 8.6 | | Orthoses/braces;  Shoes/insoles;  Walking aids |
|  |  |  |
| If indicated, the patient should receive psychological or social interventions.  * If there are indications that social variables or psychological factors interfere with effective pain management and functional status, then consider (depending on the severity) providing basic social and psychological management support or referral to a psychologist, social worker, self-management support programme, CBT or multidisciplinary treatment.  * If psychopathology (eg, depression and anxiety) is present, discuss treatment options with the patient and the patient’s primary care physician.   - Level of evidence: 1A - Strength of recommendation: A   Level of Agreement: 9.5 | |  |
| If indicated, the patient should receive sleep interventions.  * If sleep disturbance is reported, inquire about causes (eg, pain, persistent worrying, poor sleep habits) and offer basic education about good sleep hygiene practices.  * If sleep remains (severely) disturbed, refer to a therapist or programme aimed at restoring sleep, or to a specialised sleep clinic.   - Level of evidence: 1B - Strength of recommendation: B - Level of Agreement: 8.4 | |  |
| If indicated, the patient should receive weight management.  * If the patient is obese, explain to the patient that obesity can contribute to pain and disability. Discuss accessible weight management options with the patient or signpost appropriate specialised weight management support; for example, dietitian, psychologist, community lifestyle services or bariatric clinic/surgery.   - Level of evidence: 1A - Strength of recommendation: A - Level of Agreement: 9.1 | |  |
| If indicated, the patient should receive pharmacological and joint-specific pain treatment according to recent recommendations.  * Ask about the patient’s existing use of prescribed and over-the-counter pain relief including homeopathic remedies and consider if the frequency of use is safe (not over dosing) and appropriately regular. Ask or refer for further specialist or medical advice if there are concerns or if additional pharmacological treatment may be indicated.   - Level of evidence: not reported - Strength of recommendation: not reported - Level of Agreement: 9.5 | |  |
| If indicated, the patient should receive multidisciplinary treatment.  * If more than one treatment options are indicated, for example, to treat psychological distress in combination with a sedentary lifestyle, and if monotherapy failed, consider a multidisciplinary intervention.   - Level of evidence: 4 - Strength of recommendation: D - Level of Agreement: 8.8 | |  |

**EULAR (11)**

| **Characteristics** | | |
| --- | --- | --- |
| Year | 2018 | |
| Country | not specified (Europe) | |
| Target population | OA/inflammatory arthritis patients (exercise therapy) | |
| LOE Scale | - 1A: Meta-analysis of randomised controlled trials - 1B:  At least one randomised controlled trial - 2A:  At least one controlled trial without randomisation - 2B:  At least one type of quasi-experimental study - 3:  Descriptive studies, such as comparative studies, correlation studies or case-control studies - 4:  Expert committee reports or opinions and/or clinical experience of respected authorities | |
| SOR Scale | Strength of recommendations is a combination of the information from the systematic literature review and expert opinion:   - Systematic Review:   - A:  category I evidence   - B: category II evidence or extrapolated recommendations from category I evidence   - C: category III evidence or extrapolated recommendations from category I or II evidence   - D: category IV evidence or extrapolated recommendations from category II or III evidence - Expert opinion: Level of Agreement (LOA): 0-10 numerical rating scale (0=complete disagreement, 10=complete agreement) | |
| **Recommendations (10)** | | **Ambulatory health intervention before TJR quantifiable in routine data from German SHIs** |
| Promoting PA consistent with general PA recommendations should be an integral part of standard care throughout the course of disease in people with RA/SpA/HOA/KOA.   - Level of evidence: 1B - Strength of recommendation: A - Level of Agreement: 9.81 | | Exercise therapy/  referral to PT |
| All healthcare providers involved in the management of people with RA/SpA/HOA/KOA should take responsibility for promoting PA and should cooperate, including making necessary referrals, to ensure that people with RA/SpA/HOA/KOA receive appropriate PA-interventions.   - Level of evidence: 4 - Strength of recommendation: D - Level of Agreement: 9.14 | |  |
| PA interventions should be delivered by healthcare providers competent in their delivery to people with RA/SpA/HOA/KOA.   - Level of evidence: 4 - Strength of recommendation: D - Level of Agreement: 8.86 | | Exercise therapy/  referral to PT |
| Healthcare providers should evaluate the type, intensity, frequency and duration of the people’s actual PA by means of standardised methods to identify which of the four domains of general PA recommendations can be targeted for improvement.   - Level of evidence: 3 - Strength of recommendation: C - Level of Agreement: 9.05 | |  |
| General and disease-specific contraindications for PA should be identified and taken into account in the promotion of PA.   - Level of evidence: 4 - Strength of recommendation: D - Level of Agreement: 9.10 | |  |
| PA interventions should have clear personalised aims, which should be evaluated over time, preferably by use of a combination of subjective and objective measures (including self-monitoring when appropriate).   - Level of evidence: 4 - Strength of recommendation: D - Level of Agreement: 9.05 | |  |
| General and disease-specific barriers and facilitators related to performing PA, including knowledge, social support, symptom control and self-regulation should be identified and addressed.   - Level of evidence: 3 - Strength of recommendation: C - Level of Agreement: 9.19 | |  |
| Where individual adaptations to general PA recommendations are needed, these should be based on a comprehensive assessment of physical, social and psychological factors including fatigue, pain, depression and disease activity.   - Level of evidence: 4 - Strength of recommendation: D - Level of Agreement: 9.24 | | Physician visit for musculoskeletal disorders |
| Healthcare providers should plan and deliver PA interventions that include the behavioural change techniques self-monitoring, goal setting, action planning, feedback and problem solving.   - Level of evidence: 1A - Strength of recommendation: A - Level of Agreement: 9.48 | |  |
| Healthcare providers should consider different modes of delivery of PA (eg, supervised/not-supervised, individual/group, face-to-face/online, booster strategies) in line with people’s preferences.   - Level of evidence: 4 - Strength of recommendation: D - Level of Agreement: 9.00 | |  |

**EULAR (12)**

| **Characteristics** | | |
| --- | --- | --- |
| Year | 2017 | |
| Country | not specified (Europe) | |
| Target population | peripheral joint OA patients (imaging) | |
| LOE Scale | - Ia: evidence for meta-analysis of randomized controlled trials - Ib evidence from at least one randomized controlled trial - IIa: evidence from at least one controlled study without randomization - IIb: evidence from at least one other type of quasi-experimental study - III: evidence from non-experimental descriptive studies, such as comparative studies, correlation studies and case–control studies - IV: evidence from expert committee reports or opinions or clinical experience of respected authorities, or both | |
| SOR Scale | - Level of Agreement (LOA): 0-10 numerical rating scale (0=complete disagreement, 10=complete agreement) | |
| **Recommendations (7)** | | **Ambulatory health intervention before TJR quantifiable in routine data from German SHIs** |
| Imaging is not required to make the diagnosis in patients with typical* presentation of OA. *typical features include: usage-related pain, short duration morning stiffness, age>40, symptoms affecting one or a few joints.   - Level of evidence: III-IV - Level of agreement: 8.7 | | Physician visit for musculoskeletal disorders |
| In atypical presentations imaging is recommended to help confirm the diagnosis of OA and/or make alternative or additional diagnoses.   - Level of evidence: IV - Level of agreement: 9.6 | | Radiographic assessment (prior to CT, MRI, sonography) |
| Routine imaging in OA follow-up is not recommended. However, imaging is recommended if there is unexpected rapid progression of symptoms or change in clinical characteristics to determine if this relates to OA severity or an additional diagnosis.   - Level of evidence: III-IV - Level of agreement: 8.8 | | Radiographic assessment (prior to CT, MRI, sonography) |
| If imaging is needed, conventional (plain) radiography should be used before other modalities. To make additional diagnoses, soft tissues are best imaged by US or MRI and bone by CT or MRI.   - Level of evidence: III-IV - Level of agreement: 8.7 | | Radiographic assessment (prior to CT, MRI, sonography) |
| Consideration of radiographic views is important for optimizing detection of OA features; in particular for the knee, weight-bearing and patellofemoral views are recommended.   - Level of evidence: III - Level of agreement: 9.4 | |  |
| According to current evidence, imaging features do not predict non-surgical treatment response and imaging cannot be recommended for this purpose.   - Level of evidence: II-III - Level of agreement: 8.7 | |  |
| The accuracy of intra-articular injection depends on the joint and on the skills of the practitioner and imaging may improve accuracy. Imaging is particularly recommended for joints that are difficult to access due to factors including site (e.g. hip), degree of deformity and obesity.   - Level of evidence: III-IV - Level of agreement: 9.4 | |  |

**EULAR (13)**

| **Characteristics** | | |
| --- | --- | --- |
| Year | 2013 | |
| Country | not specified (Europe) | |
| Target population | KOA/HOA patients | |
| LOE Scale | - Ia: Meta-analysis of randomized controlled trials - Ib: At least one randomized controlled trial - IIa: At least one controlled study without randomization - IIb: At least one other type of quasi-experimental study - III: Descriptive studies, such as comparative studies, correlation studies or case–control studies - IV: Expert committee reports or opinions and/or clinical experience of respected authorities | |
| SOR Scale | - Level of Agreement (LOA): 0-10 numerical rating scale (0=complete disagreement, 10=complete agreement) | |
| **Recommendations (11)** | | **Ambulatory health intervention before TJR quantifiable in routine data from German SHIs** |
| In people with hip or knee OA, initial assessments should use a biopsychosocial approach including:  a physical status (including pain; fatigue; sleep quality; lower limb joint   status (foot, knee, hip); mobility; strength; joint alignment;   proprioception and posture; comorbidities; weight)  b activities of daily living  c participation (work/education, leisure, social roles)  d mood e health education needs, health beliefs and motivation to self-  manage.   - Level of evidence: Ib, mixed - Level of agreement: 8.6 | | Physician visit for musculoskeletal disorders |
| Treatment of hip and/or knee OA should be individualised according to the wishes and expectations of the individual, localisation of OA, risk factors (such as age, sex, comorbidity, obesity and adverse mechanical factors), presence of inflammation, severity of structural change, level of pain and restriction of daily activities, societal participation and quality of life.   - Level of evidence: Ib, mixed; Ib, knee - Level of agreement: 8.7 | | Physician visit for musculoskeletal disorders |
| All people with knee/hip OA should receive an individualised management plan (a package of care) that includes the core non-pharmacological approaches, specifically: Ib, hip Ib, knee  a information and education regarding OA  b addressing maintenance and pacing of activity  c addressing a regular individualised exercise regimen  d addressing weight loss if overweight or obese  e* reduction of adverse mechanical factors (eg, appropriate footwear)  f consideration of walking aids and assistive technology.   - Level of evidence: Ib, hip; Ib, knee - Level of agreement: 8.7 | | Exercise therapy/  referral to PT;  Walking aids |
| The mode of delivery of exercise education (eg, individual 1:1 sessions, group classes, etc) and use of pools or other facilities should be selected according both to the preference of the person with hip or knee OA and local availability. Important principles of all exercise include: Ia, knee, delivery mode Ia, mixed, water-based exercise  a ‘small amounts often’ (pacing, as with other activities)  b linking exercise regimens to other daily activities (eg, just before   morning shower or meals) so they become part of lifestyle rather   than additional events  c starting with levels of exercise that are within the individual’s   capability, but building up the ‘dose’ sensibly over several months.   - Level of evidence: Ia, knee, delivery mode; Ia, mixed, water-based exercise - Level of agreement: 8.9 | |  |
| People with hip and/or knee OA should be taught a regular individualised (daily) exercise regimen that includes:  a strengthening (sustained isometric) exercise for both legs, including the   quadriceps and proximal hip girdle muscles (irrespective of site or   number of large joints affected)  b aerobic activity and exercise  c adjunctive range of movement/stretching exercises  * Although initial instruction is required, the aim is for people with hip or   knee OA to learn to undertake these regularly on their own in their   own environment.   - Level of evidence: Ia, hip, overall exercise; Ia, knee, overall exercise; Ia, knee, strength; Ia, knee, aerobic; Ia, knee, mixed programmes - Level of agreement: 8.5 | | Exercise therapy/  referral to PT |
| When lifestyle changes are recommended, people with hip or knee OA should receive an individually tailored programme, including long-term and short-term goals, intervention or action plans, and regular evaluation and follow-up with possibilities for adjustment of the programme.   - Level of evidence: Ib, mixed; Ib, knee - Level of agreement: 8.0 | |  |
| To be effective, information and education for the person with hip or knee OA should: Ia, mixed  a be individualised according to the person’s illness perceptions and   educational capability  b be included in every aspect of management  c specifically address the nature of OA (a repair process triggered by a   range of insults), its causes (especially those pertaining to the   individual), its consequences and prognosis  d be reinforced and developed at subsequent clinical encounters; e†   be supported by written and/or other types of information (eg, DVD,   website, group meeting) selected by the individual  f include partners or carers of the individual, if appropriate.   - Level of evidence: Ia, mixed - Level of agreement: 8.4 | |  |
| Education on weight loss should incorporate individualised strategies that are recognised to effect successful weight loss and maintenance*—for example:  a regular self-monitoring, recording monthly weight  b regular support meetings to review/discuss progress  c increase physical activity  d follow a structured meal plan that starts with breakfast  e reduce fat (especially saturated) intake; reduce sugar; limit salt;   increase intake of fruit and vegetables (at least ‘5 portions’ a day)  f limit portion size; g† addressing eating behaviours and triggers to   eating (eg, stress) h† nutrition education  i relapse prediction and management (eg, with alternative coping   strategies)   - Level of evidence: III, hip; Ia, knee - Level of agreement: 9.1 | |  |
| a‡ The use of appropriate and comfortable shoes is recommended.  b Recommendation rejected: a lateral-wedged insole could reduce symptoms in medial knee pain.   - Level of evidence: Ib, knee - Level of agreement: 8.7 | | Shoes/insoles |
| Walking aids, assistive technology and adaptations at home and/or at work should be considered, to reduce pain and increase participation—for example:  a a walking stick used on the contralateral side, walking frames and   wheeled ‘walkers’  b increasing the height of chairs, beds and toilet seats  c hand-rails for stairs  d replacement of a bath with a walk-in shower  e change to car with high seat level, easy access and automatic gear   change   - Level of evidence: Ib, knee - Level of agreement: 8.0 | | Walking aids |
| People with hip or knee OA at risk of work disability or who want to start/return to work should have rapid access to vocational rehabilitation, including counselling about modifiable work-related factors such as altering work behaviour, changing work tasks or altering work hours, use of assistive technology, workplace modification, commuting to/from work and support from management, colleagues and family towards employment   - Level of evidence: III, hip; III, knee - Level of agreement: 8.9 | |  |

**EULAR (14)**

| **Characteristics** | | |
| --- | --- | --- |
| Year | 2005 | |
| Country | not specified (Europe) | |
| Target population | HOA patients | |
| LOE Scale | - Ia: Meta-analysis of RCTs - Ib: RCT - IIa: Controlled study without randomization - IIb: Quasi-experimental study - III: Non-experimental descriptive studies, such as comparative studies, correlation studies or case–control studies - IV: Expert committee reports or opinions or clinical experience of respected authorities, or both | |
| SOR Scale | - A:  category I evidence - B: category II evidence or extrapolated recommendations from category I evidence - C: category III evidence or extrapolated recommendations from category I or II evidence - D: category IV evidence or extrapolated recommendations from category II or III evidence | |
| **Recommendations (10)** | | **Ambulatory health intervention before TJR quantifiable in routine data from German SHIs** |
| The optimal management of hip OA requires a combination of non-pharmacological and pharmacological treatment modalities.   - Level of evidence: IV - Strength of recommendation: D | | Combination of pharmacological and non-pharmacological therapies (before TJR) |
| Treatment of hip OA should be tailored according to:  (a) Hip risk factors (obesity, adverse mechanical factors, physical   activity, dysplasia)  (b) General risk factors (age, sex, comorbidity, co-medication)  (c) Level of pain intensity, disability, and handicap  (d) Location and degree of structural damage  (e) Wishes and expectations of the patient   - Level of evidence: III - Strength of recommendation: not reported | | Physician visit for musculoskeletal disorders |
| Non-pharmacological treatment of hip OA should include regular education, exercise, appliances (stick, insoles), and weight reduction if obese or overweight.   - Level of evidence: Ib (education), III (weight loss) - Strength of recommendation: A (education), N/A (exercise), D (appliances), D (weight loss) | | Exercise therapy/  referral to PT;  Walking aids;  Shoes/insoles |
|  |  |  |
| Because of its efficacy and safety paracetamol (up to 4 g/day) is the oral analgesic of first choice for mildmoderate pain and, if successful, is the preferred long term oral analgesic.   - Level of evidence: Ia - Strength of recommendation: N/A | | Paracetamol (initial medication) |
| NSAIDs, at the lowest effective dose, should be added or substituted in patients who respond inadequately to paracetamol. In patients with increased gastrointestinal risk, non-selective NSAIDs plus a gastroprotective agent, or a selective COX-2 inhibitor (coxib) should be used.   - Level of evidence: Ia - Strength of recommendation: A | | Oral NSAID |
| Opioid analgesics, with or without paracetamol, are useful alternatives in patients in whom NSAIDs, including COX-2 selective inhibitors (coxibs), are contraindicated, ineffective, and/or poorly tolerated.   - Level of evidence: Ib - Strength of recommendation: A | | Weak opioids |
| SYSADOA (glucosamine sulphate, chondroitin sulphate, diacerhein, avocado soybean unsaponifiable, and hyaluronic acid) have a symptomatic effect and low toxicity, but effect sizes are small, suitable patients are not well defined, and clinically relevant structure modification and pharmacoeconomic aspects are not well established.   - Level of evidence: Ib (chondroitin sulphate, avocado soybean) - Strength of recommendation: A (chondroitin sulphate), not recommended (avocado soybean) | |  |
| Intra-articular steroid injections (guided by ultrasound or x ray) may be considered in patients with a flare that is unresponsive to analgesic and NSAIDs.   - Level of evidence: Ib - Strength of recommendation: not recommended | | Corticosteroid injection |
| Osteotomy and joint preserving surgical procedures should be considered in young adults with symptomatic hip OA, especially in the presence of dysplasia or varus/valgus deformity.   - Level of evidence: III - Strength of recommendation: C | |  |
| Joint replacement has to be considered in patients with radiographic evidence of hip OA who have refractory pain and disability.   - Level of evidence: III - Strength of recommendation: C | | Referral to orthopaedic surgeon if conservative therapy failed; |
|  |  | Radiographic assessment (prior to CT, MRI, sonography) |

**EULAR (15)**

| **Characteristics** | | |
| --- | --- | --- |
| Year | 2003 | |
| Country | not specified (Europe) | |
| Target population | KOA patients | |
| LOE Scale | - 1A: Meta-analysis of RCTs - 1B:  At least one RCT - 2A:  At least one controlled study without randomisation - 2B:  At least one type of quasi-experimental study - 3:  Descriptive studies, such as comparative studies, correlation studies or case-control studies - 4:  Expert committee reports or opinions and/or clinical experience of respected authorities | |
| SOR Scale | - A:  category 1 evidence - B: category 2 evidence or extrapolated recommendations from category 1 evidence - C: category 3 evidence or extrapolated recommendations from category 1 or 2 evidence - D: category 4 evidence or extrapolated recommendations from category 2 or 3 evidence | |
| **Recommendations (10)** | | **Ambulatory health intervention before TJR quantifiable in routine data from German SHIs** |
| The optimal management of knee OA requires a combination of non-pharmacological and pharmacological treatment modalities.   - Level of evidence: 1B - Strength of recommendation: A | | Combination of pharmacological and non-pharmacological therapies (before TJR) |
| The treatment of knee OA should be tailored according to:  (a) Knee risk factors (obesity, adverse mechanical factors, physical   activity)  (b) General risk factors (age, comorbidity, polypharmacy)  (c) Level of pain intensity and disability  (d) Sign of inflammation—for example, effusion  (e) Location and degree of structural damage.   - Level of evidence: not reported - Strength of recommendation: not reported | | Physician visit for musculoskeletal disorders |
| Non-pharmacological treatment of knee OA should include regular education, exercise, appliances (sticks, insoles, knee bracing), and weight reduction.   - Level of evidence: 1A (education), 1B (exercise, bracing) - Strength of recommendation: A (education, exercise), B (bracing) | | Exercise therapy/  referral to PT;  Walking aids;  Orthoses/braces;  Shoes/insoles |
| Paracetamol is the oral analgesic to try first and, if successful, the preferred long term oral analgesic.   - Level of evidence: 1B - Strength of recommendation: A | | Paracetamol (initial medication) |
| Topical applications (NSAID, capsaicin) have clinical efficacy and are safe.   - Level of evidence: 1A - Strength of recommendation: A | | Topical NSAID |
| NSAIDs should be considered in patients unresponsive to paracetamol. In patients with an increased gastrointestinal risk, non-selective NSAIDs and effective gastroprotective agents, or selective COX 2 inhibitors should be used.   - Level of evidence: 1A - Strength of recommendation: A | | Oral NSAID |
| Opioid analgesics, with or without paracetamol, are useful alternatives in patients in whom NSAIDs, including COX 2 selective inhibitors, are contraindicated, ineffective, and/or poorly tolerated.   - Level of evidence: 1B - Strength of recommendation: B | | Weak opioids |
| SYSADOA (glucosamine sulphate, chondroitin sulphate, ASU, diacerein, hyaluronic acid) have symptomatic effects and may modify structure.   - Level of evidence: 1A (glucosamine sulphate, chondroitin sulphate) - Strength of recommendation: A (glucosamine sulphate, chondroitin sulphate) | |  |
| Intra-articular injection of long acting corticosteroid is indicated for flare of knee pain, especially if accompanied by effusion.   - Level of evidence: 1B - Strength of recommendation: A | | Corticosteroid injection |
| Joint replacement has to be considered in patients with radiographic evidence of knee OA who have refractory pain and disability.   - Level of evidence: 3 - Strength of recommendation: C | | Referral to orthopaedic surgeon if conservative therapy failed |
|  |  | Radiographic assessment (prior to CT, MRI, sonography) |

**Jarl G, et al. (16)**

| **Characteristics** | | |
| --- | --- | --- |
| Year | 2019 | |
| Country | Scandinavia (Finland, Sweden, Denmark, Norway) | |
| Target population | KOA patients (orthotic treatment) | |
| LOE Scale | not reported | |
| SOR Scale | not reported | |
| **Recommendations (11)** | | **Ambulatory health intervention before TJR quantifiable in routine data from German SHIs** |
| Finland  (1) use of a knee orthosis can reduce pain and improve range-of-  motion of the knee;  (2) a rigid knee orthosis is most appropriate when symptomatic medial   OA of the knee is present;  (3) use of a foot orthosis with a lateral wedge does not reduce pain or   increase range of-motion of the knee;  (4) use of a foot orthosis with a medial wedge reduces pain and   increases range-of-motion of the knee | | Orthoses/braces;  Shoes/insoles |
| Sweden  (1) use of a rigid knee orthosis is not recommended;  (2) use of a soft knee orthosis is not recommended;  (3) the use of medially and laterally wedged insoles should not be   used routinely. They should be utilized in research and  development where systematic routines exist for follow-up. | | Orthoses/braces;  Shoes/insoles |
| Denmark  (1) use of a supinating foot orthosis with a medial wedge can reduce   pain and improve physical function (based on controlled non-  randomized study);  (2) use of a pronating foot orthosis with a lateral wedge can reduce   pain and improve physical function (based on consensus on good   clinical practice);  (3) use of rigid and soft knee orthoses are not recommended | | Orthoses/braces;  Shoes/insoles |
| Norway  1) no specific orthotic intervention recommendation. Though, physical   activity and pain control recommended as goals of appropriate   conservative interventions | |  |

**KNGF (17)**

| **Characteristics** | | |
| --- | --- | --- |
| Year | 2020 | |
| Country | The Netherlands | |
| Target population | KOA/HOA patients (exercise therapy) | |
| LOE Scale | - High - Moderate - Low - Very low   - Evidence considered high in case of RCT; downgrading of evidence level to moderate, low, or very low, based on: - the risk of bias (assessed in accordance with the Cochrane risk of bias tool) - inconsistency of results (studies showing clinical or statistical heterogeneity), - indirectness of the evidence (the study population differed from the target population of our guideline) - imprecision (a low number of studies or included patients, e.g., <300 patients or events) - publication bias | |
| SOR Scale | - strongly recommended - conditionally recommended - conditionally discouraged - strongly discouraged   - discussions of the guideline panel on the benefit-harm-ratio of the intervention, the quality of the evidence, the values and preferences of patients and clinicians, and feasibility, equity, and acceptability of the recommendations. | |
| **Recommendations (11)** | | **Ambulatory health intervention before TJR quantifiable in routine data from German SHIs** |
| Offer exercise therapy to all patients with hip OA in the conservative treatment phase, and make use of the frequency, intensity, time, and type (FITT) principles.   - Level of evidence: moderate - Strength of recommendation: strongly recommended | | Exercise therapy/  referral to PT |
| Offer exercise therapy to all patients with OA of the knee in the conservative treatment phase, and make use of the FITT principles.   - Level of evidence: moderate - Strength of recommendation: strongly recommended | |  |
| Consider offering exercise therapy in the preoperative phase if the patient has an increased risk of delayed recovery following OA-related hip joint replacement. Make use of the FITT principles.   - Level of evidence: moderate - Strength of recommendation: conditionally recommended | |  |
| Consider limiting exercise therapy in the preoperative phase, teaching the patient exercises that he/she can independently perform, and monitoring how the exercises are performed if the risk of delayed postoperative recovery is not increased. Teach all patients to use a walking aid that will be needed in the postoperative phase.   - Level of evidence: moderate - Strength of recommendation: conditionally recommended | |  |
| Consider limiting exercise therapy in the preoperative phase to teaching the patient exercises that he/she can independently perform and monitor how the exercises are performed if the risk of delayed postoperative recovery is not increased. Teach all patients to use a walking aid that will be needed in the postoperative phase.   - Level of evidence: high - Strength of recommendation: conditionally recommended | |  |
| Preferably offer exercise therapy in the postoperative phase following OA-related hip joint replacement if the patient has an increased risk of delayed recovery and/or if complications occur. Make use of the FITT principles.   - Level of evidence: high - Strength of recommendation: conditionally recommended | |  |
| Consider exercise therapy in the postoperative phase following OA-related knee joint replacement if the patient has an increased risk of delayed recovery and/or if complications occur. Make use of the FITT principles.   - Level of evidence: high - Strength of recommendation: conditionally recommended | |  |
| Consider limiting exercise therapy in the postoperative phase to teaching (and monitoring the execution of) exercises that the patient can independently perform, if the risk of delayed postoperative recovery is not increased and there are no postoperative complications.   - Level of evidence: high - Strength of recommendation: conditionally recommended | |  |
| It is not recommended to offer massage therapy to patients with hip or knee OA.   - Level of evidence: very low - Strength of recommendation: conditionally discouraged | | Massage |
| Preferably do not offer treatment with TENS therapy to patients with hip or knee OA. Consider the use of TENS only as a brief intervention for pain reduction to support exercise therapy if exercise therapy is being hampered due to severe pain symptoms.   - Level of evidence: very low - Strength of recommendation: conditionally discouraged | | TENS |
| Do not offer CPM (after total joint replacement surgery), pulsed electromagnetic field therapy, LLLT, passive mobilizations, shock wave therapy, taping, thermotherapy, or ultrasound therapy to patients with hip or knee OA.   - Level of evidence: very low - Strength of recommendation: strongly discouraged | |  |

**MOVE consensus (18)**

| **Characteristics** | | |
| --- | --- | --- |
| Year | 2005 | |
| Country | UK | |
| Target population | KOA/HOA patients (exercise therapy) | |
| LOE Scale | - 1A: Meta-analysis of RCTs - 1B:  At least one RCT - 2A:  At least one CT without randomisation - 2B:  At least one type of quasi-experimental study - 3:  Descriptive studies (comparative, correlation, case-control studies) - 4:  Expert committee reports/opinions and/or clinical opinion of respected authorities | |
| SOR Scale | - A:  Directly based on category 1 evidence - B: Directly based on category 2 evidence or extrapolated recommendations from category 1 evidence - C: Directly based on category 3 evidence or extrapolated recommendations from category 1 or 2 evidence - D: Directly based on category 4 evidence or extrapolated recommendations from category 1, 2 or 3 evidence | |
| **Recommendations (10)** | | **Ambulatory health intervention before TJR quantifiable in routine data from German SHIs** |
| Both strengthening and aerobic exercise can reduce pain and improve function and health status in patients with knee and hip OA.   - Strength of evidence: 1B (knee), 4 (hip) - Strength of recommendation: A (knee), C (hip) | |  |
| There are few contraindications to the prescription of strengthening or aerobic exercise in patients with hip or knee OA.   - Strength of evidence: 4 - Strength of recommendation: C | |  |
| Prescription of both general (aerobic fitness training) and local (strengthening) exercises is an essential, core aspect of management for every patient with hip or knee OA.   - Strength of evidence: 4 - Strength of recommendation: D | | Exercise therapy/  referral to PT |
| Exercise therapy for OA of the hip or knee should be individualized and patient-centred taking into account factors such as age, co-morbidity and overall mobility.   - Strength of evidence: 4 - Strength of recommendation: D | |  |
| To be effective, exercise programmes should include advice and education to promote a positive lifestyle change with an increase in physical activity.   - Strength of evidence: 1B - Strength of recommendation: A | |  |
| Group exercise and home exercise are equally effective and patient preference should be considered.   - Strength of evidence: 1A - Strength of recommendation: A | |  |
| Improvements in muscle strength and proprioception gained from exercise programmes may reduce the progression of knee and hip OA.   - Strength of evidence: 4 - Strength of recommendation: D | |  |
| Adherence is the principal predictor of long-term outcome from exercise in patients with knee or hip.   - Strength of evidence: 4 - Strength of recommendation: D | |  |
| Strategies to improve and maintain adherence should be adopted, e.g. long-term monitoring/review and inclusion of spouse/family in exercise.   - Strength of evidence: 1B - Strength of recommendation: A | |  |
| The effectiveness of exercise is independent of the presence or severity of radiographic findings.   - Strength of evidence: 4 - Strength of recommendation: not recommended | |  |

**MQIC (19)**

| **Characteristics** | | |
| --- | --- | --- |
| Year | 2021 | |
| Country | US | |
| Target population | OA patients | |
| LOE Scale | Levels of Evidence for the most significant recommendations:   - A: randomized controlled trials - B: controlled trials, no randomization - C: observational studies - D: opinion of expert panel | |
| SOR Scale | not reported | |
| **Recommendations (15)** | | **Ambulatory health intervention before TJR quantifiable in routine data from German SHIs** |
| Detailed history (aspirin and other anti-platelet use, pain control with over-the-counter medications, narcotic use, activity tolerance and limitations)   - Level of evidence: not reported | |  |
| Assess behavioral healthstatus including depression, sleep disturbance, and/or chronic pain syndrome.   - Level of evidence: not reported | |  |
| Physical examination, with emphasis on musculoskeletal examination Assess gastrointestinal (GI) risk:   - History of GI bleeding - History of peptic ulcer disease and/or non-steroidal induced GI symptoms - Concomitant use of corticosteroids and/or warfarin - High dose, chronic, or multiple NSAIDs including aspirin - Age > 60 years - Level of evidence: A (Concomitant use of corticosteroids and/or warfarin) | | Physician visit for musculoskeletal disorders |
| Consider racial equity and social determinants of health impact.   - Level of evidence: not reported | |  |
| Multi-faceted individualized treatment plan should include:   - Education and counseling regarding weight reduction and joint protection - Range-of-motion, aerobic and muscle strengthening exercises, aquatic exercises - For patients with functional limitations, consider physical and occupational therapy, manual medicine - Self-management resources (e.g., American Arthritis Foundation self-help tools and resources) - Level of evidence: B (range-of-motion) | | Exercise therapy/  referral to PT |
| Improved sleep hygiene may decrease perception of pain.   - Level of evidence: not reported | |  |
| Assistive devices for ambulation and activities of daily living for selected patients.   - Level of evidence: not reported | |  |
| Initial drug of choice should be individualized based on age, comorbidities and affected joints.   - Level of evidence: not reported | | Oral NSAID treatment with caution for comorbidities (GI/CV/renal) |
| NSAID analgesics:  Use with caution in patients with HTN, CKD and stable CV disorders only when the individual clinical benefit outweighs the cardiovascular or renal risk. If aspirin is used daily, COX-2 offers no advantage over NSAID.   - Level of evidence: not reported | | Oral NSAID treatment with caution for comorbidities (GI/CV/renal) |
| Avoid use of opioids including tramadol. If used, limit to 72 hours.   - Level of evidence: not reported | | Weak opioids |
| Consider acetaminophen at minimum effective dose, lower dose for patients with risk factors for hepatic toxicity (alcohol, drug interactions). Warn patients that many over-the-counter products and prescription analgesics contain acetaminophen and to monitor total dose carefully. Maximum dose from all sources 3 g/d.   - Level of evidence: not reported | | Paracetamol (initial medication) |
| Other alternatives: Nonacetylated salicylate, intra-articular drugs (glucocorticoids, anesthetics), pain-modulating SSRI (venlafaxine, duloxetine), topical preparations (methyl salicylate, diclofenac, or capsaicin [conditionally recommended for Knee only]). Prescription topicals are costly.   - Level of evidence: not reported | |  |
| Other alternatives: Consider NSAID, based on risk. Add proton-pump inhibitor if on aspirin.   - Level of evidence: not reported | | Oral NSAID treatment with concomitant PPI/misoprostol in case of Gl risk factors |
| If high GI risk: NSAID plus PPI. If NSAID not tolerated, Cyclo-oxygenase-2 (COX-2) selective inhibitor.   - Level of evidence: not reported | | Oral NSAID treatment with concomitant PPI/misoprostol in case of Gl risk factors |
| If high GI risk: For those with prior GI bleed avoid all NSAIDs/COX-2. If must use, then COX-2 plus proton-pump inhibitor.   - Level of evidence: D | | Oral NSAID treatment with concomitant PPI/misoprostol in case of Gl risk factors |

**NHG (20)**

| **Characteristics** | | |
| --- | --- | --- |
| Year | 2008 | |
| Country | The Netherlands | |
| Target population | patients with non-traumatic knee problems | |
| LOE Scale | not reported | |
| SOR Scale | not reported | |
| **Recommendations (25)** | | **Ambulatory health intervention before TJR quantifiable in routine data from German SHIs** |
| If examination of the knee does not result in an adequate explanation of the patient’s symptoms, the hip should also be examined. | |  |
| The general practitioner should concentrate on:   - pain: location, duration and course; - swelling; - locking symptoms: ‘locked knee’ or no longer being able to straighten the knee (consistent with an intra-articular condition); - morning stiffness and start-up pain (consistent with an intra-articular condition); - circumstances under which symptoms worsen or decrease (rest, exercise, climbing stairs, playing sports); - relationship with professional or other job-related activities; - functional limitations and hindrances in daily life; knee complaints or previous knee trauma. | | Physician visit for musculoskeletal disorders |
| The GP should actively manage patients with osteoarthritis and regularly evaluate the effects that recommendations are having. | | Physician visit for musculoskeletal disorders |
| The GP asks the patient to uncover both legs; look for left-right differences and carry out the following examination:   - inspection (ventral):  positional abnormalities: varus or valgus;  atrophy of the quadriceps muscles;  swelling: local or diffuse, ventral or dorsal;  widening of the joint; inspection, palpation and range of movement examination (patient in supine position):  local swelling: redness, fluctuation, pain;  ballottement of the patella;  redness and temperature of the knee;  pain on pressure over the joint space;  crepitation during range of movement examination;  active and passive flexion and extension: limited, painful;  hip rotation: limited endorotation (consistent with osteoarthritis). | |  |
| If, after history taking and physical examination, there is still some doubt about the diagnosis, referral for radiographic investigation may be indicated. | | Radiographic assessment (prior to CT, MRI, sonography) |
| The GP should try to differentiate between an intra-articular and an extra-articular disorder. The presence of hydrops, locking symptoms, crepitations and limitations in active and passive movement are indicative of an intra-articular disorder. Besides potential arthritis, diagnoses that can be made are: osteoarthritis: advancing age, short-lasting starting up and morning stiffness (< 30 minutes), bony widening of the joint, varus or valgus position, crepitation on range of movement investigation. | |  |
| The general practitioner should be active in the management of patients with osteoarthritis. Interventions that have been advised should be specifically evaluated to see if they have had the desired effect, and, if necessary, other measures should be taken. By means of guidance, recommendations on physical exercise and the prescription of analgesics, it is possible for general practitioners themselves to care for the majority of patients with osteoarthritis. | |  |
| Refer patients with osteoarthritis, particularly those who are sedentary, to a physical therapist for exercise therapy. | | Exercise therapy/  referral to PT |
| If exercise therapy and medication do not provide enough relief, then transcutaneous electrical nerve stimulation (TENS) may be used. | | TENS |
| Ultrasound treatment has not proved to be effective in osteoarthritis. | |  |
| Several studies have shown that acupuncture has equally little clinically-relevant effect. | | Acupuncture |
| Cooling the knee and massages with ice do not relieve the patient’s symptoms. | | Massage |
| The beneficial effects on the symptoms and on disease progression of using a walking stick have equally little support, but, due to the simplicity of the measure, the use of a stick (on the ‘good side’) can be tried out. | | Walking aids |
| Special shoes are not recommended as their value has not been demonstrated. | | Shoes/insoles |
| In osteoarthritis, as well as more the general recommendations, an analgesic should also be advised. If required, recommend paracetamol for a period of two weeks. Due to its broad safety profile, paracetamol should be the drug of first choice. | | Paracetamol (initial medication) |
| As second choice, or if paracetamol does not give the required result, ibuprofen, diclophenac or naproxen should be given. If necessary, this treatment can be extended by one to two weeks. | | Oral NSAID |
| When choosing from this group of NSAIDs, any comorbidities (such as cardiovascular or gastrointestinal conditions), side effects and interactions (acetylsalicyl acid), and any history of reactions to NSAIDs, should be taken into account. Due to their potential adverse effects, great caution is advised in prescribing NSAIDs to patients over the age of 70, patients with impaired renal function, hypertension, heart failure or atherosclerotic cardiovascular disease, as well as those who are taking anticoagulants. | | Oral NSAID treatment with caution for comorbidities (GI/CV/renal) |
| After a period of a maximum of a few weeks, in the event of worsening of symptoms NSAIDs should only be taken temporarily, at fixed times and only for a pre-arranged period. | |  |
| Topical NSAIDs applied to the skin in the form of a cream or a gel, have fewer gastro-intestinal side effects but are probably only effective for a short time. | | Topical NSAID |
| The addition of an opiod, such as tramadol, gives extra pain relief and is a way of reducing the dosage of the NSAID whilst maintaining adequate pain relief. However, due to their side effects (particularly nausea and dizziness), opioids should be introduced gradually so that their analgesic effect builds up slowly. | | Weak opioids |
| The frequently-occurring side effects of opioids, as well as dependency and withdrawal symptoms impose limits on their long-term use. Long-term use of medication should be avoided. | |  |
| In the event of a flare, or if general measures and analgesics do not give sufficient pain relief, an intra-articular injection of a corticosteroid, e.g. triamcinolone or methylprednisolone, should be considered. Give 20 to 40 mg each time at intervals of 1 to 3 months. | | Corticosteroid injection |
| Intra-articular injections of hyaluronic acid may give some short-term reduction of the pain as well as some functional improvement. As their long-term effects have not be adequately investigated, as they need to be given several times at intervals of a few weeks, and as they are expensive, these injections are not advised for use in general practice. | |  |
| The therapeutic effect of chondroitin and glucosamine in patients with osteoarthritis has not be adequately demonstrated. For this reason, the use of these products is not advised. | |  |
| Patients with osteoarthritis who, despite conservative therapy, continue to suffer severe symptoms and hindrances in their everyday functioning, or in whom hydrops persists, should be referred to an orthopedic surgeon. | | Referral to orthopaedic surgeon if conservative therapy failed |

**NHMRC (21)**

| **Characteristics** | | |
| --- | --- | --- |
| Year | 2018 | |
| Country | Australia | |
| Target population | KOA/HOA patients | |
| LOE Scale | - Very low - Low - Moderate - High | |
| SOR Scale | - Strong recommendation for the intervention:  benefits clearly outweigh the harms of the intervention - Conditional recommendation for the intervention: uncertainty over benefit-harm-ratio (involvement of personal preferences or costs to impact the decision) - Conditional (neutral) recommendation Conditional recommendation against the intervention: uncertainty over benefit-harm-ratio (involvement of personal preferences or costs to impact the decision) - Strong recommendation against the intervention: harms clearly outweigh the benefit of the intervention | |
| **Recommendations (64)** | | **Ambulatory health intervention before TJR quantifiable in routine data from German SHIs** |
| We strongly recommend offering land-based exercise for all people with knee OA to improve pain and function, regardless of their age, structural disease severity, functional status or pain levels.Exercise has also been found to be beneficial for other comorbidities and overall health.  We strongly recommend walking, muscle-strengthening exercise, and specifically, Tai Chi. Clinicians should prescribe an individualised exercise program, taking into account the person’s preference, capability, and the availability of resources and local facilities. Realistic goals should be set. Dosage should be progressed with full consideration given to the frequency, duration and intensity of exercise sessions, number of sessions, and the period over which sessions should occur Attention should be paid to strategies to optimise adherence. Referral to an exercise professional to assist with exercise prescription and provide supervision either in person or remotely may be appropriate for some people.   - Quality of evidence: Low (all land-based, Tai Chi), Very low (walking, muscle strengthening exercise) - Strength of recommendation: Strong for recommendation (all land-based exercise, walking, muscle-strengthening exercise, Tai Chi) | | Exercise therapy/  referral to PT |
| We strongly recommend offering land-based exercise for all people with hip OA to improve pain and function, regardless of their age, structural disease severity, functional status or pain levels. Exercise has also been found to be beneficial for other comorbidities and overall health.  The type of exercise that is most beneficial is not yet known. Clinicians should prescribe an individualised progressive exercise program, taking into account the person’s preference, capability and the availability of local facilities. Realistic goals should be set. Dosage should be progressed with full consideration given to the frequency, duration and intensity of exercise sessions, number of sessions, and the period over which sessions should occur. The clinician should monitor the person’s response to the exercise program, and could try a different form of land-based exercise if improvements are not evident. Attention should be paid to strategies to optimise adherence. Referral to an exercise professional to assist with exercise prescription and provide supervision either in person or remotely may be useful for some people.   - Quality of evidence: Moderate (land-based) - Strength of recommendation: Strong for recommendation (when combining all studies of land-based exercise) | | Exercise therapy/  referral to PT |
| Exercise has been found to be beneficial for other comorbidities and overall health. However, we are unable to specifically recommend either for or against one type of land-based exercise for hip OA over another at this stage. Clinicians should prescribe an individualised progressive exercise program, taking into account the person’s preference, capability and the availability of local facilities. Realistic goals should be set. Dosage should be progressed, with full consideration given to the frequency, duration and intensity of exercise sessions, number of sessions, and the period over which sessions should occur. The clinician should monitor the person’s response to the exercise program and could try a different form of landbased exercise if improvements are not evident. Attention should be paid to strategies to optimise adherence. Referral to an exercise professional to assist with exercise prescription and provide supervision either in person or remotely may be useful for some people.   - Quality of evidence: Very low (walking, muscle strengthening, stationary cycling, Tai Chi, Hatha yoga) - Strength of recommendation: Conditional (neutral) recommendation for recommending one type of land-based exercise over another (e.g. walking, muscle strengthening, stationary cycling, Tai Chi, Hatha yoga) | | Exercise therapy/  referral to PT |
| It may be appropriate to offer aquatic exercise/ hydrotherapy for some people with knee and/or hip OA. This will depend upon personal preference and the availability of local facilities.   - Quality of evidence: Low - Strength of recommendation: Conditional for recommendation | |  |
| We strongly recommend weight management for people with knee and/or hip OA. For those who are overweight (BMI =25 kg/m2) or obese (BMI =30 kg/m2), a minimum weight loss target  of 5–7.5% of body weight is recommended. It is beneficial to achieve a greater amount of weight loss given that a relationship exists between weight loss and symptomatic benefits. Weight loss should be combined with exercise for greater benefits. For people of healthy body weight, education about the importance of maintaining healthy body weight is essential.   - Quality of evidence: Very low - Strength of recommendation: Strong for recommendation | |  |
| We do not recommend offering oral opioids for people with knee and/or hip OA.   - Quality of evidence: Low (knee), Very low (hip) - Strength of recommendation: Strong against recommendation | | Weak opioids;  Strong opioids |
| We do not recommend offering transdermal opioids for people with knee and/or hip OA.   - Quality of evidence: Low - Strength of recommendation: Strong against recommendation | |  |
| We do not recommend offering doxycycline for people with knee and/or hip OA.   - Quality of evidence: Low (knee), Very low (hip) - Strength of recommendation: Strong against recommendation | |  |
| We do not recommend offering strontium ranelate for people with knee and/or hip OA.   - Quality of evidence: Moderate - Strength of recommendation: Strong against recommendation | |  |
| We do not recommend offering IL-1 inhibitors for people with knee and/or hip OA.   - Quality of evidence: Low - Strength of recommendation: Strong against recommendation | |  |
| We do not recommend offering FGF for people with knee and/or hip OA.   - Quality of evidence: Very low - Strength of recommendation: Strong against recommendation | |  |
| We do not recommend offering viscosupplementation injection for people with hip OA.   - Quality of evidence: Low - Strength of recommendation: Strong against recommendation | |  |
| We do not recommend offering stem cell therapy for people with knee and/or hip OA.   - Quality of evidence: Very low - Strength of recommendation: Strong against recommendation | |  |
| We do not recommend offering arthroscopic, lavage and debridement, meniscectomy and cartilage repair for people with knee OA unless the person also has mechanical symptoms of a clinically locked knee as per Australian Knee Society’s ‘Arthroscopy position statement.   - Quality of evidence: Very low (lavage and debridement), Low (meniscectomy), Very low (cartilage repair) - Strength of recommendation: Strong against recommendation | |  |
| It may be appropriate to offer CBT for some people with knee and/or hip OA. Clinicians should consider whether CBT is appropriate, taking into account psychological comorbidities and personal preference. They should be cognisant of issues related to cost and access. It is recommended that CBT is combined with exercise to improve outcomes. CBT may be offered face-to-face or via online programs.   - Quality of evidence: Low (knee), Very low (hip) - Strength of recommendation: Conditional for recommendation | |  |
| It may be appropriate to offer stationary cycling and/or Hatha yoga for some people with knee OA. Exercise has also been found to be beneficial for other comorbidities and overall health Clinicians should prescribe an individualised exercise program, taking into account the person’s preference, capability and the availability of resources and local facilities. Realistic goals should be set. Dosage should be progressed with full consideration given to the frequency, duration and intensity of exercise sessions, number of sessions, and the period over which sessions should occur Attention should be paid to strategies to optimise adherence. Referral to an exercise professional to assist with exercise prescription and to provide supervision either in person or remotely may be appropriate for some people.   - Quality of evidence: Very low - Strength of recommendation: Conditional for recommendation | |  |
| It may be appropriate to offer a short course of massage therapy for some people with knee and/or hip OA. This should be considered only as an adjunctive treatment to enable engagement with active management strategies, and only for short term, cognisant of issues related to cost and access.   - Quality of evidence: Low - Strength of recommendation: Conditional for recommendation | | Massage |
| It may be appropriate to offer a short course of manual therapy (stretching, soft tissue and/or joint mobilisation and/or manipulation) for some people with knee and/or hip OA. This should be considered only as an adjunctive treatment to enable engagement with active management strategies and only for short term, cognisant of issues related to cost and access.   - Quality of evidence: Very low - Strength of recommendation: Conditional for recommendation | |  |
| It may be appropriate to offer a combination of weight management plus exercise for some people with knee and/or hip OA. For those who are overweight (BMI ≥25 kg/m^2^) or obese (BMI ≥30 kg/m^2^), a minimum weight loss target of 5–7.5% of body weight is recommended. It is beneficial to achieve a greater amount of weight loss given that a relationship exists between the amount of weight loss and symptomatic benefits. Weight loss should be combined with exercise for greater benefits. For people of healthy body weight, education about the importance of maintaining healthy body weight is essential.   - Quality of evidence: Low (knee), Very low (hip) - Strength of recommendation: Conditional for recommendation (combination weight management plus exercise) | |  |
| It may be appropriate to offer local heat therapy (eg hot packs) as a self-management home strategy for some people with knee and/or hip OA. This should be considered only as an adjunctive treatment.   - Quality of evidence: Very low - Strength of recommendation: Conditional for recommendation | |  |
| It may be appropriate to offer an assistive walking device (eg cane) for some people with knee and/or hip OA, depending on a person’s preference and capability.   - Quality of evidence: Low (knee), Very low (hip) - Strength of recommendation: Conditional for recommendation | | Walking aids |
| It may be appropriate to offer TENS that can be used at home for some people with knee and/or hip OA. Clinicians need to provide sufficient instructions on selfuse, and consider individual accessibility and affordability.   - Quality of evidence: Very low - Strength of recommendation: Conditional for recommendation | | TENS |
| It may be appropriate to offer oral NSAIDs for some people with knee and/or hip OA. It might be reasonable to trial oral NSAIDs at the lowest effective dose for a short period, then discontinue use if not effective. Clinicians also need to inform people, monitor and capture adverse events, especially gastrointestinal, renal and cardiovascular, which may be associated with use of NSAIDs.   - Quality of evidence: Moderate - Strength of recommendation: Conditional for recommendation | | Oral NSAID; Oral NSAID treatment with caution for comorbidities (GI/CV/renal) |
| It may be appropriate to offer duloxetine for some people with knee and/or hip OA. Duloxetine currently does not have an indication via the TGA for OA, and should be considered as an investigational medication only. It could be considered for some people with knee and/or hip OA when other forms of pain relief are inadequate.   - Quality of evidence: Moderate (knee), Low (hip) - Strength of recommendation: Conditional for recommendation | | Duloxetine |
| It may be appropriate to offer an intra-articular corticosteroid injection for some people with knee and/or hip OA for short-term pain relief. Clinicians need to be cautious of the potential harms of repeated use.   - Quality of evidence: Very low - Strength of recommendation: Conditional for recommendation | | Corticosteroid injection |
| We are unable to recommend either for or against formal face-to-face self-management education programs for people with knee and/or hip OA. However, clinicians should provide information to enhance understanding about OA, its prognosis and its optimal management.   - Quality of evidence: Very low - Strength of recommendation: Conditional (neutral) recommendation | |  |
| We are unable to recommend either for or against the use of varus unloading/realignment braces for people with lateral tibiofemoral compartment knee OA.   - Quality of evidence: Very low (varus unloading/realignment braces – no RCT data) - Strength of recommendation: Conditional (neutral) recommendation (varus unloading/realignment braces) | | Orthoses/braces |
| We suggest not offering valgus unloading/realignment braces for people with medial tibiofemoral compartment knee OA.   - Quality of evidence: Low - Strength of recommendation: Conditional against recommendation | | Orthoses/braces |
| We suggest not offering realigning patellofemoral braces for patellofemoral OA.   - Quality of evidence: Very low - Strength of recommendation: Conditional against recommendation | | Orthoses/braces |
| We are unable to recommend either for or against the use of medial wedged insoles for people with lateral tibiofemoral OA and valgus deformity.   - Quality of evidence: Very low (medial wedged insoles), Very low (shock-absorbing insoles, arch support – no RCT data) - Strength of recommendation: Conditional (neutral) recommendation (medical wedged insoles) | | Shoes/insoles |
| We are unable to recommend either for or against the use of shock-absorbing insoles or arch supports for knee and/or hip OA.   - Quality of evidence: Very low (all hip orthodics– no RCT data) - Strength of recommendation: Conditional (neutral) recommendation (shock-absorbing insoles, arch support) | | Shoes/insoles |
| We suggest not offering lateral wedge insoles for people with medial tibiofemoral knee OA.   - Quality of evidence: Very low - Strength of recommendation: Conditional against recommendation | | Shoes/insoles |
| We suggest not offering unloading shoes, minimalist footwear or rocker-sole shoes for people with symptomatic knee OA. However, clinicians may consider advising people with OA to wear footwear with shock-absorbing properties and avoid high-heeled shoes.   - Quality of evidence: Very low (unloading shoes, minimalist footwear), Low (rocker-sole shoes) - Strength of recommendation: Conditional against recommendation | | Shoes/insoles |
| We are unable to recommend either for or against the use of patellar taping for people with knee OA.   - Quality of evidence: Very low - Strength of recommendation: Conditional (neutral) recommendation | |  |
| We are unable to recommend either for or against electromagnetic/shortwave therapy for people with knee and/or hip OA.   - Quality of evidence: Low (knee), Very low (hip) - Strength of recommendation: Conditional (neutral) recommendation (medical wedged insoles) | |  |
| We are unable to recommend either for or against the use of paracetamol for people with knee and/or hip OA. However, it might be reasonable to trial paracetamol for a short period and then discontinue use if it is not effective. Clinicians also need to monitor and capture adverse events that may be associated with its use.   - Quality of evidence: Very low - Strength of recommendation: Conditional (neutral) recommendation | | Paracetamol (initial medication) |
| We are unable to recommend either for or against the use of topical NSAIDs for people with knee and/or hip OA. It might be reasonable to trial topical NSAIDs for a short period and then discontinue use if not effective. Clinicians also need to monitor and capture the adverse effects along with its use.   - Quality of evidence: Moderate - Strength of recommendation: Conditional (neutral) recommendation | | Topical NSAID |
| We are unable to recommend either for or against the use of topical capsaicin for people with hip OA.   - Quality of evidence: Very low - Strength of recommendation: Conditional (neutral) recommendation | |  |
| We are unable to recommend either for or against the use of PRP injection for people with knee and/or OA.   - Quality of evidence: Very low - Strength of recommendation: Conditional (neutral) recommendation | |  |
| We are unable to recommend for or against the use of ASU for people with knee and/or hip OA.   - Quality of evidence: Very low - Strength of recommendation: Conditional (neutral) recommendation | |  |
| We are unable to recommend for or against the use of Boswellia serrata for people with knee and/or hip OA.   - Quality of evidence: Very low - Strength of recommendation: Conditional (neutral) recommendation | |  |
| We are unable to recommend for or against the use of curcuma/curcuminoid for people with knee and/or hip OA.   - Quality of evidence: Low - Strength of recommendation: Conditional (neutral) recommendation | |  |
| We are unable to recommend either for or against the use of pine bark extract for people with knee and/or hip OA.   - Quality of evidence: Low (knee), Very low (hip) - Strength of recommendation: Conditional (neutral) recommendation | |  |
| We are unable to recommend either for or against the use of collagen for people with knee and/or hip OA.   - Quality of evidence: Low (knee), Very low (hip) - Strength of recommendation: Conditional (neutral) recommendation | |  |
| We are unable to recommend either for or against the use of MSM for people with knee and/or hip OA.   - Quality of evidence: Very low - Strength of recommendation: Conditional (neutral) recommendation | |  |
| We suggest not offering local cold application (eg ice packs) for people with knee and/or hip OA.   - Quality of evidence: Very low - Strength of recommendation: Conditional against recommendation | |  |
| We suggest not offering kinesio taping for people with knee and/or hip OA.   - Quality of evidence: Very low - Strength of recommendation: Conditional against recommendation | |  |
| We suggest not offering electrotherapy modalities of shockwave, interferential or laser for people with knee and/or hip OA.   - Quality of evidence: Low (laser), Very low (shockwave, interferential laser-hip) - Strength of recommendation: Conditional against recommendation | |  |
| We suggest not offering therapeutic ultrasound for people with knee and/or hip OA.   - Quality of evidence: Moderate (knee), Low (hip) - Strength of recommendation: Conditional against recommendation | |  |
| We suggest not offering acupuncture (ie traditional, laser, electrical) for people with knee and/or hip OA.   - Quality of evidence: Low (knee), Very low (hip) - Strength of recommendation: Conditional against recommendation | | Acupuncture |
| We suggest not offering topical capsaicin for people with knee OA.   - Quality of evidence: Low - Strength of recommendation: Conditional against recommendation | |  |
| We suggest not offering bisphosphonates for people with knee and/or hip OA.   - Quality of evidence: Very low - Strength of recommendation: Conditional against recommendation | |  |
| We suggest not offering calcitonin for people with knee and/or hip OA.   - Quality of evidence: Very low - Strength of recommendation: Conditional against recommendation | |  |
| We suggest not offering NGF for people with knee and/or hip OA.   - Quality of evidence: Moderate - Strength of recommendation: Conditional against recommendation | |  |
| We suggest not offering colchicine for people with knee and/or hip OA.   - Quality of evidence: Very low - Strength of recommendation: Conditional against recommendation | |  |
| We suggest not offering methotrexate for people with knee and/or hip OA.   - Quality of evidence: Low - Strength of recommendation: Conditional against recommendation | |  |
| We suggest not offering viscosupplementation injection for people with knee OA.   - Quality of evidence: Low - Strength of recommendation: Conditional against recommendation | |  |
| We suggest not offering dextrose prolotherapy for people with knee and/or hip OA.   - Quality of evidence: Low - Strength of recommendation: Conditional against recommendation | |  |
| We suggest not offering glucosamine for people with knee and/or hip OA.   - Quality of evidence: Very low (knee), Low (hip) - Strength of recommendation: Conditional against recommendation | |  |
| We suggest not offering chondroitin for people with knee and/or hip OA.   - Quality of evidence: Very low - Strength of recommendation: Conditional against recommendation | |  |
| We suggest not offering glucosamine and chondroitin in compound form for people with knee and/or hip OA.   - Quality of evidence: Very low - Strength of recommendation: Conditional against recommendation | |  |
| We suggest not offering vitamin D for people with knee and/or hip OA.   - Quality of evidence: Low (knee), Very low (hip) - Strength of recommendation: Conditional against recommendation | |  |
| We suggest not offering omega-3 fatty acids for people with knee and/or hip OA.   - Quality of evidence: Very low - Strength of recommendation: Conditional against recommendation | |  |
| We suggest not offering diacerein for people with knee and/or hip OA   - Quality of evidence: Very low - Strength of recommendation: Conditional against recommendation | |  |

**NICE (22)**

| **Characteristics** | | |
| --- | --- | --- |
| Year | 2020 | |
| Country | UK | |
| Target population | OA patients | |
| LOE Scale | 1. Assignment of a quality rating based on the study design: RCTs start HIGH and observational studies as LOW, uncontrolled case series as LOW or VERY LOW. 2. Downgrading of the rating for specified criteria:  Study limitations, inconsistency, indirectness, imprecision and reporting bias. Observational studies were upgraded if there was:  a large magnitude of effect, dose-response gradient, and if all plausible confounding would reduce a demonstrated effect or suggest a spurious effect when results showed no effect.  Each quality element considered to have “serious” or “very serious” risk of bias was rated down -1 or -2 points respectively. 3. Downgraded/upgraded marks were then summed and the overall quality rating was revised. (e.g. all RCTs started as HIGH and the overall quality became MODERATE, LOW or VERY LOW if 1, 2 or 3 points were deducted respectively. | |
| SOR Scale | - Interventions that must (or must not) be used: use of 'must' or 'must not' only if there is a legal duty to apply the recommendation. Occasionally use 'must' (or 'must not') if the consequences of not following the recommendation could be extremely serious or potentially life threatening. - Interventions that should (or should not) be used – a 'strong' recommendation: use 'offer' (and similar words such as 'refer' or 'advise') when there is confidence that, for the vast majority of patients, an intervention will do more good than harm, and be cost effective; use similar forms of words (for example, 'Do not offer…') when there is confidence that an intervention will not be of benefit for most patients. - Interventions that could be used: use 'consider' when there is confidence that an intervention will do more good than harm for most patients, and be cost effective, but other options may be similarly cost effective. The choice of intervention, and whether or not to have the intervention at all, is more likely to depend on the patient's values and preferences than for a strong recommendation, and so the healthcare professional should spend more time considering and discussing the options with the patient. | |
| **Recommendations (43)** | | **Ambulatory health intervention before TJR quantifiable in routine data from German SHIs** |
| Diagnose osteoarthritis clinically without investigations if a person:   - is 45 or over and - has activity-related joint pain and - has either no morning joint-related stiffness or morning stiffness that lasts no longer than 30 minutes. [new 2014] - Level of evidence: moderate/low - Strength of recommendation: not reported | | Physician visit for musculoskeletal disorders |
| Be aware that atypical features, such as a history of trauma, prolonged morning joint-related stiffness, rapid worsening of symptoms or the presence of a hot swollen joint, may indicate alternative or additional diagnoses. Important differential diagnoses include gout, other inflammatory arthritides (for example, rheumatoid arthritis), septic arthritis and malignancy (bone pain). [new 2014]   - Level of evidence: moderate/low - Strength of recommendation: not reported | | Physician visit for musculoskeletal disorders |
| Assess the effect of osteoarthritis on the person's function, quality of life, occupation, mood, relationships and leisure activities. Use figure 1 as an aid to prompt questions that should be asked as part of the holistic assessment of a person with osteoarthritis. [2008]   - Level of evidence: not reported - Strength of recommendation: not reported | | Physician visit for musculoskeletal disorders |
| Agree a plan with the person (and their family members or carers as appropriate) for managing their osteoarthritis. Apply the principles in Patient experience in adult NHS services (NICE clinical guidance 138) in relation to shared decision-making. [new 2014]   - Level of evidence: moderate - Strength of recommendation: not reported | |  |
| Take into account comorbidities that compound the effect of osteoarthritis when formulating the management plan. [2008]   - Level of evidence: not reported - Strength of recommendation: not reported | |  |
| Discuss the risks and benefits of treatment options with the person, taking into account comorbidities. Ensure that the information provided can be understood. [2008]   - Level of evidence: not reported - Strength of recommendation: not reported | |  |
| Offer advice on the following core treatments to all people with clinical osteoarthritis.   - Access to appropriate information - Activity and exercise - Interventions to achieve weight loss if the person is overweight or obese [2008, amended 2014] - Level of evidence: not reported - Strength of recommendation: recommended for the vast majority of patients | |  |
| Offer accurate verbal and written information to all people with osteoarthritis to enhance understanding of the condition and its management, and to counter misconceptions, such as that it inevitably progresses and cannot be treated. Ensure that information sharing is an ongoing, integral part of the management plan rather than a single event at time of presentation. [2008]   - Level of evidence: not reported - Strength of recommendation: recommended for the vast majority of patients | |  |
| Agree individualised self-management strategies with the person with osteoarthritis. Ensure that positive behavioural changes, such as exercise, weight loss, use of suitable footwear and pacing, are appropriately targeted. [2008]   - Level of evidence: not reported - Strength of recommendation: not reported | |  |
| Ensure that self-management programmes for people with osteoarthritis, either individually or in groups, emphasise the recommended core treatments (see recommendation 1.2.5), especially exercise. [2008]   - Level of evidence: not reported - Strength of recommendation: not reported | |  |
| The use of local heat or cold should be considered as an adjunct to core treatments. [2008]   - Level of evidence: not reported - Strength of recommendation: strong | |  |
| Advise people with osteoarthritis to exercise as a core treatment (see recommendation 1.2.5), irrespective of age, comorbidity, pain severity or disability. Exercise should include:   - - local muscle strengthening and   - general aerobic fitness.   It has not been specified whether exercise should be provided by the NHS or whether the healthcare professional should provide advice and encouragement to the person to obtain and carry out the intervention themselves. Exercise has been found to be beneficial but the clinician needs to make a judgement in each case on how to effectively ensure participation. This will depend upon the person's individual needs, circumstances and self-motivation, and the availability of local facilities. [2008]   - Level of evidence: not reported - Strength of recommendation: recommended for the vast majority of patients | | Exercise therapy/  referral to PT |
| Manipulation and stretching should be considered as an adjunct to core treatments, particularly for osteoarthritis of the hip. [2008]   - Level of evidence: not reported - Strength of recommendation: strong | |  |
| Offer interventions to achieve weight loss as a core treatment (see recommendation 1.2.5) for people who are obese or overweight. [2008]   - Level of evidence: not reported - Strength of recommendation: recommended for the vast majority of patients | |  |
| Healthcare professionals should consider the use of transcutaneous electrical nerve stimulation (TENS) as an adjunct to core treatments for pain relief. [2008]   - Level of evidence: not reported - Strength of recommendation: strong | | TENS |
| Do not offer glucosamine or chondroitin products for the management of osteoarthritis. [2014]   - Level of evidence: low/very low - Strength of recommendation: recommended for the vast majority of patients | |  |
| Do not offer acupuncture for the management of osteoarthritis. [2014]   - Level of evidence: low/very low - Strength of recommendation: recommended for the vast majority of patients | | Acupuncture |
| Offer advice on appropriate footwear (including shock-absorbing properties) as part of core treatments (see recommendation 1.2.5) for people with lower limb osteoarthritis. [2008]   - Level of evidence: not reported - Strength of recommendation: recommended for the vast majority of patients | | Shoes/insoles |
| People with osteoarthritis who have biomechanical joint pain or instability should be considered for assessment for bracing/joint supports/insoles as an adjunct to their core treatments. [2008]   - Level of evidence: not reported - Strength of recommendation: strong | | Orthoses/braces;  Shoes/insoles |
| Assistive devices (for example, walking sticks and tap turners) should be considered as adjuncts to core treatments for people with osteoarthritis who have specific problems with activities of daily living. If needed, seek expert advice in this context (for example, from occupational therapists or Disability Equipment Assessment Centres). [2008]   - Level of evidence: not reported - Strength of recommendation: strong | | Walking aids |
| Do not refer for arthroscopic lavage and debridement as part of treatment for Osteoarthritis, unless the person has knee osteoarthritis with a clear history of mechanical locking (as opposed to morning joint stiffness, 'giving way' or X-ray evidence of loose bodies). [2008, amended 2014]   - Level of evidence: not reported - Strength of recommendation: recommended for the vast majority of patients | |  |
| Healthcare professionals should consider offering paracetamol for pain relief in addition to core treatments (see recommendation 1.2.5); regular dosing may be required. Paracetamol and/or topical non-steroidal anti-inflammatory drugs (NSAIDs) should be considered ahead of oral NSAIDs, cyclo-oxygenase 2 (COX-2) inhibitors or opioids. [2008]   - Level of evidence: not reported - Strength of recommendation: strong | | Paracetamol (initial medication) |
| If paracetamol or topical NSAIDs are insufficient for pain relief for people with osteoarthritis, then the addition of opioid analgesics should be considered. Risks and benefits should be considered, particularly in older people. [2008]   - Level of evidence: not reported - Strength of recommendation: strong | | Weak opioids |
| Consider topical NSAIDs for pain relief in addition to core treatments (see recommendation 1.2.5) for people with knee or hand osteoarthritis. Consider topical NSAIDs and/or paracetamol ahead of oral NSAIDs, COX-2 inhibitors or opioids. [2008]   - Level of evidence: not reported - Strength of recommendation: not reported | | Topical NSAID |
| Topical capsaicin should be considered as an adjunct to core treatments for knee or hand osteoarthritis. [2008]   - Level of evidence: not reported - Strength of recommendation: strong | |  |
| Do not offer rubefacients for treating osteoarthritis. [2008]   - Level of evidence: not reported - Strength of recommendation: recommended for the vast majority of patients | |  |
| Where paracetamol or topical NSAIDs are ineffective for pain relief for people with osteoarthritis, then substitution with an oral NSAID/COX-2 inhibitor should be considered. [2008]   - Level of evidence: not reported - Strength of recommendation: strong | | Oral NSAID |
| Where paracetamol or topical NSAIDs provide insufficient pain relief for people with osteoarthritis, then the addition of an oral NSAID/COX-2 inhibitor to paracetamol should be considered. [2008]   - Level of evidence: not reported - Strength of recommendation: strong | | Oral NSAID |
| Use oral NSAIDs/COX-2 inhibitors at the lowest effective dose for the shortest possible period of time. [2008]   - Level of evidence: not reported - Strength of recommendation: not reported | | Oral NSAID |
| When offering treatment with an oral NSAID/COX-2 inhibitor, the first choice should be either a standard NSAID or a COX-2 inhibitor (other than etoricoxib 60 mg). In either case, co-prescribe with a proton pump inhibitor (PPI), choosing the one with the lowest acquisition cost. [2008]   - Level of evidence: not reported - Strength of recommendation: strong | | Oral NSAID treatment with concomitant PPI/misoprostol in case of Gl risk factors |
| All oral NSAIDs/COX-2 inhibitors have analgesic effects of a similar magnitude but vary in their potential gastrointestinal, liver and cardio-renal toxicity; therefore, when choosing the agent and dose, take into account individual patient risk factors, including age. When prescribing these drugs, consideration should be given to appropriate assessment and/or ongoing monitoring of these risk factors. [2008]   - Level of evidence: not reported - Strength of recommendation: strong | | Oral NSAID treatment with caution for comorbidities (GI/CV/renal) |
| If a person with osteoarthritis needs to take low-dose aspirin, healthcare professionals should consider other analgesics before substituting or adding an NSAID or COX-2 inhibitor (with a PPI) if pain relief is ineffective or insufficient. [2008]   - Level of evidence: not reported - Strength of recommendation: strong | |  |
| Intra-articular corticosteroid injections should be considered as an adjunct to core treatments for the relief of moderate to severe pain in people with osteoarthritis. [2008]   - Level of evidence: not reported - Strength of recommendation: strong | | Corticosteroid injection |
| Do not offer intra-articular hyaluronan injections for the management of osteoarthritis. [2014]   - Level of evidence: low/very low - Strength of recommendation: recommended for the vast majority of patients | |  |
| Clinicians with responsibility for referring a person with osteoarthritis for consideration of joint surgery should ensure that the person has been offered at least the core (non-surgical) treatment options (see recommendation 1.2.5). [2008]   - Level of evidence: not reported - Strength of recommendation: not reported | | Combination of pharmacological and non-pharmacological therapies (before TJR) |
| When discussing the possibility of joint surgery, check that the person has been offered at least the core treatments for osteoarthritis (see recommendation 1.2.5), and give them information about:   - - the benefits and risks of surgery and the potential consequences of not having surgery   - recovery and rehabilitation after surgery• how having a prosthesis might affect them   - how care pathways are organised in their local area. [new 2014] - Level of evidence: not reported - Strength of recommendation: not reported | | Combination of pharmacological and non-pharmacological therapies (before TJR) |
| Base decisions on referral thresholds on discussions between patient representatives, referring clinicians and surgeons, rather than using scoring tools for prioritisation. [2008, amended 2014]   - Level of evidence: not reported - Strength of recommendation: not reported | |  |
| Consider referral for joint surgery for people with osteoarthritis who experience joint symptoms (pain, stiffness and reduced function) that have a substantial impact on their quality of life and are refractory to non-surgical treatment. [2008, amended 2014]   - Level of evidence: not reported - Strength of recommendation: not reported | | Referral to orthopaedic surgeon if conservative therapy failed |
| Refer for consideration of joint surgery before there is prolonged and established functional limitation and severe pain. [2008, amended 2014]   - Level of evidence: not reported - Strength of recommendation: not reported | |  |
| Patient-specific factors (including age, sex, smoking, obesity and comorbidities) should not be barriers to referral for joint surgery. [2008, amended 2014]   - Level of evidence: not reported - Strength of recommendation: not reported | |  |
| Offer regular reviews to all people with symptomatic osteoarthritis. Agree the timing of the reviews with the person (see also recommendation 1.7.2). Reviews should include:   - monitoring the person's symptoms and the ongoing impact of the condition on their everyday activities and quality of life - monitoring the long-term course of the condition - discussing the person's knowledge of the condition, any concerns they have, their personal preferences and their ability to access services - reviewing the effectiveness and tolerability of all treatments - support for self-management. [new 2014] - Level of evidence: moderate/very low - Strength of recommendation: recommended for the vast majority of patients | |  |
| Consider an annual review for any person with one or more of the following:   - troublesome joint pain - more than one joint with symptoms - more than one comorbidity - taking regular medication for their osteoarthritis. [new 2014] - Level of evidence: moderate/very low - Strength of recommendation: not reported | | Physician visit for musculoskeletal disorders |
| Apply the principles in Patient experience in adult NHS services (NICE clinical guidance 138) with regard to an individualised approach to healthcare services and patient views and preferences. [new 2014]   - Level of evidence: moderate/very low - Strength of recommendation: not reported | |  |

**OARSI (23)**

| **Characteristics** | | |
| --- | --- | --- |
| Year | 2019 | |
| Country | not specified (international) | |
| Target population | KOA/HOA/hand OA patients | |
| LOE Scale | - High - Moderate - Low - Very Low | |
| SOR Scale | Expert Consensus (n=13): Core Treatment selections were designated as “strong recommendations in favor” by default. Level designations based on percentage of votes “in favor” and strength of recommendation:   - Level 1A (“in favor” (75-100%), “against” (0-25%)): >50% strong - Level 1B (“in favor” (75-100%), “against” (0-25%)): >50% conditional - Level 2 (“in favor” (60-74%), “against” (26-40%)): >50% conditional by default - Level 3 (“in favor” (41-59%), “against” (41-59%)): >50% conditional by default - Level 4B (“in favor” (26-40%), “against” (60-74%)): >50% conditional by default - Level 4A (“in favor” (0-25%), “against” (75-100%)): >50% conditional - Level 5 (“in favor” (0-25%), “against” (75-100%)): >50% strong | |
| **Recommendations (22)** | | **Ambulatory health intervention before TJR quantifiable in routine data from German SHIs** |
| Core Treatments (treatments deemed appropriate for use by the majority of patients in nearly any scenario and deemed safe for use in conjunction with first line and second line treatments):  Structured land-based exercise programs, dietary weight management in combination with exercise, and mind-body exercise (such as Tai Chi and Yoga) were considered by the panel to be effective and safe for all patients with Knee OA, regardless of comorbidity. These treatments are recommended for use alone or along with interventions of any recommendation level, as deemed appropriate for the individual. | | Exercise therapy/  referral to PT |
| Core Treatments (treatments deemed appropriate for use by the majority of patients in nearly any scenario and deemed safe for use in conjunction with first line and second line treatments):  For patients with Hip OA, only structured land-based exercise programs were considered eligible for Core Treatment designation. Arthritis education was, again, considered a standard of care. | | Exercise therapy/  referral to PT |
| Aquatic exercise, gait aids, cognitive behavioral therapy with an exercise component, and self-management programs were the recommended non-pharmacologic options for individuals with Knee OA and no comorbidities, and for individuals with GI or CV comorbidities or with widespread pain disorders and/or depression. | |  |
| The use of gait aids was recommended in patients from each comorbidity subgroup, with the exception of patients with widespread pain and/or depression. | | Walking aids |
| Core Treatments (treatments deemed appropriate for use by the majority of patients in nearly any scenario and deemed safe for use in conjunction with first line and second line treatments):  Education about OA is considered a standard of care. | |  |
| Topical non-steroidal anti-inflammatory drugs (NSAIDs) were strongly recommended for use in Knee OA patients with no comorbidities. | | Topical NSAID |
| Topical NSAIDs were also strongly recommended for Knee OA patients with GI or CV comorbidities and for patients with frailty (…). | | Topical NSAID |
| No interventions were strongly recommended for use for individuals with Knee OA with concomitant widespread pain disorders (e.g., fibromyalgia) and/or depression. | |  |
| Aquatic exercise was not recommended for patients who suffered from frailty due to potential risk of accidental injury. | |  |
| Use of Oral NSAIDs was conditionally recommended for individuals with Knee OA who do not have comorbid conditions. | | Oral NSAID |
| Use of oral NSAIDs was conditionally recommended for Hip OA patients without comorbidities and for patients with widespread pain and/or depression. In both treatment profiles, non-selective NSAIDs preferably with the addition of a PPI, and selective COX-2 inhibitors were conditionally recommended. | | Oral NSAID |
| For individuals with GI comorbidities, selective COX-2 inhibitors and non-selective NSAIDs in combination with a PPI were conditionally recommended due to their benefits on pain and functional outcomes. | | Oral NSAID treatment with concomitant PPI/misoprostol in case of Gl risk factors |
| In both treatment profiles, non-selective NSAIDs preferably with the addition of a PPI, and selective COX-2 inhibitors were conditionally recommended. | |  |
| For patients with GI comorbidities, the use of oral NSAIDs was restricted to selective COX-2 inhibitors or non-selective NSAIDs in combination with a PPI. Though no pharmacologic treatment option was conditionally recommended for Hip OA patients with comorbid CV conditions or frailty, a Good Clinical Practice Statement was made specifying that NSAIDs with more favorable safety profiles may be used in high risk patients (including patients with frailty) at the lowest possible dose, for the shortest possible treatment duration, for symptomatic relief. | |  |
| NSAIDs of any class were not recommended for patients with CV comorbidities due to evidence associating NSAID use with heightened CV risk. | | Oral NSAID treatment with caution for comorbidities (GI/CV/renal) |
| NSAIDs were not recommended in patients with frailty. | |  |
| The use of intra-articular corticosteroids (IACS) and hyaluronan (IAHA) were conditionally recommended in individuals with knee OA in all groups. | | Corticosteroid injection |
| Conditionally recommended treatments for patients with widespread pain and/or depression included oral NSAIDs of any category, duloxetine, IACS, IAHA and topical NSAIDs. | | Duloxetine |
| Despite a lack of direct evidence, mind-body exercise (Tai Chi or Yoga) was conditionally recommended for Hip OA patients in all comorbidity subgroups because its favorable efficacy and safety profile in patients with Knee OA was considered generalizable to Hip OA. | |  |
| Self-management programs were also conditionally recommended for patients in all comorbidity subgroups. | |  |
| Cognitive behavioral therapy was only recommended for patients with widespread pain and/or depression. | |  |
| Dietary weight management was not recommended for Hip OA individuals of any comorbidity subgroup because of lack of direct evidence for its effectiveness specifically for symptoms of Hip OA. (…) weight management may be recommended for certain individuals (e.g., individuals presenting with body mass index ≥30 kg/m^2^) of any comorbidity subgroup as a part of a healthy lifestyle regimen. | |  |

**Ottawa Panel (24)**

| **Characteristics** | | |
| --- | --- | --- |
| Year | 2017 | |
| Country | Canada | |
| Target population | KOA patients (exercise therapy) | |
| LOE Scale | Hierarchical grading system considering clinical importance, stastical significance and study design:   - A (strongly recommended): clinical importance: ≥15%; stastical significance: <.05; study design: RCT (single or meta-analysis) - B (recommended): clinical importance: ≥15%; stastical significance: <.05; study design: CCT or observational (single or meta-analysis) - C+ (suggested used): clinical importance: ≥15%; stastical significance: not significant; study design: RCT/CCT or observational (single or meta-analysis) - C (neutral): clinical importance: <15%; stastical significance: unimportant; study design: any study design - D (neutral): clinical importance: <15% (favors control); stastical significance: unimportant; study design: any study design - D+ (suggested no use): clinical importance: ≥15% (favors control); stastical significance: not significant; study design: RCT/CCT or observational (single or meta-analysis) - D- (strongly not recommended): clinical importance: ≥15% (favors control); stastical significance: <.05 (favors control); study design: well-designed RCT with >100 patients (if <100 patients, becomes grade D) | |
| SOR Scale |  |  |
| **Recommendations (26)** | | **Ambulatory health intervention before TJR quantifiable in routine data from German SHIs** |
| Home-based progressive strengthening exercise program:  A four-month home-based progressive strengthening exercise program (isotonic and resistance exercises of hips and knees with ankle weights and functional strengthening) (three days per week) for knee osteoarthritis management for pain relief (Western Ontario and McMaster Universities Arthritis Index (WOMAC) subscale) and improved physical function (WOMAC subscale) at four months end of treatment is recommended. There is a neutral improvement for quality of life (Short Form 36 (SF-36) SF-36 mental health component subscale (MCS) subscale) at end of treatment of four months. | |  |
| Progressive hip muscle strengthening home-based exercise program:  A 12-week progressive hip muscle strengthening home-based exercise program (isotonic resisted with ankle weights or therapeutic elastic bands) (five days per week at home plus seven physiotherapy consultations of 15–30 minutes during two months) for medial knee osteoarthritis management for pain relief (WOMAC subscale) and improved physical function (WOMAC subscale) at the one-week follow-up is recommended. | |  |
| Group education program followed by an unsupervised home-based exercise program:  A four-week group education program followed by eight weeks of the unsupervised home-based exercise program (active range-of-motion exercises, muscle strengthening and muscle stretching) (45minutes one day per week) for the management of knee osteoarthritis for pain relief (WOMAC subscale) and improved physical function (WOMAC subscale) after those eight weeks of home exercises is recommended. There is a neutral improvement for pain relief (WOMAC subscale) at end of treatment of four weeks. The Ottawa Panel does not suggest physical function (WOMAC subscale) at end of treatment of four weeks | |  |
| Isokinetic strengthening exercise program:  A eight-week isokinetic strengthening exercise program (including heat; passive knee Range of Motion (ROM) concentric and eccentric exercises) (three times weekly) for the management of knee osteoarthritis for pain relief (Visual Analogue Scale (VAS))54 and improved physical function (Lequesne Index (LI) subscale) at eight weeks end of treatment and at six-months follow-up is recommended. | |  |
| Physiotherapy intervention program:  A three-month physiotherapy intervention program (manual therapy; patellar taping; osteoarthritis education; functional and strengthening exercises for the quadriceps and hip muscles) (60minutes once a week for four weeks followed by once bi-weekly for eight weeks for each group) for patella-femoral knee osteoarthritis management for pain relief during motion (VAS) at three months end of treatment is recommended. The use of this program is also suggested for pain relief during motion (VAS) at the six-month follow-up. There is a neutral improvement for pain relief (Knee injury and Osteoarthritis Outcome Score (KOOS) pain subscale) and physical function (KOOS in daily living subscale) at end of treatment of three months and follow-up six months. There is also a neutral improvement for quality of life (KOOS quality of life subscale) at three months end of treatment and six-months follow-up. | |  |
| Osteoarthritis education and supervised strengthening exercise program with home exercises:  A six-week osteoarthritis education and supervised strengthening exercise program with home exercises (resistance exercises for the knee and hip independently of the site of major pain) (one two-hour session per week) for the management of adults with knee osteoarthritis for pain relief (VAS) at the six-month follow-up is suggested. There is a neutral improvement for pain relief (VAS), quality of life (VAS subscale) and quality of life (Quality of life scale) at six weeks end of treatment. There is also a neutral improvement for quality of life (Quality of life scale) and Quality of life (VAS) at six-months follow-up. | | Exercise therapy/  referral to PT |
| Supervised isokinetic, isotonic and isometric muscle strengthening exercise programs:  An eight-week supervised isokinetic, isotonic or isometric muscle strengthening exercise programs (warm-up stationary bike; ROM assessment in each arthritic joint; stretching; hot packs) (three days per week) for the management of bilateral knee osteoarthritis for pain relief (VAS) and for improved physical function (LI subscale) at eight weeks end of treatment and at one-year follow-up is strongly recommended. | |  |
| Supervised isokinetic muscle strengthening exercise program and hot packs application:  An eight-week supervised isokinetic muscle strengthening exercise program and hot packs application (concentric and eccentric exercises; warm-up stationary bike; ROM assessment in each arthritic joint; stretching; hot packs) (three days per week) for the management of knee osteoarthritis for pain relief (VAS) and improved physical function (LI subscale) at the one-year follow-up is recommended. There is a neutral improvement for pain relief (VAS) and physical function (LI disability subscale) at end of treatment of eight weeks. | |  |
| Group-based supervised progressive strengthening and coordination exercise program:  A 12-week group-based supervised progressive strengthening and coordination exercise program (circuit training) (one hour sessions three days per week) for the management of knee osteoarthritis for improved quality of life (KOOS quality of life subscale) at 12 weeks end of treatment is suggested. There is a neutral improvement for pain relief (KOOS pain subscale) and physical function (KOOS in daily living subscale) at end of treatment of 12 weeks. | |  |
| Physiotherapy exercise interventions:  A three-month physiotherapy exercise interventions (mobility training, venous therapy, lower extremity and trunk muscle strengthening, flexibility, coordination and balance exercises) (twice weekly for three months) for the management of knee osteoarthritis for pain relief at night (VAS) at nine-months follow-up is recommended. Also, the Ottawa Panel recommends the intervention for pain relief weight-bearing (VAS) and pain relief at rest (VAS) at 12-weeks end of treatment as well as at nine-months follow-up. There is a neutral improvement for pain relief (pain at night VAS) and physical function (algofunctional index (AFI) subscale) at end of treatment of three months. There is also a neutral improvement for physical function (AFI subscale) at nine-months follow-up and for physical function (usual walking speed) at three-months end of treatment and nine-months follow-up. | |  |
| Physiotherapy exercise interventions:  A three-month physiotherapy exercise interventions (mobility training, venous therapy, lower extremity and trunk muscle strengthening, flexibility, coordination and balance exercises) (twice weekly for three months) for the management of knee osteoarthritis for pain relief at night (VAS) at nine-months follow-up is recommended. Also, the Ottawa Panel recommends the intervention for pain relief weight-bearing (VAS) and pain relief at rest (VAS) at 12-weeks end of treatment as well as at nine-months follow-up. There is a neutral improvement for pain relief (pain at night VAS) and physical function (algofunctional index (AFI) subscale) at end of treatment of three months. There is also a neutral improvement for physical function (AFI subscale) at nine-months follow-up and for physical function (usual walking speed) at three-months end of treatment and nine-months follow-up. | |  |
| Progressive supervised squat exercise program:  A 12-week progressive supervised squat exercise program (bicycle warm-up; squat exercises with/without whole body vibration) (three days per week on alternate days) for the management of knee osteoarthritis for pain relief (WOMAC subscale) and improved physical function (WOMAC subscale) at 12 weeks end of treatment is suggested. There is a neutral improvement for physical function (6MWT) at end of treatment of 12 weeks. | |  |
| Progressive quadriceps strengthening exercise program with nonsteroidal anti-inflammatory drugs:  A eight-week progressive quadriceps strengthening exercise program with Nonsteroidal anti-inflammatory drugs (NSAIDs) (quadriceps exercises while sitting on a chair or in a supine position using ankle weights) for the management of knee osteoarthritis for pain relief (VAS) at eight weeks end of treatment is suggested. There is a neutral improvement for physical function (WOMAC global score) at end of treatment of eight weeks. | |  |
| Progressive exercise program, education and usual care:  A six-week progressive exercise program (warm-up, strengthening, balance and motor control exercises), education and usual care (45 minutes, two times per week), either in a group or individually, for the management of knee osteoarthritis for improved physical function (WOMAC subscale) at the end of treatment of six weeks is strongly recommended. There is a neutral improvement for pain relief (WOMAC subscale) and physical function (WOMAC subscale) at six-months follow-up. | |  |
| High and low-resistance strengthening exercise programs:  An eight-week high or low-resistance strengthening exercise program (10% and 60% 1-RM leg training) (both high and low-resistance groups completed it three times weekly) for the management of knee osteoarthritis for pain relief (WOMAC subscale) and improved physical function (WOMAC subscale)52 at eight weeks end of treatment is strongly recommended. | |  |
| Non-weight-bearing and weight-bearing exercise programs:  An eight-week progressive non-weight-bearing or weight-bearing exercise program (including heat and ROM before; eccentric contraction during lower-extremity flexion/extension; ice after) (three days per week) for the management of knee osteoarthritis for improved physical function (WOMAC subscale) at eight weeks end of treatment is strongly recommended. | |  |
| Quadriceps strengthening exercise program:  A 24-month quadriceps strengthening exercise program (with graded therapeutic elastic bands exercises; flexibility exercises; functional strengthening exercises; stretching exercises (twice daily) plus a dietary intervention) for the management of overweight adults with knee osteoarthritis for pain relief (WOMAC subscale) at 24 months end of treatment is strongly recommended. | |  |
| Progressive resistance exercise program of knees and hip muscles:  A 12-week progressive resistance exercise program of knees and hip muscles (with machines and free weights) (two days per week) for the management of women with knee osteoarthritis for pain relief (VAS), pain relief (WOMAC subscale), improved physical function (WOMAC subscale), improved physical function (Short Form 36 (SF-36) subscale) and improved quality of life (SF-36 subscale) at 90 days end of treatment is recommended. There is a neutral improvement for physical function (Six Minute Walk Test (6MWT)) at end of treatment of 90 days. | |  |
| Strengthening and balance exercise program:  A three-month strengthening (all Lower Extremity (LE) muscles with ankle weights, therapeutic elastic bands, non-weight-bearing and weight-bearing) and balance (gait training) exercise program (twice weekly) for knee osteoarthritis management for improved physical function (usual walking speed) at three months end of treatment is recommended. The use of the program is also suggested for pain relief (VAS) at three months end of treatment | |  |
| Home-based physiotherapist prescribed supervised quadriceps strengthening exercise program:  A 12-week home-based physiotherapist prescribed supervised quadriceps strengthening exercise program (using ankle weights, therapeutic elastic bands) (five days per week) for the management of knee osteoarthritis for pain relief (WOMAC subscale) at 12 weeks end of treatment is strongly recommended. There is a neutral improvement for physical function (WOMAC subscale) at end of treatment of 12 weeks. | |  |
| Concentric-eccentric quadriceps strengthening exercise program:  A eight-week concentric-eccentric quadriceps strengthening exercise program (concentric quadriceps action followed by an eccentric quadricep action) (50 minute classes three times a week) for the management of knee osteoarthritis for pain relief (WOMAC subscale) and improved physical function (WOMAC subscale) at eight weeks end of treatment is recommended. | |  |
| Lower extremity strengthening exercise program:  A six-month lower extremity strengthening exercise program (isometric, isotonic and dynamic exercises) (one set of exercises performed daily) for the management of knee osteoarthritis for improved quality of life (Hospital Anxiety and Depression Scale (HADS) subscale) at six months end of treatment is strongly recommended. The Ottawa Panel also suggests the intervention for pain relief (WOMAC subscale) as well as improved physical function (WOMAC subscale)at six-months end of treatment. | |  |
| Mechanical diagnosis and therapy exercise program:  A two-week mechanical diagnosis and therapy exercise program (end-range exercises; advice on exercises for aerobic as well strengthening of the quadriceps; biking; walking) (10 repetitions every 2–3hours) for the management of knee osteoarthritis for pain relief (P4 subscale), pain relief (KOOS pain subscale) and improved physical function (KOOS in daily living subscale) at two weeks end of treatment is strongly recommended. There is a neutral improvement for pain relief (P4 subscale), pain relief (KOOS pain subscale) and physical function (KOOS in daily living subscale) at 10-weeks follow-up. | |  |
| Concentric-eccentric isokinetic and isometric exercise programs + paracetamol:  A eight-week concentric-eccentric isokinetic or isometric exercise programs + paracetamol (cycling warm-up before the exercises; application of isokinetic dynamometer for exercises; cool-down after the exercises) (three days weekly) for the management of knee osteoarthritis for pain relief during rest (VAS), pain relief during motion (VAS), improved physical function (WOMAC subscale) and improved quality of life (SF-36 subscale) at eight-weeks end of treatment and at 12-weeks follow-up is recommended. | |  |
| Strengthening exercise program with patient education:  A eight-week strengthening exercise program (stretching, strength, mobility, functional, balance, relaxation) with patient education (60 minute sessions completed twice weekly) for the management of adults with knee osteoarthritis for pain relief (LI subscale) and suggests its use for improved physical function (LI subscale) at eight weeks end of treatment is recommended. There is a neutral improvement for physical function (6MWT) and quality of life (SF-36 MCS) at end of treatment of eight weeks. | |  |
| Strengthening exercise program with home exercises and usual care, including patient education and medication if necessary:  A 12-week strengthening exercise program with home exercises and usual care, including patient education and medication if necessary (exercises for muscle functions, mobility and coordination and instructions) (one to three days per week) for the management of elderly individuals with knee osteoarthritis for pain relief (VAS) at 12 weeks end of treatment is suggested. There is a neutral improvement for physical function (self-reported disability IRGL) at end of treatment of 12 weeks. | |  |

**Ottawa Panel (25)**

| **Characteristics** | | |
| --- | --- | --- |
| Year | 2017 | |
| Country | Canada | |
| Target population | KOA patients (exercise therapy) | |
| LOE Scale | Hierarchical grading system considering clinical importance, stastical significance and study design:   - A (strongly recommended): clinical importance: ≥15%; stastical significance: <.05; study design: RCT (single or meta-analysis) - B (recommended): clinical importance: ≥15%; stastical significance: <.05; study design: CCT or observational (single or meta-analysis) - C+ (suggested used): clinical importance: ≥15%; stastical significance: not significant; study design: RCT/CCT or observational (single or meta-analysis) - C (neutral): clinical importance: <15%; stastical significance: unimportant; study design: any study design - D (neutral): clinical importance: <15% (favors control); stastical significance: unimportant; study design: any study design - D+ (suggested no use): clinical importance: ≥15% (favors control); stastical significance: not significant; study design: RCT/CCT or observational (single or meta-analysis) - D- (strongly not recommended): clinical importance: ≥15% (favors control); stastical significance: <.05 (favors control); study design: well-designed RCT with >100 patients (if <100 patients, becomes grade D) | |
| SOR Scale |  |  |
| **Recommendations (5)** | | **Ambulatory health intervention before TJR quantifiable in routine data from German SHIs** |
| Leg functional aerobic and strengthening exercise program:  A four-week leg functional aerobic and strengthening exercise program (supervised exercise: riding a stationary bike, active range of motion for the knee, muscle strengthening exercises for the hip and knee, muscle stretching and manual physical therapy) (two 30-minute sessions per week) for the management of knee osteoarthritis for improved physical function (WOMAC subscale) at end of treatment of four weeks and at the four weeks follow-up is recommended. There is a neutral improvement for physical function (6MWT) at end of treatment of four weeks and four weeks follow-up. | |  |
| Individual and group supervised aerobic and strengthening exercise programs:  An eight-week individual or group supervised aerobic and strengthening exercise programs (running, eccentric and concentric exercises, stairs, stepper machine, home exercise program) (at the therapist discretion or one hour two times per week) for the management of knee osteoarthritis for pain relief (WOMAC subscale) at the end of treatment of eight weeks is strongly recommended. There is a neutral improvement for quality of life (SF-36 subscale) and physical function (WOMAC subscale) at end of treatment of eight weeks. | | Exercise therapy/  referral to PT |
| Community physiotherapy exercise interventions:  A 10-week community physiotherapy exercise interventions (an individualised aerobic and strengthening exercise program and advice leaflet about activity and pacing) (20 minutes, three-six times over 10 weeks) for the management of knee osteoarthritis for pain relief (WOMAC subscale) and improved physical function (WOMAC subscale) at the three months follow-up is strongly recommended. There is a neutral improvement for pain relief (WOMAC subscale) and physical function (WOMAC subscale) at the three and nine-month follow-up measures. | |  |
| Aerobic, strengthening exercise program and osteoarthritis health education:  A three-month aerobic, strengthening exercise program and osteoarthritis health education (brisk walking, isometric and isotonic muscle strengthening with therapeutic elastic bands, stretching) (one hour sessions, three times per week) for the management of knee osteoarthritis for pain relief during weight-bearing activities (AIMS2 subscale) and improved physical function (AIMS2 subscale) at the end of treatment of three months is strongly recommended. | |  |
| Cycling exercise program:  A 12-week cycling exercise program (warm-up, aerobic loading, cool-down) (20-60-minute classes, two-six days per week) for the management of knee osteoarthritis for pain relief (WOMAC subscale) and improved physical function (WOMAC subscale) is recommended and is suggested for its use for improved quality of life (KOOS quality of life subscale) at end of treatment of 12 weeks. | |  |

**Ottawa Panel (26)**

| **Characteristics** | | |
| --- | --- | --- |
| Year | 2016 | |
| Country | Canada | |
| Target population | HOA patients (exercise therapy) | |
| LOE Scale | Hierarchical grading system considering clinical importance, stastical significance and study design:   - A (strongly recommended): clinical importance: ≥15%; stastical significance: <.05; study design: RCT (single or meta-analysis) - B (recommended): clinical importance: ≥15%; stastical significance: <.05; study design: CCT or observational (single or meta-analysis) - C+ (suggested used): clinical importance: ≥15%; stastical significance: not significant; study design: RCT/CCT or observational (single or meta-analysis) - C (neutral): clinical importance: <15%; stastical significance: unimportant; study design: any study design - D (neutral): clinical importance: <15% (favors control); stastical significance: unimportant; study design: any study design - D+ (suggested no use): clinical importance: ≥15% (favors control); stastical significance: not significant; study design: RCT/CCT or observational (single or meta-analysis) - D- (strongly not recommended): clinical importance: ≥15% (favors control); stastical significance: <.05 (favors control); study design: well-designed RCT with >100 patients (if <100 patients, becomes grade D) | |
| SOR Scale |  |  |
| **Recommendations (16)** | | **Ambulatory health intervention before TJR quantifiable in routine data from German SHIs** |
| Supervised group strength training with unsupervised home exercises versus control (GP care), level I RCT (n = 94, high quality [PEDro score 7/10]) (Tak et al., 2005):   - Grade A (clinically important benefit demonstrated with statistical significance) for: pain (observed, Harris Hip Score) at 8 weeks (end of treatment); for pain (subjective, 10 cm Visual Analogue Scale), and self-reported disability (Sickness Impact Profile) at 12 weeks (follow up post treatment). - Grade C+ (clinically important benefit demonstrated without statistical significance) for: self-reported disability (Sickness Impact Profile) at 8 weeks (end of treatment). - Grade C (no benefit demonstrated) for: pain (subjective, 10 cm Visual Analogue Scale), self-reported disability (physical function [Groningen Activity Restriction Scale score from 18-72]), hip function (Harris Hip Score), observed disability (walking 20m [sec]), timed up and go (sec), toe reaching (left + right), and health related quality of life at end of treatment 8 weeks; for: hip function (Harris Hip Score), observed disability (walking 20m [sec]), stairs (up & down), timed up and go (sec), toe reaching (left & right), health related quality of life, self-reported disability (physical function) [Groningen Activity Restriction Scale score from 18-72]), and pain (observed, Harris Hip Score) at 12 weeks (follow up post treatment). - Grade D (no benefit demonstrated but favouring control) for: stairs (up & down [sec]) at 8 weeks (end of treatment); for: quality of life (generic, [10 cm Visual Analogue Scale]) at 12 weeks (follow up post treatment). - Grade D+ (clinically important benefit demonstrated favouring control without statistical significance) for: quality of life (generic, [10 cm Visual Analogue Scale]) at 8 weeks (end of treatment). | | Exercise therapy/  referral to PT |
| Supervised group strength training and stretching exercises with GP-care versus control (GP care), level I RCT (n = 120, high quality [PEDro score 8/10]) (Juhakoski et al., 2011):   - Grade A (clinically important benefit demonstrated with statistical significance) for: physical function (WOMAC function [0-100 mm]) at 12 weeks (follow up post treatment). - Grade C (no benefit demonstrated) for: six minute walk (m), 10 m walk (sec), timed up and go (sec) at 12 weeks (end of treatment). - Grade C+ (clinically important benefit demonstrated without statistical significance) for: pain (WOMAC pain [0-100 mm]) at 12 weeks (end of treatment) - Grade D (no benefit demonstrated but favouring control) for: pain (WOMAC pain [0-100 mm]), and physical function (WOMAC physical function [0- 100 mm]) at 12 weeks (end of treatment). | |  |
| Supervised group low-load strength training and flexibility exercises versus control (waitlist), level I RCT (n = 131, high quality [PEDro score 8/10]) (French et al., 2013):   - Grade A (clinically important benefit demonstrated with statistical significance) for: pain with activity (Numerical rating scale), physical function (WOMAC physical function [0-68]), and aggregated range of motion (Range of Motion; degrees) at 8 weeks (end of treatment). - Grade C (no benefit demonstrated) for: general physical health status (SF-36 physical component summary [0-100]) and 50-foot walk (sec) at 8 weeks (end of treatment). - Grade D (no benefit demonstrated but favouring control) for: range of motion (FABER test) and sit-to-stand (sec) at 8 weeks (end of treatment). | |  |
| Supervised group strength training, functional, and flexibility exercises with patient education versus control (patient education), level I RCT (n = 109, high quality [PEDro score 8/10]) (Fernandes et al., 2010):   - Grade A (clinically important benefit demonstrated with statistical significance) for: physical function (WOMAC physical function [0-100]) at 24 weeks (follow-up post treatment). - Grade C+ (clinically important benefit demonstrated without statistical significance) for: pain (WOMAC pain [0-100]), and stiffness (WOMAC stiffness [0-100]) at 24 weeks (follow-up post treatment). - Grade C (no benefit demonstrated) for: pain (WOMAC pain [0-100]), physical function (WOMAC physical function [0-100]), stiffness (WOMAC stiffness [0-100]), physical function (SF-36), role physical (SF-36), and bodily pain (SF-36) at 12 weeks (end of treatment); for: physical function (SF-36), role physical (SF-36), and bodily pain (SF-36) at 24 weeks (follow-up post treatment). - Grade D (no benefit demonstrated but favouring control) for: general health (SF-36) at 12 weeks (end of treatment); for: general health (SF-36) at 24 weeks (follow-up post treatment) | |  |

**Ottawa Panel (27)**

| **Characteristics** | | |
| --- | --- | --- |
| Year | 2005 | |
| Country | Canada | |
| Target population | OA patients (exercise therapy) | |
| LOE Scale | - A: clinical importance: >15%; stastical significance: <.05; study design: RCT (single or meta-analysis) - B: clinical importance: >15%; stastical significance: <.05; study design: CCT or observational (single or meta-analysis), with a quality score of 3 or more on the 5-point Jadad methodologic quality checklist - C+: clinical importance: >15%; stastical significance: not significant; study design: RCT or CCT or observational (single or meta-analysis) - C: clinical importance: <15%; stastical significance: unimportant; study design: any study design - D: clinical importance: <0% (favors control); stastical significance: /; study design: well-designed RCT with >100 patients | |
| SOR Scale |  |  |
| **Recommendations (15)** | | **Ambulatory health intervention before TJR quantifiable in routine data from German SHIs** |
| Lower-extremity strengthening versus control, level 1 (RCT, n=345):  grade A for pain getting up from floor and functional status (clinically important benefit); grade C+ for pain during walking, pain while climbing stairs, functional tasks, and quadriceps femoris muscle peak torque (clinical benefit); grade C for stiffness, mobility, quadriceps femoris muscle force, muscle activation, and quality of life (no benefit).  Patients with a diagnosis of OA of the knee. | | Exercise therapy/  referral to PT |
| Lower-extremity isometric strengthening versus control, level 1 (RCT, n=102):  grade A for pain getting down to and up from floor (clinically important benefit); grade C+ for pain getting down and up stairs and timed functional tasks (clinical benefit); grade C for stiffness and functional status (no benefit). Patients with a diagnosis of OA of the knee. | |  |
| Isotonic resistance training versus isotonic combined with isokinetic (Kinetron*) resistance training for knee, level 1 (RCT, n=32):  grade C for quadriceps femoris muscle peak torque (no benefit). Patients with a primary diagnosis of OA of the knee. | |  |
| Isotonic combined with isokinetic resistance training for knee versus control, level 1 (RCT, n=32):  grade C for muscle force (no benefit). Patients with primary diagnosis of OA of the knee. | |  |
| Eccentric resistance training for knee versus control, level 1 (RCT, n=32):  grade C for muscle force (no benefit). Patients with primary diagnosis of OA of the knee. | |  |
| Concentric resistance training for knee versus control, level 1 (RCT, n=23):  grade A for pain at rest and during activities (clinically important benefit); grade C for global functional status (no benefit). Patients with knee OA bilaterally and grade II or III OA. | |  |
| Concentric-eccentric resistance training for knee versus control, level 1 (RCT, n=23):  grade A for pain at rest and during specific functional activities: 15-m walk and stair climbing/descending time (clinically important benefit). Patients with knee OA bilaterally and grade II or III OA. | |  |
| Home program strengthening for knee versus control, level 1 (CCT, n=81):  grade A for pain, functional status, energy level, and ROM in flexion (clinically important benefit); grade C for physical mobility, muscle force, swelling, and exercise (no benefit). Patients with OA of the knee. | |  |
| General LE exercise program (including muscle force, flexibility, and mobility/coordination) versus control, level 1 (RCT, n=490):  grade A for pain at night and ability on stairs (clinically important benefit); grade C for knee flexion ROM, muscle force, knee joint position, gait, functional status, quality of life, muscle activation, stiffness, and physical activity (no benefit). Patients with a diagnosis of OA. | |  |
| Progression versus no-progression LE strengthening exercises, level 1 (RCT, n=179):  grade A for pain at rest and ROM (clinically important benefit); grade C for stiffness and functional status (no benefit). Patients with radiographic evidence of OA in the tibiofemoral compartment. | |  |
| Whole-body functional exercise versus control, level 1 (RCT, n=864): grade A for pain and functional status (mobility, walking, work, disability in ADL) (clinically important benefit); grade C for knee flexor ROM, quadriceps femoris muscle force, hamstring muscle force, gait, and quality of life (no benefit). Patients with OA of the knee. | |  |
| Walking program versus control, level 1 (RCT, n=1,089):  grade A for pain, functional status, stride length, disability transferring from bed, disability bathing, aerobic capacity, energy level, and medication use (clinically important benefit); grade C for disability in ADL (clinical benefit); grade C for walking speed, disability toileting, disability dressing, blood pressure, morning stiffness, and quality of life (no benefit). Patients with OA. | |  |
| Jogging in water versus control, level 1 (RCT, n=115):  grade A for physical activity and aerobic capacity (clinically important benefit); grade C for morning stiffness, pain, grip force, trunk ROM, functional status, and exercise endurance (no benefit). Patients with current symptoms of chronic pain and stiffness in involved weight-bearing joints. | |  |
| Water exercises versus control, level 1 (RCT, n=30):  grade C for torque and ROM (no benefit). Patients with OA or RA diagnosed by a rheumatologist or an orthopedic physician. | |  |
| Manual therapy combined with exercise versus control, level 1 (RCT, n=83):  grade A for pain (clinically important benefit); grade C for functional status (no benefit). Patients with a diagnosis of OA. | |  |

**PANLAR (28)**

| **Characteristics** | | |
| --- | --- | --- |
| Year | 2016 | |
| Country | not specified (South America) | |
| Target population | KOA/HOA/hand OA patients | |
| LOE Scale | - A: Information from various randomized clinical trials or meta-analysis. - B:  Information from a randomized clinical trial or nonrandomized studies. - C:  Experts’ consensus, case studies, or care standards. | |
| SOR Scale | - I:  There is evidence and/or general agreement that a procedure or treatment is beneficial, useful, or effective. - II: Conflicting evidence and/or differing opinions about the efficacy of a procedure or treatment. - IIa: Evidence and/or agreement favor usefulness or efficacy. - IIb: Usefulness or efficacy is not established by evidence or opinion. - III: Conditions for which there is evidence, general agreement, or both that the procedure treatment is not useful/effective and in some cases may be harmful. | |
| **Recommendations (51)** | | **Ambulatory health intervention before TJR quantifiable in routine data from German SHIs** |
| Information and education regarding the therapeutic objectives and the importance of changes in lifestyle, which include an exercise regimen, weight reduction, use of walking aids (walking stick and crutches) and shoe adjustments and other measures to prevent the progression of joint damage.   - Level of evidence: B - Strength of recommendation: I | | Walking aids |
| Strengthening the extensors and abductors improves functionality and can be used to prepare the patient before a hip implant.   - Level of evidence: B - Strength of recommendation: I | |  |
| Aerobic exercise performed on a regular basis and muscle stretching and strengthening and joint mobility exercises are recommended.   - Level of evidence: B - Strength of recommendation: I | |  |
| Mechanotherapy, including flexibility programs and mobilization and stretching exercises, can reduce pain and improve the range of motion of the knee.   - Level of evidence: A - Strength of recommendation: IIb | |  |
| A program of flexibility, stretching, and strengthening exercises for symptomatic knee OA is recommended as this reduces pain during walking and climbing stairs and improves the strength of the quadriceps femoris.   - Level of evidence: A - Strength of recommendation: I | | Exercise therapy/  referral to PT |
| The use of orthoses is recommended to prevent the progression of degenerative changes and improve hip function.   - Level of evidence: B - Strength of recommendation: IIb | | Orthoses/braces |
| Thermotherapy can be performed to relieve pain.   - Level of evidence: B - Strength of recommendation: I | |  |
| Transcutaneous electrical nerve stimulation (TENS) should also be used for pain relief and to reduce stiffness.   - Level of evidence: B - Strength of recommendation: IIb | | TENS |
| The use of a walking stick in the contralateral hand is also recommended. The handle should be at the level of the greater trochanter of the femur.   - Level of evidence: B - Strength of recommendation: IIb | | Walking aids |
| The use of assistive devices such as a walking stick, walker, or crutches is suggested as a preventive measure. A walking stick must be used in the contralateral hand and the height must be adjusted to the level of the greater trochanter, with the elbow bent at an angle of 25 to 30 degrees.   - Level of evidence: B - Strength of recommendation: IIa | | Walking aids |
| A neuromuscular bandage may be beneficial as it aids analgesia, stimulates circulation, and reduces pressure. Consequently, the patient’s posture is improved.   - Level of evidence: B - Strength of recommendation: IIa | |  |
| The use of acetaminophen/paracetamol is recommended in mild to moderate pain, owing to its safety profile.   - Level of evidence: B - Strength of recommendation: I | | Paracetamol (initial medication) |
| Acetaminophen/paracetamol is recommended at a dose of up to 3 g/day for the treatment of mild pain resulting from knee OA. Moderate gastrolesive effects may occur and patients should be monitored for possible hepatic complications.   - Level of evidence: B - Strength of recommendation: I | | Paracetamol (initial medication) |
| NSAIDs (ibuprofen, naproxen, diclofenac, meloxicam) or selective COX-2 inhibitors (celecoxib, etoricoxib) may be indicated higher than usual doses in more severe pain.   - Level of evidence: B - Strength of recommendation: I | | Oral NSAID |
| Naproxen could be used in patients with cardiovascular risk. It should be administered in conjunction with a proton-pump inhibitor owing to the high gastrointestinal risk.   - Level of evidence: A - Strength of recommendation: I | | Oral NSAID treatment with caution for comorbidities (GI/CV/renal) |
| Weak opioids such as tramadol may be beneficial if there is no response to NSAIDs or COX-2 inhibitors, no toleration, or are contraindicated.   - Level of evidence: B - Strength of recommendation: IIb | | Weak opioids |
| The use of tramadol in the case of severe pain in its various administration forms is recommended.   - Level of evidence: A - Strength of recommendation: I | | Weak opioids |
| The use of hyaluronic acid may be beneficial and, thus, could help to reduce the NSAID use.   - Level of evidence: B - Strength of recommendation: IIb | |  |
| The administration of low-dose oral steroids for a maximum of 12 weeks could be considered in patients older than 65 years.   - Level of evidence: C - Strength of recommendation: IIb | |  |
| Intra-articular corticosteroid injection (ultrasound-guided) may be beneficial to provide fast pain relief in patients who suffer painful relapses and who do not respond to analgesics and NSAIDs.   - Level of evidence: B - Strength of recommendation: IIa | | Corticosteroid injection |
| Intra-articular corticosteroid injection (ultrasound-guided) may be beneficial to provide fast pain relief.   - Level of evidence: B - Strength of recommendation: IIa | | Corticosteroid injection |
| The administration of intra-articular steroids may be reasonable for knee OA accompanied by inflammation.   - Level of evidence: B - Strength of recommendation: IIb | | Corticosteroid injection |
| Avocado and soybean unsaponifiable may play a useful role, and recent studies have provided the evidence that they may slow the progression of OA.   - Level of evidence: A - Strength of recommendation: II | |  |
| The use of diacerein has reported a high rate of adverse effects, such as diarrhea and risk of liver damage.   - Level of evidence: B - Strength of recommendation: III | |  |
| Total hip arthroplasty is indicated when OA is accompanied by pain and walking difficulty and when the quality of life is impaired. It improves not only these factors but also patient survival. A variety of models and metal implants are available and different approaches can be chosen such as the use of a cemented, uncemented, or hybrid prosthesis.   - Level of evidence: A - Strength of recommendation: I | | Referral to orthopaedic surgeon if conservative therapy failed |
| Total knee arthroplasty may be indicated owing to its outstanding effect on pain and stiffness and the improvement obtained in physical activity 6 months after intervention. Proper preoperative planning is essential so that deformities (varus or valgus) and long-term instabilities may be corrected.   - Level of evidence: B - Strength of recommendation: IIa | | Referral to orthopaedic surgeon if conservative therapy failed |
| Information and education regarding treatment goals and the importance of lifestyle changes to reduce the degenerative damage of the knee joint should be provided.   - Level of evidence: A - Strength of recommendation: I | |  |
| Hydrotherapy in a therapeutic tank may be indicated in mild knee pain without swelling or stiffness; it is especially beneficial for elderly patients. A program of exercises for flexibility, mobilization, and stretching can be included.   - Level of evidence: A - Strength of recommendation: IIa | |  |
| Thermotherapy (heat and cold) may help to improve the symptoms of knee OA.   - Level of evidence: A - Strength of recommendation: IIa | |  |
| The use of heat to reduce pain and stiffness before performing flexion exercises in moderate and persistent pain is recommended.   - Level of evidence: B - Strength of recommendation: I | |  |
| A daily walk is recommended as this improves muscle strength, aerobic capacity, and endurance; facilitates a good night’s sleep; and reduces knee pain.   - Level of evidence: A - Strength of recommendation: I | |  |
| Aerobic exercise can be implemented gradually and progressively according to each patient’s level of fitness at a frequency of three or more times per week, with a minimum duration of 20 to 30 minutes per session.   - Level of evidence: A - Strength of recommendation: I | |  |
| Exercises for concentric contraction of the flexor and extensor muscles of the knee are indicated as these have been shown to reduce pain both at rest and during activity.   - Level of evidence: A - Strength of recommendation: I | |  |
| Support devices may be useful for reducing pain and stiffness and improving the functionality of the knee. Insoles and knee braces have been shown to decrease valgus or varus and knee pain.   - Level of evidence: A - Strength of recommendation: IIa | | Shoes/insoles;  Orthoses/braces |
| The use of bandage tape may help to reduce pain in patients with joint instability knee OA.   - Level of evidence: B - Strength of recommendation: IIa | |  |
| NSAIDs such as diclofenac, ibuprofen, and naproxen, and selective NSAIDs including celecoxib and etoricoxib are indicated in moderate pain. In all cases, gastric protection, such as a proton-pump inhibitor, is required and naproxen is recommended in patients with cardiovascular risk.   - Level of evidence: A - Strength of recommendation: I | | Oral NSAID treatment with concomitant PPI/misoprostol in case of Gl risk factors |
| Topical NSAIDs may be indicated in patients with gastrointestinal risk, even though the analgesic response decreases after 1 year of use.   - Level of evidence: A - Strength of recommendation: I | | Topical NSAID |
| Capsaicin gel was shown to be an effective treatment for knee OA accompanied by mild to moderate pain.   - Level of evidence: B - Strength of recommendation: II | |  |
| Chondroitin sulfate has shown to have a beneficial effect on symptoms in patients with knee OA and a high safety profile. It has been proven that its effect persists for 3 months after stopping the treatment (carryover effect). Recent studies have provided evidence that chondroitin sulfate use may delay OA progression.   - Level of evidence: A - Strength of recommendation: I | |  |
| The combined use of glucosamine and chondroitin sulfate is indicated in patients with knee OA and moderate to severe pain.   - Level of evidence: A - Strength of recommendation: I | |  |
| Glucosamine may be beneficial for pain relief and for improving joint function in patients.   - Level of evidence: A - Strength of recommendation: I | |  |
| Avocado soybean unsaponifiable may help to slow the progression of joint damage associated with knee OA.   - Level of evidence: A - Strength of recommendation: IIb | |  |
| Intra-articular injection of hyaluronic acid of different molecular weights has proven to be beneficial in the treatment of knee OA.   - Level of evidence: B - Strength of recommendation: IIa | |  |
| Oral administration of hyaluronic acid may have a beneficial therapeutic effect in patients with symptomatic knee OA and may possibly have an even greater effect in relatively young patients.   - Level of evidence: C - Strength of recommendation: IIb | |  |
| The use of strontium ranelate may be beneficial for the treatment of knee pain.   - Level of evidence: B - Strength of recommendation: IIb | |  |
| Duloxetine may be helpful for knee OA accompanied by chronic pain.   - Level of evidence: C - Strength of recommendation: IIb | | Duloxetine |
| Intra-articular injection of platelet-rich plasma may help to relieve pain associated with knee OA; however, our recommendation is to conduct better quality studies.   - Level of evidence: C - Strength of recommendation: IIb | |  |
| The use of a supplement containing omega-3 and omega-6 fatty acids, zinc and vitamin E could be considered to reduce pain and stiffness and improve joint function, and also to reduce the intake of NSAIDs/analgesics.   - Level of evidence: B - Strength of recommendation: IIb | |  |
| Intra-articular injection of mesenchymal stem cells derived from the infrapatellar fat pad may be effective at reducing pain and improving knee function.   - Level of evidence: C - Strength of recommendation: III | |  |
| There is no benefit associated with the use of arthroscopy in the treatment of knee OA, even in the presence of a partial meniscal tear.   - Level of evidence: A - Strength of recommendation: III | |  |
| In patients with a partial rupture of the meniscus, a partial meniscectomy performed arthroscopically may be beneficial, followed by a physical therapy program.   - Level of evidence: B - Strength of recommendation: IIa | |  |

**Philadelphia Panel (29)**

| **Characteristics** | | |
| --- | --- | --- |
| Year | 2001 | |
| Country | US | |
| Target population | KOA patients | |
| LOE Scale | - A: clinical importance: >15%; stastical significance: <.05; study design: RCT (single or meta-analysis) - B: clinical importance: >15%; stastical significance: <.05; study design: CCT or observational (single or meta-analysis), with a quality score of 3 or more on the 5-point Jadad methodologic quality checklist - C+: clinical importance: >15%; stastical significance: not significant; study design: RCT or CCT or observational (single or meta-analysis) - C: clinical importance: <15%; stastical significance: unimportant; study design: any study design - D: clinical importance: <0% (favors control); stastical significance: /; study design: well-designed RCT with >100 patients | |
| SOR Scale |  |  |
| **Recommendations (10)** | | **Ambulatory health intervention before TJR quantifiable in routine data from German SHIs** |
| The Philadelphia Panel recommends that there is poor evidence to include or exclude therapeutic ultrasound alone (grade C for patient global assessment) as an intervention for patellofemoral pain syndrome. | |  |
| The Philadelphia Panel recommends that there is poor evidence to include or exclude preoperative strengthening exercises alone (grade C for pain and function) prior to unilateral knee arthroplasty surgery. | |  |
| The Philadelphia Panel recommends that there is good evidence to include strengthening, stretching, and functional exercises alone (grade A for pain and patient global assessment, grade C for function) as interventions for knee osteoarthritis pain. | | Exercise therapy/  referral to PT |
| The Philadelphia Panel recommends that there is poor evidence to include or exclude cryotherapy (grade C for pain) as an adjunct intervention to home exercises after knee surgery. | |  |
| The Philadelphia Panel recommends that there is poor evidence to include or exclude TENS alone (grade C for pain) as an intervention after knee surgery). | | TENS |
| The Philadelphia Panel recommends that there is good evidence to include TENS as an intervention for pain associated with knee osteoarthritis (grade A for pain and patient global assessment). | | TENS |
| The Philadelphia Panel recommends that there is poor evidence to include or exclude ice massage alone (grade C for pain) as an intervention for knee osteoarthritis. | | Massage |
| The Philadelphia Panel recommends that there is poor evidence to include or exclude therapeutic ultrasound alone (grade C for pain) as an intervention for knee osteoarthritis. | |  |
| The Philadelphia Panel recommends that there is poor evidence to include or exclude electrical stimulation alone (grade C for function) as an intervention for knee osteoarthritis. Because electrical stimulation is usually used to improve strength, this recommendation is inconclusive until evidence of effects on strength have been shown in clinical trials. | | NMES |
| The Philadelphia Panel recommends that there is poor evidence to include or exclude deep friction massage alone (grade C for pain) as an intervention for iliotibial band syndrome. | | Massage |

**SFR (30)**

| **Characteristics** | | |
| --- | --- | --- |
| Year | 2020 | |
| Country | France | |
| Target population | KOA patients (medication) | |
| LOE Scale | - 1A: Meta-analysis of randomised controlled trials - 1B:  At least one randomised controlled trial - 2A:  At least one controlled trial without randomisation - 2B:  At least one type of quasi-experimental study - 3:  Descriptive studies, such as comparative studies, correlation studies or case-control studies - 4:  Expert committee reports or opinions and/or clinical experience of respected authorities | |
| SOR Scale | - A:  category 1 evidence - B: category 2 evidence or extrapolated recommendations from category 1 evidence - C: category 3 evidence or extrapolated recommendations from category 1 or 2 evidence - D: category 4 evidence or extrapolated recommendations from category 2 or 3 evidence - Expert consensus: Level of Agreement (n=31): 0-10 numerical rating scale (0=complete disagreement, 10=complete agreement) | |
| **Recommendations (11)** | | **Ambulatory health intervention before TJR quantifiable in routine data from German SHIs** |
| Paracetamol must not necessarily be prescribed systematically and/or continuously.   - Level of evidence: 1A - Strength of recommendation: A - Level of Agreement: 9.3 | | Paracetamol (initial medication) |
| Oral NSAIDs must be used for the shortest time and at the lowest effective dose possible. They can be proposed as first-line therapy if there are no contraindications or cardiovascular risk factors and/or risk factors for gastrointestinal complications. The prescription and choice of NSAID must take into account the patient's comorbidities and must be prescribed after providing information about adverse effects.   - Level of evidence: 1A (shortest time and lowest effective dose), 1A (first-line therapy if there are no contraindications), 4 (taking into account comorbidities and information about possible adverse effects) - Strength of recommendation: A (shortest time and lowest effective dose), A (first-line therapy if there are no contraindications), D (taking into account comorbidities and information about possible adverse effects) - Level of Agreement: 9.0 | | Oral NSAID;  Oral NSAID treatment with caution for comorbidities (GI/CV/renal) |
| Topical NSAIDs can be proposed.   - Level of evidence: 1A - Strength of recommendation: B - Level of Agreement: 9.0 | | Topical NSAID |
| Weak opioids, alone or in combination with paracetamol, may be proposed for analgesic purposes and must be prescribed while taken into account comorbidities and after providing information about adverse effects.   - Level of evidence: 1A (prescription for analgesic purposes), 4 (taking into account comorbidities and information about possible adverse effects) - Strength of recommendation: A (prescription for analgesic purposes), D (taking into account comorbidities and information about possible adverse effects) - Level of Agreement: 9.5 | | Weak opioids |
| Strong opioids must only be prescribed to patients who have a contraindication to knee surgery, who have failed or have a contraindication to other treatments, while taking into account comorbidities and after providing information about adverse effects.   - Level of evidence: 4 - Strength of recommendation: D - Level of Agreement: 9.1 | | Strong opioids |
| Intra-articular corticosteroid injections can be proposed, especially for inflammatory flare-ups with joint effusion.   - Level of evidence: 1A (proposition of intra-articular corticosteroids), 4 (joint effusion) - Strength of recommendation: A (proposition of intra-articular corticosteroids), D (joint effusion) - Level of Agreement: 9.7 | | Corticosteroid injection |
| Intra-articular hyaluronic acid injections can be proposed, without expecting a chondroprotective effect.   - Level of evidence: 1A (proposition of intra-articular hyaluronic acid), 1B (without expaectation of chondroprotective effect) - Strength of recommendation: A (proposition of intra-articular hyaluronic acid), B (without expaectation of chondroprotective effect) - Level of Agreement: 9.1 | |  |
| Symptomatic slow-acting drugs of osteoarthritis (avocado and soybean unsaponifiables, chondroitin, glucosamine, diacerein) can be proposed, without expecting a chondroprotective effect.   - Level of evidence: 1A (proposition of symptomatic slow-acting drugs), 1A (without expaectation of chondroprotective effect) - Strength of recommendation: A (proposition of symptomatic slow-acting drugs), A (without expaectation of chondroprotective effect) - Level of Agreement: 8.7 | |  |
| Low-dose topical capsaicin (< 1%) can be considered.   - Level of evidence: 1A - Strength of recommendation: C - Level of Agreement: 8.2 | |  |
| Off-label duloxetine can be considered in the absence of therapeutic alternatives.   - Level of evidence: 1A - Strength of recommendation: C - Level of Agreement: 8.2 | | Duloxetine |
| No conclusions could be made on the benefits of intra-articular injections of platelet concentrates given the lack of follow-up and insufficient data.   - Level of evidence: N/A - Strength of recommendation: N/A - Level of Agreement: 8.2 | |  |

**SIR (31)**

| **Characteristics** | | |
| --- | --- | --- |
| Year | 2019 | |
| Country | Italy | |
| Target population | KOA/HOA/hand OA patients | |
| LOE Scale | - 1: From meta-analysis of randomized controlled trials or from at least one randomized controlled trial - 2:  From at least one controlled study without randomization or from at least one cohort study - 3:  From at least one case-control study - 4:  From case-series or poor-quality cohort and case-control studies - 5:  From expert committee reports or opinions and/or clinical experience of respected authorities | |
| SOR Scale | - Rating of strength of recommendation by external reviewers: 0-10 numerical rating scale | |
| **Recommendations (16)** | | **Ambulatory health intervention before TJR quantifiable in routine data from German SHIs** |
| Findings indicative of OA: Symptoms: painful and/or restricted movement, instability and regional pain. More persistent rest and night pain may occur in advanced OA. OA symptoms are often episodic or variable in severity and slow to change. Signs: crepitus, bony enlargement/deformity, absent or modest joint inflammation, bony ankylosis, only short-lived morning stiffness, malalignment, and the presence of Heberden’s nodes. Clinical/family history: female sex, age over 40 years, menopausal status, family history, obesity, joint laxity, prior hand injury, occupation or recreation-related usage.   - Level of evidence: 1-4 - Rating of recommendation: 100% of scores ≥7 | | Physician visit for musculoskeletal disorders |
| Imaging is not required to make the diagnosis in patients with typical presentation of OA. In atypical presentations, imaging is recommended to help confirm the diagnosis of OA and/or make alternative or additional diagnoses. Imaging features do not predict non-surgical treatment response.   - Level of evidence: 2-4 - Rating of recommendation: 67% of scores ≥7 | | Radiographic assessment (prior to CT, MRI, sonography) |
| If imaging is needed, conventional (plain) radiography should be used before other modalities because it is the current gold standard for morphological assessment of OA. The recommended views are: A) [for the knee], weight bearing and patello-femoral views, B) [for the hand] a postero-anterior radiograph of both hands on a single film/field of view. Classical features are focal joint space narrowing, osteophyte, subchondral bone sclerosis and subchondral cysts. To make additional diagnoses, soft tissues are best imaged by ultrasounds (US) or magnetic resonance imaging (MRI) and bone by computed tomography (CT) or MRI.   - Level of evidence: 1-2 - Rating of recommendation: 100% of scores ≥7 | | Radiographic assessment (prior to CT, MRI, sonography) |
| If a palpable effusion is present, synovial fluid should be aspirated and analysed to exclude inflammatory disease and to identify urate and calcium pyrophosphate crystals. OA synovial fluid is typically non-inflammatory with <2000 leukocytes/mm^3^; if specifically sought, basic calcium phosphate crystals are often present.   - Level of evidence: 2 - Rating of recommendation: 75% of scores ≥7 | |  |
| Blood, urine or synovial fluid tests are not required for diagnosis of OA but may be required for differential diagnosis. In OA patients with marked inflammatory symptoms and/or signs, especially involving atypical sites, laboratory tests should be undertaken.   - Level of evidence: 1-2 - Rating of recommendation: 100% of scores ≥7 | |  |
| Optimal management of OA requires a combination of non-pharmacological and pharmacological treatment modalities individualized to the patient’s needs.   - Level of evidence: 5 - Rating of recommendation: 100% of scores ≥7 | | Combination of pharmacological and non-pharmacological therapies (before TJR) |
| Treatment of hand, hip and knee OA should be individualized according to: 1) the wishes and expectations of the individual, 2) localization, severity of structural change and type of OA, 3) risk factors (such as age, sex, obesity and adverse mechanical factors), 4) presence of inflammation, 5) comorbidity and co-medication, 6) OA in other sites.   - Level of evidence: 1-4 - Rating of recommendation: 100% of scores ≥7 | |  |
| Paracetamol (acetaminophen) (up to 3 g/day) is an effective initial oral analgesic for treatment of mild to moderate pain. In elderly patients it should be preferred because of its relative safety in comparison with NSAIDs. The use of weak opioids in case of severe pain or no response, intolerance or contraindication to NSAIDs, is recommended. Stronger opioids should only be used for the management of severe pain in exceptional circumstances. Duloxetine may be helpful for knee (and maybe hip) OA accompanied by chronic pain.   - Level of evidence: 1-4 - Rating of recommendation: 92% of scores ≥7 | | Paracetamol (initial medication);  Weak opioids;  Strong opioids;  Duloxetine |
| Oral NSAIDs are recommended at the lowest effective dose and for the shortest duration in patients who respond inadequately to paracetamol. NSAIDs (such as ibuprofen, diclofenac and naproxen) and selective COX-2 inhibitors (including celecoxib and etoricoxib) are indicated in moderate pain. Higher doses of oral NSAIDs may be indicated in more severe pain. In patients with increased gastrointestinal risk, non-selective NSAIDs plus a proton-pump inhibitor, or a selective COX-2 inhibitor, should be used. In patients with increased cardiovascular risk, naproxen can be used; COX-2 inhibitors are contraindicated and other non-selective NSAIDs should be used with caution. In nephropathic patients the use of NSAID and COX-2 inhibitors should be avoided.   - Level of evidence: 1 - Rating of recommendation: 92% of scores ≥7 | | Oral NSAID;  Oral NSAID treatment with concomitant PPI/misoprostol in case of Gl risk factors;  Oral NSAID treatment with caution for comorbidities (GI/CV/renal) |
| Topical pharmacological treatments are preferred over systemic treatments, especially for mild to moderate pain and when only a few joints are affected. Topical NSAIDs and capsaicin gel are effective and safe treatments. Patients with age >75 years should use topical rather than oral NSAIDs even though the analgesic response decreases after 1 year of use.   - Level of evidence: 1-2 - Rating of recommendation: 100% of scores ≥7 | | Topical NSAID |
| The accuracy of intra-articular injection depends on the joint and on the skills of the practitioner. Ultrasound-guidance may improve accuracy and it is particularly recommended for joints that are difficult to access due to the site itself, degree of deformity or obesity. Hyaluronic Acid: intra-articular injection of hyaluronic acid of different molecular weights may give symptomatic benefit with low toxicity and could help to reduce the NSAID use. Steroids: intra-articular corticosteroid injection may be beneficial, providing fast pain relief in patients who suffer painful relapses and who do not respond or have a contraindication to analgesics and NSAIDs. Mesenchymal stem cells and/or platelet rich plasma: it is unclear if intra-articular injection of mesenchymal stem cells or platelet-rich plasma can help to relieve pain associated with knee OA.   - Level of evidence: 1-5 - Rating of recommendation: 83% of scores ≥7 | | Corticosteroid injection |
| In patients with symptomatic knee OA, glucosamine sulphate and chondroitin sulphate may have a beneficial effect on symptoms. Structural effects, patients suitable for treatment and the cost to benefit ratio of the therapy remain to be defined.   - Level of evidence: 1-2 - Rating of recommendation: 92% of scores ≥7 | |  |
| Concerning patient education, lifestyle changes and therapeutic exercise:  Patients’ education – Information, education and an individually tailored program, including long-term and short-term goals, intervention or action plans to reduce the degenerative damage of the OA should be provided. People with hip and/or knee OA should be taught a regular individualized (daily) exercise regimen and participate in self-management programs, strengthening, low-impact aerobic exercises, and neuromuscular education.  Life styles – Patients with hip and knee OA, who are overweight, should be encouraged to lose weight and maintain their weight at a lower level. People with hip or knee OA at risk of work disability should have access to vocational rehabilitation, including counselling on modifiable work-related factors.  Exercise – The mode of delivery of exercise education should be selected according both to the preference of the person with hip or knee OA and local availability. Patients with knee OA should participate in aerobic and/or resistance land-based and/or aquatic exercise.   - Level of evidence: 1-3 - Rating of recommendation: 100% of scores ≥7 | | Exercise therapy/  referral to PT |
| Orthoses prevent the progression of degenerative changes and improve function. In hip and knee OA, the use of assistive devices such as a walking-stick or crutches is suggested as a preventive measure. The use of appropriate and comfortable shoes is recommended. The combination of splints for thumb base OA, orthoses and exercise regimen reduce pain and improve functionality in the short and long term and prevent/correct lateral angulation and flexion deformity.   - Level of evidence: 1-2 - Rating of recommendation: 92% of scores ≥7 | | Walking aids  Shoes/insoles |
| Concerning TENS, acupuncture, balneotherapy and exercises in water, manual therapy and patellar taping: TENS: transcutaneous electrical nerve stimulation (TENS) may help with short-term pain control in some patients with hip or knee OA. Acupuncture: the usefulness in patients with symptomatic OA of the knee and hip remains to be defined. Balneotherapy and exercises in water are effective for relieving symptoms in hip and knee (and hand) OA. Manual therapy/Taping: it is unclear if manual therapy can be useful in patients with symptomatic osteoarthritis of the knee. The use of bandage tape may help to reduce pain in patients with joint instability knee OA.   - Level of evidence: 1-4 - Rating of recommendation: 92% of scores ≥7 | | TENS;  Acupuncture |
| Orthopedic surgery should be considered in patients with radiographic evidence of OA, who have marked disability, reduced quality of life and pain refractory to other treatments.   - Level of evidence: 5 - Rating of recommendation: 92% of scores ≥7 | | Referral to orthopaedic surgeon if conservative therapy failed |

**TLAR (32)**

| **Characteristics** | | |
| --- | --- | --- |
| Year | 2018 | |
| Country | Turkey | |
| Target population | KOA patients | |
| LOE Scale | - Ia: Meta-analysis of randomized controlled studies - Ib At least one randomized controlled study - IIa: At least one well-designed controlled study, but without randomization - IIb: At least one well-designed quasi-experimental study - III: At least one non-experimental, descriptive studies (comparative, correlation or a case–control study) - IV: Expert committee reports, opinions and/or experience of respected authors | |
| SOR Scale | - Rating of strength of recommendation by expert panel (n=17): 0-10cm VAS | |
| **Recommendations (11)** | | **Ambulatory health intervention before TJR quantifiable in routine data from German SHIs** |
| The main goal in the treatment of knee OA should be directed toward controlling pain, preserving and improving the function of joints, providing functional independency, and increasing the quality of life. In order to reach these goals, management of knee OA should contain non-pharmacologic, pharmacologic, and, when necessary, surgical approaches. Treatment should be tailored for each patient individually.   - Level of evidence: IV - Strength of recommendation: 9.93 | | Combination of pharmacological and non-pharmacological therapies (before TJR) |
| All patients with knee OA should be informed about the disease and its treatment. Patients should be educated how they can protect their joints and how to save energy while performing occupational, sportive, daily living and recreational activities. The educational programme should include instructions about life style alteration, joint protection techniques and issues concerning body weight control such as dietary and exercise programmes.   - Level of evidence: Ib - Strength of recommendation: 9.61 | |  |
| People with symptomatic knee osteoarthritis and body mass index of equal to and greater than 25 should be advised to lose weight.   - Level of evidence: Ia, IIa - Strength of recommendation: 9.71 | |  |
| All patients should be evaluated individually and appropriate exercise programme should be planned accordingly.   - Level of evidence: Ia - Strength of recommendation: 9.78 | | Exercise therapy/  referral to PT |
| Asymptomatic people who have risk factors (overweight, malalignment, joint hypermobility, etc.) should be evaluated by physical medicine and rehabilitation (PM&R) specialists, and required precautions should be applied and followed accordingly.   - Level of evidence: IV - Strength of recommendation: 8.71 | |  |
| The electrotherapeutic agents with analgesic properties (TENS, interferential and diadynamic current) may have beneficial effects over pain, functional status and quality of life and can be used at all stages of the disease. Physical agents with superficial heating (hot-packs, infrared, etc.) and deep heating (ultrasound, short-wave diathermy) property may have beneficial effects over pain and functional status of selected patients who have not active synovitis. Superficial cold can be applied in case of active synovitis.   - Level of evidence: Ia, Ib, IV - Strength of recommendation: 9.10 | | TENS |
| Acetaminophen can be used in addition to primary precautions in patients who have mild disease with intermittent pain, short-term stiffness after inactivation and radiological knee OA of grade 0–1. But comorbidities should be kept in mind along with required precautions against gastrointestinal (GI), hepatic and cardiovascular (CV) side effects. These patients can be used topical NSAIDs. Appropriate arrangements can be applied according to biomechanical assessment if necessary. Although the evidence about the use of glucosamine and/or chondroitin sulphate is uncertain, these agents may be used.   - Level of evidence: Ia, Ib, III - Strength of recommendation: 8.95 | | Paracetamol (initial medication);  Oral NSAID treatment with caution for comorbidities (GI/CV/renal);  Topical NSAID |
| Patients with moderate–severe symptoms, functional capacity of either normal or minimally limited and/or radiologic grade of 2–3 may be treated with NSAIDs in case of response to acetaminophen is absent or insufficient. These patients may be treated with intraarticular hyaluronic acid even though the evidence about its efficiency is uncertain. If there are inflammatory findings and the other treatment options have failed, intraarticular glucocorticoids can be applied, not more than 3 times-a-year  A. Duloxetine can be used in patients with chronic pain which is   unresponsive to other treatment options  B. Balneotherapy can also be applied in these stages of the   disease)  C. Assistive and adaptive devices should also be advised to these   patients in order to control biomechanical defects and to lessen   load  D. Osteotomy, as a biomechanical correcting and preservative   surgical method, can be advised to middle-aged and active   patients with malalignment   - Level of evidence: Ia, Ib (A); Ia, Ib, IIa (B); Ia, IIa (C); Ia (D) - Strength of recommendation: 7.81 (A); 8.84 (B); 8.71 (C); 7.50 (D) | | Oral NSAID;  Corticosteroid injection;  Duloxetine |
| All of the treatment options mentioned before are indicated also for patients who are severely symptomatic, and with limited functional capacity, deformities and/or radiological grade of 4. Weak opioid analgesics can be given for a short period of time to the patients who are not responding to other options.   - Level of evidence: Ib - Strength of recommendation: 8.46 | | Weak opioids |
| In patients with neuropathic pain components also, an additional neuropathic pain protocol for diagnosis and treatment should be applied.   - Level of evidence: Ib, III - Strength of recommendation: 7.44 | |  |
| Total knee arthroplasty (TKA) should be considered in patients with advanced knee OA, who are resistant to pharmacological and non-pharmacological treatments and having pain, functional limitation and deterioration in daily living of life. While deciding TKA, not only the radiographic findings but also the pain and functional status of patient should be taken into account.   - Level of evidence: Ia - Strength of recommendation: 9.79 | | Referral to orthopaedic surgeon if conservative therapy failed |

**Yabuki S, et al. (33)**

| **Characteristics** | | |
| --- | --- | --- |
| Year | 2019 | |
| Country | South Korea | |
| Target population | OA/cLBP patients (medication) | |
| LOE Scale | not reported | |
| SOR Scale | - Strong - Weak - N/A (no recommendation, where evidence is conflicting or limited) | |
| **Recommendations (28)** | | **Ambulatory health intervention before TJR quantifiable in routine data from German SHIs** |
| Pain management should be individualized to each patient and optimized with the goal of improving activity of daily living, quality of life, and ability to carry out rehabilitation exercises.   - Strength of recommendation: not reported | |  |
| Pain management strategy should be multimodal and multidisciplinary, encompassing physical exercise, pharmacological therapy, surgery, dietary advice, weight management, patient education, and aids and assistive devices.   - Strength of recommendation: not reported | | Combination of pharmacological and non-pharmacological therapies (before TJR) |
| Modification to pain management strategy should be based on the patient’s response to treatment, and with careful considerations for potential side effects.   - Strength of recommendation: not reported | |  |
| Clinicians should formulate an agreeable pain management plan with the patient.   - Strength of recommendation: not reported | |  |
| We recommend non-pharmacological and non-surgical treatment, such as physical exercise and exercise therapies, as the primary approach in the management of patients with OA pain and cLBP; advice to stay active should be given to improve disability in the long term.   - Strength of recommendation: not reported | | Exercise therapy/  referral to PT |
| The use of pharmacological treatment as an adjuvant to physical exercise should be based on benefits for each individual patient; the adverse effects of the therapy choice should not affect the general well-being of the patient and should be acceptable to the patient. Previous treatment history, including adverse effect profile, should be carefully recorded.   - Strength of recommendation: not reported | | Combination of pharmacological and non-pharmacological therapies (before TJR) |
| Clinicians should regularly assess and monitor patients with OA pain and cLBP, including treatment efficacy and adverse effects; continuous monitoring of adverse effects is important to ensure patient safety and prevent nonadherence to treatment.   - Strength of recommendation: not reported | | Physician visit for musculoskeletal disorders |
| Clinicians should frequently follow-up patients until the optimal treatment can be determined based on patients’ response; follow-up interval could be adjusted gradually.   - Strength of recommendation: not reported | | Physician visit for musculoskeletal disorders |
| Clinicians should do assess pain, function, AEs, mood and aberrant behaviors at regular intervals during strong opioids use.   - Strength of recommendation: Strong | | Physician visit for musculoskeletal disorders |
| Referral to a pain management programme should be considered for patients with chronic pain who are unresponsive to recommended pharmacological, physical, and psychological interventions.   - Strength of recommendation: not reported | |  |
| Clinicians should also assess pain behaviors and pain-associated complaints of the patient, and monitor for objective changes such as bone change, muscle and joint contractures, and presence of red flags (tumor, inflammations, etc.).   - Strength of recommendation: not reported | | Physician visit for musculoskeletal disorders |
| In addition to the standard multi-dimensional pain assessment, quality of sleep and global impression on improvement may be included as additional assessment criteria.   - Strength of recommendation: not reported | |  |
| Long-term use of acetaminophen is not recommended in patients with OA pain and cLBP.   - Strength of recommendation: Weak | | Paracetamol (initial medication) |
| Liver function should be monitored when acetaminophen is used in patients with OA pain and cLBP who may be at risk for liver toxicity.   - Strength of recommendation: Strong | |  |
| Topical NSAIDs should be given as a first-line pharmacological treatment of OA pain.   - Strength of recommendation: Weak | | Topical NSAID |
| If topical NSAIDs are ineffective or provide insufficient pain relief, addition of an oral NSAID should be considered.   - Strength of recommendation: Strong | | Oral NSAID |
| Oral NSAIDs should be avoided in patients with a high risk of GI, cardiovascular, and renal complications, or chronic kidney disease. Long-term use of NSAIDs should be avoided.   - Strength of recommendation: Strong | | Oral NSAID treatment with caution for comorbidities (GI/CV/renal) |
| Gastroprotective agents, such as PPIs, should be used together with traditional NSAIDs in patients with higher GI risk.   - Strength of recommendation: Strong | | Oral NSAID treatment with concomitant PPI/misoprostol in case of Gl risk factors |
| Duloxetine could be considered for the management of OA pain and cLBP.  Strength of recommendation: Weak | | Duloxetine |
| Monitoring for AEs during treatment with antidepressants or anticonvulsants should be regularly done.   - Strength of recommendation: Strong | |  |
| Opioids should only be considered in patients with OA pain and cLBP whose pain control and ADL is not adequately achieved by simple analgesics, NSAIDs, or antidepressants, and when the patient is committed to participating in a multimodal management program.   - Strength of recommendation: Strong | | Weak opioids |
| Clinicians should inform patients the liability of psychological dependence and potential AEs from chronic long-term use of opioids.   - Strength of recommendation: Strong | |  |
| The use of opioids should only be continued if there are clinically relevant improvements in both pain and function.   - Strength of recommendation: Strong | |  |
| Combination pharmacotherapy should be considered in OA and cLBP in order to treat both the nociceptive and neuropathic components of pain.   - Strength of recommendation: Weak | |  |
| Combination pharmacotherapy should be considered if the combination is more effective and/or associated with fewer side-effects than the component drugs alone.   - Strength of recommendation: Weak | |  |
| No recommendation could be made with regard to the use of the combination of acetaminophen with NSAIDs, or acetaminophen with tramadol in patients with OA and cLBP.   - Strength of recommendation: N/A | |  |
| The combination of pregabalin with NSAIDs or transdermal buprenorphine should be considered in patients with a neuropathic component to OA and cLBP.   - Strength of recommendation: Weak | |  |
| Pharmacological action and interaction of each pharmacotherapy should be considered when prescribing combination pharmacotherapies.   - Strength of recommendation: Strong | |  |

**Yeap SS, et al. (34)**

| **Characteristics** | | |
| --- | --- | --- |
| Year | 2021 | |
| Country | Malaysia | |
| Target populatio | KOA patients | |
| LOE Scale | not reported | |
| SOR Scale | Expertpanel consensus (n=9): 5-point Likert scale (1=strongly disagree, 2=disagree, 3=neutral, 4=agree, 5=strongly agree); consensus defined a priori as a combined “strongly agree” and “agree” acceptance rate of ≥70% with no more than one outlier, or ≥1 Likert point from the mean in either direction | |
| **Recommendations (9)** | | **Ambulatory health intervention before TJR quantifiable in routine data from German SHIs** |
| The group recommends that all patients with osteoarthritis of the knee should be assessed carefully to establish their diagnosis, severity and clinical risk profile before initiating treatment.   - Expertpanel consensus: 100% agreement | | Physician visit for musculoskeletal disorders |
| The group recommends that treatment should be individualised to each patient’s symptoms, and a combination of treatment modalities, including pharmacological and physical therapies, should be used to manage patients with knee osteoarthritis.   - Expertpanel consensus: 100% agreement | | Combination of pharmacological and non-pharmacological therapies (before TJR) |
| The group recommends the application of a core treatment set, consisting of patient education, weight loss (if overweight), exercise programmes, and reduction of knee loading throughout the management of knee osteoarthritis.   - Expertpanel consensus: 100% agreement | | Exercise therapy/  referral to PT |
| The group recommends the use of crystalline glucosamine sulphate as background therapy for the management of knee osteoarthritis, with as needed topical nonsteroidal anti-inflammatory drugs and/or oral paracetamol. Background pharmacological therapy can be initiated concurrently with prescribed physical therapies when indicated.   - Expertpanel consensus: 100% agreement | |  |
| The group recommends intermittent or longer cycles of oral non-steroidal anti-inflammatory drugs (NSAIDs; selective or non-selective) as advanced pharmacological treatment for patients who are still symptomatic after receiving background pharmacological and/or physical therapies. The use of oral NSAIDs should be based on an individual patient’s risk profile.   - Expertpanel consensus: 100% agreement | | Oral NSAID |
| The group recommends the use of intraarticular hyaluronate (IAHA) or intraarticular corticosteroids (IACS) in patients who have contraindications to oral non-steroidal anti-inflammatory drugs (NSAIDs), or if patients are still symptomatic despite the use of oral NSAIDs. In patients with knee effusion or synovitis, the use of IACS is preferred.   - Expertpanel consensus: 100% agreement | |  |
| The group recommends the judicious use of short-term weak opioids or paracetamol/tramadol combination as rescue medication in patients who have not achieved satisfactory pain control despite the use of oral nonsteroidal anti-inflammatory drugs and/or intraarticular injections.   - Expertpanel consensus: 100% agreement | | Weak opioids |
| The group recommends the concurrent use of individual advanced pharmacological therapies (oral non-steroidal anti-inflammatory drugs, intraarticular injections, and rescue medication) when patients do not respond sufficiently to monotherapy. Patients should be assessed regularly, and treatment adjusted based on their response.   - Expertpanel consensus: 100% agreement | |  |
| The group recommends knee replacement surgery (total or partial) for patients with severe/advanced knee osteoarthritis.   - Expertpanel consensus: 100% agreement | | Referral to orthopaedic surgeon if conservative therapy failed |

**Zhang Z, et al. (35)**

| **Characteristics** | | |
| --- | --- | --- |
| Year | 2020 | |
| Country | China | |
| Target population | OA patients | |
| LOE Scale | - Level A (high) - Level B (moderate) - Level C (low) - Level D (very low) | |
| SOR Scale | - Strong (Class 1) - Weak (Class 2) | |
| **Recommendations (16)** | | **Ambulatory health intervention before TJR quantifiable in routine data from German SHIs** |
| It is recommended that clinicians diagnose OA under the premise of excluding other types of joint diseases based on the main clinical manifestations such as pain in joint activity and morning stiffness (≤30 minutes).   - Level of evidence: B - Strength of recommendation: 1 | | Physician visit for musculoskeletal disorders |
| It is recommended that clinicians evaluate patients comprehensively based on risk factors (weight load, inflammation, metabolism, etc.), clinical manifestations and position of joint involvement.   - Level of evidence: B - Strength of recommendation: 1 | | Physician visit for musculoskeletal disorders |
| The purpose of OA treatment is to relieve pain, prevent deformity, improve function and life quality.   - Level of evidence: B - Strength of recommendation: 1 | |  |
| It is recommended that OA patients should control their weight, and those who are overweight or obese should lose weight.   - Level of evidence: A - Strength of recommendation: 1 | |  |
| It is recommended to carry out health education for OA patients, mainly to educate them about the causes, prevention, progress and treatment of the disease, reduce the burden of patients’ thoughts, and improve their self-management efficiency. OA patients should reduce long-term standing, kneeling and squatting positions, ascending stairs activity, as well as bad posture, etc. It is recommended for OA patients to take reasonable joint muscle training and moderate aerobic exercise. It is recommended for OA patients to choose different activities according to the location of the disease, such as grasping and holding activities of hand joints, flexion and extension activities of knee joints under the condition of non-load, and gentle activities in different directions of cervical and lumbar joints.   - Level of evidence: B (education), B (avoidance of long-term standing etc.), B (exercise), B (activities according to the location of the disease) - Strength of recommendation: 1 (education), 2 (avoidance of long-term standing etc.), 1 (exercise), 1 (activities according to the location of the disease) | | Exercise therapy/  referral to PT |
| For patients with mild pain, topical application of non-steroidal anti-inflammatory drugs (NSAIDs) is recommended to reduce local pain, and external application of Chinese medicine may also be considered.   - Level of evidence: B (topical NSAIDs), B (Chinese medicine) - Strength of recommendation: 1 (topical NSAIDs), 2 (Chinese medicine) | | Topical NSAID |
| Physical therapy such as manipulation therapy, massage, and acupuncture is recommended for OA patients to relieve pain and improve physical function.   - Level of evidence: B - Strength of recommendation: 2 | | Massage;  Acupuncture |
| For some patients, treatment with glucosamine or chondroitin sulfate can be selected. It should be stopped if no symptom improved after 3 to 6 months.   - Level of evidence: C - Strength of recommendation: 2 | |  |
| For OA patients with persistent pain or moderate or severe pain, it is recommended to choose oral NSAIDs after risk assessment, and use the lowest effective dose for a short period (1–3 months) alone. The combination of COX-2 inhibitor and proton pump inhibitor is recommended for patients with high risk of gastrointestinal adverse reactions.   - Level of evidence: B (NSAIDs at lowest effective dose for a short period), B (COX-2 inhibitor and PPI) - Strength of recommendation: 1 (NSAIDs at lowest effective dose for a short period), 1 (COX-2 inhibitor and PPI) | | Oral NSAID;  Oral NSAID treatment with concomitant PPI/misoprostol in case of Gl risk factors |
| For patients with OA treated with oral drugs, some oral Chinese medicine can be considered in combination.   - Level of evidence: C - Strength of recommendation: 2 | |  |
| For patients of knee OA with persistent or moderate to severe pain, intra-articular injection of glucocorticoids is recommended for rapid relief of pain in patients with OA, the injection interval should not be shorter than 4 to 6 months.   - Level of evidence: B - Strength of recommendation: 1 | | Corticosteroid injection |
| For patients of knee OA with persistent or moderate to severe pain, intra-articular injection of HA can be considered to improve the patient’s symptoms in the long term and delay the time required for joint replacement.   - Level of evidence: C - Strength of recommendation: 2 | |  |
| For OA patients with NSAIDs contraindications or ineffective pain treatment, it is suggested to take opioids or duloxetine for analgesia, or to combine diacerein, inflammatory skin extract of cowpox vaccine to inoculate of rabbits, tanezumab, technetium-99m methylene diphosphonate or bulleyaconitine A.   - Level of evidence: C (opioids or duloxetine), D (combination of diacerin etc.) - Strength of recommendation: 2 (opioids or duloxetine), 2 (combination of diacerin etc.) | | Weak opioids;  Duloxetine |
| For patients with knee OA who have poor responses with intra-articular injection of HA, stem cell injection may be considered.   - Level of evidence: D - Strength of recommendation: 2 | |  |
| For knee OA patients with poor pain treatment response and mechanical symptoms, we recommend arthroscopy to reduce symptoms after assessing the risk of surgery.   - Level of evidence: C - Strength of recommendation: 2 | |  |
| For patients with hip or knee OA who have poor response to conservative treatment and whose quality of life is significantly affected, we recommend to perform joint replacement after assessing the risk of surgery, which can relieve pain, increase the range of joint movement, and improve quality of life.   - Level of evidence: B - Strength of recommendation: 1 | | Referral to orthopaedic surgeon if conservative therapy failed |

**REFERENCES**

1. American Academy of Orthopaedic Surgeons (AAOS) (2021) Management of Osteoarthritis of the Knee (Non-Arthroplasty): Evidence-Based Clinical Practice Guideline. <https://www.aaos.org/globalassets/quality-and-practice-resources/osteoarthritis-of-the-knee/oak3cpg.pdf>

2. American Academy of Orthopaedic Surgeons (AAOS) (2017) Management of Osteoarthritis of the Hip. Evidence-based Clinical Practice Guideline. <https://www.aaos.org/globalassets/quality-and-practice-resources/osteoarthritis-of-the-hip/oa-hip-cpg_6-11-19.pdf>

3. Kolasinski SL, Tuhina N, Hochberg MC, Oatis C, Guyatt G, Block J et al (2020) 2019 American College of Rheumatology/Arthritis Foundation Guideline for the Management of Osteoarthritis of the Hand, Hip, and Knee. Arthritis Care Res 72(2):149-162. <https://doi.org/10.1002/acr.24131>

4. Cibulka MT, Bloom NJ, Enseki KR, Macdonald CW, Woehrle J, McDonough CM (2017) Hip Pain and Mobility Deficits – Hip Osteoarthritis: Revision 2017. J Orthop Sports Phys Ther 47(6):A1-A37. <https://doi.org/10.2519/jospt.2017.0301>

5. Deutsche Gesellschaft für Orthopädie und Unfallchirurgie e.V. (DGOU) (2021) Evidenz- und konsensbasierte Indikationskriterien zur Hüfttotalendoprothese bei Coxarthrose (EKIT-Hüfte). S3-Leitlinie der Deutschen Gesellschaft für Orthopädie und Unfallchirurgie e.V. (DGOU). <https://register.awmf.org/assets/guidelines/187-001l_S3_Indikationskriterien_H%C3%BCfttotalendoprothese_bei_Coxarthrose_2021-04.pdf>

6. Deutsche Gesellschaft für Orthopädie und Unfallchirurgie e.V. (DGOU) (2019) S2k-Leitlinie Koxarthrose.

7. Deutsche Gesellschaft für Orthopädie und Unfallchirurgie e.V. (DGOU) (2018) S2k-Leitlinie Gonarthrose.

8. Department of Veterans Affairs (DVA) (2020) VA/DoD Clinical Practice Guideline for the Non-Surgical Management of Hip & Knee Osteoarthritis. <https://www.healthquality.va.gov/guidelines/cd/oa/index.asp>

9. Bruyère O, Honvo G, Veronese N, Arden NK, Branco J, Curtis EM et al (2019) An updated algorithm recommendation for the management of knee osteoarthritis from the European Society for Clinical and Economic Aspects of Osteoporosis, Osteoarthritis and Musculoskeletal Diseases (ESCEO). Semin Arthritis Rheum 49(3):337-350. <https://doi.org/10.1016/j.semarthrit.2019.04.008>

10. Geenen R, Overman CL, Christensen R, Åsenlöf P, Capela S, Huisinga KL et al (2018) EULAR recommendations for the health professional’s approach to pain management in inflammatory arthritis and osteoarthritis. Ann Rheum Dis 77(6):797-807. <https://doi.org/10.1136/annrheumdis-2017-212662>

11. Rausch Osthoff AK, Niedermann K, Braun J, Adams J, Brodin N, Dagfinrud H et al (2018) 2018 EULAR recommendations for physical activity in people with inflammatory arthritis and osteoarthritis. Ann Rheum Dis 77(9):1251-1260. <https://doi.org/10.1136/annrheumdis-2018-213585>

12. Sakellariou G, Conaghan PG, Zhang W, Bijlsma JWJ, Boyesen P, D’Agostino MA et al (2017) EULAR recommendations for the use of imaging in the clinical management of peripheral joint osteoarthritis. Ann Rheum Dis 76(9):1484-1494. <https://doi.org/10.1136/annrheumdis-2016-210815>

13. Fernandes L, Hagen KB, Bijlsma JW, Andreassen O, Christensen P, Conaghan PG et al (2013) EULAR recommendations for the non-pharmacological core management of hip and knee osteoarthritis. Ann Rheum Dis 72(7):1125-1135. <https://doi.org/10.1136/annrheumdis-2012-202745>

14. Zhang W, Doherty M, Arden N, Bannwarth B, Bijlsma J, Gunther KP et al (2005) EULAR evidence based recommendations for the management of hip osteoarthritis: report of a task force of the EULAR Standing Committee for International Clinical Studies Including Therapeutics (ESCISIT). Ann Rheum Dis 64(5):669-681. <https://doi.org/10.1136/ard.2004.028886>

15. Jordan KM, Arden NK, Doherty M, Bannwarth B, Bijlsma JW, Dieppe P et al (2003) EULAR Recommendations 2003: an evidence based approach to the management of knee osteoarthritis: Report of a Task Force of the Standing Committee for International Clinical Studies Including Therapeutic Trials (ESCISIT). Ann Rheum Dis 62(12):1145-1155. <https://doi.org/10.1136/ard.2003.011742>

16. Jarl G, Hellstrand Tang U, Nordén E, Johannesson A, Rusaw DF (2019) Nordic clinical guidelines for orthotic treatment of osteoarthritis of the knee: A systematic review using the AGREE II instrument. Prosthet Orthot Int 43(5):556-563. <https://doi.org/10.1177/0309364619857854>

17. van Doormaal MCM, Meerhoff GA, Vliet Vlieland TPM, Peter WF (2020) A clinical practice guideline for physical therapy in patients with hip or knee osteoarthritis. Musculoskelet Care 18(4):575-595. <https://doi.org/10.1002/msc.1492>

18. Roddy E, Zhang W, Doherty M, Arden NK, Barlow J, Birrell F et al (2005) Evidence-based recommendations for the role of exercise in the management of osteoarthritis of the hip or knee – the MOVE consensus. Rheumatology (Oxford) 44(1):67-73. <https://doi.org/10.1093/rheumatology/keh399>

19. Michigan Quality Improvement Consortium Guideline (MQIC) (2021) Medical Management of Adults with Osteoarthritis. <https://www.mahp.org/wp-content/uploads/2024/03/mqicmedicalmanagementofadultswithosteoarthritisFinal2024.pdf>

20. Belo JN, Bierma-Zeinstra SMA, Raaijmakers AJ, van der Wissel F, Opstelten W (2008) Nontraumatic Knee Complaints in Adults in General Practice. Huisarts en Wetenschap 51(5):229-240. <https://repub.eur.nl/pub/17454/090701__Belo,%20Jannetje%20Neeltje.pdf>

21. National Health and Medical Research Council (NHMRC) (2018) Guideline for the management of knee and hip osteoarthritis. Second edition. <https://www.racgp.org.au/getattachment/71ab5b77-afdf-4b01-90c3-04f61a910be6/Guideline-for-the-management-of-knee-and-hip-osteoarthritis.aspx>

22. National Institute for Health and Care Excellence (NICE) (2020) Osteoarthritis: care and management. <https://www.nice.org.uk/guidance/cg177>

23. Bannuru RR, Osani MC, Vaysbrot EE, Arden NK, Bennell K, Bierma-Zeinstra SMA et al (2019) OARSI guidelines for the non-surgical management of knee, hip, and polyarticular osteoarthritis. Osteoarthr Cartil 27(11):1578-1589. <https://doi.org/10.1016/j.joca.2019.06.011>

24. Brosseau L, Taki J, Desjardins B, Thevenot O, Fransen M, Wells GA et al (2017) The Ottawa panel clinical practice guidelines for the management of knee osteoarthritis. Part two: strengthening exercise programs. Clin Rehabil 31(5):596-611. <https://doi.org/10.1177/0269215517691084>

25. Brosseau L, Taki J, Desjardins B, Thevenot O, Fransen M, Wells GA et al (2017) The Ottawa panel clinical practice guidelines for the management of knee osteoarthritis. Part three: aerobic exercise programs. Clin Rehabil 31(5):612-624. <https://doi.org/10.1177/0269215517691085>

26. Brosseau L, Wells GA, Pugh AG, Smith CA, Rahman P, Àlvarez Gallardo IC et al (2016) Ottawa Panel evidence-based clinical practice guidelines for therapeutic exercise in the management of hip osteoarthritis. Clin Rehabil 30(10):935-946. <https://doi.org/10.1177/0269215515606198>

27. Brosseau L, Wells GA, Tugwell P, Egan M, Dubouloz CJ, Casimiro L et al (2005) Ottawa panel evidence-based clinical practice guidelines for therapeutic exercises and manual therapy in the management of osteoarthritis. Phys Ther 85(9):907-971. <https://doi.org/10.1093/ptj/85.9.907>

28. Rillo O, Riera H, Acosta C, Liendo V, Bolaños J, Monterola L et al (2016) PANLAR Consensus Recommendations for the Management in Osteoarthritis of Hand, Hip, and Knee. J Clin Rheumatol 22(7):345-354. <https://doi.org/10.1097/RHU.0000000000000449>

29. Philadelphia Panel (2001) Philadelphia Panel evidence-based clinical practice guidelines on selected rehabilitation interventions for knee pain. Phys Ther 81(10):1675-1700. <https://doi.org/10.1093/ptj/81.10.1675>

30. Sellam J, Courties A, Eymard F, Ferrero S, Latourte A, Ornetti P et al (2020) Recommendations of the French Society of Rheumatology on pharmacological treatment of knee osteoarthritis. Jt Bone Spine 87(6):548-555. <https://doi.org/10.1016/j.jbspin.2020.09.004>

31. Ariani A, Manara M, Fioravanti A, Iannone F, Salaffi F, Ughi N, Prevete I et al (2019) The Italian Society for Rheumatology clinical practice guidelines for the diagnosis and management of knee, hip and hand osteoarthritis. Reumatismo 71(S1):5-21. <https://doi.org/10.4081/reumatismo.2019.1188>

32. Tuncer T, Cay FH, Altan L, Gurer G, Kacar C, Ozcakir S et al (2018) 2017 update of the Turkish League Against Rheumatism (TLAR) evidence-based recommendations for the management of knee osteoarthritis. Rheumatol Int 38(8):1315-1331. <https://doi.org/10.1007/s00296-018-4044-y>

33. Yabuki S, Ip AKK, Tam CK, Murakami T, Ushida T, Wang JH et al (2019) Evidence-Based Recommendations on the Pharmacological Management of Osteoarthritis and Chronic Low Back Pain: An Asian Consensus. AJA 57(2):37-54. <https://doi.org/10.6859/aja.201906_57(2).0003>

34. Yeap SS, Amin SRA, Baharuddin H, Koh KC, Lee JK, Lee VKM et al (2021) A Malaysian Delphi consensus on managing knee osteoarthritis. BMC Musculoskelet Disord 22:514. <https://doi.org/10.1186/s12891-021-04381-8>

35. Zhang Z, Huang C, Jiang Q, Zheng Y, Liu Y, Liu S et al (2020) Guidelines for the diagnosis and treatment of osteoarthritis in China (2019 edition). Ann Transl Med 8(19):1213. <https://doi.org/10.21037/atm-20-4665>
